# Nutrient adequacy for poor households in Africa would improve with higher income but not necessarily with lower food prices

**DOI:** 10.1038/s43016-024-00927-w

**Published:** 2024-02-21

**Authors:** Ellen B. McCullough, Meichen Lu, Yawotse Nouve, Joanne Arsenault, Chen Zhen

**Affiliations:** 1 Department of Agricultural and Applied Economics, University of Georgia, Athens, GA USA; 2 Department of Agricultural Economics, University of Kentucky, Lexington, KY USA; 3 Intake Center for Dietary Assessment, FHI 360, Washington, DC USA

**Keywords:** Economics, Development studies

## Abstract

Healthy diets are not affordable to all in Africa due to a combination of high food prices and low incomes. However, how African consumers might change demand patterns if prices or incomes were to change remains poorly understood. Using nationally representative household panel survey data from five sub-Saharan African countries, we model consumer preferences and examine how nutrient intake responds to changing food prices, total expenditures and other demand determinants. Here we find a stronger positive relationship between growth in poor consumers’ total expenditures and their nutrient intake adequacy than has been previously documented. We also find that poor consumers’ intake adequacy is especially sensitive to food staple prices in countries where one food staple dominates poor consumers’ diets. In countries with multiple food staples, no single staple’s price is a strong determinant of poor consumers’ dietary intake adequacy.

## Abstract (web summary)

Most people in sub-Saharan Africa cannot afford a nutritious diet. Using consumer demand system modelling, this study estimates diet quality sensitivity to changing income and prices of specific foods in Malawi, Niger, Uganda, Tanzania and Nigeria.

## Main

Historically, progress in improving global food security has come from both lowering food prices and raising poor consumers’ incomes. One of the most important drivers of dietary improvement, the Green Revolution, raised per capita incomes in developing countries by 50%, lowered the real prices of food staples by 35–66%, increased caloric intake in developing countries by 13.3–14.4%, and decreased child under-nourishment by 6.1–7.9% (refs. ^1,2^). The Green Revolution began later in Africa than Asia, with only 25% of food crop area planted to modern varieties and average cereal yields of 1.3 t ha^−1^ even in the late 1990s; adoption increased rapidly to 43% and yields to 1.6 t ha^−1^ by 2010 (refs. ^3,4^).

Compared with the rest of the world, improvements in nutrition have come later to Africa than to other regions, leaving sub-Saharan Africa with the lowest share of its population meeting internationally comparable diet quality standards (for example, the minimum dietary diversity standard for women)^5^. African diets are limited in intake of not just dietary energy (DE) and protein for some people, but also important micronutrients such as iron, zinc, vitamin A and folate, regardless of energy sufficiency^6^. Anaemia afflicts 39% of African women of reproductive age^6^. Altogether, the disease burden associated with nutrition deficiencies is higher in sub-Saharan Africa than anywhere else in the world, with 56 disability-adjusted life-years lost per 1,000 people in 2010 to protein and energy deficiencies and an additional 30 disability-adjusted life-years lost to micronutrient intake deficiencies^7^. The urgency of addressing nutrition deficiencies is underscored by the fact that children under the age of 5 years, 31% of whom are stunted^6^, bear over half of Africa’s hunger-related disease burden^8^. Young children experience irreversible, life-long health and economic productivity consequences as a result of undernutrition^9^.

Efforts to understand and address the challenge of undernutrition in sub-Saharan Africa have highlighted the importance of raising consumer incomes by demonstrating that nutritious diets are out of reach for most poor consumers. Diets that follow official national dietary guidelines are not affordable for an estimated 80% of people across sub-Saharan Africa^10,11^. High per calorie prices of nutrient-rich foods relative to starchy staples (SS) offer one explanation for the low affordability of good quality diets^12–15^. Many have emphasized the need to lower the prices of nutrient-rich foods, especially in Africa^16–19^. While there is widespread recognition that the cost of a healthy diet is quite large compared with poor households’ purchasing power, very little recent evidence from Africa quantifies, in a comprehensive way, how consumers alter their overall diets in response to changes in food prices^20^.

Consumer demand system modelling, exemplified by the work of Deaton and Muelbauer^21^, developed in the 1970s and flourished in the 1980s and 1990s as an important empirical application in consumer economics, allowing researchers to formally understand consumers’ preferences, test theoretical predictions about consumption behaviour and predict the impacts of policies that affect key demand determinants. A key advantage of the demand system approach is that it allows researchers to understand consumer behaviours, such as substitutions, that occur on average among population in ways that are internally consistent over time. The goal of demand system estimation is to extract a common underlying set of preferences from the observed choices of diverse individuals in a population, providing a structural explanation to support predictions of how the population’s average consumption patterns would respond to differences in prices, incomes and other factors. Demand modelling studies done in the 1980s and 1990s gave us much of our conventional knowledge about income and price elasticities in low-income countries^22–27^. Early demand modelling research mostly ignored Africa, where few household survey datasets were suitable for estimating demand models. Those early studies are not fully relevant for today’s nutritional landscape, which is characterized by the Westernization of diets, widespread fortification of food staples, and a rapid rise in consumption of packaged and processed foods^28^. Since the 1970s and 1980s, demand modelling tools have improved considerably, with more flexibility and features^29–31^. However, very few studies in Africa have incorporated these new modelling features, and none of them offer evidence from more than one African country. Furthermore, new methods and data warrant new demand system estimations, as elasticities generated using meta-analyses of prior published work are internally inconsistent^32^.

In this Article, building on consumer demand theory and careful implementation of new modelling techniques, we use a structural approach to model consumer demand using nationally representative panel data from five sub-Saharan African countries, the full set of countries for which comparable data are available (as explained with our  Methods). Our approach offers a comprehensive application, drawing on evidence from multiple countries, using a flexible functional form with respect to total expenditures and prices, allowing consumers to substitute freely between food and non-food expenditures by modelling demand conditional on total expenditures rather than total food expenditures, addressing price endogeneity and controlling for unobserved heterogeneity in consumer preferences. Using modelled consumer preferences, we quantify diet quality sensitivity to changing income and prices of specific foods, exploring how the dietary patterns of poor consumers differ from those of wealthy consumers. Using these demand system parameters, we then link macro- and micronutrient adequacy to total household expenditures and prices in ways that are directly relevant for major policies and programmes (that is, those seeking to raise consumers’ incomes or lower the prices consumers face in the marketplace). We use the model to evaluate how consumers’ diets change with very simple policy interventions that mimic the key drivers of food demand—a cash transfer (CT) designed to raise households’ total expenditures and price discounts, which would lower households’ purchase prices of specific foods.

## Modelling consumer demand

Much of what we know about the expenditure elasticity of food demand in Africa comes from estimating demand models, with more analyses focusing on Africa as data become available^33,34^. According to a meta-analysis of of 66 studies that modelled expenditure elasticities of demand for foods and nutrients across 48 African countries, African consumer demand generally follows patterns observed in other regions, with income-inelastic demand for basic staples and more elastic demand for more aspirational foods^35^. Income elasticities for the same food groups vary widely across countries, however, which begs the question of whether these differences are driven by differences in modelling methodologies, consumer attributes, or consumer preferences themselves. The vast majority (95%) of African food expenditure elasticities were estimated using cross-sectional data^35^. Our study extends the panel approach of McCullough et al. to a total of five countries^36^, holding methodological decisions constant across them, which allows us to isolate differences in either demand determinants (that is, prices, expenditures and covariates) or preferences (model parameters) as the reason for differences in demand patterns. The only other recent study to carefully estimate food expenditure elasticities in multiple African countries relies on cross-sectional data, assumes that food demand is fully separable from non-food demand, restricts the relationship between total expenditures and demand to quadratic, and does not report price elasticities of demand^15^.

In recent decades, experimental methods to measure consumer preferences have supplanted demand modelling. These methods, which include choice experiments or randomized controlled trials, experimentally induce variation in key demand determinants and then measure the demand response. Evidence from both randomized controlled trials and quasi-experiments confirms that poor consumers raise their intake of DE and their dietary diversity in response to rising incomes^37–39^. Experimental assessments of demand responses often differ from those predicted using demand models, with a demand model under-predicting food expenditure elasticities following conditional CT in Colombia and over-predicting food expenditure elasticities following an unconditional CT in Kenya^37,39^. In both cases, the authors compare experimentally derived elasticities that use pre- and post-intervention data with a cross-sectional prediction based only on pre-intervention data. Differences in observed and predicted elasticities could also arise due to misspecification of demand models, bias in demand model estimation due to to endogeneity of consumption or prices, or programme implementation resulting in a change in household preferences (for example, by targeting women).

The classic demand modelling literature, mostly from Asia, finds conflicting relationships between changing food prices and nutrient intake, even after compensating for income effects, which highlights the important substitution patterns that consumers make when prices change^40^. Studies show that poor consumers often react very differently to price changes than wealthy consumers^40^, suggesting that demand models should allow the price response to vary across the expenditure distribution. Recent evidence from Africa about consumers’ price elasticities of demand is much more scant than evidence about consumers’ expenditure elasticities of demand. McCullough et al. provide the only panel evidence of price elasticities of demand, and the only evidence that allows price elasticities to vary with total household expenditures^36^. Several prior studies report price elasticities measured using cross-sectional data from Malawi and Tanzania^33,41^. These studies find that DE, protein, and iron intake are more sensitive to maize prices than to any other prices, with rural consumers’ DE intake decreasing by 0.62% following a 1% increase in maize prices in Malawi^33^. Demand for most food groups in Tanzania is less price-elastic when estimated using panel data than when using cross-sectional data^36^. Given the scarcity of food price elasticity evidence from Africa, especially in countries with different types of diets, additional price elasticity modelling is warranted.

There is less evidence available that either models ex ante or evaluates ex post the impacts of consumer price subsidies or discounts on food demand. The literature on taxing unhealthy foods or subsidizing healthy foods is restricted to middle- and high-income countries^15^. In China, a randomly allocated food staple voucher did not improve recipients’ dietary intake of energy or nutrients, although the evidence suggests that consumers altered their demand to enhance non-nutritive aspects of their diets (for example, grain quality)^42^. When India began subsidizing pulses by incorporating them into the public distribution system, it was found to have a positive but very small effect on demand for pulses^43^. Both 10% and 25% price discounts on healthy foods in South Africa were found to increase intake of fresh fruits and whole grains but were also found to increase consumption of meat and foods that were fried or high in salt, fat and added sugar^44^.

## Results

### Diet quality and total household expenditures

We explore the relationship between total household expenditures and diet quality in two ways. First, we use the parameters of large food demand systems estimated in five sub-Saharan African countries (Malawi, Niger, Uganda, Tanzania and Nigeria) to describe intake sensitivity of dietary DE, macronutrients and micronutrients (hereafter dietary intake) to changes in total expenditures. We summarize these results according to expenditure groups (for ease of notation referred to as quartiles), which use the same cut-offs in all countries. Second, we simulate a cash CT and then examine its impact on dietary intake and on two additional per calorie measures of diet quality: macronutrient balance and the Nutrient-Rich Food Index (NRFI, a measure of a diet’s nutrient density).

#### Food demand is more expenditure elastic than in prior studies

Supplementary Fig. 1 and Supplementary Tables 1–20 depict the expenditure elasticities of demand calculated for each food group and expenditures quartile using estimated model parameters. Similar to Colen et al., we find that demand is most expenditure elastic for animal-source foods (ASF) and beverages, and less so for fruits, vegetables, nuts, fats, cereals and tubers^35^. For most food groups (staple grains (SG), SS, pulses and nuts (PN), and ASF), our average expenditure elasticity is 80–140% larger than the average elasticity reported by Colen et al. For fats and oils, our expenditure elasticities are very close, while for fruits and vegetables, our expenditure elasticities are 16% lower, and for beverages they are 10% lower. Our elasticities are also higher, by 28–58% on average per country, than those estimated by Muhammad et al. using International Comparison Program data^45^. For three additional countries where cross-sectional demand models have been carefully estimated (Malawi, Uganda and Tanzania), our expenditure elasticities are very close in Tanzania and are larger by 24% in Malawi and by 21% in Uganda^15,33^. Our larger expenditure elasticities suggest both a greater opportunity for economic growth to increase food intake and a greater risk for income shocks to result in nutrient poverty traps.

#### Nutrient intake increases with expenditure growth

Using estimated expenditure elasticities of food demand, we then calculate expenditure elasticities of intake for each macro- and micronutrient, which represent the extent to which intake would increase if household expenditures increase by 1%. Supplementary Fig. 2 shows these intake elasticities, separated by total expenditures quartile, for each country. Supplementary Tables 21–40 report these same nutrient demand elasticities by country and expenditures quartile.

Demand for DE is expenditure inelastic (that is, intake increases by less than 1% when total expenditures increase by 1%) for most consumers, except for poor consumers (Q1) in Niger, Uganda and Tanzania. Demand for carbohydrates is similar to demand for DE. Consumers show more expenditure-elastic demand for fat and for protein than for DE, with a few exceptions (demand for fat is less elastic than demand for DE in Uganda (Q3–Q4) and Nigeria (Q1–Q4). As consumers become wealthier, they generally shift food expenditures toward fat and/or protein at the expense of carbohydrates, which is consistent with improving diet quality.

Micronutrient expenditure elasticity patterns vary from country to country. Poor consumers have expenditure-elastic demand for iron in Nigeria only. They have expenditure-elastic demand for zinc everywhere except Malawi. They have expenditure-elastic demand for vitamin A in Niger, Uganda and Tanzania but not Malawi or Nigeria. Poor (Q1) consumers have expenditure-elastic demand for folate everywhere except for Nigeria. in Niger, Tanzania,

For all five countries and for all macro- and micronutrients (with the exception of vitamin A in Malawi and Niger and folate in Malawi), wealthier consumers have smaller expenditure intake elasticities than poorer consumers. Therefore, income growth results in a larger increase in macro- and micronutrient intake for poorer consumers (Q1 and Q2) than for wealthier consumers.

#### DE sufficiency rises with expenditure growth

**Fig. 1:**
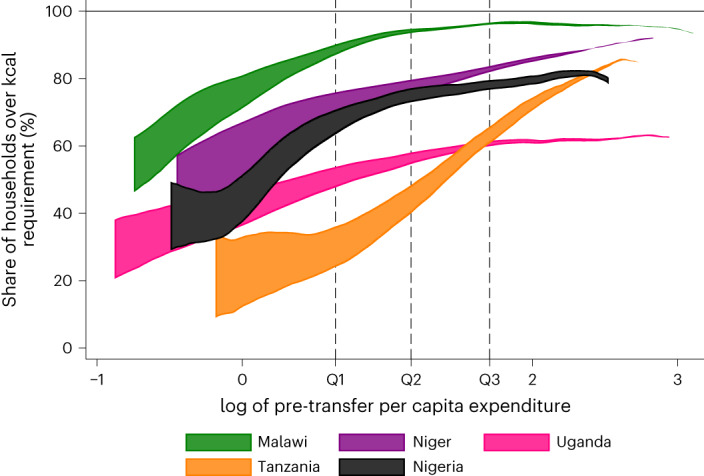
Predicted probability of DE sufficiency conditional on total household expenditures, before and after a simulated CT. The horizontal axis depicts the log of total household expenditures per adult equivalent (in US$, PPP). The vertical axis depicts the share of households with sufficient intake of DE (that is, DE intake exceeds the household’s EER). The shaded area depicts the predicted increase in the share of households with sufficient DE intake following the simulated CT. Each country’s shaded area is bounded below by the pre-transfer share of households with sufficient DE intake and above by the post-transfer share of households with sufficient DE intake. The dashed vertical lines represent the upper expenditure cut-off for each expenditure quartile, as labelled.

Figure 1 shows the relationship between total per capita expenditures and the probability that a household’s DE intake is sufficient (that is, the sum of all members’ 7-day consumption exceeds the sum of all members’ estimated energy requirements (EER)). This probability of sufficient intake increases rapidly with a rise in total household expenditures. The increase in DE sufficiency with expenditure growth is especially strong, increasing by 35 percentage points (pp) or more, in Malawi, Niger and Nigeria.

In all countries, the median Q3 and Q4 household’s intake of DE meets the EER for DE (Supplementary Fig. 3). This is not the case for Q1 households anywhere, except Malawi, or for Q2 households in Uganda or Tanzania who do not, on average, consume sufficient DE to meet household requirements. The energy requirements we used are based on individuals of average build and moderate activity levels, and even in a resource-adequate population we would expect some prevalence of DE intake that is less than EER. It is possible that energy requirements are overestimated for some wealthier households that may be less active or that individuals within wealthier households are meeting their energy needs by consuming DE outside the home. Many households that consume sufficient DE to meet the household’s total requirements do not consume sufficient micronutrients to meet the household’s total requirements.

#### CTs close intake gaps for poor consumers

**Fig. 2:**
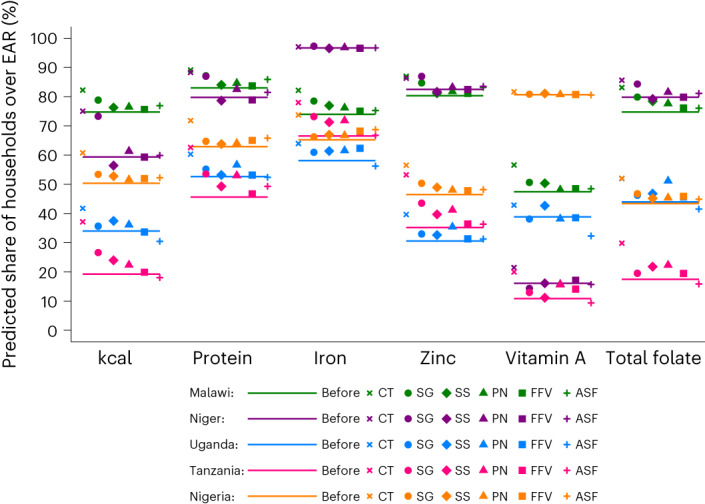
Predicted share of Q1 households with sufficient intake (exceeding the EAR) of each macro- and micronutrient before (solid line) and after the CT and each of the five PDs. The PDs are denoted by SG (which include crops like rice, maize, wheat and other cereals, millet and teff), SS (which include cassava, roots, tubers and other starches), PN, FFV and ASF (which include red meat, poultry, eggs, dairy, fish and seafood). Food group mapping into PD categories is listed country by country in Supplementary Table 62. Each country is depicted by one colour, and each symbol depicts one CT or PD simulation as represented in the legend.

**Extended Data Fig. 1:**
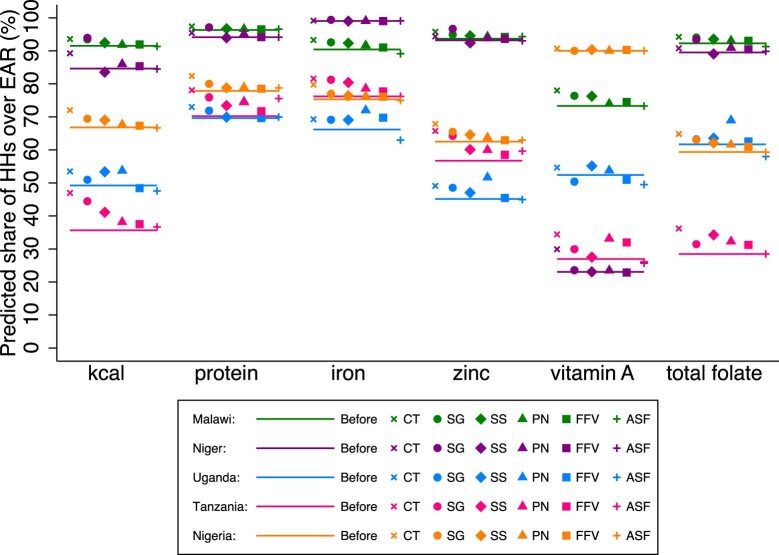
Predicted share of Q2 households with sufficient intake (exceeding the estimated average requirement (EAR)) of each macro- and micro-nutrient before (solid line) and after the cash transfer (CT) and each of the five price discounts (PDs). The PDs are denoted by SG (staple grains, which include crops like rice, maize, wheat & other cereals, millet, and teff; SS (starchy staples, which include cassava, roots, tubers, & other starches); PN (pulses, nuts, & seeds); FFV (fresh fruits & vegetables); and ASF (animal-source foods, which include red meat, poultry, eggs, dairy, fish, & seafood). Food group mapping into PD categories is listed, country by country, in Suppl. Table 62. Each country is depicted by one color, and each symbol depicts one CT or PD simulation as represented in the legend.

**Extended Data Fig. 2:**
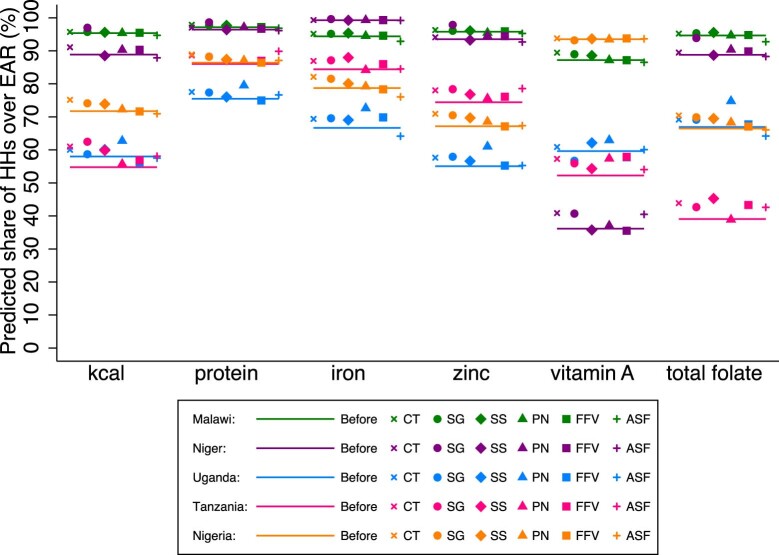
Predicted share of Q3 households with sufficient intake (exceeding the estimated average requirement (EAR)) of each macro- and micro-nutrient before (solid line) and after the cash transfer (CT) and each of the five price discounts (PDs). The PDs are denoted by SG (staple grains, which include crops like rice, maize, wheat & other cereals, millet, and teff; SS (starchy staples, which include cassava, roots, tubers, & other starches); PN (pulses, nuts, & seeds); FFV (fresh fruits & vegetables); and ASF (animal-source foods, which include red meat, poultry, eggs, dairy, fish, & seafood). Food group mapping into PD categories is listed, country by country, in Suppl. Table 62. Each country is depicted by one color, and each symbol depicts one CT or PD simulation as represented in the legend.

**Extended Data Fig. 3:**
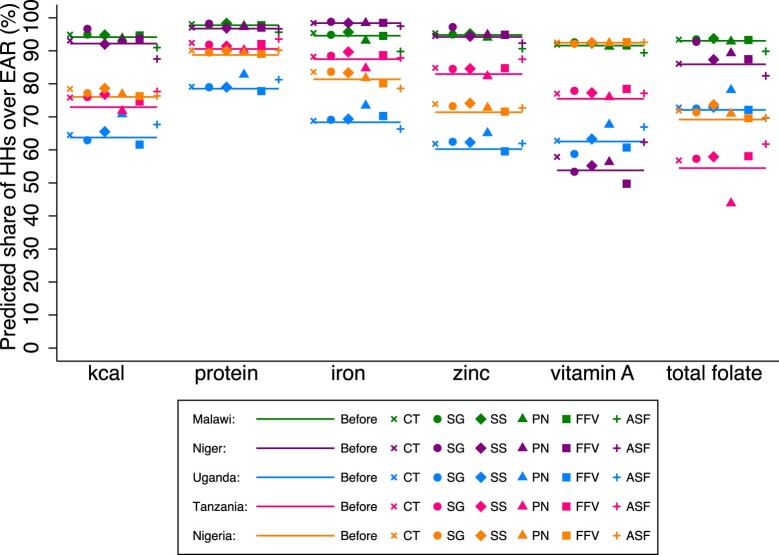
Predicted share of Q4 households with sufficient intake (exceeding the estimated average requirement (EAR)) of each macro- and micro-nutrient before (solid line) and after the cash transfer (CT) and each of the five price discounts (PDs). The PDs are denoted by SG (staple grains, which include crops like rice, maize, wheat & other cereals, millet, and teff; SS (starchy staples, which include cassava, roots, tubers, & other starches); PN (pulses, nuts, & seeds); FFV (fresh fruits & vegetables); and ASF (animal-source foods, which include red meat, poultry, eggs, dairy, fish, & seafood). Food group mapping into PD categories is listed, country by country, in Suppl. Table 62. Each country is depicted by one color, and each symbol depicts one CT or PD simulation as represented in the legend.

A CT simulation results in a large increase in the probability that extremely poor (Q1) households consume sufficient DE in all countries. Figure 2 depicts the share of Q1 households with sufficient dietary intake pre- and post-CT for each country and Extended Data Figs. 1–3 show the same for Q2–Q4 households. A CT targeting Q1 consumers would increase the share of Q1 households whose intake of DE meets the household’s estimated requirement (from 75% to 82% in Malawi, 59% to 76% in Niger, 34% to 42% in Uganda, 19% to 37% in Tanzania, and 50% to 61% in Nigeria). The effect of the CT in pushing households above the DE intake requirement is largest for the poorest households and decreases in total expenditures (Fig. 1).

A CT would raise the share of consumers with sufficient intake of key macro- and micronutrients in addition to DE. Following the CT, the share of Q1 households with sufficient protein intake increases from 45% to 61% in Tanzania, the country with the largest increase. The increase is also large in Malawi (7 pp), Niger (11 pp), Uganda (9 pp) and Nigeria (9 pp). The CT is also effective in increasing the share of poor households that have sufficient intake of iron, zinc and total folate. The only exception is iron in Niger and vitamin A in Nigeria, where pre-CT intake is already sufficient for most poor households.

#### CTs improve dietary nutrient density for the poor

CTs also improve the NRFI, our measure of overall density of nutrients per unit of DE consumed by the household. As discussed in Methods, NRFI is composed of 12 dietary components, of which 9 are positively associated with improved diet quality and 3 are negatively associated^46^. Because consumers’ diets evolve in many ways as their expenditures increase, the CT sometimes improves and sometimes worsens nutrient density (Supplementary Fig. 4). The CT improves NRFI for all but the poorest consumers in Malawi and Niger. It improves NRFI for all but the wealthiest consumers in Tanzania. It decreases NRFI for all consumers in Nigeria and all but the wealthiest consumers in Uganda.

#### CTs do not exacerbate dietary imbalance

Many Q1 consumers in all five countries consume protein in a smaller share of total DE than is recommended by the World Health Organization (WHO), a sign of dietary imbalance. Many Q1 consumers in Malawi and Tanzania also consume a larger share of carbohydrates in total DE than recommended. Fat is often under-consumed by poor consumers in Malawi, Niger and Tanzania. In Malawi, Uganda and Nigeria, dietary imbalance is common even for Q4 consumers.

**Fig. 3:**
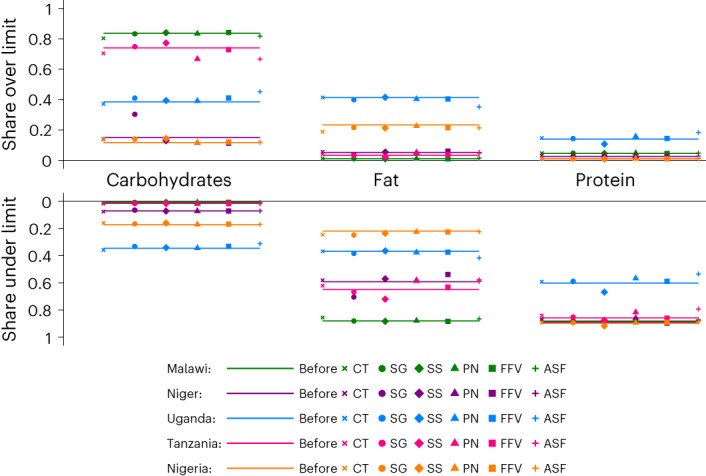
Dietary balance before and after CT and each of the five PD simulations for Q1 households. Each country is depicted by one colour, and each symbol depicts one CT or PD simulation as represented in the legend. The PD simulations are denoted by SG, SS, PN, FFV and ASF as described in Supplementary Table 62. The left column above the label depicts the share of the country’s population whose intake of carbohydrates exceeds the WHO recommended 75% limit, while the left column below the label depicts the share of the country’s population whose intake of carbohydrates is less than the recommended 55% lower limit. The solid line depicts the share of households at baseline that consume above (below) the upper (lower) recommended limit. The second column depicts the same dietary balance analysis for fat, while the right-most column is for protein.

As shown in Fig. 3, the CT improves problems of DE under-consumption for Q1 consumers in that it lowers the share of consumers who consume an excess share of carbohydrates (exceeding 75% of DE) in Malawi and Tanzania; however, it slightly increases the share of Q1 households with carbohydrate-heavy diets in Nigeria. It does not have a large impact on the share of Q1 households who under-consume fat or protein.

**Extended Data Fig. 4:**
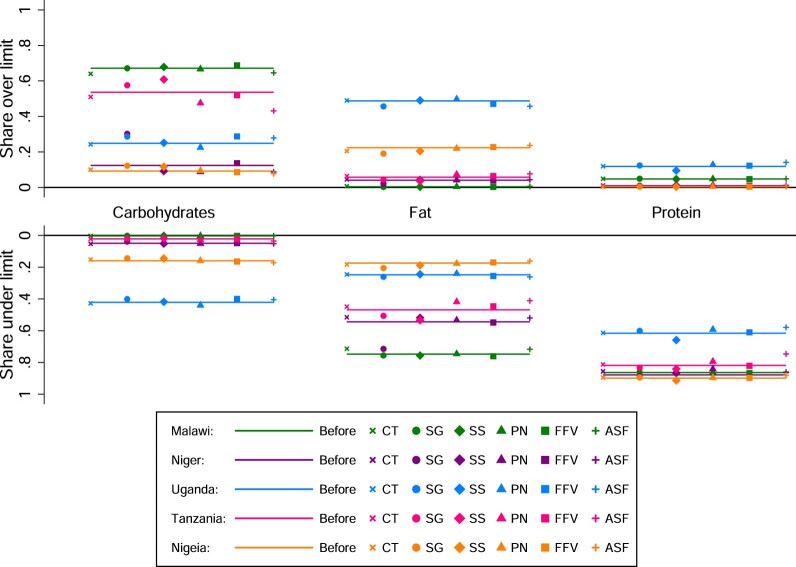
Dietary balance before (solid line) and after cash transfer (CT) and each of the five price discount (PD) simulations for Q2 households. Each country is depicted by one color, and each symbol depicts one CT or PD simulation as represented in the legend. The PD simulations are denoted by SG (staple grains) SS (starchy staples), PN (pulses, nuts, & seeds); FFV (fresh fruits & vegetables); and ASF (animal-source foods) as described in Suppl. Table 62. The left column above the label depicts the share of the country’s population whose intake of carbohydrates exceeds the WHO recommended 75% limit, while the left column below the label depicts the share of the country’s population whose intake of carbohydrates is less than the recommended 55% lower limit. The solid line depicts the share of households at baseline that consume above (below) the upper (lower) recommended limit. The second column depicts the same dietary balance analysis for fat, while the right-most column for protein.

**Extended Data Fig. 5:**
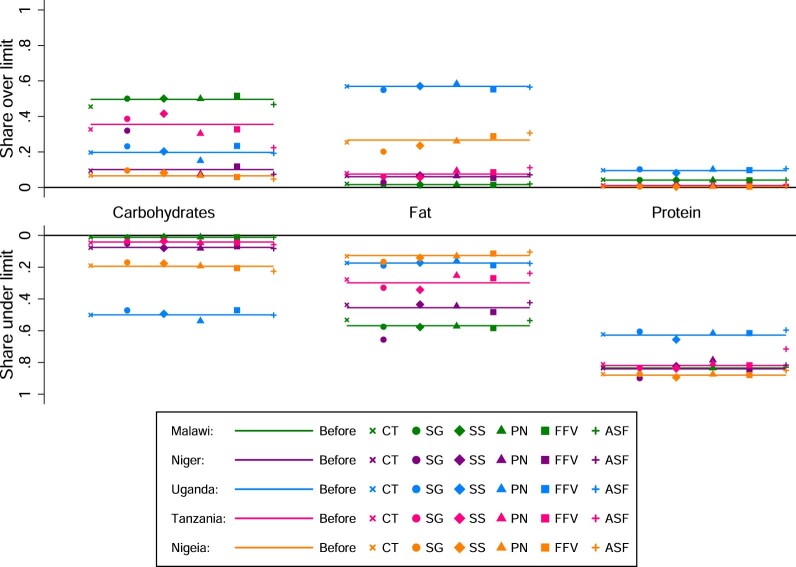
Dietary balance before (solid line) and after cash transfer (CT) and each of the five price discount (PD) simulations for Q3 households. Each country is depicted by one color, and each symbol depicts one CT or PD simulation as represented in the legend. The PD simulations are denoted by SG (staple grains) SS (starchy staples), PN (pulses, nuts, & seeds); FFV (fresh fruits & vegetables); and ASF (animal-source foods) as described in Suppl. Table 62. The left column above the label depicts the share of the country’s population whose intake of carbohydrates exceeds the WHO recommended 75% limit, while the left column below the label depicts the share of the country’s population whose intake of carbohydrates is less than the recommended 55% lower limit. The solid line depicts the share of households at baseline that consume above (below) the upper (lower) recommended limit. The second column depicts the same dietary balance analysis for fat, while the right-most column for protein.

**Extended Data Fig. 6:**
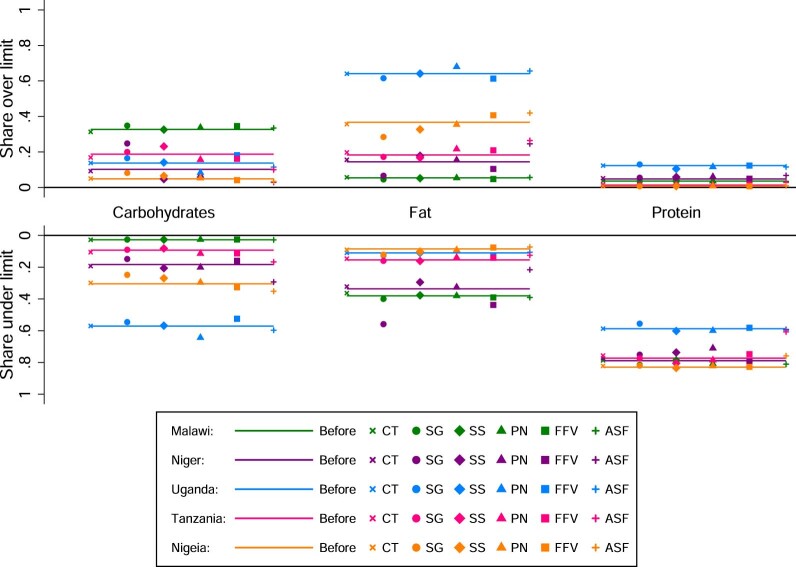
Dietary balance before (solid line) and after cash transfer (CT) and each of the five price discount (PD) simulations for Q4 households. Each country is depicted by one color, and each symbol depicts one CT or PD simulation as represented in the legend. The PD simulations are denoted by SG (staple grains) SS (starchy staples), PN (pulses, nuts, & seeds); FFV (fresh fruits & vegetables); and ASF (animal-source foods) as described in Suppl. Table 62. The left column above the label depicts the share of the country’s population whose intake of carbohydrates exceeds the WHO recommended 75% limit, while the left column below the label depicts the share of the country’s population whose intake of carbohydrates is less than the recommended 55% lower limit. The solid line depicts the share of households at baseline that consume above (below) the upper (lower) recommended limit. The second column depicts the same dietary balance analysis for fat, while the right-most column for protein.

Few Q1 households in any country exceed the upper bound of recommended fat or protein intake as a share of DE, although prevalence of fat- and protein-heavy diets in Q1 households is reduced by the CT in Niger. For Q2–Q4 households, the CT does not increase the share of households with fat- or protein-heavy diets in any country (Extended Data Figs. 4–6).

### Diet quality and food prices

When the price of one food becomes more expensive, consumers adjust demand not only for that food but also for other foods, thus affecting all three of our diet quality measures. By allowing for a flexible price response over total expenditures, our demand model does not impose by assumption that price effects are the same for wealthy and poor consumers. After we explore consumers’ price substitution patterns and their implications for determining dietary intake of macro- and micronutrients, we simulate consumers’ responses to price discounts (PDs) targeting five food categories: SG, SS, PN, fresh fruits and vegetables (FFV) and ASF.

Supplementary Tables 1–20 and Supplementary Figs. 5–9 present median price elasticities of demand by quartile and country. There is almost no comparable cross-price elasticity evidence available for us to compare our findings and there is limited own-price elasticity evidence. While differences vary across food groups and countries, our model results suggest consumers are slightly less responsive to own-price changes than previous comparable studies^15,33^.

#### Food staple prices drive intake in single-staple countries

Supplementary Figs. 10–14 depict the elasticity of each nutrient’s intake with respect to each food group’s price by country and expenditure quartile.

We see two distinct patterns in the relationship between food prices and dietary intake depending on whether any single food staple dominates a country’s diet. Single-staple sensitivity is pronounced in Malawi with maize, Niger with millet, and Tanzania with maize, which account for 34%, 31%, and 21% of Q1 households’ total expenditures, respectively (Supplementary Tables 41–45). In these countries, sensitivity of dietary intake by poor (Q1–Q2) consumers to the dominant staple’s price can be seen in a prominent vertical red band in the matrix that depicts intake elasticity with respect to food group prices (Supplementary Figs. 10, 11 and 13). A large negative intake elasticity with respect to the dominant staple’s price cuts across all (or almost all) dietary components, even those that are not concentrated in the food staple. Maize’s price dominates intake even for Q4 consumers in Niger. Micronutrient intake sensitivity to a staple food’s price has previously been documented with maize in Malawi and rice in Bangladesh^22,33^.

In countries with multiple staple foods, where no single food dominates households’ expenditures, intake is not highly sensitive to any single food’s price. In these countries, dietary intake responds to collective increases in staple food prices, but also comparably to the prices of PN and other foods. No single food group strongly determines intake in these settings. We observe this pattern in Uganda and Nigeria, where the largest food group’s budget share is 12% of poor households’ total expenditures.

#### Cross-price effects are important intake determinants

Cross-price effects help to explain why food staple prices are such strong determinants of macro- and micronutrient intake, even for nutrients that are not themselves highly concentrated in food staples. For example, in Supplementary Fig. 15 we decompose protein intake elasticities with respect to staple food prices into an own-price effect (that is, the change in protein intake arising from a change in the price of the food staple resulting from changing intake of that same food staple) and a cross-price effect (that is, the change in protein intake arising from changing intake of all other foods, apart from that food staple). Decreased consumption of complementary foods accounts for about half of the decrease in protein intake that occurs after a millet price increase in Niger or one third of the decrease in protein intake after a maize price increase in Malawi. Cross-price patterns explain a large share of the reduction of protein intake following a rice price increase in Niger, a rice price increase in Uganda, a rice or root and tuber price increase in Tanzania, or a cassava price increase in Nigeria.

Due to cross-price effects, intake of a specific macro- or micronutrient often is more sensitive to the prices of foods with low concentrations of that nutrient than it is to the prices of foods with high concentrations of that nutrient (Supplementary Figs. 16 and 17, respectively).

Because of complex patterns of substitutions and complementaries, lowering a single food’s price can result in opposing overall diet impacts in different contexts (Supplementary Fig. 18). For example, when pulses become more expensive, NRFI worsens in Malawi, Uganda and Tanzania but improves in Nigeria. When maize becomes more expensive, NRFI improves in Uganda and Tanzania, but the response varies over income quartiles in Malawi and Nigeria. Similar opposing effects are seen with the prices of cassava, vegetables, fruit and eggs. As a result, NRFI is often quite sensitive to the prices of food groups that do not have high NRFI scores. The most nutrient-dense foods are fruits and vegetables, but NRFI is not as responsive to the prices of less nutrient-dense foods (for example, PN in Nigeria and Tanzania) (Supplementary Fig. 19).

#### PDs targeting the poor close dietary intake gaps

Except in a few cases, PDs targeting any of the five categories we modelled (SG, SS, PN, FFV and ASF) would increase the share of Q1 households with sufficient DE. The exceptions include price discounts targeting SS in Niger and ASF in Nigeria, which reduce DE intake for Q1 households. For poor consumers, no single discount impacts DE intake as much as the CT (Fig. 2), but SG price discounts come close to the CT in Niger and Malawi. To increase the share of poor households with sufficient protein, discounts targeting SG and PN in Malawi, Niger and Tanzania are effective, as are discounts targeting PN in Niger and Uganda and ASF in Malawi, Tanzania and Nigeria.

No single food category offers a consistent vehicle for improving poor consumers’ intake of all micronutrients in all countries. SG discounts do not worsen intake of any nutrient in any country (except for vitamin A intake in Niger and Uganda). SS discounts do not reduce intake except for zinc and folate in Niger. PN discounts achieve at least small improvements in intake except for vitamin A in Uganda. FFV discounts also achieve at least small improvements in intake with the exception of vitamin A in Uganda. ASF discounts do not achieve large intake increases, but they decrease iron intake in Uganda and vitamin A and folate intake in Uganda and Tanzania.

Wealthier households are more likely than poor households to consume adequate macro- and micronutrients at baseline; in most cases, price discounts do not have a major impact on their sufficiency. The exceptions include discounts targeting PN in Uganda, which considerably improve Q3 and Q4 households’ sufficiency for all dietary components. Wealthier Tanzanian consumers are also responsive to SG, SS and FFV discounts. In a few cases (for example, SG discounts in Niger and PN discounts in Uganda), price discounts can increase wealthier consumers’ dietary intake more than the CT (Fig. 2). In Tanzania, the PN discounts considerably reduces the share of Q4 consumers with sufficient intake of folate.

#### Price discounts can exacerbate dietary imbalance

Because almost all price discounts targeting Q1 households increase DE intake (Fig. 2), one might be concerned that PDs could exacerbate over-consumption. At baseline, we find little evidence of over-consumption in the form of excess shares of fat or protein in DE intake, except for in Uganda and Nigeria, where fat comprises an excess share of DE intake for about 40% and 30% of households, respectively (Fig. 3). Under-consumption is the larger problem, with carbohydrates comprising an excess share of DE intake for the majority of poor households in Malawi and Tanzania. Similarly, protein comprises too small a share of DE intake for the majority of poor households all countries, as does fat in Malawi, Niger and Tanzania.

No single price discount universally improves or worsens macronutrient balance across countries. The SG discount is problematic in that it exacerbates the share of households for which carbohydrates comprise too large a share of DE everywhere except for Malawi (where carbohydrate-heavy imbalance is already above 80% before the PD). The PN and ASF discounts reduce the share of carbohydrate-heavy households in Malawi. For wealthy consumers, over-consumption of fat is high in Uganda and made worse by the PN and ASF discounts. In Niger, dietary imbalance of even Q4 consumers is exacerbated by the SG discount, which increases over-consumption of carbohydrates and under-consumption of fats. The SS discount achieves the opposite balance-improving effect for Q4 consumers in Niger.

#### PDs targeting the poor have mixed effects on diets

Many PDs improve NRFI, while some lower it. No single discount would raise NRFI for consumers in all expenditure quartiles in all countries (Supplementary Fig. 20). The SG discount would improve only wealthy households’ NRFI only in Niger, Uganda and Tanzania. The SS discount would improve NRFI for all households in Malawi and Niger and for poor households in Tanzania. The PN discount would improve NRFI in all countries across the expenditure distribution, with very small negative effects for wealthy households only in Uganda. The FFV discount would improve NRFI in most cases except for in Niger and for wealthy households in Malawi. The ASF discount would decrease NRFI for all households in Niger and Nigeria and for wealthy households in Tanzania.

#### Costs of PDs vary considerably

The redeemed value of a PD, which by assumption would reduce the consumer’s purchase price of targeted food items by 25%, varies according to how much the household would demand of the subsidized food items. We report the mean household-level value of each PD, which is based on predicted post-PD demand, in Table 1.

**Table 1:** Costs of implementing a CT programme and five different PD programmes, by country and targeted population quartile

			Malawi	Niger	Uganda	Tanzania	Nigeria
Median marginal propensity to consume CT on food (% of CT)		Q1	0.42	0.58	0.48	0.54	0.46
		Q2	0.50	0.52	0.40	0.54	0.43
		Q3	0.56	0.50	0.31	0.48	0.35
		Q4	0.43	0.47	0.17	0.38	0.17
Average monthly transfer size per household (2011 US$, PPP)		Q1–4	35.65	43.53	29.65	32.80	47.33
Median monthly cost of PD programme per household (2011 US$, PPP)	SG	Q1	13.15	24.09	2.97	8.64	11.30
		Q2	22.58	40.03	5.68	15.89	14.52
		Q3	31.83	50.97	7.32	20.69	14.69
		Q4	40.54	58.42	8.84	20.72	12.42
	SS	Q1	1.03	0	6.69	1.47	3.33
		Q2	3.22	0	12.01	3.19	7.98
		Q3	4.46	0	14.46	4.17	11.04
		Q4	5.91	1.10	14.89	4.31	11.65
	PN	Q1	2.12	0	4.01	2.10	2.29
		Q2	4.30	1.49	7.03	3.04	3.59
		Q3	5.16	2.49	8.24	3.08	4.03
		Q4	5.90	2.50	8.77	2.05	5.58
	FFV	Q1	3.98	0.77	2.34	3.27	3.90
		Q2	6.03	1.78	3.95	5.18	6.03
		Q3	7.92	3.31	5.64	7.36	7.21
		Q4	8.29	6.02	7.76	10.31	8.30
	ASF	Q1	0	0	0.98	1.14	4.83
		Q2	4.71	0.99	7.21	6.53	11.83
		Q3	15.99	3.18	13.55	13.53	18.72
		Q4	24.35	8.28	18.93	21.91	24.86

The average monthly transfer size depicts the absolute size of CT that we simulated for each household. The median marginal propensity to consume food in total is based on the share of the modelled CT that is used to increase total food expenditures (as opposed to numéraire good expenditures). The median monthly cost of each PD programme is based on the post-PD quantities consumed of the food groups contained in each category, which varies by total expenditure quartile. Supplementary Table 62 lists the food groups included in each PD category.

Ignoring implementation costs, we assume that the cost to the government of granting a price discount equals its redeemed value to beneficiary households. PDs that target poor households are almost always less costly than those targeting non-poor households, as poor households consume less food and also lower quality foods within each category than wealthier households. The only exception is PN discounts for poor households, which cost less for Q3 households in Malawi, Tanzania and Nigeria. These regressive features of a PD could be addressed by designing a programme that excludes higher quality items or places a cap on the quantity subsidized. Because our focus is on a PD’s impact on poor consumers’ food demand, we did not incorporate such design features into these simulations.

Across all food categories, Q1 price discounts are always lower cost per household than the CT, which ranges in cost from US$30 per household per month in Uganda to US$47 in Nigeria. The SG discount is by far the most expensive, costing up to US$24 per household per month for Q1 households in Niger and US$58 for Q4 households. Only in Uganda, where starchy bananas are a key staple, is a different food category discount (SS) more expensive than the SG discount. PN discounts are reasonably cost effective, with a median cost of US$4 per month per poor (Q1) household in Uganda (the highest of the countries). The PN discount is not excessively expensive for wealthy consumers either, suggesting that the cost of programme leakage in the case of imperfect targeting would not be overly burdensome. The FFV discount is also relatively low cost per Q1 household (the most expensive country average is US$3.98 per household per month in Malawi). The ASF discount is small for Q1 consumers (the highest country is Nigeria at US$4.83 per household per month) but quite expensive for wealthy consumers (topping off at US$24.86 per month per Q4 household in Malawi).

When assessing the dietary impacts of CTs and price discounts (Supplementary Fig. 20) alongside the predicted costs, PN and FFV PDs are more promising than SG or ASF discounts with regards to impacts on NRFI.

## Discussion

With recent comprehensive evidence, our findings support the widespread belief that poverty reduction is central to improving diet quality. Compared with other studies, we find a stronger link between growth in total expenditures and improvements in multiple dimensions of diet quality, and this link is especially strong for poor consumers with expenditures below the international US$1.90 per day purchasing power parity (PPP) extreme poverty line. Poor consumers associate increased expenditures very closely with increased intake of macro- and micronutrients.

We assess the diet quality impacts of poverty reduction by simulating a stylized CT that would raise consumers’ total expenditures without changing prices. We find that a CT would lead to more households consuming sufficient levels of macro- and micronutrients almost without exception. A CT would not close all intake gaps between recommended and actual intake, nor would it noticeably improve macronutrient imbalance. The simulated CT would broadly improve diets without exacerbating problems associated with over-consumption (for example, excessive intake of fat as a share of DE).

We also find evidence that food prices are important determinants of nutrient intake and diet quality. Intake is highly sensitive to the main staple food’s price in countries that rely on one major staple food (for example, Malawi, Niger and Tanzania). In multi-staple countries (for example, Uganda and Nigeria), intake is sensitive to the prices of many different foods, including SG, SS and PN.

Our results highlight the importance of understanding consumers’ complex cross-price adjustments in response to food price changes, which vary from country to country and within a country between wealthy and poor consumers. Due to these adjustments, it is difficult to predict the overall diet quality response of any food price change. There is no single food category subsidy that would improve diet quality along all dimensions for all consumers in all countries. Lowering SG prices with discounts could improve diet quality along many dimensions, athough it is also quite costly to provide poor consumers with these discounts. Lowering the prices of nutrient-dense foods like FFV and ASF can help to improve diet quality along many dimensions, although the effects are much smaller and require tradeoffs along other dimensions of diet quality. The pulse and nut discount is promising in that it results in meaningful improvements in poor consumers’ intake of macro- and micronutrients without tradeoffs, and these subsidies are not as costly to implement as other subsidies.

Our findings are consistent with the idea that healthy diets are costly relative to the purchasing power of poor consumers^14^. While many studies emphasize the high relative prices of food items that are rich in nutrients as a key barrier to diet quality^12,47^, we find that lowering the prices of healthy foods alone is unlikely to close dietary intake gaps. Rather, consumers show a strong overall propensity to improve diet quality as staple food prices are decreased and as poverty is reduced.

Due to data availability, our analysis has limitations. First, we do not observe, and thus cannot model, consumption of individuals within households. Instead, we model household intake and compare it with the household’s dietary requirements. While sufficient household intake is necessary for ensuring sufficient intake for individuals within a household, it cannot guarantee sufficient individual intake when intra-household distribution is inequitable. Second, due to survey design, we observe 7-day estimates of food consumption, which is measured with error^48^. Measurement error could potentially cause bias in coefficient estimates. Third, we do not have enough information about the content of food consumed away from home to model its contribution to dietary intake, which could be meaningful in some contexts but is unlikely to be shared by entire households so is less relevant for our assessment of household-level intake adequacy. Fourth, we do not observe processing steps such as milling or fortification, which determine the nutritive values of foods. While no survey dataset meets all desired characteristics, the Living Standards Measurement Study—Integrated Surveys on Agriculture (LSMS–ISA) surveys offer the best existing data for this research application, which emphasizes longitudinal coverage (allowing us to use longitudinal variation rather than cross-sectional variation to identify consumers’ responses to changing price and expenditures) and harmonized methods across countries (allowing us to compare results across countries holding methodology constant). Future demand modelling efforts could be improved with more consumption panel surveys offering longer food item lists, better measurement of participation by individual household members and guests in food consumption at home, and better coverage of the items, quantities, expenditures and participation consumption of food away from home.

Our simulations are designed to trace out diet quality sensitivity to the key demand drivers of total expenditures and food prices. We make important simplifying assumptions, and the simulations should not be interpreted as ex ante predictions of any programme’s full impact. We do not have a way to model what portion of a CT households may use to raise total expenditures (as opposed to accumulating assets or investing in a household enterprise). In our CT simulations, we assume that consumers use a fixed proportion of the CT to raise their total household expenditures and that there is no change in their total incomes arising from investments made with the remaining portion of the CT. These are conservative assumptions, as CTs could generate additional returns beyond what we model. CT impacts on diet quality are positive even when we ignore these additional channels through which CTs could raise total household expenditures. Fourth, we assume that any CT or PD will not result in a change in producer prices, wage rates or consumer prices (apart from the PD being simulated). Equilibrium price increases induced by a CT or PD could dampen or undermine impacts, but these price effects could be minimized if CTs and PDs are well targeted and implemented in open markets.

For policy makers and development practitioners seeking to improve diet quality in developing countries, we offer analysis of the role consumer preferences play in shaping consumers’ responses to changing price signals. Because of consumers’ complex patterns of substitutions and complementarities, our findings are counter intuitive in that lowering the relative prices of healthy foods does not necessarily offer the best intervention to raise intake of healthy foods. Staple food prices are key important determinants of overall diet quality especially in single-staple settings. Our modelling approach can assist the agricultural research and development community in prioritizing crops whose productivity gains are likely to result in the most substantial benefits for poor consumers.

## Methods

We include more details about the methods used in this study, from data to estimation to policy simulation, in Supplementary Text 1.

### Household data

We model food demand systems using Living Standards Measurement Study—Integrated Surveys on Agricultural (LSMS–ISA) nationally representative panel data from five countries: Malawi, Niger, Uganda, Tanzania and Nigeria^49^. These countries represent the full set of countries from sub-Saharan Africa for which fully nationally representative household-level panel datasets are available. While a LSMS–ISA panel dataset is also available from Ethiopia, the food item list is quite limited in two of the three survey waves, and the first wave excludes urban households, so Ethiopia is not included in this study. Table 2 reports details about each country’s survey years and sample. All samples include urban and rural households.

**Table 2:** Sample characteristics of nationally representative datasets used to model demand systems

	Malawi	Niger	Uganda	Tanzania	Nigeria
Survey rounds	2010–2011, 2012–2013, 2016–2017	2011, 2014	2005–2006, 2009–2010, 2011–2012, 2013–2014, 2015–2016, 2018–2019	2008–2009, 2010–2011, 2012–2013	2010–2011, 2012–2013, 2015–2016^a^
Unique households	3,104	3,973	3,279	3,165	4,407
Household-year observations	8,089	13,086	14,420	9,196	25,977
Food items reported (number)	58	73	47	50	74
Food groups modelled (number)	18	19	19	19	19
Share of households in Q1 (round 1)	34.16	20.99	36.93	16.40	25.72
Share of households in Q2 (round 1)	28.31	30.74	27.27	29.01	30.82
Share of households in Q3 (round 1)	21.46	30.18	21.42	31.23	28.01
Share of households in Q4 (round 1)	16.07	18.09	14.38	23.36	15.46

Quartiles (denoted Q1–Q4) divide the observations by the household’s total expenditures within each round, with Q1 representing the poorest households, which consume less than the international extreme poverty line (US$1.90 per capita per day, PPP). Q2 households represent the international poverty line (above the extreme poverty line <US$3.20 per capita per day, PPP). Q3 households are characterized by consuming above the international poverty line, namely <US$5.50 per capita per day, PPP, and Q4 households consume >US$5.50 per capita per day, PPP. In Nigeria, consumption data were collected twice within each survey rounds, so we effectively have six rounds of panel data. Household-year observations reflect the size of the pooled sample that includes all cross-sectional waves, with households observed multiple times over the survey.

Households report at-home food consumption by food item and source (purchased, self-provisioned or gift/transfer) over the 7-day period preceding the household interview. We impute the value per standard unit (hereafter, unit value) of each non-purchased item using acquisition costs. We aggregate nutritionally similar food items into 18–19 food groups, depending on the country. Supplementary Tables 46–50 list the food items included in each country’s survey and shows how they are placed into food groups. Consumption by food group is summarized in Supplementary Tables 41–45.

Many Household Consumption and Expenditure Surveys (HCES), of which LSMS–ISA datasets are one type, track household food consumption along with other topics^50^. The LSMS–ISA datasets we use offer several important advantages in their panel design, their methodological harmonization across countries, and their high quality. That said, no dataset can excel in every dimension, and LSMS–ISA data have some shortcomings. First, their item lists are not always as comprehensive as those in other types of HCES surveys. Survey designers must weigh the tradeoffs between longer item lists, which offer more comprehensive coverage within food categories^51^, generally leading to higher total reported consumption^52^, against the time required to collect additional data and its implications for respondent fatigue^53^. While surveys from sub-Saharan Africa with longer food item lists exist, the LSMS–ISA surveys used in this study offer reasonably comprehensive item coverage across many food categories, in line with those offered by many other HCES surveys from the region^51^.

We match food items to their nutritional content using food composition tables as described in Supplementary Text 1. Supplementary Tables 51–55 summarize DE intake from each food group in each country. We do not directly observe daily intake per household member, but we infer it on the basis of reported household total consumption and the household composition.

We construct a 7-day total expenditures aggregate for each household which includes total expenditures on food consumed at home as well as other expenditures, such as food consumed away from the home, alcoholic beverages and tobacco, education and health expenses, and expenditures on non-food goods and services. Food away from home consumption is included as numéraire good (that is, non-food) consumption because we do not have enough information about the items consumed to know their nutrient content or to compute unit values. Consumption of food away from home is largest in Nigeria, where it accounts for under 10% of total household expenditures^54^.

For the purpose of describing results, we use the total expenditures aggregate to partition each country’s sample into four comparable total expenditure ‘quartiles’ based on expenditures per day per adult equivalent. Children and elderly members are counted as less than one full adult equivalent, reflecting lower average levels of economic consumption. The poorest group (Q1) has expenditures below the international extreme poverty line (US$1.90 per capita per day in constant 2017 international US$, adjusted for 2011 PPP and the country’s consumer price index). Daily per capita expenditures are between US$1.90 and US$3.20 (the international poverty line) for Q2 households, between US$3.20 and US$5.50 for Q3 households, and above US$5.50 for Q4 households. Table 2 reports the share of each country’s population in each quartile.

### Prices

For each food item, we use the values per unit from purchasing households to impute unit values for non-purchasing households, as market prices are not observed for food items not purchased by households even when the items are consumed. Even if a household did not purchase an item from a market, an imputed unit value is relevant because it reflects the opportunity cost that the household would face when choosing to consume the item versus storing it or selling it.

### Statistical analyses

#### Demand model

We characterize household demand for food and a composite numéraire good (incorporating all non-food consumption goods and services) using the Exact Affine Stone Index (EASI) demand model^29^. The EASI model is an incomplete demand system, omitting demand for leisure. Consumers’ preferences for leisure relative to consumption goods dictate the tradeoff between working more to consume more goods and working less to increase leisure. Because we include a numéraire good encompassing non-food consumption, we avoid the common (yet problematic) assumption that food consumption and non-food consumption are separable.

The EASI model is much more flexible than alternatives that are more commonly used, such as the Almost Ideal Demand system or the Quadratic Almost Ideal Demand system. This flexibility allows for more curvature in the Engel curves that describe the relationship between expenditures and food demand and in the Slutsky matrix that describes the relationship between prices and demand^29^.

We estimate the specification used by McCullough et al.^36^. The dependent variable is a vector of budget shares for each food group (and one for the numéraire good). The independent variables include a vector of log price indices, the log of real total expenditures (total expenditures deflated by the Stone Price Index) and a polynomial of real total expenditures to the highest degree as selected during the estimation procedure. We also include a vector of price–expenditure interactions and demand shifters to control for observed household characteristics. Finally, we include community-level correlated random effects to control for time-invariant unobserved heterogeneity in preferences.

#### Estimation

To account for censoring (non-consumption of a food group during the 7-day recall period), we estimate latent demand using a Tobit model^30,55,56^. We impose cross-equation restrictions on the latent demand system parameters consistent with the widely acknowledged properties of a well-behaved demand system: homogeneity, symmetry and adding-up. We address two sources of endogeneity in estimation. The first source arises from the fact that each household’s own budget shares (the dependent variables in demand system) are contained within its Stone Price Index, which is part of the household’s real total expenditures variable (a regressor in the demand system). We instrument each household’s Stone Price Index with a modified index that deflates expenditures by the sample average budget share for each food group^29^.

The second source of endogeneity arises from unobserved quality heterogeneity and price search behaviour. Quality could vary systematically with total expenditures, for example, if wealthier households seek out higher quality items within a food group (for example, beef rather than goat meat within the red meat food group). Consumers could engage in price search behaviour, for example, if those who prefer an item more try harder to find better prices for that item. Several approaches have been used to address unobserved quality heterogeneity and price search behaviour. These include the regression-adjusted unit value approach^57^, structural modelling of unobserved quality preferences^58^, directly using market prices to replace unit values^59,60^ and using instrumental variables^36^. Following the approach of McCullough et al.^36^, we address quality heterogeneity within food groups by constructing a Fisher Ideal Price Index at the food group level. We address bias caused by price search behaviour by constructing price instruments for each household using neighbour households. We confirm the relevance of the price instruments in the first stage regressions, as depicted in Supplementary Tables 56–60, and we present a suggestive test for the exclusion restriction in Supplementary Table 61, with further discussion of the test and remaining concerns about price endogeneity in Supplementary Text 1.

### Food demand elasticities

We use estimated parameters to predict budget shares, expenditure elasticities and price elasticities, evaluating each price and expenditure elasticity at each household-year observation. We simulate standard errors for each elasticity by drawing parameters from a multivariate normal distribution with means equal to the parameter vector and variance equal to its covariance matrix^61^.

Supplementary Tables 1–20 report the sample-wide median price and expenditure elasticities and their standard errors by expenditure quartile for each country. Supplementary Figs. 5–9 show the own- and cross-price elasticities in matrix form for all four expenditure groups in each country.

### Nutrient demand elasticities

We derive nutrient demand elasticities using the price and expenditure elasticities described above and each food’s nutrient composition. We report the median nutrient demand elasticity of each macro- and micronutrient with respect to total expenditures and each food group’s price by country and expenditure quartile in Supplementary Tables 21–40.

### Diet quality assessment

We assess diet quality using three measures. First, intake sufficiency refers to the sufficiency of each household’s consumption of a nutrient relative to the household’s total requirement for that nutrient. To examine the intake sufficiency of each dietary component (DE, carbohydrates, protein, fat, iron, zinc, vitamin A and total folate, hereafter ‘nutrient’), we assess whether a household’s consumption of that nutrient exceeds the household-level estimated average requirement (EAR) for that nutrient, which we describe in detail in the Supplementary Text 1. Energy requirements are typically referred to as EER, but when we discuss DE requirements alongside macro- and micronutrients in the same sentence, we use the term EAR for simplicity.

Our next two diet quality measures are normalized per unit of energy intake and thus are not sensitive to misreporting of quantities consumed, assuming quantities are similarly misreported for all food items. The dietary balance measure is based on the share of carbohydrates, fat and protein in total DE intake. Imbalanced consumption of macronutrients (that is, consuming in ratios that fall outside of WHO specified ranges of 10–15% for protein, 15–30% for fat and 55–75% for carbohydrates) is predictive of chronic disease^62^. Consumers are not guaranteed low disease risk if macronutrient consumption falls outside of these ranges. Other diet-related indicators, such as including high consumption of sodium, low consumption of fruit and low consumption of whole grains and vegetables, such as inadequate fruit intake, can also predict chronic disease^63^.

The NRFI is a single diet quality measure that favours higher concentration of nine healthy components (density of protein, fibre, vitamin A, vitamin C, vitamin E, calcium, iron, magnesium and potassium) and penalizes higher concentration of three moderation components (density of saturated fat, added sugar and sodium)^46,64^.

### Policy simulations

We use policy simulations to assess the sensitivity of diet quality to changes in total expenditures and food prices. We simulate two policies: a CT of fixed size and a PD that offers a 25% price discount on various categories of foods. Both the size of the CT and the PD discount level are selected using real-world policy precedents. For each country, we select a CT size that corresponds with 20% of the median household expenditure levels of Q1 households, as depicted in Table 1. We do not adjust CT based on household size or composition. We provide more details about the CT simulation and its underlying assumptions in Supplementary Text 1.

The PD policy simulation mimics policies intended to lower the costs of healthy foods thereby encouraging their consumption^65^, or to subsidize food staple prices as a safety net intervention (for example, India’s Public Distribution System)^66^. The PD simulation could mimic the effect of raising productivity and supply of specific crops by investing in agricultural research.

We separately apply a 25% PD to five food categories (SG, SS, PN, FFV and ASF), which we map to their corresponding food groups in Supplementary Table 62. We discuss relevant PD policy precedents, our PD estimation procedures, and underlying assumptions in Supplementary Text 1.

### Simulation cost calculations

While CT costs are constant for all households in a country, the cost of each PD depends on the discount provided and the post-subsidy demand for each subsidized food. We use predicted post-PD demand, which is based on pre-PD demand and each household’s price elasticity estimates, to calculate each PD’s implementation cost, ignoring costs of programme administration.

### Reporting summary

Further information on research design is available in the Nature Portfolio Reporting Summary linked to this article.

### Supplementary information

Supplementary Information Supplementary Text 1, Figs. 1–20 and Tables 1–62.

Reporting Summary

## Data Availability

The household data we use to model demand systems are available from the World Bank’s Living Standards and Measurement Studies—Integrated Surveys in Agriculture website (https://www.worldbank.org/en/programs/lsms/initiatives/lsms-ISA). This study did not generate additional data.

## Code availability

Code to replicate all of the analysis reported in this manuscript are available through the Harvard Dataverse^67^.
