# Supplementary material for: Nutrient adequacy for poor households in Africa would improve with higher income but not necessarily with lower food prices
Source: Nat Food. 2024 Feb 21;5(2):171–81. doi: 10.1038/s43016-024-00927-w (PMC10896716; doi:10.1038/s43016-024-00927-w)
Supplement: Supplementary file 1 — Supplementary Text 1, Figs. 1–20 and Tables 1–62. [file 43016_2024_927_MOESM1_ESM.pdf]

nature food

Article https://doi.org/10.1038/s43016-024-00927-w

Nutrient adequacy for poor households in Africa would improve with higher income but not necessarily with lower food prices

In the format provided by the authors and unedited

Supplementary Information

for

Nutrient adequacy for poor households in Africa would improve with higher income but not necessarily with lower food prices

Ellen McCullough, Meichen Lu, Yawotse Nouve, Joanne Arsenault, and Chen Zhen

Contents:

Suppl. Text 1
Suppl. Figs. 1– 9
Suppl. Tables 1– 62

Suppl. Text 1: Methods Supplementary Text

Consumption

Households report food items consumed at home over the 7 full days preceding the interview, according to the item list in Tables 46–50. Food items are grouped into 18–19 food categories in each country as depicted in the tables. We use the interquartile method to clean outliers of consumption per adult equivalent at the household-item level.[1] We assign nutrient values to food items using available food composition tables from Africa,[2–7, 7] and from the USDA.[8] We assume rates of fortification of staple foods based on data in the Global Fortification Data Exchange.[9] We adjust for edible portions using information and nutrient losses in cooking from USDA.[10, 11]

Prices

To construct unit values for households that do not purchase any given food item, we follow several steps. First, we convert consumption of each food item to the most commonly reported unit for that item. Second, we use total expenditures and the total quantity purchased to create a unit value for each purchased item. Third, we clean unit value outliers by top- (bottom-) coding them at the item level to the 99th (1st) percentiles. Fourth, we impute item-level unit values for households that do not consume any market-purchased portion of an item during the 7-day recall period preceding the interview.[12] We impute these unit values for each food item and unit at the most dis-aggregated geographic level for which we have at least three unit value observations, in order to reduce the influence of unit value outliers. We start with the most dis-aggregated geographic level (the enumeration area) and then continue with the ward, district, region (interacted with urban), and then national level (interacted with urban).

Demand Model

Our two-way Exact Affine Stone Index (EASI) demand model consists of the following system of equations:

$$w^*_{hit} = \mu_i + \sum_{j=1}^{J} \alpha_{ij} p_{hjt} + \sum_{r=1}^{L} \beta_{ir} y^r_{ht} + \sum_{j=1}^{J} \alpha_{ijy}(y_{ht} \times p_{hjt}) + \sum_{k=1}^{K} \gamma_{ik} z_{hkt} + u_{hit},$$
$$(h = 1, ..., H;\ i = 1, ..., J-1;\ t = 1, ..., T). \quad (1)$$

In Equation 1, w^*_{hit} represents household h's latent budget share for food group i at time t. The household's observed budget share during the 7-day recall period, w_{hit}, is the latent budget share that is censored at zero. The

[1] In Tanzania, we retain the 1st and 99th percentile outlier cleaning approach used by McCullough *et al.*.[1]

log price index for household h and food group j at time t is denoted by p_{hjt}. The variable y_{ht} represents the log of household h's real total expenditures in period t. We construct y_{ht} as the log of total household expenditures deflated by the Stone price index: $\log x_{ht} - \sum_{j=1}^{J} w_{hjt} p_{hjt}$, where x_{ht} is nominal total household expenditures on food and non-food items [13]. J represents the total number of goods in our demand system (the number of food groups in Table 2 plus the numéraire good). H represents the total number of households in each dataset. L represents the highest degree of total expenditure polynomial included in the specification and is selected during the estimation procedure.

The vector z_{hkt} in Equation 1 represents K demand shifters used to control for observed household characteristics that explain consumption. This vector includes the household head's age, household size adjusted for adult equivalence,[2] household dependency ratio, indicators for the household head's marital status and gender, indicators for each survey wave, geographic controls for each region and for urban (vs rural) areas, and an intercept.[3] We control for time-invariant unobserved heterogeneity in tastes and preferences at the community level by including a vector of correlated random effects in z_{hkt}.[1] These effects include enumeration area (EA) level means across survey waves of both the price vector ($\overline{p_{hjt}}^{c}$) and the interaction between the price vector and real total household expenditures ($\overline{y_{ht} \times p_{hjt}}^{c}$) into z_{hkt}. Each household is linked with its corresponding EA in the first survey wave. We associate households that move away from their communities with the tastes and preferences from their originating communities.[16, 17]

By including a numéraire good in our demand model, which encompasses all non-food consumption, we avoid a common and problematic assumption that it is appropriate to model demand for food groups conditional on *total food expenditures* (as opposed to *total household expenditures*).[18, 19]

Estimation

With the cross-equation restrictions that we impose on homogeneity, symmetry and adding up, we ensure that modeled households view their incomes in real terms, respond to cross price changes symmetrically once compensated to maintain a fixed standard of living, and do not spend more than what they have. The predictive performance of a demand model is improved when these these theoretical constraints are imposed on a demand system's parameters.[20, 21]

We estimate this censored demand system with cross-equation restrictions using the extended Amemiya's generalized least squares (AGLS) estimator, which was adapted to a very similar application by Zhen *et al.*[22] Using the

---

[2] Following the literature, we adjust for adult equivalence using 1 as a weight for household members who are above age 17, 0.5 for members who are 13–17, 0.3 for members who are 7–12, 0.2 for members who are under 7.[14, 15]

[3] The demand shifters of Uganda's model do not include the indicator for the household head's gender and household dependency ratio. Continuous demand shifters are logged and demeaned in the models for consumers in Tanzania, Malawi, and Nigeria. The choice of the demand shifters and whether to log or demean the continuous variables is guided by the performance of the model based upon a series of tests to evaluate the model.

adding up restriction, we recover the parameters of the budget share equation for the numéraire good. We create a Fisher Ideal price index at the food group level in order to reduce the influence of unit value bias caused by substitution between food items within a food group (e.g., between types of red meat). For the numéraire good, we use each country's consumer price index (CPI) less food, alcoholic beverages, tobacco, and narcotics, as the price index.

One key estimation concern is that unit values can be biased by households' price search behavior and unobserved quality differences. In one approach to addressing such bias, the unit value is regressed on observed household socio-demographic characteristics, and the residualized unit value is used as the price in the demand equation.[23] This approach assumes quality preference is explained entirely by observable household characteristics, an assumption that has been questioned when comparing residualized unit values with market prices.[24] Another approach involves structural modeling of unobserved quality preferences, relying on the assumption that heterogeneity in quality preferences can be attributed entirely to income differences after controlling for other observables.[25] The elasticity of demand is recovered structurally using unit values and income elasticities. This approach has been shown to fail to fully correct unit value bias.[24, 26] Another approach calls for directly using market prices in place of unit values.[24, 26] This approach is preferred if market price data are available to the researcher and if they represent prices paid by shoppers, which could be threatened if market haggling is common or if the attributes of products for which prices are collected are not the same as those purchased by consumers.

In this study, we use the instrumental variables approach developed in McCullough *et al.*,[1] which assumes quality preferences are uncorrelated across households after controlling for observed characteristics (through correlated random effects), and an array of fixed effects. We reduce the influence of household-level price search behavior and within-item substitutions by constructing three instruments for each price index; the first uses donor households from the same survey wave and EA, the second uses donor households from the same survey wave and region, and the third uses donor households from the same geographic zone and survey month and year. For the numéraire good, the instrument is based on CPI lagged by 2 months. The identifying assumption of this instrumental variables approach enables us to use average neighboring households' price indexes as instruments. If the assumption is valid, this approach allows for more flexible preference heterogeneity than other approaches.[23, 25] Another advantage of this approach is that it does not require availability of market price data and thus can be implemented comparably across five sub-Saharan African countries.

To address concerns that the instrument approach might not sufficiently control for unobserved quality differences, leading to biased estimation of demand system parameters and incorrect conclusions from our policy simulations, we provide additional evidence in support of the exclusion restriction, following the approach of Alcott et al. (2019) and Zhen et al (2023). Using

additional variables from the datasets that could be correlated with shopping behaviors (such as a desire for quality or price search behaviors) and also could also be affected by covariate demand shocks, we show that these variables are not related to the price instruments. The additional demand variables are not closely related to the price instruments after controlling for household demand shifters and total expenditures. These results, presented in columns 1-3 of Suppl. Table 61, provide additional evidence in support of the exclusion restriction.

We also show that the price instruments are not closely related to whether households consumed food away from home, which could also threaten the exclusion restriction. Using indicators for whether anyone from the household consumed different types of food away from home within the reference period, we implemented a similar exclusion restriction test. Columns 4-8 of Suppl. Table 61 show that the price instruments did not explain consumption of food away from home, and any statistically significant coefficients were small in magnitude.

Food Demand Elasticities

In order to calculate total expenditure elasticity of demand for each food group, we take the partial derivative of a matrix formation of Eq. 1 with respect to total expenditures, $\log x$. We must account for the fact that the budget share w_i appears on both sides of the demand equation. This gives the following $J \times 1$ semi-expenditure elasticity vector, se:

$$se = (I_J + TP')^{-1} P, \tag{2}$$

where I_J is an identify matrix with dimension J, T is a $J \times 1$ vector whose i^{th} element equals $\sum_{r=1}^{L} r\beta_{ir} y^{r-1}$, and P is the $J \times 1$ vector of log prices. J represents the number of food groups in the demand system plus 1 for the numéraire good. Because the total expenditure elasticity, η_i, is a function of the semi-elasticity ($\eta_i = \frac{se_i}{w_i} + 1$), we calculate the $J \times 1$ vector of total expenditure elasticities as:

$$\eta = (diag(W))^{-1} \left[(I_J + TP')^{-1} T \right] + 1_J, \tag{3}$$

where W is the $J \times 1$ vector of observed budget shares and 1_J is a $J \times 1$ vector of ones.

We calculate predicted budget shares (i.e., conditional means of observed budget shares) and replace the observed budget shares with the predicted in the above equations to obtain expected demand elasticities.

Following Lewbel *et al.*,[13] and as discussed in more detail in McCullough *et al.*,[1] we calculate price elasticities from the partial derivatives of the demand system equation with respect to $\log p_j$. This gives the Hicksian budget share semi-elasticity of $\frac{\partial w_i}{\partial \log p_j} = \alpha_{ij} + \alpha_{ijy} y$. Because $w_i = {q_i}^H p_i / x^H$ where the superscript H emphasizes variables are compensated, we can express the

Hicksian semi-elasticity as a function of the conventional Hicksian price elasticity, so that $\frac{\partial w_i}{\partial \log p_j} = \frac{\partial ({q_i}^H p_i / x^H)}{\partial \log p_j}$, which becomes $\frac{\partial \log {q_i}^H}{\partial \log p_j} w_i + 1_{ij} w_i - w_i w_j$ with some rearrangement, where $1_{ij} = 1$ when $i = j$ and 0 otherwise [1]. The Hicksian price elasticity matrix is then $h_{ij} = \frac{\alpha_{ij} + \alpha_{ijy} y}{w_i} - 1_{ij} + w_j$.

Nutrient Demand Elasticities

We derive nutrient demand elasticities following Huang.[27, 28] First, we start with the total quantity φ consumed of each macro- and micro-nutrient arising from each food group i ($\varphi_{\gamma i}$). Hereafter we use "nutrient" as shorthand for DE and each macro- and micro-nutrient. The total intake of each nutrient is then the sum across all food groups of nutrient intake for that food group, so that $\varphi_\gamma = \sum_i \varphi_{\gamma i}$, for $i \neq J$, i.e., excluding the numéraire good good.

Each nutrient γ's content in food group i is given by:

$$\varphi_{\gamma i} = \alpha_{\gamma i} q_i(p_1, ..., p_J, y), \tag{4}$$

where $\alpha_{\gamma i}$ represents the nutrient γ content of food group i, and q_i represents the quantity demanded of food group i.

We derive nutrient demand elasticities by totally differentiating Eq. 4 with respect to prices and total expenditures, which results in

$$\frac{d\varphi_\gamma}{\varphi_\gamma} = \sum_i a_{\gamma i}\, dq_i \frac{1}{\varphi_\gamma}. \tag{5}$$

Given q_i is a function of prices (including for the numéraire good) and total expenditure y, we totally differentiate q_i with respect to prices and total expenditure to get the following formula:

$$\begin{aligned} \frac{dq_i}{q_i} &= \sum_j e_{ij} \frac{dp_j}{p_j} + \eta_i \frac{dy}{y} \\ dq_i &= \Big[\sum_j e_{ij} \frac{dp_j}{p_j} + \eta_i \frac{dy}{y}\Big] q_i, \end{aligned} \tag{6}$$

where e_{ij} indicates own- or cross-price elasticities and η_i represents total expenditure elasticities.

Substituting the expression dq_i from Eq. 6 into Eq. 5, we have the following equation:

$$\begin{aligned} \frac{d\varphi_\gamma}{\varphi_\gamma} &= \sum_i a_{\gamma i} \Big[\sum_j e_{ij} \frac{dp_j}{p_j} + \eta_i \frac{dy}{y}\Big] \frac{q_i}{\varphi_\gamma} \\ &= \sum_j \pi_{\gamma j} \frac{dp_j}{p_j} + \rho_\gamma \frac{dy}{y}, \end{aligned} \tag{7}$$

where $\pi_{\gamma j} = \sum_i a_{\gamma i} q_i \frac{e_{ij}}{\varphi_\gamma}$ is the elasticity of demand for nutrient γ with respect to price of the j th food and $\rho_\gamma = \sum_i a_{\gamma i} q_i \frac{\eta_i}{\varphi_\gamma}$ is the total expenditure elasticity of demand for nutrient γ.

Diet Quality Assessment

We construct household-level estimated average requirements (EARs) following McCullough *et al.*[1] These requirements are based on the age and gender composition of the household, assuming adults are of average weight and moderate activity levels. We take individual DE and protein requirements from FAO/WHO/UNU,[29] adjusting protein for low quality (by a factor of 75% of dietary protein) due to African diets' low reliance on animal-source protein. EARs for vitamin A, total folate and iron are from Otten *et al.*,[30] for zinc from International Zinc Nutrition Consultative Group*et al.*,[31] and for iron from Institute of Medicine (US) Panel on Micronutrients.[32] We assume low bioavailability of zinc because diets rely heavily on unrefined cereals and of iron because diets are high in phytate and low in animal-source foods.[33]

FAO and WHO requirements for DE and nutrients are based on literature that is widely used despite other sources of energy requirements. We acknowledge other recent papers have used different methods of assessing nutrient intakes for households,[34] as well as for individuals within households.[35]

In addition to our binary sufficiency variable which equals 1 if intake exceeds the household's EAR and 0 if intake is less than EAR, we also present a nutrient intake gap in some cases. This is the ratio between a household's nutrient intake and the household's requirement (EAR). When the gap is greater than one, the household's intake exceeds the EAR. When the gap is less than 1, the household's intake is that fraction of the EAR.

Policy Simulations

Social protection programs such as cash transfers are seen as an important vehicle for improving nutrition because they raise incomes for poor consumers, which is strongly associated with poverty reduction and improved food security.[36] Delivery mechanisms used in social protection programs can also affect behaviors that determine household food security, for example by empowering women, imposing conditions that reinforce desired behaviors such as school enrollment for girls, or by delivering specific food products in kind.[37]

We select a CT size following a meta-analysis of 57 evaluations conducted on 24 different social safety net programs across Africa.[38] We benchmark the CT at 20% of the median household expenditure levels of households whose per capita expenditures fall below the $1.90/day international poverty line equivalent. We present the CT size for each country in Table 1. No adjustments are made based on household size or composition. In discussing program costs, we assume there are no overhead administration or targeting costs.

Following the same meta-analysis social safety net programs Africa,[38] we assume that recipient households use 75% of the CT to increase total household expenditures (rather than, e.g., saving it or investing it in a farm or non-farm enterprise). Additional CT simulation details are presented in the Methods SM. For the sake of comparing the distributional effects of CTs, we simulate CTs not just for Q1 (poor) consumers but for all consumers. Social protection

programs often include non-poor households,[39] so CT simulations for non-poor households are also policy relevant.

When implementing the CT, we assume it does not result in any equilibrium impacts on relative prices that consumers face (for each food group and the numéraire good). There is evidence that CTs can increase prices of perishable foods in remote markets if CT program saturation rates are large.[40] We also assume that CTs would not change consumers' incomes apart from the transfer itself, either by changing the household's labor supply or wage rates, or if the household uses CT proceeds to invest in a household managed enterprise (e.g., a farm). CTs could enable investments or allocations that result in increased earnings,[41, 42] thus enhancing total impacts on household expenditures.

Next, we design a price discount (PD) policy simulation. High nutrient density foods are generally costlier per calorie than low nutrient-density foods worldwide and especially in sub-Saharan Africa, which deters poor consumers from consuming high nutrient density foods.[43] Nutritious diets are expensive relative to poor consumers' purchasing power, with the EAT Lancet diet costing about 75% of the daily mean per capita income of a sub-Saharan African household.[44] Price discounts have been used to lower the costs of, and thereby encourage consumption of, healthy foods.[45] Discounts are also used to subsidize the prices of food staples for poor consumers as a safety net intervention, such as the Targeted Public Distribution System in India.[46]

We select a PD size of 25% to align with consumer subsidy interventions that have been used to influence diet quality. Consumer price subsidies between the range of 10% and 50% have been used to encourage consumption of healthy foods like fresh fruits & vegetables, though discounts that exceed 30% are uncommon.[45] Much PD evidence comes from developed countries, where fresh fruits & vegetables are often targeted. A piloted FFV subsidy of 30% targeting SNAP beneficiaries increased FFV intake by about 26% in the USA.[47] In Sweden, a 50% subsidy of whole grain bread and breakfast cereals led to a 38% increase in fiber intake.[48] In South Africa, the HealthyFood program, which offered a 25% price discount on selected fresh fruits and vegetables and whole grains to 260,000 households increased consumption of healthy items while lowering consumption of unhealthy foods.[49] In China, staple grain PDs ranging from 8% to 25% have altered food consumption patterns.[50]

We identify the food groups in each country that fall into each PD category (staple grains, starchy staples, pulses & nuts, fruits & vegetables (FFV), and animal-source foods (ASF)), as depicted in Suppl. Table 62. In order to predict post-PD consumption, we multiply pre-PD intake of each food group by the 25% PD and the vector of own- and cross-price elasticities with respect to all of the category's subsidized food groups, thus accounting for the own-price rand cross-price effects. We then compare households' diet quality indicators with and without each PD. As with CTs, we assume that the PD does not result in any equilibrium price effects (e.g., by driving up demand for specific foods or

altering production of specific foods) or income effects (which could occur if smallholder farming households were to face different output or input prices).

Simulation Cost Calculations

In order to calculate the cost of a giving a household a price discount that targets food groups within category a with a discount of PD, we multiply the post-discount total household expenditures on food by the size of the price discount. The value to a recipient household of a discount discounting food groups in category a by 25% ($PD_a = 25\%$) follows. Ignoring administrative costs, this value to the beneficiary is the same as the cost of the discount to the government:

$$\begin{aligned} V_{PD_a} &= \sum_{i \in a} \left(1 + \sum_{j \in a} (-PD_a \cdot \varepsilon_{ij}) \right) \cdot q_i p_i \cdot PD_a \\ &= \sum_{i \in a} \left(1 + \sum_{j \in a} (-PD_a \cdot \varepsilon_{ij}) \right) \cdot w_i y \cdot PD_a \end{aligned}$$

We also calculate the marginal propensity to consume food in the aggregate for a household, as depicted in rows 9–12 of Table 1. The marginal propensity to consume food (MPC) represents the share of the cash transfer that is used to increase food consumption. V_{CT} represents the change in food expenditure that is induced by the cash transfer as a proportion of baseline food expenditure. We assume that a fixed percentage (e.g., 75%) of the cash transfer (CT) is used to raise total consumption expenditures (EI).

$$\begin{aligned} MPC_F &= V_{CT} \frac{\sum_{j \neq 20} w_j \cdot y}{CT}, \\ \text{where} \quad V_{CT} &= \frac{\partial ln(\sum_{j \neq 20} w_j y)}{\partial lny} \cdot \frac{EI}{y} = \frac{1}{\sum_{j \neq 20} w_j y} \cdot \frac{\partial (\sum_{j \neq 20} w_j y)}{\partial lny} \cdot \frac{EI}{y} \\ &= \frac{1}{\sum_{j \neq 20} w_j y} \cdot \frac{\partial (\sum_{j \neq 20} \frac{p_j q_j}{y} y)}{\partial lny} \cdot \frac{EI}{y} \\ &= \frac{1}{\sum_{j \neq 20} w_j y} \cdot \frac{EI}{y} \cdot \frac{\partial (\sum_{j \neq 20} \frac{p_j q_j}{y} y)}{\partial lny} \\ &= \frac{1}{\sum_{j \neq 20} w_j y} \cdot \frac{EI}{y} \cdot \sum_{j \neq 20} \frac{\partial q_j}{\partial lny} \frac{p_j q_j}{q_j} \\ &= \frac{1}{\sum_{j \neq 20} w_j y} \cdot \frac{EI}{y} \cdot \sum_{j \neq 20} \eta_j p_j q_j \end{aligned}$$

$$= \frac{EI}{\sum_{j \neq 20} w_j y} \cdot \sum_{j \neq 20} \eta_j w_j,$$

where CT is the size of the cash transfer in level \$US PPP, EI is the increase in total consumption expenditures due to the cash transfer, y is total pre-transfer household expenditures (in level \$US PPP) for a poor household (i.e., median across Q1 and Q2), w_j is budget share for food group j (with $j = 20$ representing the the non-food "other" expenditures), and η_j represents the total expenditure elasticity of demand for food group j.

References

[1] McCullough, E., Zhen, C., Shin, S., Lu, M., Arsenault, J.: The role of food preferences in determining diet quality for Tanzanian consumers. Journal of Development Economics **155**(March 2022), 673–711 (2021). https://doi.org/10.1016/j.jdeveco.2021.102789

[2] Lukmanji, Z., Hertzmark, E., Mlingi, N., Assey, V., Ndossi, G., Fawzi., W.: Tanzania Food Composition Tables. MUHAS-TFNC, HSPH, Dar es Salaam, Tanzania (2008)

[3] Vincent, A., Grande, F., Compaoré, E., Amponsah Annor, G., Addy, P.A., Aburime, L.C., Ahmed, D., Bih Loh, A.M., Dahdouh Cabia, S., Deflache, N., Dembélé, F.M., Dieudonné, B., Edwige, O.B., Ene-Obong, H.N., Fanou Fogny, N., Ferreira, M., Omaghomi Jemide, J., Kouebou, P.C., Muller, C., Nájera Espinosa, S., Ouattara, F., Rittenschober, D., Schönfeldt, H., Stadlmayr, B., van Deventer, M., Razikou Yiagnigni, A., Charrondière, U.R.: FAO/INFOODS Food Composition Table for Western Africa (2019) User Guide & Condensed Food Composition Table. FAO, Rome, Italy (2020)

[4] Stadlmayr, B., Charrondiere, U., Enujiugha, V., Bayili, R., Fagbohoun, E., Samb, B., Addy, P., Barikmo, I., Ouattara, F., Oshaug, A., Akinyele, I., Annor, G., Bomfeh, K., EneObong, H., Smith, I., Thiam, I., Burlingame, B.: West African Food Composition. FAO, Rome, Italy (2012)

[5] University of Ibadan: Nigeria Food Database. http://nigeriafooddata.ui.edu.ng/ Accessed Jan 10, 2022

[6] Hotz, C., Lubowa, A., Sison, C., Moursi, M., Loechl, C.: A Food Composition Table for Central and Eastern Uganda. HarvestPlus Technical Monograph. International Food Policy Research Institute (IFPRI) and International Center for Tropical Agriculture (CIAT), Washington, DC and Cali, Colombia (2012)

[7] Calloway, D., Murphy, S.: WorldFood Dietary Assessment System, 2nd

edn. University of California at Berkeley, Berkeley, CA (2006)

[8] US Department of Agriculture Agricultural Research Service: Food-Data Central. US Department of Agriculture, Washington, DC (2019). fdc.nal.usda.gov

[9] Global Fortification Data Exchange: Global Fortification Data Exchange. https://fortificationdata.org Accessed 2021-01-15

[10] Matthews, R.H., Garrison, Y.J.: Food yields summarized by different stages of preparation. Agriculture Handbook 102, US Department of Agriculture, Beltsville, MD (1975)

[11] US Department of Agriculture Agricultural Research Service: USDA Table of Nutrient Retention Factors, Release 6. US Department of Agriculture, Beltsville, MD (2007)

[12] Perali, F., Chavas, J.-P.: Estimation of censored demand equations from large cross-section data. American Journal of Agricultural Economics **82**(4), 1022–1037 (2000)

[13] Lewbel, A., Pendakur, K.: Tricks with Hicks: The EASI demand system. American Economic Review **99**(3), 827–863 (2009). https://doi.org/10.1257/aer.99.3.827

[14] Haughton, J., Khandker, S.R.: Handbook on Poverty and Inequality. World Bank Publications, Washington, DC (2009)

[15] De Janvry, A., Sadoulet, E.: Development Economics: Theory and Practice. Routledge, London, UK (2015)

[16] Atkin, D.: Trade, tastes, and nutrition in India. American Economic Review **103**(5), 1629–63 (2013)

[17] Cockx, L., Colen, L., De Weerdt, J.: From corn to popcorn? Urbanization and dietary change: Evidence from rural-urban migrants in Tanzania. World Development **110**, 140–159 (2018). https://doi.org/10.1016/j.worlddev.2018.04.018

[18] Hanemann, M., Morey, E.: Separability, partial demand systems, and consumer's surplus measures. Journal of Environmental Economics and Management **22**(3), 241–258 (1992)

[19] LaFrance, J.T., Hanemann, W.M.: The dual structure of incomplete demand systems. American Journal of Agricultural Economics **71**(2), 262–274 (1989)

[20] Kastens, T.L., Brester, G.W.: Model selection and forecasting ability of theory-constrained food demand systems. American Journal of Agricultural Economics **78**(2), 301–312 (1996)

[21] Klaiber, H.A., Holt, M.T.: The role of theoretical restrictions in forecasting with inverse demand models. American Journal of Agricultural Economics **92**(1), 70–85 (2010). https://doi.org/10.1093/ajae/aap023

[22] Zhen, C., Finkelstein, E.A., Nonnemaker, J.M., Karns, S.A., Todd, J.E.: Predicting the effects of sugar-sweetened beverage taxes on food and beverage demand in a large demand system. American Journal of Agricultural Economics **96**(1), 1–25 (2014). https://doi.org/10.1093/ajae/aat049

[23] Cox, T.L., Wohlgenant, M.K.: Prices and Quality Effects in Cross-Sectional Demand Analysis. American Journal of Agricultural Economics **68**(4), 908–919 (1986). https://doi.org/10.2307/1242137

[24] Gibson, J., Kim, B.: Quality, quantity, and spatial variation of price: Back to the bog. Journal of Development Economics **137**, 66–77 (2019). https://doi.org/10.1016/j.jdeveco.2018.11.008

[25] Deaton, A.: Quality, quantity, and spatial variation of price. The American Economic Review **78**(3), 418–430 (1988)

[26] McKelvey, C.: Price, unit value, and quality demanded. Journal of Development Economics **95**, 157–169 (2011). https://doi.org/10.1016/j.jdeveco.2010.05.004

[27] Huang, K.S.: Nutrient elasticities in a complete food demand system. American Journal of Agricultural Economics **78**(1), 21–29 (1996)

[28] Huang, K.S., Lin, B.-H.: Estimation of food demand and nutrient elasticities from household survey data. Technical Bulletin TB-1887, US Department of Agriculture, Washington, DC (2000)

[29] FAO/WHO/UNU: Human Energy Requirements: Human Energy Report of a Joint FAO/WHO/UNU Expert Consultation. FAO/WHO/UNU, Rome, Italy (2004)

[30] Institute of Medicine: Dietary Reference Intakes: The Essential Guide to Nutrient Requirements. National Academies Press, Washington, DC (2006)

[31] International Zinc Nutrition Consultative Group, Brown, K., Rivera, J., Bhutta, Z., Gibson, R., King, J., Lonnerdal, B., Ruel, M., Sandtrom, B., Wasantwisut, E., Hotz, C.: International zinc nutrition consultative group (izincg) technical document no. 1: Assessment of the risk of zinc deficiency

in populations and options for its control. Food Nutrition Bulletin **25**(1), 99–203 (2004)

[32] Institute of Medicine (US) Panel on Micronutrients: Dietary Reference Intakes for Vitamin A, Vitamin K, Arsenic, Boron, Chromium, Copper, Iodine, Iron, Manganese, Molybdenum, Nickel, Silicon, Vanadium, and Zinc. National Academies Press, Washington, DC (2002)

[33] WHO and FAO: Vitamin and Mineral Requirements in Human Nutrition, 2nd edn. World Health Organization, Geneva, Switzerland (2004)

[34] Bai, Y., Herforth, A., Masters, W.A.: Global variation in the cost of a nutrient-adequate diet by population group: an observational study. The Lancet Planetary Health **6**(1), 19–28 (2022). https://doi.org/10.1016/S2542-5196(21)00285-0

[35] Schneider, K.R., Christiaensen, L., Webb, P., Masters, W.A.: Assessing the affordability of nutrient-adequate diets. American Journal of Agricultural Economics **105**, 503–524 (2023). https://doi.org/10.1111/ajae.12334

[36] Bastagli, F., Hagen-Zanker, J., Harman, L., Barca, V., Sturge, G., Schmidt, T., Pellerano, L.: Cash Transfers: What Does the Evidence Say? Overseas Development Institute, London, UK (2016). https://cdn.odi.org/media/documents/11316.pdf

[37] Alderman, H.: Can transfer programs be made more nutrition sensitive? In: Sahn, D.E. (ed.) The Fight Against Hunger and Malnutrition: The Role of Food, Agriculture, and Targeted Policies, pp. 37–60. Oxford University Press, Oxford, UK (2015). Chap. 2

[38] Ralston, L., Andrews, C., Hsiao, A.: The impacts of safety nets in Africa: What are we learning? Policy Research Working Paper 8255, World Bank (2017). https://doi.org/10.1596/1813-9450-8255

[39] Beegle, K., Coudousel, A., Monsalve, E.: Realizing the Full Potential of Social Safety Nets in Africa. Africa Development Forum Series, Agence Française de Développement and The World Bank Group, Washington, DC, and Paris, France (2018)

[40] Filmer, D., Friedman, J., Kandpal, E., Onishi, J.: Cash Transfers, Food Prices, and Nutrition Impacts on Nonbeneficiary Children. Policy Research Working Paper 8377, World Bank (2018)

[41] Handa, S., Daidone, S., Peterman, A., Davis, B., Pereira, A., Palermo, T., Yablonski, J.: Myth-busting? Confronting six common perceptions about unconditional cash transfers as a poverty reduction strategy in Africa.

World Bank Research Observer **33**(2), 259–298 (2018). https://doi.org/10.1093/wbro/lky003

[42] Gertler, P., Martinez, S., Rubio-Codina, M.: Investing cash transfers to raise long term living standards. American Economic Journal: Applied Economics **4**(1), 164–192 (2012). https://doi.org/10.1596/1813-9450-3994

[43] Headey, D.D., Alderman, H.H.: The relative caloric prices of healthy and unhealthy foods differ systematically across income levels and continents. The Journal of Nutrition **149**, 2020–2033 (2019). https://doi.org/10.1093/jn/nxz158

[44] Hirvonen, K., Bai, Y., Headey, D., Masters, W.A.: Affordability of the EAT–Lancet reference diet: A global analysis. The Lancet Global Health **8**(1), 59–66 (2020). https://doi.org/10.1016/S2214-109X(19)30447-4

[45] Gittelsohn, J., Trude, A.C.B., Kim, H.: Availability, purchase, and consumption of healthy foods and beverages: A systematic review. Preventing Chronic Disease **14**(E107), 1–24 (2017)

[46] Kishore, A., Chakrabarti, S.: Is more inclusive more effective? The 'New Style' public distribution system in India. Food Policy **55**, 117–130 (2015). https://doi.org/10.1016/j.foodpol.2015.06.006

[47] Olsho, L.E.W., Klerman, J.A., Wilde, P.E., Bartlett, S.: Financial incentives increase fruit and vegetable intake among Supplemental Nutrition Assistance Program participants: A randomized controlled trial of the USDA Healthy Incentives Pilot. American Journal of Clinical Nutrition **104**(2), 423–435 (2016). https://doi.org/10.3945/ajcn.115.129320

[48] Nordström, J., Thunström, L.: The impact of tax reforms designed to encourage healthier grain consumption. Journal of Health Economics **28**(3), 622–634 (2009). https://doi.org/10.1016/j.jhealeco.2009.02.005

[49] An, R., Patel, D., Segal, D., Sturm, R.: Eating better for less: A national discount program for healthy food purchases in South Africa. American Journal of Health Behavior **37**(1), 56–61 (2013). https://doi.org/10.5993/AJHB.37.1.6

[50] Jensen, R.T., Miller, N.H.: Do consumer price subsidies really raise nutrition? Review of Economics and Statistics **93**(4), 1205–1223 (2010). https://doi.org/10.1162/REST_a_00118

Supplementary Figures

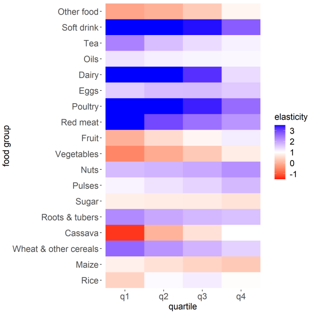

(a) Malawi

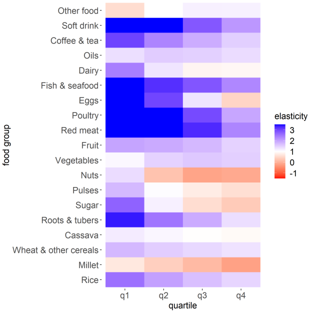

(b) Niger

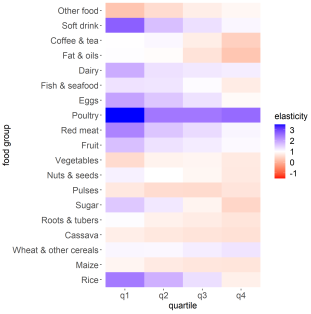

(c) Uganda

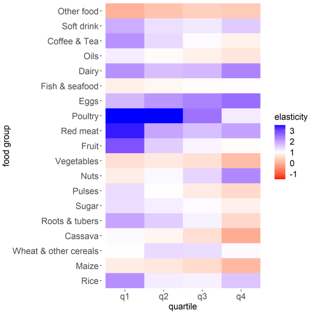

(d) Tanzania

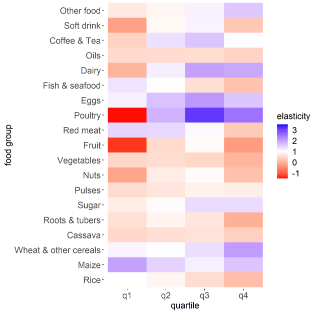

(e) Nigeria

Suppl. Fig. 1: Expenditure elasticities of food demand, separated by total expenditure quartile, in (a) Malawi, (b) Niger, (c) Uganda, (d) Tanzania, and (e) Nigeria. Each cell's color depicts the median expenditure elasticity of demand for the corresponding food group (row) and total expenditure quartile (column).

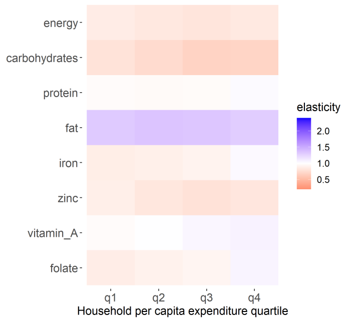

(a) Malawi

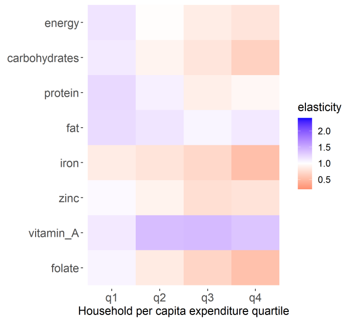

(b) Niger

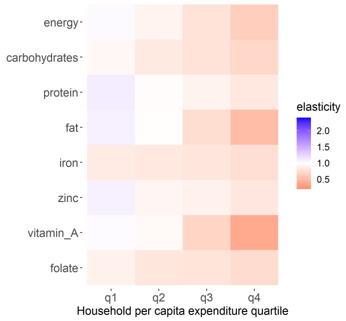

(c) Uganda

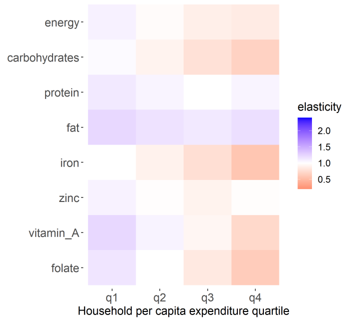

(d) Tanzania

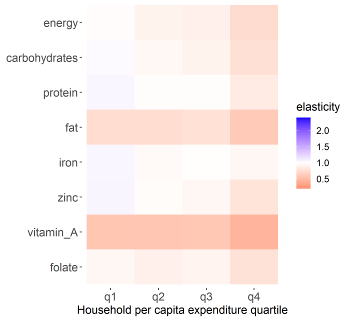

(e) Nigeria

Suppl. Fig. 2: Expenditure elasticities of demand for each macro- and micro-nutrient, by total expenditure quartile, in Malawi (a), Niger (b), Uganda (c), Tanzania (d), and Nigeria (e). Each cell's color depicts the median expenditure elasticity of demand for the corresponding nutrient (row) and total expenditure quartile (column).

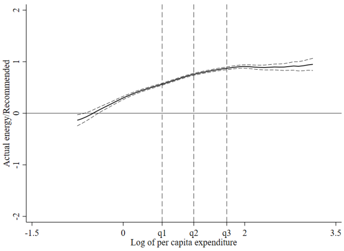

(a) Malawi

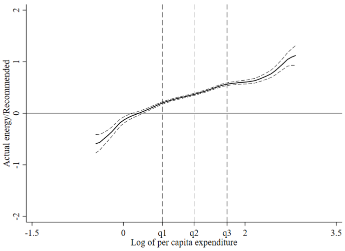

(b) Niger

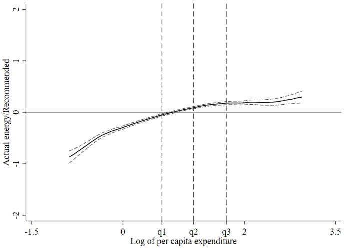

(c) Uganda

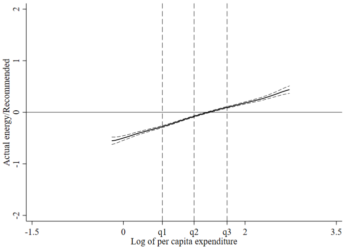

(d) Tanzania

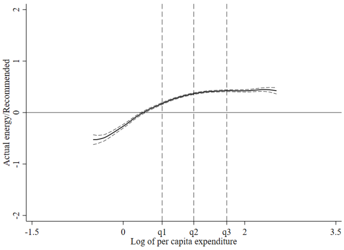

(e) Nigeria

Suppl. Fig. 3: Dietary energy expansion path. The horizontal axis depicts the log of total household expenditures per adult equivalent. The vertical dashed lines mark the upper bound of each consumer total expenditure quartile. The vertical axis depicts the log of dietary energy intake as a share of the household's total dietary energy requirement (which is specific to each household's composition).

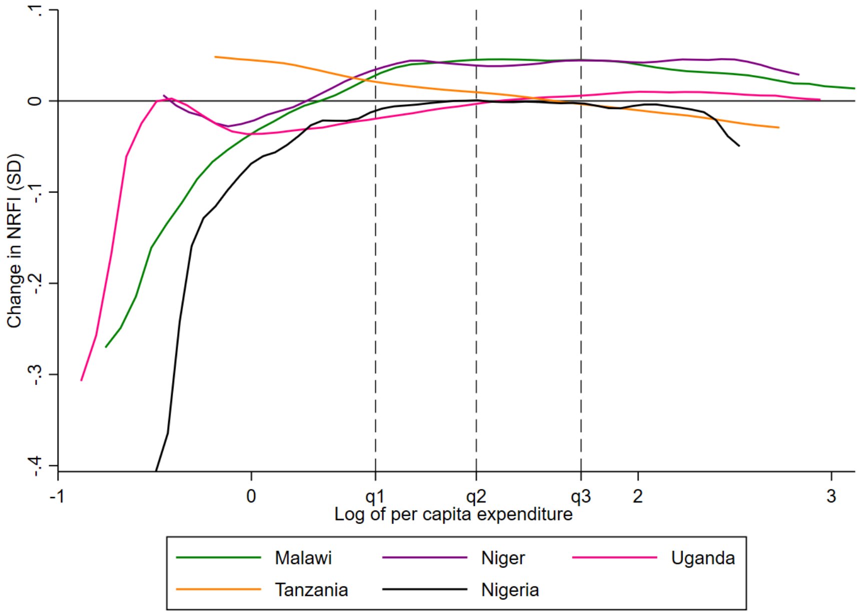

Suppl. Fig. 4: NRFI response to a simulated cash transfer. The horizontal axis depicts the log of total household expenditures per adult equivalent. The vertical dashed lines mark the upper bound of each consumer total expenditure quartile. The vertical axis depicts the predicted change in NRFI after a CT. NRFI is standardized within each country, so an increase of NRFI by 0.05 corresponds with a one-twentieth of a standard deviation increase in NRFI.

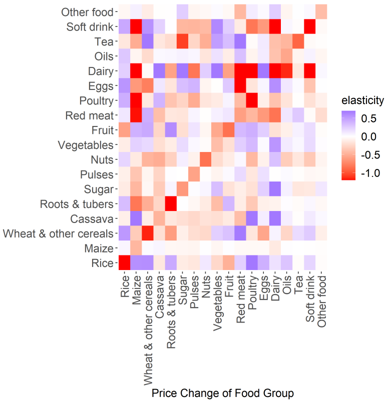

(a) Q1

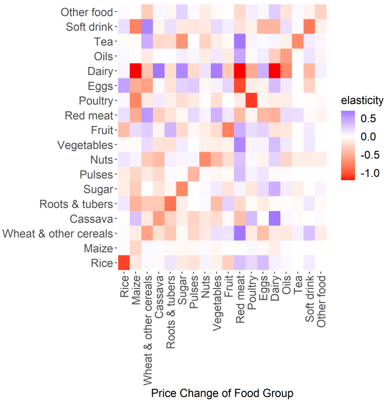

(b) Q2

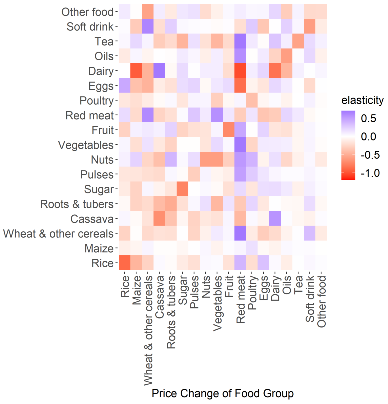

(c) Q3

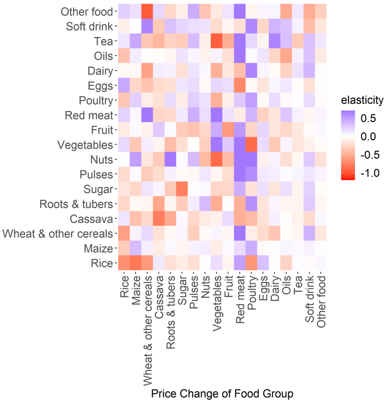

(d) Q4

Suppl. Fig. 5: Malawi: median elasticities of demand for each food group with respect to each food group's price separated by consumer total expenditure quartile: (a) Q1, (b) Q2, (c) Q3, and (d) Q4. Each cell's color depicts the median elasticity of demand for its corresponding row's food group with respect to a price change in the corresponding column's food group.

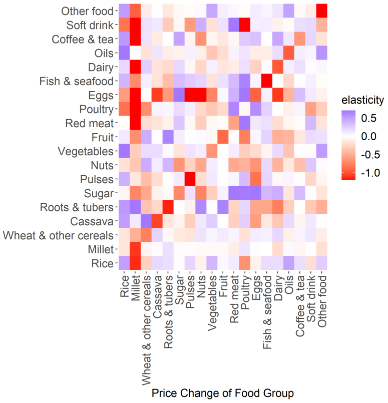

(a) Q1

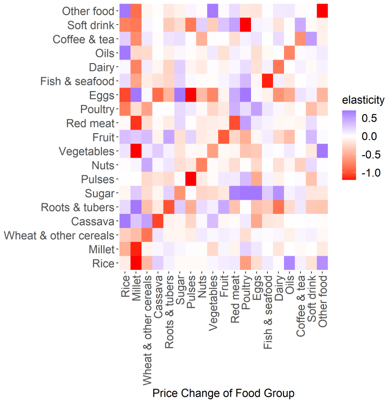

(b) Q2

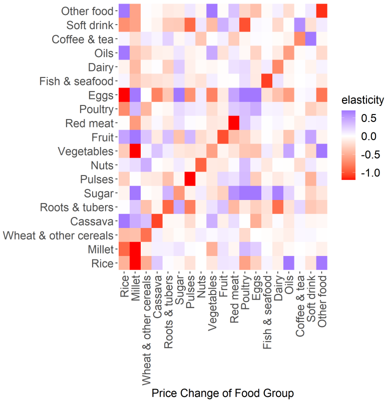

(c) Q3

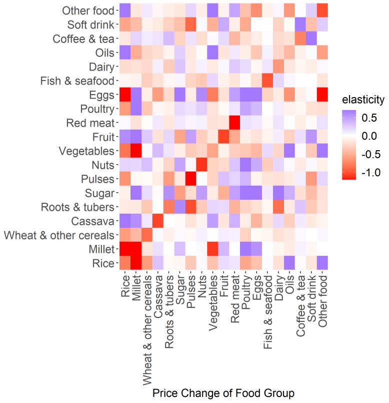

(d) Q4

Suppl. Fig. 6: Niger: median elasticities of demand for each food group with respect to each food group's price separated by consumer total expenditure quartile: (a) Q1, (b) Q2, (c) Q3, and (d) Q4. Each cell's color depicts the median elasticity of demand for its corresponding row's food group with respect to a price change in the corresponding column's food group.

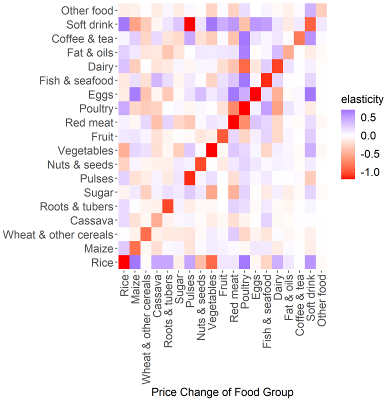

(a) Q1

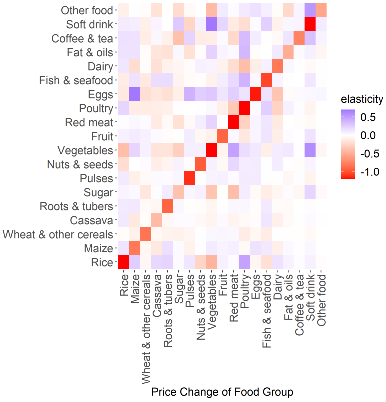

(b) Q2

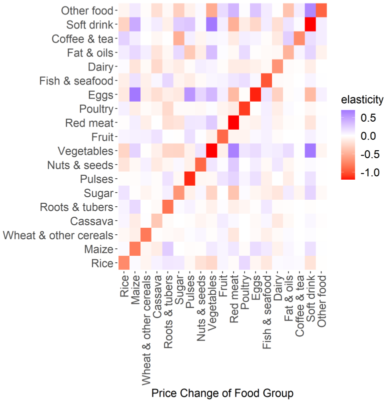

(c) Q3

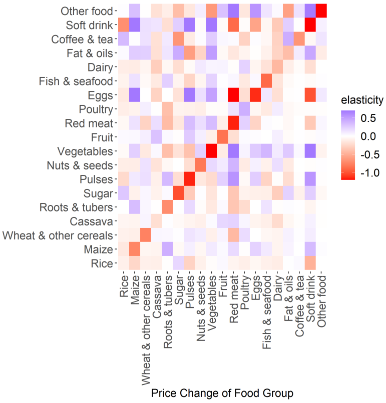

(d) Q4

Suppl. Fig. 7: Uganda: median elasticities of demand for each food group with respect to each food group's price separated by consumer total expenditure quartile: (a) Q1, (b) Q2, (c) Q3, and (d) Q4. Each cell's color depicts the median elasticity of demand for its corresponding row's food group with respect to a price change in the corresponding column's food group.

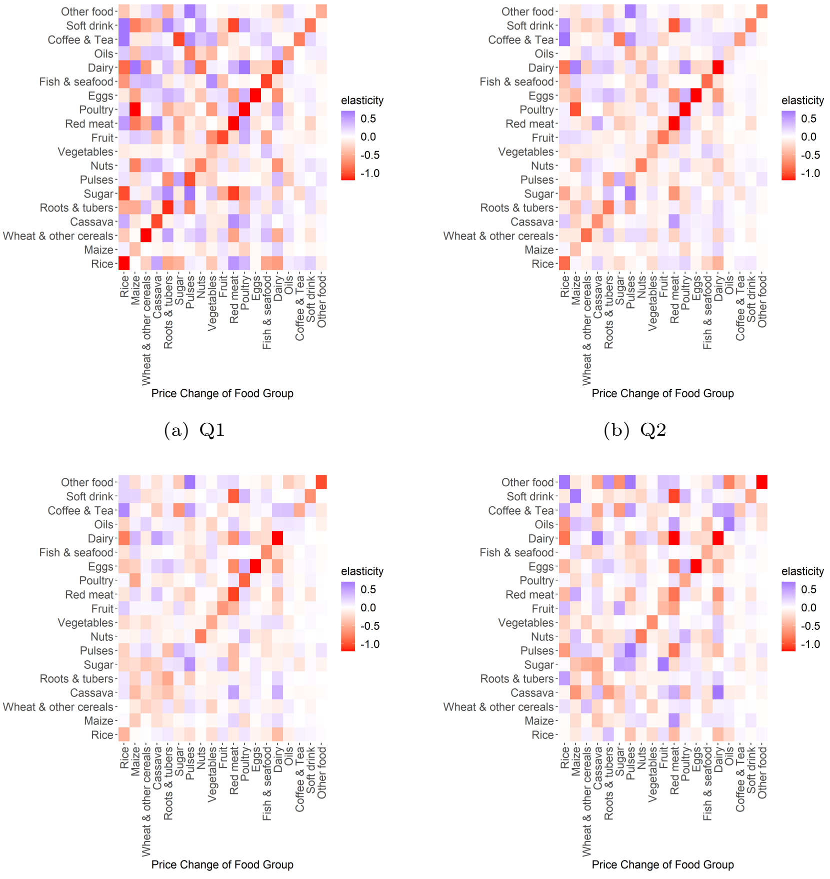

(a) Q1

(b) Q2

(c) Q3

(d) Q4

Suppl. Fig. 8: Tanzania: median elasticities of demand for each food group with respect to each food group's price separated by consumer total expenditure quartile: (a) Q1, (b) Q2, (c) Q3, and (d) Q4. Each cell's color depicts the median elasticity of demand for its corresponding row's food group with respect to a price change in the corresponding column's food group.

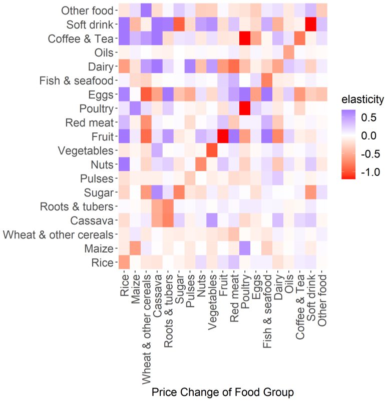

(a) Q1

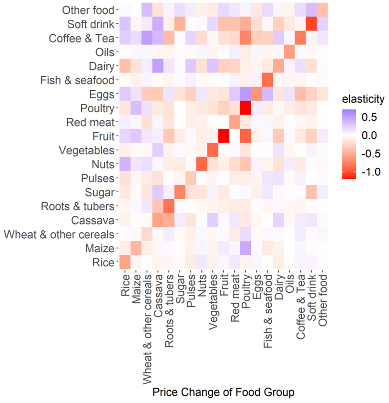

(b) Q2

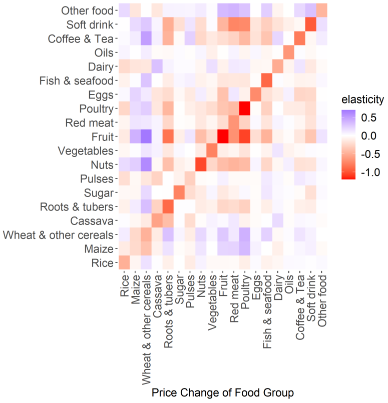

(c) Q3

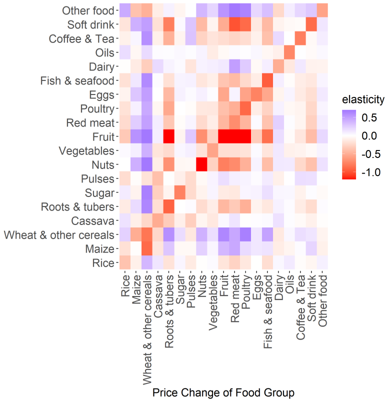

(d) Q4

Suppl. Fig. 9: Nigeria: median elasticities of demand for each food group with respect to each food group's price separated by consumer total expenditure quartile: (a) Q1, (b) Q2, (c) Q3, and (d) Q4. Each cell's color depicts the median elasticity of demand for its corresponding row's food group with respect to a price change in the corresponding column's food group.

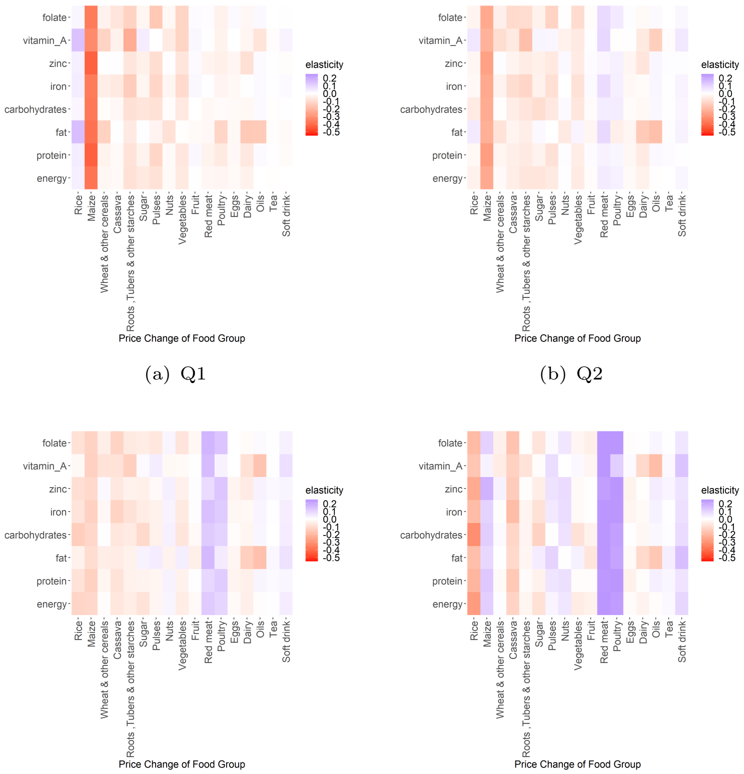

(a) Q1

(b) Q2

(c) Q3

(d) Q4

Suppl. Fig. 10: Malawi: elasticities of demand for each macro- and micro-nutrient (including DE) with respect to each food group's price, for consumers in each expenditure quartile: (a) Q1, (b) Q2, (c) Q3, and (d) Q4. Each cell's color depicts the median elasticity of demand for the corresponding nutrient (row) with respect to the price of the good depicted in each column.

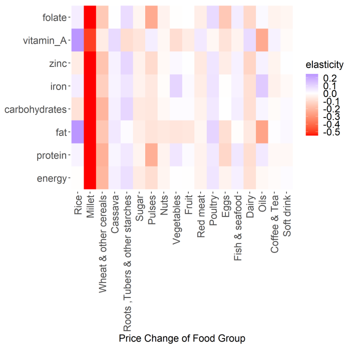

(a) Q1

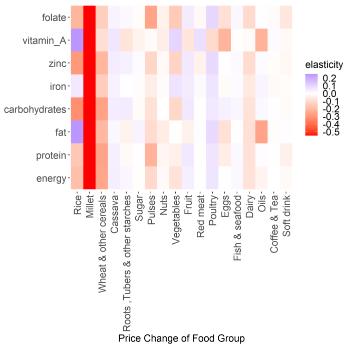

(b) Q2

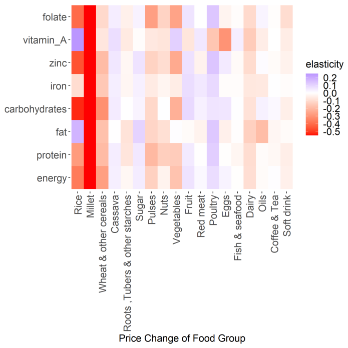

(c) Q3

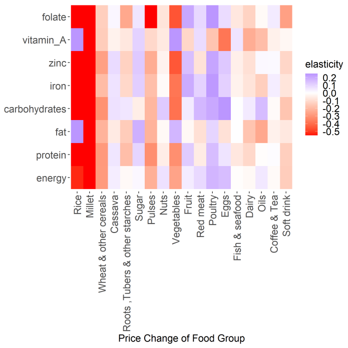

(d) Q4

Suppl. Fig. 11: Niger: elasticities of demand for each macro- and micronutrient (including DE) with respect to each food group's price, for consumers in each expenditure quartile: (a) Q1, (b) Q2, (c) Q3, and (d) Q4. Each cell's color depicts the median elasticity of demand for the corresponding nutrient (row) with respect to the price of the good depicted in each column.

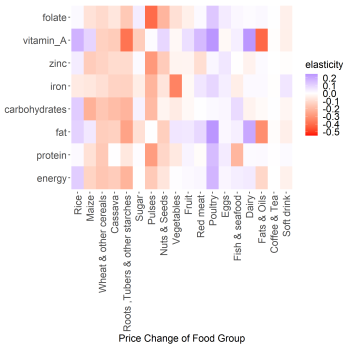

(a) Q1

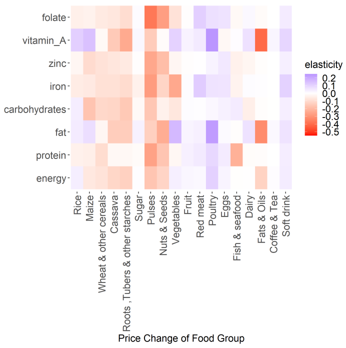

(b) Q2

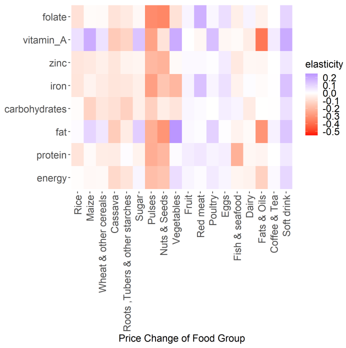

(c) Q3

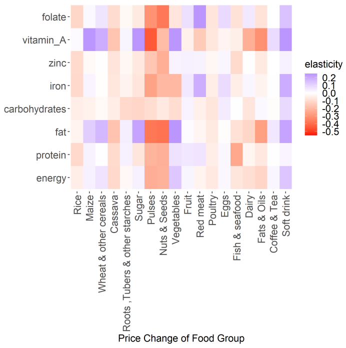

(d) Q4

Suppl. Fig. 12: Uganda: elasticities of demand for each macro- and micro-nutrient (including DE) with respect to each food group's price, for consumers in each expenditure quartile: (a) Q1, (b) Q2, (c) Q3, and (d) Q4. Each cell's color depicts the median elasticity of demand for the corresponding nutrient (row) with respect to the price of the good depicted in each column.

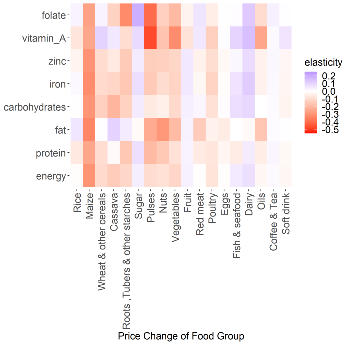

(a) Q1

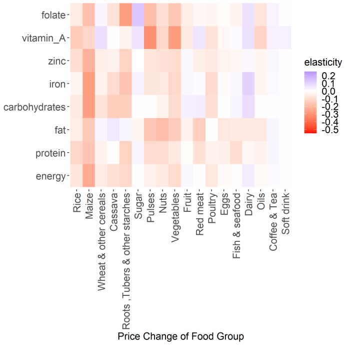

(b) Q2

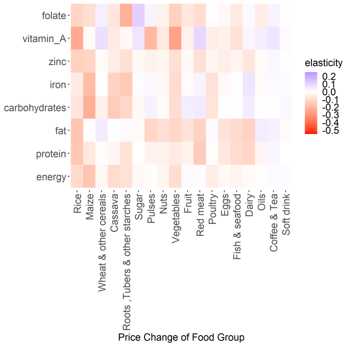

(c) Q3

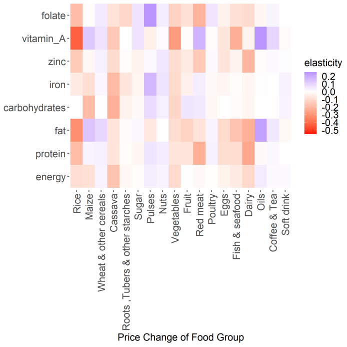

(d) Q4

Suppl. Fig. 13: Tanzania: elasticities of demand for each macro- and micro-nutrient (including DE) with respect to each food group's price, for consumers in each expenditure quartile: (a) Q1, (b) Q2, (c) Q3, and (d) Q4. Each cell's color depicts the median elasticity of demand for the corresponding nutrient (row) with respect to the price of the good depicted in each column.

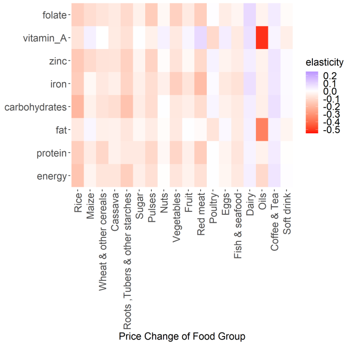

(a) Q1

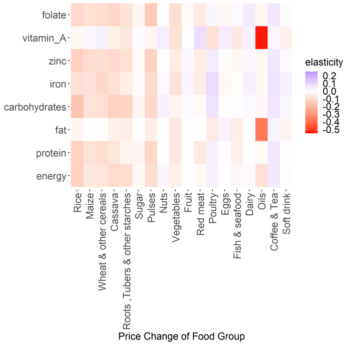

(b) Q2

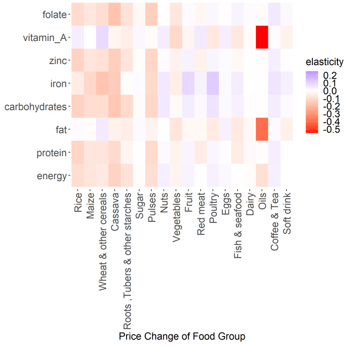

(c) Q3

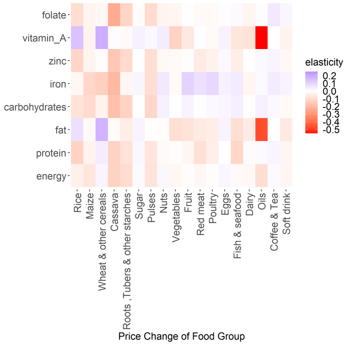

(d) Q4

Suppl. Fig. 14: Nigeria: elasticities of demand for each macro- and micronutrient (including DE) with respect to each food group's price, for consumers in each expenditure quartile: (a) Q1, (b) Q2, (c) Q3, and (d) Q4. Each cell's color depicts the median elasticity of demand for the corresponding nutrient (row) with respect to the price of the good depicted in each column.

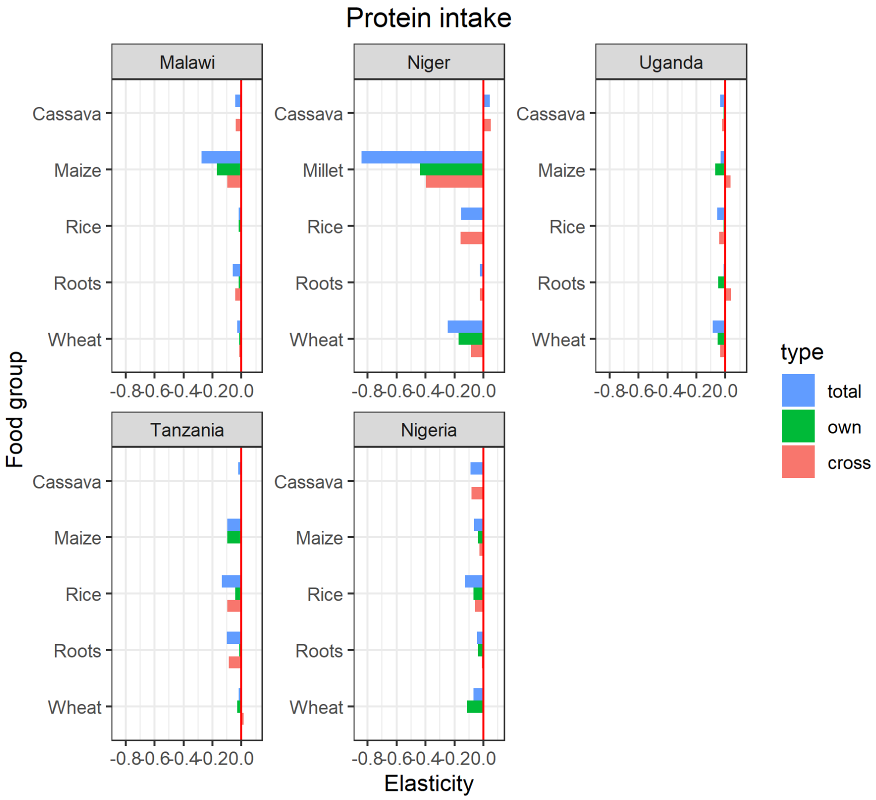

Suppl. Fig. 15: Decomposition of the total elasticity of protein intake with respect to the price of each food staple (blue bar labeled “total”) into two effects: an own effect (green bar labeled “own,” which reflects a change in protein intake arising from a change in intake of that same food staple) and a cross-effect (red bar labeled “cross,” which reflects a change in protein intake arising from a change in intake of all other foods). The food group "wheat and other cereals" is represented by the label "wheat," and the food group "roots, tubers & other starches" is represented by the label "roots." Cassava is excluded from this group as it is represented by its own food group. Items contained in each food group are listed by country in Suppl. Tables 46–60.

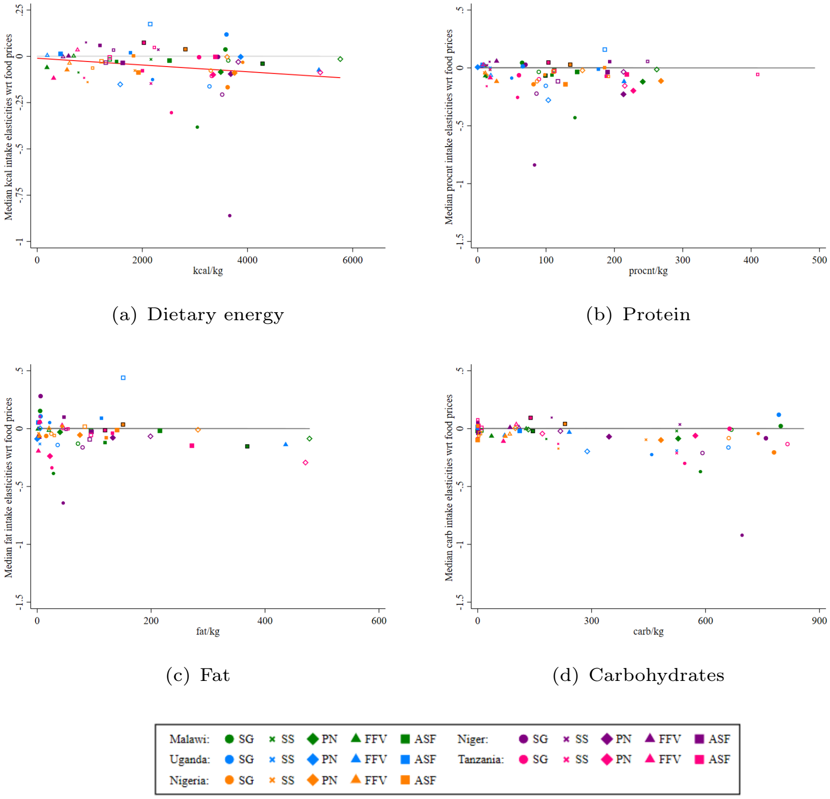

(a) Dietary energy

(b) Protein

(c) Fat

(d) Carbohydrates

Suppl. Fig. 16: Visualization of the importance of each food's price in determining dietary intake versus that foods' dietary composition. Each panel depicts a scatter plot between elasticity of the macro-nutrient intake with respect to each food group's concentration of that nutrient (quantity per kg). We categorize food groups into five categories: staple grains, starchy staples, pulses & nuts, fruits & vegetables, and animal-source foods, with food groups from each category represented by one common symbol.

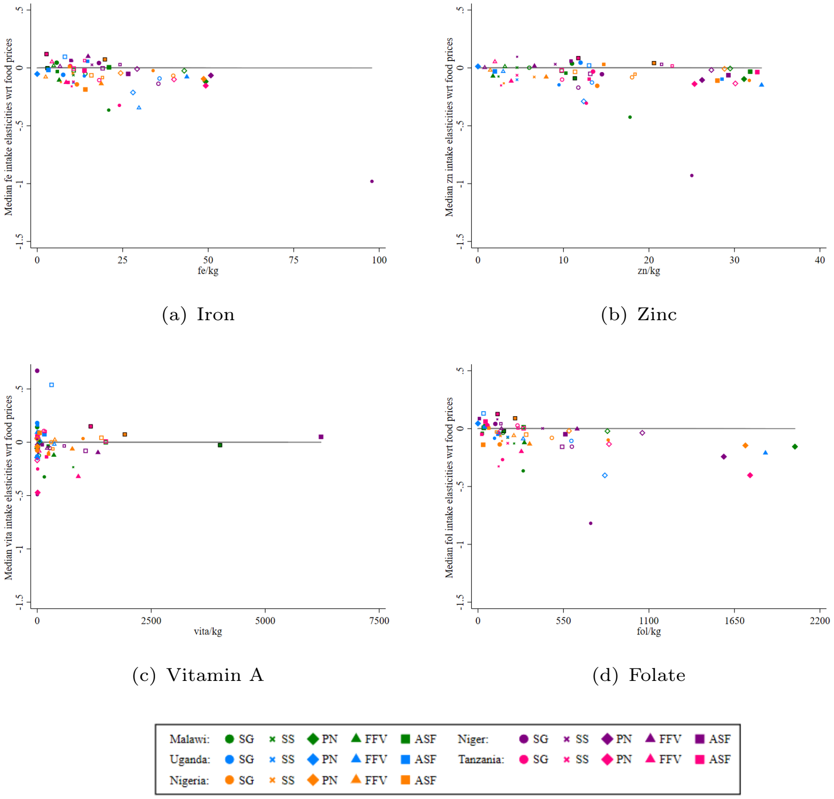

Suppl. Fig. 17: Visualization of the importance of each food's price in determining dietary intake versus that foods' dietary composition. Each panel depicts a scatter plot between elasticity of the micro-nutrient intake with respect to each food group's concentration of that nutrient (quantity per kg). We categorize food groups into five categories: staple grains, starchy staples, pulses & nuts, fruits & vegetables, and animal-source foods, with food groups from each category represented by one common symbol.

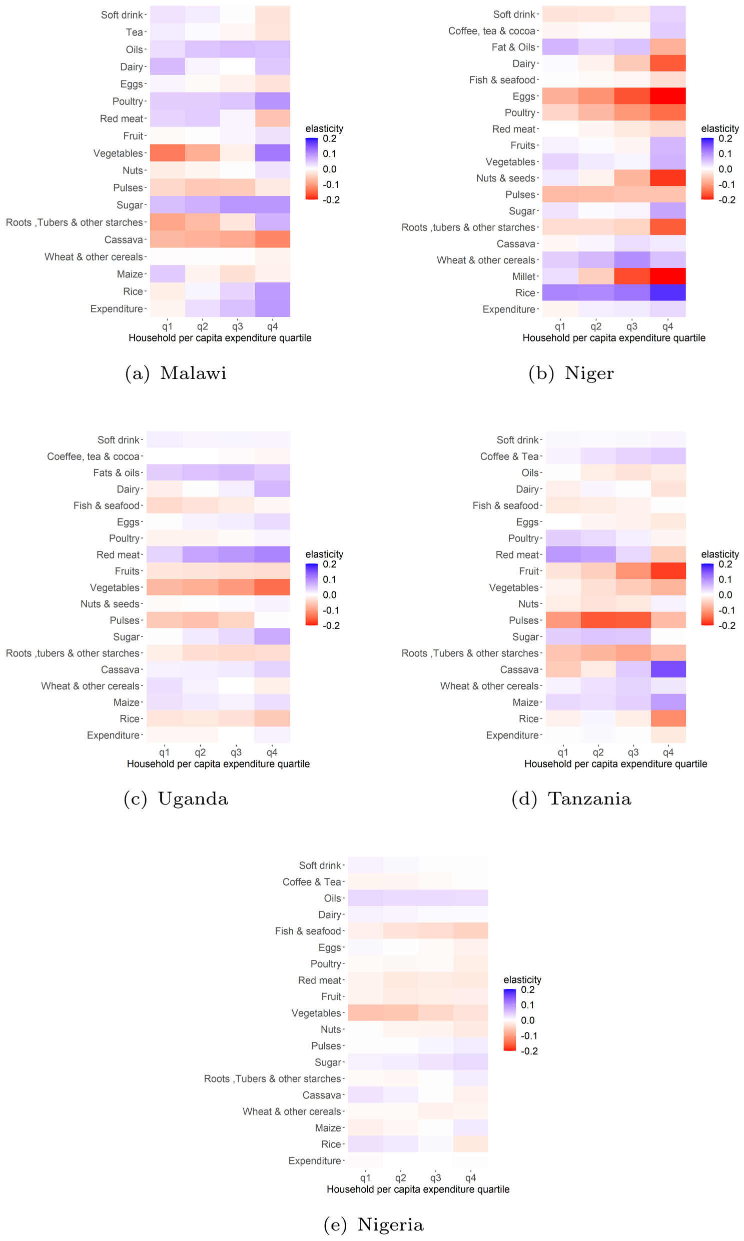

Suppl. Fig. 18: Elasticity of Nutrient-Rich Food Index (NRFI) with respect to each food group's price and with respect to total expenditures (bottom row of each matrix), reported by total expenditures quartile.

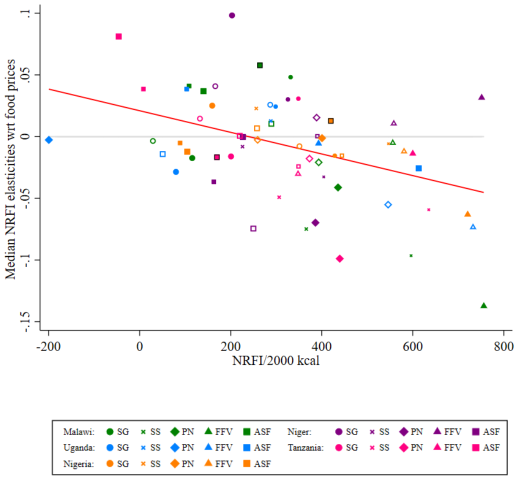

Suppl. Fig. 19: Scatter plot between the sample-wide median elasticity of overall dietary NRFI with respect to each food's price and each food's own NRFI score (per 2000 kCal). Each country is represented by one color. We categorize food groups into five categories: staple grains (SG), starchy staples (SS), pulses & nuts (PN), fruits & vegetables (FFV), and animal-sources food (ASF), with food groups from each category represented by one common symbol.

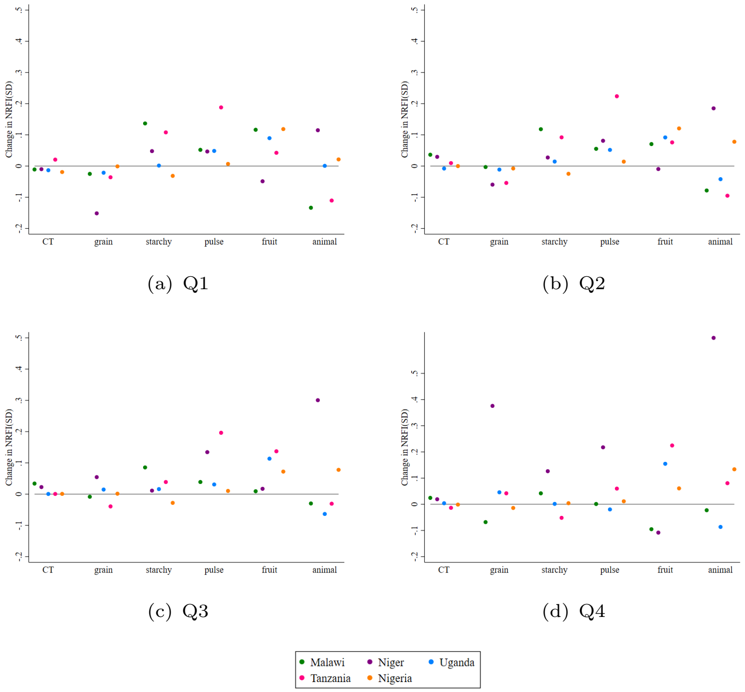

Suppl. Fig. 20: Nutrient-Rich Food Index (NRFI) response to the cash transfer simulation (labeled “CT") and to each of the five price discount (PD) simulations (each labeled by the targeted food category "grain" (staple grains), "starchy" (starchy staples), "pulse" (pulses & nuts), "fruit" (fruits & vegetables), and "animal" (animal-source foods)). The NRFI measure is standardized in each country by the sample-wide standard deviation. Panel 20(a) depicts the NRFI response for Q1 consumers relative to the NRFI level at baseline. The dot corresponding to each country depicts NRFI following the CT. Dots above the horizontal line indicate an improved NRFI score compared to baseline, while dots below the horizontal line represent a lower NRFI score compared to baseline and dots on the line indicate no change in NRFI compared to baseline. Response of Q2, Q3, and Q4 consumers to the same simulations are depicted in Panels 20(b), 20(c) and 20(d) respectively.

Supplementary Tables

Suppl. Table 1: Food demand elasticities with respect to food prices and total household expenditures for Q1 consumers (with per capita expenditures less than $1.90 per day) in Malawi

	Food Group																		Exp.
	Rice	Maize	Wheat	Cassava	Roots	Sugar	Pulses	Nuts	Vegetables	Fruit	RedMeat	Poultry	Eggs	Dairy	Oils	Tea	SoftDrink	OtherFood	
1. Rice	-1.38*	0.58	0.58**	-0.14	0.44	-0.12	-0.15	0.12	-0.24	-0.49*	0.24	0.90*	0.41*	0.15	0.30	-0.02	0.22	-0.02	0.42
	(0.56)	(0.37)	(0.22)	(0.34)	(0.25)	(0.19)	(0.24)	(0.14)	(0.29)	(0.23)	(0.43)	(0.46)	(0.20)	(0.33)	(0.16)	(0.07)	(0.24)	(0.04)	(0.46)
2. Maize	0.05	-0.45**	0.03	0.05	-0.05	-0.06**	-0.06	0.01	-0.06	0.01	-0.04	-0.04	-0.04	-0.13**	0.01	-0.00	-0.01	-0.02*	0.80**
	(0.05)	(0.08)	(0.03)	(0.04)	(0.03)	(0.02)	(0.04)	(0.02)	(0.05)	(0.03)	(0.05)	(0.07)	(0.03)	(0.05)	(0.02)	(0.01)	(0.03)	(0.01)	(0.07)
3. Wheat	0.53*	-0.32	-1.15**	-0.20	-0.61**	-0.10	-0.21	-0.24**	0.25	0.31*	0.69*	-0.16	-0.55**	0.06	-0.26	0.18**	0.27	-0.10**	2.64**
	(0.23)	(0.23)	(0.22)	(0.17)	(0.16)	(0.14)	(0.15)	(0.09)	(0.22)	(0.15)	(0.34)	(0.24)	(0.17)	(0.22)	(0.14)	(0.06)	(0.17)	(0.04)	(0.36)
4. Cassava	-0.10	1.07**	-0.11	-0.47	-0.15	-0.20	-0.24	-0.18*	-0.20	-0.13	-0.40	0.76**	0.09	1.45**	0.08	-0.01	0.11	0.03	-1.30**
	(0.22)	(0.25)	(0.10)	(0.31)	(0.14)	(0.10)	(0.16)	(0.08)	(0.23)	(0.12)	(0.26)	(0.26)	(0.12)	(0.25)	(0.08)	(0.04)	(0.17)	(0.03)	(0.34)
5. Roots	0.34	-0.82*	-0.50*	-0.26	-1.24**	0.01	-0.05	-0.13	-0.41	0.37*	-0.11	-0.34	-0.10	-0.08	0.01	-0.03	0.04	-0.04	2.26**
	(0.28)	(0.33)	(0.20)	(0.26)	(0.29)	(0.17)	(0.20)	(0.13)	(0.31)	(0.18)	(0.32)	(0.39)	(0.16)	(0.25)	(0.12)	(0.06)	(0.19)	(0.05)	(0.60)
6. Sugar	-0.13	-0.45**	-0.05	-0.33*	0.05	-0.63**	0.02	0.05	0.33*	-0.18	-0.11	-0.19	0.24*	0.86**	0.08	-0.15**	-0.14	0.12**	0.79**
	(0.17)	(0.15)	(0.11)	(0.14)	(0.13)	(0.17)	(0.12)	(0.06)	(0.17)	(0.11)	(0.23)	(0.14)	(0.10)	(0.16)	(0.08)	(0.05)	(0.11)	(0.04)	(0.17)
7. Pulses	-0.10	-0.36*	-0.06	-0.27	0.01	-0.00	-0.57**	-0.01	-0.07	0.03	0.19	-0.15	0.16	-0.01	0.03	-0.01	-0.05	-0.04	1.12**
	(0.15)	(0.18)	(0.08)	(0.15)	(0.11)	(0.08)	(0.18)	(0.08)	(0.16)	(0.10)	(0.21)	(0.23)	(0.10)	(0.13)	(0.07)	(0.03)	(0.12)	(0.03)	(0.25)
8. Nuts	0.21	-0.13	-0.47*	-0.51	-0.29	0.09	-0.08	-0.84**	-0.22	-0.12	0.04	-0.44	-0.09	0.32	-0.32*	-0.15*	-0.34	-0.07	1.73**
	(0.37)	(0.36)	(0.24)	(0.36)	(0.29)	(0.17)	(0.32)	(0.20)	(0.34)	(0.22)	(0.42)	(0.59)	(0.19)	(0.34)	(0.16)	(0.07)	(0.31)	(0.06)	(0.64)
9. Vegetables	-0.11	0.23*	0.19**	-0.20	-0.12	0.19**	0.04	-0.02	-0.41**	-0.20*	0.25	0.36**	0.02	0.55**	0.11*	0.04	0.19*	0.01	-0.54**
	(0.09)	(0.12)	(0.07)	(0.12)	(0.08)	(0.07)	(0.09)	(0.04)	(0.15)	(0.09)	(0.14)	(0.13)	(0.07)	(0.12)	(0.05)	(0.02)	(0.08)	(0.02)	(0.20)
10. Fruit	-0.58*	0.40	0.44**	-0.26	0.59**	-0.20	0.14	-0.06	-0.54	-0.85**	0.36	0.41	0.25	-0.19	-0.10	0.07	0.18	-0.02	-0.02
	(0.26)	(0.25)	(0.16)	(0.22)	(0.20)	(0.14)	(0.19)	(0.09)	(0.29)	(0.20)	(0.27)	(0.30)	(0.13)	(0.20)	(0.09)	(0.05)	(0.17)	(0.04)	(0.34)
11. RedMeat	0.12	-1.19**	0.42	-0.40	-0.15	-0.16	0.06	-0.01	0.08	0.13	-0.59	-0.45	-0.63**	-0.84**	0.12	0.28**	-0.03	-0.22**	3.98**
	(0.30)	(0.38)	(0.22)	(0.30)	(0.20)	(0.19)	(0.25)	(0.12)	(0.30)	(0.17)	(0.62)	(0.39)	(0.16)	(0.30)	(0.16)	(0.07)	(0.24)	(0.07)	(0.76)
12. Poultry	0.41	-2.21**	-0.14	0.38	-0.37	-0.27*	-0.52*	-0.16	-0.12	0.06	-0.42	-2.44**	-0.10	-0.38	-0.17	-0.01	-0.19	-0.09*	7.05**
	(0.29)	(0.50)	(0.14)	(0.26)	(0.21)	(0.13)	(0.24)	(0.15)	(0.31)	(0.18)	(0.34)	(0.83)	(0.18)	(0.29)	(0.10)	(0.04)	(0.20)	(0.04)	(1.32)
13. Eggs	0.55*	-0.63*	-0.78**	0.12	-0.16	0.33*	0.38	-0.06	-0.01	0.27	-1.34**	-0.16	0.05	0.58*	0.12	0.02	-0.37*	0.01	1.52**
	(0.28)	(0.29)	(0.22)	(0.25)	(0.19)	(0.16)	(0.23)	(0.10)	(0.29)	(0.16)	(0.33)	(0.39)	(0.30)	(0.23)	(0.15)	(0.06)	(0.15)	(0.04)	(0.33)
14. Dairy	0.24	-7.09**	-0.03	3.86**	-0.57	1.59**	-0.82	0.25	1.57	-0.58	-3.12*	-1.67	0.85	-3.94**	-1.14*	-0.15	-1.36	0.04	14.72**
	(1.08)	(1.68)	(0.67)	(1.28)	(0.72)	(0.59)	(0.74)	(0.44)	(1.14)	(0.66)	(1.38)	(1.50)	(0.55)	(1.47)	(0.47)	(0.17)	(0.83)	(0.15)	(3.41)
15. Oils	0.29	-0.06	-0.23	0.04	0.05	0.07	0.04	-0.16*	0.15	-0.11	0.32	-0.07	0.10	-0.38*	-0.55**	-0.07	0.07	-0.06	1.31**
	(0.18)	(0.16)	(0.15)	(0.14)	(0.12)	(0.11)	(0.12)	(0.06)	(0.17)	(0.09)	(0.27)	(0.17)	(0.12)	(0.15)	(0.14)	(0.04)	(0.12)	(0.03)	(0.20)
16. Tea	-0.15	-0.51	1.18**	-0.15	-0.22	-1.07**	-0.26	-0.51**	0.51	0.34	2.85**	-0.02	0.11	-0.39	-0.46*	-0.91**	-0.03	-0.02	2.36**
	(0.46)	(0.36)	(0.36)	(0.39)	(0.33)	(0.36)	(0.37)	(0.18)	(0.46)	(0.29)	(0.66)	(0.41)	(0.30)	(0.35)	(0.22)	(0.20)	(0.31)	(0.09)	(0.59)
17. SoftDrink	0.45	-1.46	0.57	0.23	0.00	-0.45	-0.46	-0.43	0.61	0.26	-0.12	-0.82	-0.63*	-1.36*	0.04	-0.02	-1.25	-0.04	5.07**
	(0.62)	(0.78)	(0.41)	(0.69)	(0.44)	(0.35)	(0.53)	(0.31)	(0.66)	(0.41)	(0.89)	(0.85)	(0.29)	(0.69)	(0.28)	(0.12)	(0.69)	(0.09)	(1.55)
18. OtherFood	-0.05	0.08	-0.12*	-0.01	-0.00	0.25**	-0.06	-0.04	0.03	-0.04	-0.44**	0.10	0.03	0.26**	-0.07	0.00	0.04	-0.41**	-0.16
	(0.08)	(0.08)	(0.06)	(0.08)	(0.07)	(0.08)	(0.07)	(0.03)	(0.10)	(0.06)	(0.14)	(0.07)	(0.06)	(0.07)	(0.05)	(0.02)	(0.05)	(0.07)	(0.13)

Note: This table shows the sample-wide median elasticity of food demand (quantity consumed) with respect to food prices (columns 1 thru 18) and total household expenditures (the last column). For a list of items in each food group, see Suppl. Table 46.

Suppl. Table 2: Food demand elasticities with respect to food prices and total household expenditures for Q2 consumers (with per capita expenditures between \$1.90 and \$3.20 per day) in Malawi

	Food Group																		Exp.
	Rice	Maize	Wheat	Cassava	Roots	Sugar	Pulses	Nuts	Vegetables	Fruit	RedMeat	Poultry	Eggs	Dairy	Oils	Tea	SoftDrink	OtherFood	
1. Rice	-1.07*	-0.14	0.04	-0.04	0.14	-0.13	-0.19	0.06	-0.09	-0.29	0.33	0.20	0.35	0.08	0.04	-0.00	0.10	-0.00	1.06**
	(0.42)	(0.26)	(0.15)	(0.25)	(0.18)	(0.15)	(0.20)	(0.10)	(0.22)	(0.16)	(0.32)	(0.37)	(0.20)	(0.21)	(0.11)	(0.05)	(0.18)	(0.03)	(0.38)
2. Maize	-0.02	-0.26**	0.04	0.02	-0.03	-0.05**	-0.03	0.02	-0.06	0.02	0.00	0.04	-0.04	-0.11**	0.02	0.01	0.01	-0.01	0.60**
	(0.04)	(0.07)	(0.02)	(0.04)	(0.03)	(0.02)	(0.03)	(0.02)	(0.04)	(0.02)	(0.05)	(0.06)	(0.02)	(0.03)	(0.02)	(0.01)	(0.03)	(0.00)	(0.08)
3. Wheat	0.02	-0.12	-0.56**	-0.19	-0.34**	-0.04	-0.25*	-0.15**	0.16	0.14	0.94**	-0.14	-0.40**	-0.12	-0.15	0.07*	0.32**	-0.12**	2.16**
	(0.14)	(0.13)	(0.15)	(0.12)	(0.11)	(0.09)	(0.10)	(0.06)	(0.14)	(0.08)	(0.21)	(0.17)	(0.12)	(0.13)	(0.09)	(0.03)	(0.10)	(0.03)	(0.22)
4. Cassava	-0.01	0.31	-0.14	-0.60	-0.27	-0.11	-0.27	-0.16	-0.21	-0.03	-0.41	0.34	0.00	0.92**	0.05	-0.03	-0.00	0.02	0.01
	(0.22)	(0.24)	(0.12)	(0.34)	(0.14)	(0.11)	(0.16)	(0.09)	(0.25)	(0.12)	(0.28)	(0.26)	(0.15)	(0.22)	(0.08)	(0.04)	(0.16)	(0.03)	(0.35)
5. Roots	0.12	-0.48**	-0.34**	-0.35*	-0.83**	-0.11	-0.00	0.04	-0.42*	0.24*	-0.24	-0.03	-0.12	-0.03	-0.02	-0.04	0.12	-0.04	1.95**
	(0.17)	(0.17)	(0.13)	(0.16)	(0.19)	(0.10)	(0.14)	(0.07)	(0.20)	(0.10)	(0.24)	(0.28)	(0.12)	(0.13)	(0.08)	(0.03)	(0.11)	(0.03)	(0.36)
6. Sugar	-0.12	-0.31**	0.01	-0.16	-0.06	-0.72**	0.01	0.04	0.11	-0.18*	0.06	-0.16	0.18	0.42**	0.09	-0.11**	-0.01	0.06	0.75**
	(0.14)	(0.10)	(0.10)	(0.11)	(0.10)	(0.13)	(0.09)	(0.04)	(0.14)	(0.07)	(0.18)	(0.11)	(0.10)	(0.11)	(0.07)	(0.03)	(0.07)	(0.03)	(0.15)
7. Pulses	-0.12	-0.26*	-0.13	-0.24*	0.02	-0.01	-0.45*	0.03	-0.07	-0.04	0.34	0.07	0.14	0.02	0.02	0.00	0.01	-0.01	1.30**
	(0.12)	(0.13)	(0.07)	(0.11)	(0.09)	(0.06)	(0.17)	(0.06)	(0.12)	(0.07)	(0.18)	(0.22)	(0.09)	(0.09)	(0.05)	(0.02)	(0.09)	(0.02)	(0.23)
8. Nuts	0.13	0.07	-0.33*	-0.43	0.08	0.05	0.06	-0.72**	-0.42	-0.21	0.31	0.03	-0.08	0.23	-0.27*	-0.11*	-0.12	-0.10*	1.80**
	(0.25)	(0.24)	(0.16)	(0.25)	(0.18)	(0.11)	(0.23)	(0.15)	(0.24)	(0.14)	(0.32)	(0.47)	(0.15)	(0.21)	(0.12)	(0.05)	(0.19)	(0.04)	(0.48)
9. Vegetables	-0.03	-0.03	0.18	-0.18	-0.19	0.10	0.02	-0.09	-0.11	-0.09	0.63**	0.07	0.06	0.34**	0.10	-0.00	0.12	-0.01	-0.08
	(0.12)	(0.14)	(0.10)	(0.16)	(0.12)	(0.09)	(0.11)	(0.06)	(0.24)	(0.09)	(0.22)	(0.16)	(0.11)	(0.13)	(0.07)	(0.03)	(0.09)	(0.02)	(0.27)
10. Fruit	-0.41*	0.18	0.26*	-0.07	0.38**	-0.24*	-0.05	-0.12	-0.22	-0.78**	0.42	0.18	0.24	-0.17	-0.15	-0.00	0.11	-0.01	0.53
	(0.21)	(0.19)	(0.13)	(0.18)	(0.15)	(0.11)	(0.15)	(0.08)	(0.21)	(0.16)	(0.22)	(0.24)	(0.13)	(0.14)	(0.08)	(0.04)	(0.12)	(0.03)	(0.29)
11. RedMeat	0.16	-0.51**	0.54**	-0.31	-0.18	-0.05	0.20	0.07	0.40*	0.12	-0.42	-0.06	-0.44**	-0.48**	0.18	0.17**	-0.03	-0.07*	2.97**
	(0.17)	(0.18)	(0.15)	(0.17)	(0.14)	(0.10)	(0.16)	(0.07)	(0.19)	(0.09)	(0.36)	(0.33)	(0.12)	(0.17)	(0.10)	(0.04)	(0.12)	(0.03)	(0.46)
12. Poultry	0.04	-0.75**	-0.13	0.11	-0.10	-0.21**	-0.14	-0.02	-0.20	-0.01	-0.12	-1.09*	-0.07	-0.10	-0.12	-0.00	-0.02	-0.03	4.35**
	(0.17)	(0.24)	(0.08)	(0.13)	(0.13)	(0.07)	(0.16)	(0.09)	(0.15)	(0.08)	(0.27)	(0.53)	(0.11)	(0.14)	(0.06)	(0.02)	(0.13)	(0.02)	(0.76)
13. Eggs	0.48*	-0.51*	-0.58**	-0.03	-0.18	0.23	0.27	-0.05	0.07	0.21	-1.03**	-0.11	-0.09	0.34*	0.10	0.01	-0.28*	0.00	1.71**
	(0.24)	(0.21)	(0.19)	(0.21)	(0.16)	(0.14)	(0.20)	(0.08)	(0.24)	(0.12)	(0.29)	(0.30)	(0.27)	(0.17)	(0.13)	(0.05)	(0.13)	(0.04)	(0.23)
14. Dairy	0.06	-2.60**	-0.36	1.88**	-0.21	0.62**	-0.24	0.15	0.67	-0.34	-1.79**	-0.51	0.41	-1.83**	-0.72**	-0.00	-0.44	0.08	6.54**
	(0.37)	(0.54)	(0.26)	(0.49)	(0.26)	(0.22)	(0.29)	(0.16)	(0.38)	(0.21)	(0.55)	(0.56)	(0.25)	(0.47)	(0.19)	(0.06)	(0.26)	(0.05)	(1.18)
15. Oils	0.04	0.01	-0.13	0.02	0.01	0.09	0.04	-0.12*	0.11	-0.13*	0.46*	-0.03	0.10	-0.31**	-0.60**	-0.01	0.14	-0.07**	1.17**
	(0.11)	(0.11)	(0.11)	(0.10)	(0.09)	(0.08)	(0.10)	(0.05)	(0.13)	(0.06)	(0.19)	(0.14)	(0.11)	(0.10)	(0.11)	(0.03)	(0.09)	(0.03)	(0.16)
16. Tea	-0.03	-0.02	0.42*	-0.25	-0.22	-0.65**	0.01	-0.29*	-0.11	-0.03	1.72**	0.06	0.02	0.05	-0.10	-0.70**	0.10	0.05	1.70**
	(0.27)	(0.21)	(0.20)	(0.24)	(0.20)	(0.21)	(0.23)	(0.11)	(0.29)	(0.16)	(0.39)	(0.25)	(0.24)	(0.19)	(0.14)	(0.12)	(0.18)	(0.05)	(0.34)
17. SoftDrink	0.17	-0.76*	0.64*	-0.07	0.17	-0.14	-0.16	-0.14	0.19	0.10	-0.18	-0.09	-0.46*	-0.46	0.17	0.03	-0.80*	-0.09*	4.15**
	(0.35)	(0.39)	(0.25)	(0.36)	(0.25)	(0.17)	(0.31)	(0.16)	(0.30)	(0.18)	(0.44)	(0.60)	(0.20)	(0.29)	(0.17)	(0.06)	(0.36)	(0.05)	(0.90)
18. OtherFood	0.02	-0.01	-0.32**	0.04	-0.06	0.23*	0.03	-0.12**	-0.04	-0.01	-0.24	0.09	0.04	0.26**	-0.18**	0.03	-0.08	-0.28**	-0.01
	(0.09)	(0.09)	(0.09)	(0.10)	(0.09)	(0.10)	(0.09)	(0.04)	(0.13)	(0.06)	(0.16)	(0.09)	(0.09)	(0.09)	(0.07)	(0.03)	(0.06)	(0.10)	(0.17)

Note: This table shows the sample-wide median elasticity of food demand (quantity consumed) with respect to food prices (columns 1 thru 18) and total household expenditures (the last column). For a list of items in each food group, see Suppl. Table 46.

Suppl. Table 3: Food demand elasticities with respect to food prices and total household expenditures for Q3 consumers (with per capita expenditures between \$3.20 and \$5.50 per day) in Malawi

	Food Group																		Exp.
	Rice	Maize	Wheat	Cassava	Roots	Sugar	Pulses	Nuts	Vegetables	Fruit	RedMeat	Poultry	Eggs	Dairy	Oils	Tea	SoftDrink	OtherFood	
1. Rice	-0.89*	-0.46	-0.27	0.03	-0.02	-0.13	-0.20	0.04	0.03	-0.18	0.38	-0.21	0.32	0.03	-0.11	0.01	0.04	0.02	1.19**
	(0.40)	(0.25)	(0.17)	(0.24)	(0.19)	(0.15)	(0.22)	(0.10)	(0.22)	(0.14)	(0.34)	(0.36)	(0.20)	(0.18)	(0.11)	(0.04)	(0.19)	(0.03)	(0.37)
2. Maize	-0.10*	-0.06	0.06	-0.01	-0.01	-0.05*	0.00	0.05*	-0.07	0.02	0.06	0.16	-0.04	-0.08*	0.03	0.01*	0.02	-0.00	0.43**
	(0.05)	(0.10)	(0.03)	(0.05)	(0.04)	(0.02)	(0.04)	(0.02)	(0.05)	(0.03)	(0.06)	(0.09)	(0.03)	(0.03)	(0.02)	(0.01)	(0.03)	(0.01)	(0.11)
3. Wheat	-0.28*	-0.01	-0.23	-0.18	-0.19	0.00	-0.26**	-0.09	0.11	0.05	1.06**	-0.13	-0.31**	-0.23*	-0.08	0.01	0.34**	-0.13**	1.81**
	(0.13)	(0.11)	(0.14)	(0.11)	(0.11)	(0.08)	(0.10)	(0.05)	(0.12)	(0.06)	(0.19)	(0.15)	(0.10)	(0.10)	(0.08)	(0.03)	(0.09)	(0.03)	(0.17)
4. Cassava	0.04	-0.03	-0.18	-0.68	-0.38*	-0.06	-0.28	-0.15	-0.18	0.05	-0.44	0.04	-0.07	0.56**	0.02	-0.05	-0.08	0.02	0.61
	(0.25)	(0.26)	(0.15)	(0.37)	(0.16)	(0.12)	(0.20)	(0.10)	(0.28)	(0.12)	(0.33)	(0.30)	(0.18)	(0.21)	(0.10)	(0.04)	(0.17)	(0.04)	(0.37)
5. Roots	-0.04	-0.27	-0.23	-0.43**	-0.52*	-0.20*	0.04	0.16*	-0.44*	0.14	-0.34	0.21	-0.14	0.01	-0.04	-0.05	0.18	-0.04	1.78**
	(0.16)	(0.15)	(0.13)	(0.16)	(0.20)	(0.10)	(0.14)	(0.08)	(0.19)	(0.09)	(0.27)	(0.30)	(0.11)	(0.11)	(0.08)	(0.03)	(0.11)	(0.03)	(0.32)
6. Sugar	-0.13	-0.28**	0.05	-0.08	-0.16	-0.75**	0.00	0.03	-0.03	-0.19**	0.19	-0.16	0.17	0.18	0.11	-0.09*	0.08	0.03	0.74**
	(0.15)	(0.10)	(0.11)	(0.11)	(0.11)	(0.14)	(0.09)	(0.05)	(0.15)	(0.07)	(0.20)	(0.11)	(0.11)	(0.10)	(0.08)	(0.03)	(0.08)	(0.03)	(0.16)
7. Pulses	-0.16	-0.16	-0.20*	-0.23	0.04	-0.02	-0.30	0.06	-0.06	-0.11	0.50*	0.27	0.13	0.05	0.02	0.02	0.05	0.02	1.46**
	(0.13)	(0.13)	(0.09)	(0.12)	(0.10)	(0.05)	(0.22)	(0.06)	(0.12)	(0.07)	(0.21)	(0.25)	(0.10)	(0.09)	(0.06)	(0.03)	(0.10)	(0.02)	(0.25)
8. Nuts	0.08	0.23	-0.26	-0.39	0.38	0.04	0.17	-0.60**	-0.60*	-0.30*	0.53	0.39	-0.08	0.16	-0.25*	-0.09*	0.04	-0.13**	1.92**
	(0.21)	(0.22)	(0.15)	(0.23)	(0.20)	(0.10)	(0.22)	(0.15)	(0.25)	(0.12)	(0.33)	(0.48)	(0.13)	(0.17)	(0.12)	(0.04)	(0.15)	(0.04)	(0.47)
9. Vegetables	0.05	-0.20	0.15	-0.15	-0.27	-0.01	-0.01	-0.17*	0.19	0.04	1.01**	-0.26	0.11	0.10	0.09	-0.06	0.05	-0.02	0.30
	(0.16)	(0.17)	(0.13)	(0.20)	(0.15)	(0.11)	(0.15)	(0.08)	(0.34)	(0.10)	(0.32)	(0.21)	(0.13)	(0.12)	(0.09)	(0.04)	(0.11)	(0.03)	(0.29)
10. Fruit	-0.28	0.08	0.11	0.08	0.23	-0.27**	-0.19	-0.17*	0.06	-0.72**	0.45*	-0.01	0.22	-0.16	-0.19*	-0.06	0.05	0.01	0.88**
	(0.19)	(0.17)	(0.12)	(0.16)	(0.14)	(0.10)	(0.15)	(0.07)	(0.18)	(0.13)	(0.23)	(0.23)	(0.11)	(0.12)	(0.08)	(0.03)	(0.11)	(0.03)	(0.29)
11. RedMeat	0.18	-0.23	0.61**	-0.27	-0.20	0.03	0.29	0.11	0.58**	0.13	-0.31	0.14	-0.36**	-0.32**	0.21**	0.12**	-0.04	-0.01	2.53**
	(0.15)	(0.13)	(0.14)	(0.14)	(0.14)	(0.08)	(0.16)	(0.07)	(0.17)	(0.07)	(0.30)	(0.33)	(0.11)	(0.11)	(0.08)	(0.03)	(0.10)	(0.02)	(0.37)
12. Poultry	-0.15	-0.20	-0.12	-0.03	0.04	-0.16**	0.06	0.06	-0.30*	-0.05	0.05	-0.40	-0.06	0.03	-0.08	-0.00	0.07	-0.02	3.35**
	(0.13)	(0.17)	(0.07)	(0.10)	(0.12)	(0.05)	(0.15)	(0.08)	(0.12)	(0.06)	(0.27)	(0.48)	(0.08)	(0.09)	(0.05)	(0.01)	(0.12)	(0.01)	(0.56)
13. Eggs	0.45*	-0.39*	-0.47**	-0.12	-0.19	0.17	0.22	-0.04	0.12	0.18	-0.87**	-0.08	-0.16	0.20	0.10	-0.01	-0.23*	0.01	1.72**
	(0.22)	(0.17)	(0.16)	(0.19)	(0.14)	(0.12)	(0.18)	(0.07)	(0.20)	(0.09)	(0.27)	(0.26)	(0.24)	(0.14)	(0.12)	(0.05)	(0.12)	(0.04)	(0.24)
14. Dairy	0.00	-0.95**	-0.48**	0.89**	-0.04	0.18	0.01	0.09	0.09	-0.22	-1.03**	0.09	0.21	-0.84**	-0.46**	0.08*	0.01	0.05	3.20**
	(0.23)	(0.25)	(0.17)	(0.27)	(0.17)	(0.13)	(0.20)	(0.10)	(0.20)	(0.11)	(0.34)	(0.35)	(0.16)	(0.23)	(0.12)	(0.04)	(0.13)	(0.04)	(0.53)
15. Oils	-0.13	0.05	-0.07	0.00	-0.02	0.11	0.05	-0.10*	0.09	-0.15*	0.58**	-0.01	0.11	-0.27**	-0.60**	0.02	0.20*	-0.09**	1.11**
	(0.11)	(0.10)	(0.11)	(0.09)	(0.10)	(0.08)	(0.11)	(0.05)	(0.12)	(0.06)	(0.17)	(0.15)	(0.10)	(0.09)	(0.12)	(0.03)	(0.09)	(0.03)	(0.16)
16. Tea	0.04	0.20	0.06	-0.32	-0.23	-0.47**	0.16	-0.19*	-0.45	-0.23	1.27**	0.11	-0.02	0.30	0.09	-0.57**	0.18	0.08	1.37**
	(0.24)	(0.19)	(0.17)	(0.20)	(0.18)	(0.18)	(0.22)	(0.09)	(0.26)	(0.13)	(0.33)	(0.21)	(0.22)	(0.16)	(0.14)	(0.10)	(0.16)	(0.05)	(0.27)
17. SoftDrink	0.02	-0.32	0.63**	-0.21	0.25	0.04	0.02	0.01	-0.02	0.01	-0.20	0.27	-0.35*	0.00	0.24	0.05	-0.60*	-0.12**	3.44**
	(0.29)	(0.27)	(0.22)	(0.25)	(0.20)	(0.12)	(0.26)	(0.11)	(0.22)	(0.12)	(0.34)	(0.51)	(0.16)	(0.16)	(0.15)	(0.05)	(0.28)	(0.04)	(0.67)
18. OtherFood	0.11	-0.01	-0.57**	0.10	-0.14	0.12	0.16	-0.22**	-0.09	0.04	0.09	0.05	0.05	0.21	-0.31**	0.07	-0.23**	-0.22	0.32
	(0.14)	(0.11)	(0.15)	(0.13)	(0.12)	(0.13)	(0.12)	(0.05)	(0.18)	(0.07)	(0.21)	(0.13)	(0.13)	(0.11)	(0.10)	(0.04)	(0.09)	(0.13)	(0.20)

Note: This table shows the sample-wide median elasticity of food demand (quantity consumed) with respect to food prices (columns 1 thru 18) and total household expenditures (the last column). For a list of items in each food group, see Suppl. Table 46.

Suppl. Table 4: Food demand elasticities with respect to food prices and total household expenditures for Q4 consumers (with per capita expenditures greater than $5.50 per day) in Malawi

	Food Group																		Exp.
	Rice	Maize	Wheat	Cassava	Roots	Sugar	Pulses	Nuts	Vegetables	Fruit	RedMeat	Poultry	Eggs	Dairy	Oils	Tea	SoftDrink	OtherFood	
1. Rice	-0.67	-0.81*	-0.63*	0.11	-0.19	-0.12	-0.20	0.01	0.17	-0.07	0.49	-0.65	0.32	-0.01	-0.27	0.02	-0.02	0.04	0.97*
	(0.58)	(0.36)	(0.28)	(0.35)	(0.28)	(0.21)	(0.33)	(0.13)	(0.32)	(0.19)	(0.54)	(0.46)	(0.23)	(0.26)	(0.18)	(0.07)	(0.28)	(0.05)	(0.47)
2. Maize	-0.32**	0.34	0.08	-0.12	0.03	-0.06	0.08	0.10*	-0.12	0.02	0.18	0.42*	-0.04	0.01	0.05	0.04*	0.07	0.01	0.30
	(0.11)	(0.18)	(0.06)	(0.10)	(0.08)	(0.04)	(0.10)	(0.04)	(0.09)	(0.05)	(0.13)	(0.19)	(0.05)	(0.07)	(0.04)	(0.02)	(0.07)	(0.01)	(0.18)
3. Wheat	-0.59**	0.07	0.19	-0.19	-0.04	0.06	-0.30*	-0.03	0.05	-0.05	1.29**	-0.11	-0.23*	-0.36**	-0.01	-0.05	0.39**	-0.15**	1.49**
	(0.17)	(0.13)	(0.19)	(0.14)	(0.13)	(0.10)	(0.13)	(0.06)	(0.14)	(0.08)	(0.25)	(0.18)	(0.09)	(0.11)	(0.09)	(0.03)	(0.11)	(0.04)	(0.15)
4. Cassava	0.13	-0.39	-0.24	-0.78	-0.52*	0.03	-0.24	-0.13	-0.09	0.19	-0.50	-0.42	-0.18	0.08	-0.01	-0.08	-0.22	0.04	1.00*
	(0.39)	(0.36)	(0.24)	(0.52)	(0.26)	(0.16)	(0.32)	(0.15)	(0.39)	(0.18)	(0.52)	(0.48)	(0.23)	(0.29)	(0.14)	(0.06)	(0.26)	(0.05)	(0.44)
5. Roots	-0.26	-0.04	-0.07	-0.54**	-0.05	-0.32*	0.09	0.35**	-0.51*	0.01	-0.48	0.57	-0.17	0.06	-0.07	-0.06	0.27	-0.06	1.67**
	(0.21)	(0.20)	(0.15)	(0.19)	(0.30)	(0.13)	(0.18)	(0.11)	(0.22)	(0.11)	(0.36)	(0.39)	(0.11)	(0.14)	(0.11)	(0.04)	(0.16)	(0.04)	(0.28)
6. Sugar	-0.16	-0.25	0.13	0.05	-0.33	-0.77**	-0.00	0.03	-0.26	-0.25*	0.41	-0.18	0.16	-0.17	0.16	-0.07	0.24	-0.02	0.63**
	(0.23)	(0.14)	(0.18)	(0.15)	(0.17)	(0.22)	(0.16)	(0.08)	(0.22)	(0.10)	(0.32)	(0.18)	(0.13)	(0.14)	(0.13)	(0.05)	(0.14)	(0.06)	(0.19)
7. Pulses	-0.22	0.03	-0.32**	-0.21	0.07	-0.03	-0.05	0.12	-0.05	-0.21*	0.75*	0.59	0.12	0.08	0.02	0.05	0.13	0.06*	1.75**
	(0.17)	(0.14)	(0.12)	(0.15)	(0.12)	(0.07)	(0.29)	(0.08)	(0.14)	(0.09)	(0.29)	(0.32)	(0.10)	(0.11)	(0.08)	(0.03)	(0.13)	(0.03)	(0.28)
8. Nuts	-0.01	0.50	-0.15	-0.33	0.81**	0.02	0.35	-0.43*	-0.88**	-0.42**	0.87*	0.93	-0.08	0.07	-0.21	-0.05	0.27	-0.19**	2.19**
	(0.24)	(0.25)	(0.16)	(0.24)	(0.29)	(0.13)	(0.27)	(0.19)	(0.31)	(0.15)	(0.42)	(0.55)	(0.13)	(0.18)	(0.13)	(0.04)	(0.19)	(0.06)	(0.45)
9. Vegetables	0.20	-0.35	0.09	-0.07	-0.41	-0.20	-0.02	-0.30*	0.82	0.27	1.72**	-0.88*	0.18	-0.33	0.05	-0.16*	-0.08	-0.03	0.76**
	(0.26)	(0.24)	(0.18)	(0.28)	(0.22)	(0.17)	(0.24)	(0.12)	(0.50)	(0.14)	(0.52)	(0.37)	(0.16)	(0.18)	(0.13)	(0.06)	(0.18)	(0.05)	(0.29)
10. Fruit	-0.13	0.03	-0.08	0.28	0.02	-0.32*	-0.37	-0.24*	0.43	-0.63**	0.53	-0.26	0.21	-0.16	-0.25*	-0.14**	-0.02	0.04	1.20**
	(0.26)	(0.24)	(0.16)	(0.22)	(0.20)	(0.13)	(0.23)	(0.10)	(0.22)	(0.17)	(0.32)	(0.33)	(0.11)	(0.18)	(0.11)	(0.05)	(0.18)	(0.03)	(0.31)
11. RedMeat	0.23	0.04	0.75**	-0.25	-0.23	0.11	0.41*	0.17*	0.81**	0.15	-0.12	0.38	-0.29**	-0.17	0.29**	0.07*	-0.05	0.06*	2.14**
	(0.17)	(0.13)	(0.18)	(0.15)	(0.15)	(0.09)	(0.18)	(0.07)	(0.20)	(0.08)	(0.35)	(0.34)	(0.09)	(0.10)	(0.09)	(0.03)	(0.11)	(0.03)	(0.30)
12. Poultry	-0.36**	0.25	-0.11	-0.18	0.19	-0.11*	0.24	0.15	-0.48**	-0.10	0.28	0.50	-0.04	0.17	-0.03	0.01	0.17	-0.02	2.60**
	(0.13)	(0.16)	(0.07)	(0.11)	(0.13)	(0.05)	(0.16)	(0.08)	(0.14)	(0.06)	(0.28)	(0.54)	(0.07)	(0.10)	(0.05)	(0.01)	(0.12)	(0.01)	(0.42)
13. Eggs	0.45*	-0.25	-0.37**	-0.23	-0.20	0.13	0.18	-0.03	0.20	0.17*	-0.72**	-0.04	-0.17	0.05	0.12	-0.02	-0.19	0.01	1.58**
	(0.20)	(0.15)	(0.14)	(0.19)	(0.13)	(0.10)	(0.17)	(0.07)	(0.18)	(0.08)	(0.25)	(0.25)	(0.21)	(0.12)	(0.10)	(0.04)	(0.12)	(0.03)	(0.23)
14. Dairy	-0.04	-0.04	-0.58**	0.09	0.07	-0.20	0.14	0.04	-0.49*	-0.14	-0.40	0.63	0.06	-0.03	-0.28*	0.15**	0.37*	0.01	1.39**
	(0.26)	(0.22)	(0.19)	(0.25)	(0.18)	(0.13)	(0.22)	(0.10)	(0.22)	(0.12)	(0.31)	(0.37)	(0.12)	(0.28)	(0.12)	(0.05)	(0.16)	(0.04)	(0.36)
15. Oils	-0.38*	0.10	0.01	-0.01	-0.06	0.15	0.06	-0.09	0.06	-0.20**	0.83**	0.03	0.13	-0.26*	-0.54**	0.07	0.31*	-0.13**	1.09**
	(0.18)	(0.12)	(0.14)	(0.12)	(0.14)	(0.11)	(0.15)	(0.07)	(0.16)	(0.08)	(0.23)	(0.19)	(0.11)	(0.11)	(0.16)	(0.04)	(0.13)	(0.04)	(0.19)
16. Tea	0.13	0.45	-0.35	-0.45	-0.28	-0.32	0.35	-0.10	-0.94**	-0.51**	0.88*	0.20	-0.07	0.63**	0.32	-0.33*	0.31	0.13*	1.15**
	(0.34)	(0.25)	(0.25)	(0.26)	(0.26)	(0.24)	(0.32)	(0.11)	(0.35)	(0.17)	(0.44)	(0.26)	(0.22)	(0.23)	(0.20)	(0.13)	(0.24)	(0.07)	(0.27)
17. SoftDrink	-0.09	0.00	0.63**	-0.33	0.32	0.21	0.17	0.15	-0.23	-0.06	-0.20	0.62	-0.25	0.39*	0.31*	0.07	-0.42	-0.16**	2.76**
	(0.28)	(0.22)	(0.21)	(0.24)	(0.21)	(0.13)	(0.25)	(0.10)	(0.22)	(0.12)	(0.34)	(0.45)	(0.13)	(0.17)	(0.14)	(0.05)	(0.29)	(0.05)	(0.45)
18. OtherFood	0.25	0.15	-0.96**	0.20	-0.25	-0.10	0.43*	-0.36**	-0.14	0.14	0.75*	-0.07	0.05	0.05	-0.52**	0.12*	-0.51**	-0.31	0.88**
	(0.23)	(0.16)	(0.25)	(0.19)	(0.19)	(0.22)	(0.21)	(0.09)	(0.27)	(0.11)	(0.35)	(0.21)	(0.15)	(0.17)	(0.17)	(0.06)	(0.16)	(0.19)	(0.21)

Note: This table shows the sample-wide median elasticity of food demand (quantity consumed) with respect to food prices (columns 1 thru 18) and total household expenditures (the last column). For a list of items in each food group, see Suppl. Table 46.

Suppl. Table 5: Food demand elasticities with respect to food prices and total household expenditures for Q1 consumers (with per capita expenditures less than $1.90 per day) in Niger

	Food Group																			Exp.
	Rice	Millet	Wheat	Cassava	Roots	Sugar	Pulses	Nuts	Vegetables	Fruit	RedMeat	Poultry	Eggs	Fish	Dairy	Oils	Coffee	SoftDrink	OtherFood	
1. Rice	0.50	-1.14	-0.29	0.20	0.17	-0.02	0.22	-0.09	0.38	0.04	0.10	-0.68	-0.08	0.18	0.17	0.48	0.17	-0.20	0.25	2.55**
	(1.37)	(1.17)	(0.18)	(0.16)	(0.18)	(0.25)	(0.34)	(0.19)	(0.25)	(0.13)	(0.17)	(0.46)	(0.23)	(0.12)	(0.23)	(0.34)	(0.11)	(0.21)	(0.23)	(0.88)
2. Millet	-0.16	-1.10**	-0.07	0.02	0.16*	-0.02	0.02	-0.03	-0.03	-0.03	-0.11	0.13	0.01	0.00	-0.22*	0.03	-0.06	0.01	-0.20*	0.70*
	(0.26)	(0.34)	(0.07)	(0.04)	(0.07)	(0.09)	(0.12)	(0.06)	(0.07)	(0.04)	(0.07)	(0.20)	(0.08)	(0.04)	(0.10)	(0.09)	(0.04)	(0.07)	(0.09)	(0.34)
3. Wheat	-0.16	-0.49**	-0.77**	0.22**	-0.04	-0.14	-0.17*	0.14**	0.05	0.10**	0.06	-0.19	0.09	0.08**	0.21**	-0.10*	0.06**	0.13*	-0.10**	1.76**
	(0.09)	(0.18)	(0.06)	(0.04)	(0.04)	(0.09)	(0.07)	(0.05)	(0.04)	(0.03)	(0.05)	(0.15)	(0.05)	(0.03)	(0.07)	(0.04)	(0.02)	(0.05)	(0.04)	(0.28)
4. Cassava	0.51	0.11	0.73**	-1.06**	-0.15	-0.08	-0.31*	0.11	0.25*	0.01	-0.29	0.25	-0.61**	-0.15	-0.35*	0.19	-0.08	0.11	0.01	1.21
	(0.34)	(0.39)	(0.15)	(0.10)	(0.13)	(0.16)	(0.14)	(0.17)	(0.11)	(0.07)	(0.15)	(0.43)	(0.18)	(0.09)	(0.17)	(0.11)	(0.07)	(0.14)	(0.09)	(0.68)
5. Roots	0.53	1.41	-0.38	-0.24	-1.16*	0.01	-0.09	0.61	-0.01	0.64*	-0.27	0.21	-0.55	-0.56	-0.77	-0.30	-0.00	-0.34	-0.50	3.39
	(0.66)	(1.32)	(0.34)	(0.25)	(0.54)	(0.68)	(0.39)	(0.51)	(0.32)	(0.29)	(0.58)	(1.54)	(0.56)	(0.33)	(0.72)	(0.26)	(0.17)	(0.47)	(0.31)	(2.82)
6. Sugar	-0.05	-0.73	-0.54	-0.09	0.03	-0.72	0.15	-0.75	-0.31	0.07	0.75	0.69	0.91	0.36	0.42	-0.16	0.35	0.02	-0.17	2.70
	(0.85)	(2.62)	(1.13)	(0.32)	(0.80)	(1.40)	(0.65)	(1.23)	(0.77)	(0.24)	(1.14)	(3.08)	(1.42)	(0.57)	(1.32)	(0.36)	(0.42)	(0.63)	(0.48)	(5.16)
7. Pulses	0.41	-0.15	-0.41*	-0.21	-0.02	0.11	-1.38**	-0.21	0.11	0.01	0.05	0.35	-0.84**	0.21	-0.04	0.05	-0.01	-0.25	-0.06	1.75*
	(0.55)	(0.90)	(0.20)	(0.12)	(0.18)	(0.25)	(0.50)	(0.16)	(0.20)	(0.13)	(0.18)	(0.46)	(0.32)	(0.13)	(0.20)	(0.24)	(0.10)	(0.20)	(0.20)	(0.84)
8. Nuts	-0.19	-0.35	0.43	0.10	0.38	-0.65	-0.28	-0.54	0.10	0.02	-0.53	-0.48	-0.66	0.19	-0.46	-0.14	-0.40	0.29	0.02	1.37
	(0.43)	(0.92)	(0.34)	(0.20)	(0.37)	(0.65)	(0.29)	(0.63)	(0.25)	(0.16)	(0.66)	(1.53)	(0.52)	(0.23)	(0.50)	(0.20)	(0.25)	(0.44)	(0.15)	(2.47)
9. Vegetables	0.77	-0.32	0.18	0.18	0.03	-0.17	0.14	0.08	-0.66**	-0.01	0.09	-0.31	-0.22	-0.07	0.19	-0.36	-0.00	-0.02	0.52*	1.08
	(0.49)	(0.62)	(0.14)	(0.11)	(0.15)	(0.25)	(0.22)	(0.17)	(0.24)	(0.08)	(0.16)	(0.48)	(0.21)	(0.10)	(0.22)	(0.22)	(0.07)	(0.14)	(0.23)	(0.85)
10. Fruit	0.11	-0.69	0.42**	0.01	0.64**	0.09	-0.01	0.02	-0.04	-0.89**	-0.03	-0.78	0.05	0.23	-0.49*	-0.46*	-0.14	0.18	0.15	1.98*
	(0.41)	(0.61)	(0.16)	(0.11)	(0.21)	(0.21)	(0.26)	(0.21)	(0.15)	(0.16)	(0.22)	(0.52)	(0.21)	(0.12)	(0.21)	(0.22)	(0.09)	(0.21)	(0.13)	(0.93)
11. RedMeat	-0.01	-1.71*	-0.22	-0.17*	-0.09	0.29	-0.05	-0.31	-0.05	-0.02	-0.58	0.81	0.12	0.16	-0.22	-0.06	-0.02	0.36	-0.17	5.16**
	(0.23)	(0.80)	(0.19)	(0.08)	(0.18)	(0.40)	(0.17)	(0.32)	(0.14)	(0.08)	(0.39)	(0.65)	(0.19)	(0.11)	(0.36)	(0.11)	(0.08)	(0.21)	(0.12)	(1.86)
12. Poultry	-0.85	-1.26	-0.70	0.06	0.05	0.18	0.08	-0.24	-0.35	-0.22	0.63	-0.04	0.59	0.23	0.00	-0.09	0.06	-0.58	-0.30	6.95
	(0.62)	(2.07)	(0.72)	(0.23)	(0.51)	(0.85)	(0.33)	(0.76)	(0.49)	(0.19)	(0.63)	(2.10)	(0.66)	(0.29)	(0.69)	(0.19)	(0.12)	(0.43)	(0.33)	(4.47)
13. Eggs	-0.61	-1.56	0.01	-1.08*	-0.63	1.39	-2.21*	-1.26	-0.75	0.03	0.41	2.52	-0.93	0.08	-1.10	-0.47	0.33	0.11	-0.09	7.38
	(1.05)	(2.47)	(0.61)	(0.50)	(0.75)	(1.19)	(0.94)	(0.91)	(0.66)	(0.29)	(0.75)	(2.53)	(0.80)	(0.38)	(0.90)	(0.36)	(0.26)	(0.56)	(0.42)	(4.76)
14. Fish	0.38	-0.71	0.08	-0.17	-0.40	0.34	0.27	0.22	-0.18	0.16	0.35	0.65	0.06	-1.29**	0.01	-0.24	-0.00	0.07	-0.02	3.50**
	(0.38)	(0.81)	(0.19)	(0.13)	(0.25)	(0.34)	(0.25)	(0.30)	(0.19)	(0.12)	(0.31)	(0.77)	(0.28)	(0.26)	(0.31)	(0.14)	(0.11)	(0.32)	(0.12)	(1.24)
15. Dairy	0.20	-1.41	0.25	-0.17	-0.23	0.20	-0.05	-0.26	0.08	-0.16	-0.12	0.16	-0.28	0.03	-0.97**	0.08	0.16*	0.01	0.04	2.39*
	(0.22)	(0.79)	(0.15)	(0.12)	(0.23)	(0.33)	(0.13)	(0.21)	(0.13)	(0.09)	(0.24)	(0.64)	(0.22)	(0.09)	(0.27)	(0.08)	(0.08)	(0.16)	(0.09)	(1.06)
16. Oils	0.91	0.01	-0.19	0.14	-0.11	-0.08	0.07	-0.10	-0.35*	-0.21*	0.07	0.14	-0.12	-0.11	0.17	-0.94**	0.09	0.06	0.56**	1.34*
	(0.53)	(0.63)	(0.11)	(0.08)	(0.09)	(0.12)	(0.23)	(0.10)	(0.15)	(0.10)	(0.11)	(0.22)	(0.11)	(0.07)	(0.11)	(0.21)	(0.06)	(0.09)	(0.15)	(0.53)
17. Coffee	0.53	-1.41*	0.14	-0.13	0.00	0.48*	-0.07	-0.67**	-0.05	-0.15	-0.05	0.28	0.32	-0.00	0.46*	0.12	-0.64**	0.20	-0.18	3.02**
	(0.37)	(0.65)	(0.14)	(0.12)	(0.16)	(0.23)	(0.22)	(0.24)	(0.15)	(0.10)	(0.24)	(0.38)	(0.20)	(0.13)	(0.23)	(0.15)	(0.13)	(0.22)	(0.14)	(0.76)
18. SoftDrink	-0.83	-1.36	0.15	0.12	-0.32	-0.05	-0.62	0.41	-0.19	0.15	0.99	-2.09	0.09	0.09	-0.08	-0.03	0.17	0.19	-0.48	6.14
	(0.87)	(1.91)	(0.42)	(0.26)	(0.45)	(0.65)	(0.49)	(0.63)	(0.36)	(0.25)	(0.64)	(1.36)	(0.49)	(0.39)	(0.59)	(0.25)	(0.25)	(1.01)	(0.37)	(3.68)
19. OtherFood	0.52	-0.94**	-0.11	0.01	-0.14*	-0.04	-0.02	0.03	0.46**	0.08	0.04	-0.02	0.09	0.05	0.13	0.48**	-0.03	-0.10	-1.25**	0.55
	(0.27)	(0.33)	(0.07)	(0.05)	(0.07)	(0.09)	(0.15)	(0.06)	(0.10)	(0.05)	(0.06)	(0.13)	(0.08)	(0.04)	(0.09)	(0.11)	(0.04)	(0.08)	(0.09)	(0.28)

Note: This table shows the sample-wide median elasticity of food demand (quantity consumed) with respect to food prices (columns 1 thru 19) and total household expenditures (the last column). For a list of items in each food group, see Suppl. Table 47.

Suppl. Table 6: Food demand elasticities with respect to food prices and total household expenditures for Q2 consumers (with per capita expenditures between $1.90 and $3.20 per day) in Niger

	Food Group																			Exp.
	Rice	Millet	Wheat	Cassava	Roots	Sugar	Pulses	Nuts	Vegetables	Fruit	RedMeat	Poultry	Eggs	Fish	Dairy	Oils	Coffee	SoftDrink	OtherFood	
1. Rice	-0.12	-1.76	-0.43**	0.25	0.07	-0.03	-0.03	-0.03	0.05	0.09	0.11	-0.60*	-0.21	0.11	0.01	0.63*	0.08	-0.17	0.59**	2.02**
	(1.41)	(0.91)	(0.14)	(0.14)	(0.11)	(0.16)	(0.20)	(0.11)	(0.18)	(0.07)	(0.14)	(0.30)	(0.15)	(0.07)	(0.14)	(0.31)	(0.07)	(0.13)	(0.21)	(0.53)
2. Millet	-0.49	-1.15**	-0.07	0.05	0.11	0.05	0.02	-0.02	-0.23*	0.05	-0.08	0.23	0.12	0.00	-0.17	0.00	-0.03	-0.01	-0.13	0.38
	(0.34)	(0.38)	(0.07)	(0.04)	(0.07)	(0.11)	(0.10)	(0.07)	(0.10)	(0.04)	(0.08)	(0.21)	(0.09)	(0.04)	(0.09)	(0.13)	(0.03)	(0.06)	(0.07)	(0.35)
3. Wheat	-0.30**	-0.41**	-0.83**	0.14**	-0.06*	-0.06	-0.11*	0.11**	0.04	0.09**	0.06	-0.20	0.04	0.02	0.05	-0.09**	0.05**	0.07	-0.04	1.53**
	(0.08)	(0.12)	(0.05)	(0.02)	(0.03)	(0.06)	(0.05)	(0.04)	(0.03)	(0.02)	(0.04)	(0.11)	(0.04)	(0.02)	(0.05)	(0.03)	(0.01)	(0.04)	(0.02)	(0.16)
4. Cassava	0.69	0.29	0.49**	-1.06**	-0.09	-0.06	-0.25*	0.04	0.34**	-0.05	-0.03	0.11	-0.54**	-0.16*	-0.13	0.01	-0.01	0.02	-0.02	1.09*
	(0.36)	(0.32)	(0.11)	(0.08)	(0.10)	(0.13)	(0.10)	(0.14)	(0.10)	(0.05)	(0.11)	(0.35)	(0.15)	(0.07)	(0.12)	(0.09)	(0.05)	(0.11)	(0.07)	(0.46)
5. Roots	0.19	0.71	-0.40	-0.14	-1.00**	0.27	-0.51	0.18	0.09	0.44*	-0.38	-0.01	-0.40	-0.33	-0.83	-0.23	0.12	-0.34	-0.36	2.49
	(0.46)	(0.87)	(0.27)	(0.17)	(0.37)	(0.50)	(0.34)	(0.40)	(0.24)	(0.21)	(0.48)	(1.19)	(0.41)	(0.23)	(0.59)	(0.19)	(0.13)	(0.37)	(0.22)	(1.80)
6. Sugar	-0.07	0.28	-0.22	-0.06	0.23	-0.66	0.02	-0.26	-0.23	-0.14	0.66	0.73	0.93	0.27	0.57	0.07	0.14	-0.15	-0.14	1.16
	(0.59)	(1.44)	(0.60)	(0.21)	(0.58)	(0.98)	(0.43)	(0.73)	(0.52)	(0.22)	(0.92)	(2.25)	(1.15)	(0.40)	(1.02)	(0.25)	(0.20)	(0.42)	(0.28)	(3.01)
7. Pulses	-0.01	0.00	-0.24	-0.17*	-0.23	0.01	-1.44**	-0.12	-0.03	0.12	0.11	0.35	-0.52*	0.06	-0.09	-0.01	0.02	-0.38*	0.05	1.03
	(0.37)	(0.58)	(0.13)	(0.08)	(0.14)	(0.18)	(0.30)	(0.12)	(0.13)	(0.08)	(0.15)	(0.34)	(0.21)	(0.08)	(0.15)	(0.14)	(0.07)	(0.17)	(0.12)	(0.55)
8. Nuts	-0.02	0.05	0.45	0.04	0.13	-0.21	-0.14	-0.76	-0.03	-0.07	-0.21	-0.01	-0.24	-0.00	-0.19	-0.06	-0.29	0.19	0.01	0.22
	(0.34)	(0.73)	(0.35)	(0.17)	(0.32)	(0.55)	(0.22)	(0.56)	(0.22)	(0.14)	(0.54)	(1.40)	(0.41)	(0.20)	(0.43)	(0.15)	(0.19)	(0.37)	(0.12)	(1.75)
9. Vegetables	0.14	-1.58*	0.11	0.26*	0.07	-0.17	-0.04	-0.05	-0.59*	-0.04	-0.06	-0.37	-0.33	0.03	0.02	0.11	0.01	-0.14	0.76**	1.48*
	(0.41)	(0.70)	(0.12)	(0.12)	(0.13)	(0.22)	(0.16)	(0.15)	(0.23)	(0.06)	(0.15)	(0.44)	(0.21)	(0.07)	(0.18)	(0.13)	(0.05)	(0.13)	(0.29)	(0.67)
10. Fruit	0.33	0.28	0.35**	-0.08	0.47**	-0.20	0.22	-0.13	-0.09	-0.96**	-0.25	-0.49	-0.05	0.09	-0.24	-0.40**	-0.01	0.36*	0.02	1.91**
	(0.26)	(0.43)	(0.12)	(0.07)	(0.15)	(0.17)	(0.15)	(0.15)	(0.10)	(0.09)	(0.18)	(0.35)	(0.14)	(0.08)	(0.14)	(0.13)	(0.06)	(0.15)	(0.07)	(0.57)
11. RedMeat	-0.03	-1.11*	-0.20	-0.04	-0.12	0.20	-0.01	-0.12	-0.11	-0.09	-1.02**	0.49	0.12	0.06	-0.05	-0.04	-0.07	0.17	-0.01	4.18**
	(0.18)	(0.46)	(0.12)	(0.05)	(0.14)	(0.27)	(0.11)	(0.20)	(0.10)	(0.06)	(0.26)	(0.47)	(0.14)	(0.08)	(0.24)	(0.07)	(0.06)	(0.15)	(0.07)	(1.07)
12. Poultry	-0.78	-0.22	-0.57	0.01	-0.01	0.19	0.11	-0.03	-0.25	-0.14	0.41	0.18	0.49	0.13	0.04	-0.08	0.01	-0.41	-0.21	4.31
	(0.46)	(1.00)	(0.46)	(0.15)	(0.35)	(0.59)	(0.21)	(0.50)	(0.30)	(0.12)	(0.47)	(1.62)	(0.48)	(0.20)	(0.44)	(0.13)	(0.07)	(0.29)	(0.17)	(2.43)
13. Eggs	-1.04	0.93	0.03	-0.87*	-0.46	1.30	-1.27*	-0.43	-0.74	-0.07	0.44	2.11	0.03	-0.08	-0.66	-0.53	0.14	0.07	-0.47	3.00
	(0.76)	(1.30)	(0.38)	(0.36)	(0.48)	(0.83)	(0.60)	(0.58)	(0.44)	(0.17)	(0.57)	(1.87)	(0.66)	(0.26)	(0.63)	(0.28)	(0.17)	(0.36)	(0.30)	(2.55)
14. Fish	0.18	-0.55	-0.12	-0.18*	-0.26	0.22	0.04	-0.02	-0.02	0.06	0.14	0.36	-0.05	-1.16**	0.09	-0.13	0.00	0.04	-0.08	3.22**
	(0.25)	(0.54)	(0.14)	(0.09)	(0.18)	(0.27)	(0.14)	(0.23)	(0.13)	(0.08)	(0.25)	(0.61)	(0.20)	(0.18)	(0.23)	(0.09)	(0.07)	(0.23)	(0.08)	(0.78)
15. Dairy	0.04	-0.76	0.10	-0.07	-0.27	0.25	-0.07	-0.12	0.01	-0.07	0.04	0.18	-0.19	0.07	-0.82**	0.00	0.07	-0.04	0.07	1.28*
	(0.15)	(0.43)	(0.10)	(0.06)	(0.17)	(0.24)	(0.10)	(0.15)	(0.09)	(0.05)	(0.18)	(0.46)	(0.16)	(0.07)	(0.20)	(0.05)	(0.05)	(0.11)	(0.06)	(0.62)
16. Oils	1.26*	-0.24	-0.23**	0.00	-0.10	0.04	-0.02	-0.07	0.11	-0.19**	0.09	0.04	-0.21*	-0.04	-0.00	-0.75**	0.02	0.04	0.10	1.54**
	(0.57)	(0.63)	(0.09)	(0.06)	(0.07)	(0.10)	(0.13)	(0.07)	(0.10)	(0.06)	(0.08)	(0.17)	(0.08)	(0.04)	(0.08)	(0.21)	(0.04)	(0.08)	(0.08)	(0.35)
17. Coffee	0.25	-0.70	0.12	-0.02	0.14	0.17	0.02	-0.49**	-0.01	-0.01	-0.20	0.08	0.14	0.00	0.18	0.00	-0.68**	0.51**	-0.09	2.32**
	(0.26)	(0.42)	(0.09)	(0.08)	(0.13)	(0.16)	(0.15)	(0.14)	(0.10)	(0.07)	(0.18)	(0.28)	(0.15)	(0.09)	(0.15)	(0.09)	(0.09)	(0.17)	(0.09)	(0.44)
18. SoftDrink	-0.76	-0.81	0.05	-0.01	-0.33	-0.21	-0.80*	0.24	-0.31	0.31	0.49	-1.44	0.06	0.05	-0.18	-0.01	0.43	-0.04	-0.09	3.84*
	(0.57)	(1.06)	(0.26)	(0.18)	(0.31)	(0.38)	(0.41)	(0.42)	(0.24)	(0.19)	(0.44)	(0.94)	(0.31)	(0.27)	(0.36)	(0.17)	(0.23)	(0.69)	(0.17)	(1.80)
19. OtherFood	1.17**	-0.86**	-0.06	-0.01	-0.14*	-0.08	0.05	-0.02	0.69**	0.03	0.19**	-0.14	-0.17*	0.01	0.11	0.11	-0.02	0.01	-1.21**	1.00**
	(0.32)	(0.29)	(0.06)	(0.04)	(0.07)	(0.08)	(0.11)	(0.06)	(0.12)	(0.03)	(0.07)	(0.13)	(0.07)	(0.04)	(0.08)	(0.08)	(0.03)	(0.07)	(0.07)	(0.25)

Note: This table shows the sample-wide median elasticity of food demand (quantity consumed) with respect to food prices (columns 1 thru 19) and total household expenditures (the last column). For a list of items in each food group, see Suppl. Table 47.

Suppl. Table 7: Food demand elasticities with respect to food prices and total household expenditures for Q3 consumers (with per capita expenditures between \$3.20 and \$5.50 per day) in Niger

	Food Group																			Exp.
	Rice	Millet	Wheat	Cassava	Roots	Sugar	Pulses	Nuts	Vegetables	Fruit	RedMeat	Poultry	Eggs	Fish	Dairy	Oils	Coffee	SoftDrink	OtherFood	
1. Rice	-0.48	-2.15*	-0.52**	0.28	0.02	-0.04	-0.18	0.00	-0.15	0.13	0.12	-0.58*	-0.29	0.06	-0.09	0.71*	0.02	-0.16	0.79**	1.68**
	(1.56)	(0.91)	(0.16)	(0.15)	(0.10)	(0.17)	(0.16)	(0.11)	(0.19)	(0.08)	(0.16)	(0.27)	(0.16)	(0.07)	(0.14)	(0.34)	(0.06)	(0.12)	(0.25)	(0.40)
2. Millet	-0.90	-1.29*	-0.09	0.08	0.07	0.14	0.02	-0.00	-0.48*	0.15	-0.05	0.36	0.25	0.01	-0.13	-0.03	-0.00	-0.02	-0.09	0.11
	(0.53)	(0.53)	(0.10)	(0.07)	(0.09)	(0.18)	(0.11)	(0.10)	(0.20)	(0.08)	(0.14)	(0.32)	(0.15)	(0.06)	(0.13)	(0.20)	(0.04)	(0.09)	(0.09)	(0.52)
3. Wheat	-0.42**	-0.40**	-0.85**	0.09**	-0.07*	-0.02	-0.08	0.09*	0.03	0.08**	0.06	-0.23*	0.00	-0.01	-0.06	-0.10*	0.04**	0.04	-0.01	1.41**
	(0.11)	(0.13)	(0.05)	(0.02)	(0.03)	(0.06)	(0.04)	(0.04)	(0.03)	(0.02)	(0.05)	(0.12)	(0.04)	(0.02)	(0.05)	(0.05)	(0.01)	(0.04)	(0.02)	(0.14)
4. Cassava	0.83	0.43	0.32**	-1.05**	-0.05	-0.04	-0.21*	-0.02	0.41**	-0.10	0.17	0.00	-0.50**	-0.17*	0.02	-0.13	0.05	-0.05	-0.04	0.98*
	(0.43)	(0.37)	(0.10)	(0.09)	(0.11)	(0.15)	(0.10)	(0.14)	(0.12)	(0.05)	(0.12)	(0.37)	(0.15)	(0.07)	(0.13)	(0.12)	(0.05)	(0.12)	(0.07)	(0.45)
5. Roots	0.01	0.29	-0.39	-0.06	-0.90*	0.44	-0.78	-0.08	0.15	0.32	-0.44	-0.14	-0.30	-0.19	-0.86	-0.18	0.21	-0.34	-0.27	1.90
	(0.46)	(0.76)	(0.27)	(0.16)	(0.37)	(0.55)	(0.40)	(0.43)	(0.24)	(0.19)	(0.53)	(1.20)	(0.41)	(0.21)	(0.60)	(0.19)	(0.15)	(0.36)	(0.20)	(1.56)
6. Sugar	-0.07	0.82	-0.01	-0.04	0.39	-0.63	-0.08	0.10	-0.17	-0.30	0.58	0.73	0.90	0.19	0.65	0.23	-0.02	-0.27	-0.15	0.56
	(0.82)	(1.62)	(0.52)	(0.24)	(0.69)	(1.05)	(0.49)	(0.77)	(0.54)	(0.35)	(0.96)	(2.31)	(1.16)	(0.36)	(1.06)	(0.40)	(0.17)	(0.49)	(0.30)	(3.01)
7. Pulses	-0.30	0.01	-0.13	-0.14	-0.39*	-0.06	-1.50**	-0.06	-0.14	0.20*	0.16	0.36	-0.31	-0.04	-0.13	-0.06	0.05	-0.48*	0.13	0.75
	(0.36)	(0.49)	(0.14)	(0.08)	(0.16)	(0.22)	(0.24)	(0.13)	(0.14)	(0.09)	(0.21)	(0.38)	(0.21)	(0.08)	(0.18)	(0.12)	(0.06)	(0.21)	(0.11)	(0.57)
8. Nuts	0.10	0.18	0.42	-0.00	-0.05	0.11	-0.05	-0.92	-0.13	-0.14	0.03	0.33	0.07	-0.14	-0.00	-0.01	-0.21	0.12	-0.03	-0.18
	(0.45)	(0.82)	(0.39)	(0.18)	(0.37)	(0.67)	(0.25)	(0.63)	(0.28)	(0.17)	(0.63)	(1.63)	(0.49)	(0.26)	(0.51)	(0.18)	(0.16)	(0.38)	(0.16)	(1.85)
9. Vegetables	-0.33	-2.44**	0.05	0.30*	0.10	-0.15	-0.18	-0.15	-0.58*	-0.06	-0.17	-0.36	-0.38	0.11	-0.11	0.46*	0.02	-0.23	0.90**	1.59**
	(0.44)	(0.88)	(0.12)	(0.13)	(0.13)	(0.25)	(0.16)	(0.17)	(0.25)	(0.07)	(0.18)	(0.47)	(0.23)	(0.08)	(0.19)	(0.20)	(0.05)	(0.14)	(0.32)	(0.60)
10. Fruit	0.48	0.92*	0.31*	-0.13*	0.34*	-0.38	0.37*	-0.22	-0.12	-1.01**	-0.40*	-0.30	-0.11	0.00	-0.07	-0.36**	0.08	0.47**	-0.07	1.76**
	(0.29)	(0.46)	(0.12)	(0.07)	(0.15)	(0.20)	(0.14)	(0.15)	(0.11)	(0.10)	(0.20)	(0.32)	(0.14)	(0.08)	(0.15)	(0.13)	(0.07)	(0.16)	(0.08)	(0.48)
11. RedMeat	-0.02	-0.62	-0.14	0.04	-0.13	0.15	0.02	-0.02	-0.15	-0.13*	-1.27**	0.31	0.12	0.01	0.04	-0.02	-0.09	0.06	0.08	3.26**
	(0.21)	(0.36)	(0.10)	(0.05)	(0.14)	(0.23)	(0.10)	(0.18)	(0.10)	(0.07)	(0.27)	(0.44)	(0.15)	(0.08)	(0.20)	(0.07)	(0.05)	(0.15)	(0.07)	(0.81)
12. Poultry	-0.71	0.36	-0.46	-0.02	-0.05	0.19	0.13	0.10	-0.22	-0.09	0.27	0.33	0.43	0.06	0.07	-0.08	-0.02	-0.30	-0.20	2.95
	(0.43)	(0.74)	(0.36)	(0.15)	(0.34)	(0.57)	(0.21)	(0.48)	(0.27)	(0.10)	(0.48)	(1.67)	(0.48)	(0.19)	(0.42)	(0.13)	(0.07)	(0.27)	(0.15)	(1.85)
13. Eggs	-1.31	2.17	-0.00	-0.76*	-0.36	1.24	-0.67	0.10	-0.76	-0.13	0.47	1.90	0.71	-0.19	-0.39	-0.60	0.02	0.05	-0.82*	1.29
	(0.89)	(1.47)	(0.37)	(0.33)	(0.47)	(0.87)	(0.50)	(0.62)	(0.48)	(0.18)	(0.66)	(1.94)	(0.88)	(0.28)	(0.66)	(0.32)	(0.16)	(0.38)	(0.38)	(2.27)
14. Fish	0.05	-0.35	-0.22	-0.19*	-0.16	0.14	-0.11	-0.18	0.09	-0.01	-0.01	0.17	-0.13	-1.08**	0.14	-0.04	0.00	0.02	-0.12	2.84**
	(0.26)	(0.47)	(0.14)	(0.09)	(0.17)	(0.27)	(0.12)	(0.23)	(0.13)	(0.08)	(0.27)	(0.61)	(0.21)	(0.17)	(0.22)	(0.09)	(0.06)	(0.21)	(0.08)	(0.64)
15. Dairy	-0.08	-0.43	-0.04	0.01	-0.29	0.28	-0.09	-0.03	-0.05	-0.01	0.16	0.19	-0.12	0.10	-0.72**	-0.06	0.01	-0.07	0.06	0.88
	(0.17)	(0.35)	(0.11)	(0.06)	(0.18)	(0.25)	(0.11)	(0.16)	(0.10)	(0.05)	(0.20)	(0.47)	(0.17)	(0.07)	(0.22)	(0.07)	(0.04)	(0.11)	(0.07)	(0.60)
16. Oils	1.51*	-0.36	-0.25*	-0.10	-0.08	0.13	-0.08	-0.04	0.44**	-0.18**	0.10	-0.04	-0.27**	0.01	-0.13	-0.61*	-0.03	0.03	-0.23*	1.50**
	(0.66)	(0.68)	(0.11)	(0.07)	(0.07)	(0.13)	(0.10)	(0.08)	(0.12)	(0.06)	(0.11)	(0.17)	(0.09)	(0.04)	(0.09)	(0.26)	(0.03)	(0.09)	(0.09)	(0.28)
17. Coffee	0.05	-0.21	0.12	0.06	0.24	-0.05	0.08	-0.35**	0.01	0.08	-0.32	-0.06	0.02	0.01	-0.02	-0.08	-0.71**	0.74**	-0.04	1.89**
	(0.24)	(0.37)	(0.08)	(0.07)	(0.13)	(0.15)	(0.12)	(0.12)	(0.09)	(0.08)	(0.18)	(0.27)	(0.13)	(0.07)	(0.13)	(0.08)	(0.08)	(0.16)	(0.08)	(0.34)
18. SoftDrink	-0.68	-0.57	-0.01	-0.08	-0.32	-0.33	-0.90*	0.13	-0.42	0.41*	0.17	-1.01	0.04	0.02	-0.24	-0.02	0.59*	-0.19	0.10	2.82*
	(0.53)	(0.86)	(0.25)	(0.16)	(0.30)	(0.37)	(0.42)	(0.38)	(0.25)	(0.20)	(0.49)	(0.87)	(0.31)	(0.24)	(0.33)	(0.18)	(0.26)	(0.61)	(0.16)	(1.22)
19. OtherFood	1.75**	-0.57	0.01	-0.03	-0.13	-0.12	0.12	-0.06	0.88**	-0.02	0.31**	-0.26	-0.39**	-0.03	0.08	-0.21*	-0.00	0.11	-1.12**	1.16**
	(0.40)	(0.29)	(0.07)	(0.04)	(0.07)	(0.10)	(0.09)	(0.07)	(0.15)	(0.04)	(0.11)	(0.15)	(0.10)	(0.04)	(0.09)	(0.09)	(0.03)	(0.08)	(0.08)	(0.24)

Note: This table shows the sample-wide median elasticity of food demand (quantity consumed) with respect to food prices (columns 1 thru 19) and total household expenditures (the last column). For a list of items in each food group, see Suppl. Table 47.

Suppl. Table 8: Food demand elasticities with respect to food prices and total household expenditures for Q4 consumers (with per capita expenditures greater than $5.50 per day) in Niger

	Food Group																			Exp.
	Rice	Millet	Wheat	Cassava	Roots	Sugar	Pulses	Nuts	Vegetables	Fruit	RedMeat	Poultry	Eggs	Fish	Dairy	Oils	Coffee	SoftDrink	OtherFood	
1. Rice	-0.79	-2.62*	-0.61**	0.31	-0.03	-0.05	-0.34	0.04	-0.37	0.17	0.13	-0.55	-0.38	0.02	-0.19	0.84*	-0.03	-0.15	1.03**	1.45**
	(1.82)	(1.06)	(0.21)	(0.18)	(0.13)	(0.23)	(0.21)	(0.15)	(0.24)	(0.12)	(0.22)	(0.32)	(0.22)	(0.10)	(0.18)	(0.42)	(0.06)	(0.17)	(0.35)	(0.30)
2. Millet	-1.89	-1.80	-0.16	0.16	-0.00	0.33	0.01	0.03	-1.10*	0.37	0.00	0.69	0.58	0.02	-0.06	-0.12	0.06	-0.06	0.01	-0.22
	(1.07)	(0.99)	(0.22)	(0.14)	(0.17)	(0.40)	(0.21)	(0.20)	(0.46)	(0.19)	(0.31)	(0.70)	(0.36)	(0.13)	(0.26)	(0.40)	(0.09)	(0.21)	(0.16)	(0.84)
3. Wheat	-0.62**	-0.44*	-0.87**	0.03	-0.10*	0.03	-0.04	0.08	0.02	0.09**	0.08	-0.29	-0.04	-0.06	-0.20*	-0.12	0.04	0.01	0.04	1.30**
	(0.16)	(0.18)	(0.06)	(0.03)	(0.05)	(0.07)	(0.06)	(0.05)	(0.05)	(0.03)	(0.07)	(0.15)	(0.06)	(0.03)	(0.08)	(0.07)	(0.02)	(0.05)	(0.04)	(0.14)
4. Cassava	1.02	0.59	0.12	-1.05**	0.00	-0.03	-0.17	-0.08	0.51**	-0.16*	0.41*	-0.12	-0.46**	-0.18	0.20	-0.29	0.11	-0.14	-0.07	0.93
	(0.55)	(0.50)	(0.13)	(0.12)	(0.15)	(0.21)	(0.14)	(0.18)	(0.16)	(0.07)	(0.19)	(0.49)	(0.18)	(0.10)	(0.19)	(0.17)	(0.06)	(0.16)	(0.09)	(0.48)
5. Roots	-0.14	-0.15	-0.35	0.00	-0.80	0.61	-1.02	-0.33	0.21	0.19	-0.48	-0.27	-0.19	-0.05	-0.86	-0.12	0.29	-0.34	-0.18	1.35
	(0.57)	(0.82)	(0.29)	(0.20)	(0.46)	(0.73)	(0.55)	(0.53)	(0.30)	(0.21)	(0.68)	(1.44)	(0.53)	(0.24)	(0.69)	(0.23)	(0.18)	(0.42)	(0.22)	(1.35)
6. Sugar	-0.10	1.37	0.19	-0.02	0.59	-0.60	-0.21	0.51	-0.13	-0.49	0.49	0.76	0.90	0.11	0.76	0.42	-0.20	-0.42	-0.19	0.32
	(1.39)	(2.57)	(0.78)	(0.36)	(1.08)	(1.60)	(0.76)	(1.23)	(0.72)	(0.61)	(1.28)	(3.34)	(1.55)	(0.45)	(1.54)	(0.73)	(0.32)	(0.81)	(0.45)	(3.72)
7. Pulses	-0.67	-0.04	-0.02	-0.12	-0.61*	-0.15	-1.60**	0.01	-0.28	0.32*	0.22	0.38	-0.07	-0.16	-0.19	-0.12	0.09	-0.62*	0.22	0.57
	(0.54)	(0.60)	(0.21)	(0.12)	(0.26)	(0.35)	(0.33)	(0.20)	(0.24)	(0.16)	(0.34)	(0.57)	(0.31)	(0.14)	(0.28)	(0.21)	(0.08)	(0.30)	(0.17)	(0.68)
8. Nuts	0.22	0.20	0.33	-0.06	-0.26	0.47	0.04	-1.11	-0.28	-0.23	0.30	0.76	0.44	-0.32	0.20	0.03	-0.13	0.04	-0.09	-0.11
	(0.74)	(1.09)	(0.45)	(0.25)	(0.56)	(1.00)	(0.38)	(0.87)	(0.44)	(0.26)	(0.93)	(2.35)	(0.81)	(0.40)	(0.74)	(0.26)	(0.18)	(0.50)	(0.26)	(2.00)
9. Vegetables	-0.84	-3.38*	0.02	0.35*	0.14	-0.11	-0.33	-0.26	-0.56	-0.09	-0.31	-0.34	-0.44	0.19	-0.26	0.86*	0.03	-0.34	1.04*	1.51**
	(0.62)	(1.31)	(0.14)	(0.15)	(0.17)	(0.31)	(0.24)	(0.23)	(0.32)	(0.10)	(0.25)	(0.58)	(0.29)	(0.13)	(0.26)	(0.38)	(0.06)	(0.21)	(0.41)	(0.51)
10. Fruit	0.60	1.50*	0.27	-0.18*	0.20	-0.55*	0.50*	-0.31	-0.15	-1.06**	-0.53*	-0.11	-0.17	-0.08	0.09	-0.28	0.16	0.56**	-0.15	1.48**
	(0.43)	(0.61)	(0.16)	(0.09)	(0.18)	(0.26)	(0.20)	(0.18)	(0.15)	(0.14)	(0.26)	(0.38)	(0.18)	(0.10)	(0.20)	(0.17)	(0.09)	(0.21)	(0.11)	(0.40)
11. RedMeat	0.04	-0.19	-0.02	0.12	-0.15	0.11	0.06	0.08	-0.18	-0.17*	-1.49**	0.15	0.12	-0.04	0.13	0.01	-0.11*	-0.03	0.15	2.32**
	(0.26)	(0.34)	(0.11)	(0.07)	(0.16)	(0.25)	(0.12)	(0.19)	(0.11)	(0.08)	(0.35)	(0.49)	(0.17)	(0.09)	(0.21)	(0.09)	(0.05)	(0.19)	(0.09)	(0.61)
12. Poultry	-0.62	0.72	-0.36	-0.05	-0.08	0.20	0.15	0.24	-0.18	-0.04	0.14	0.47	0.37	-0.01	0.09	-0.09	-0.05	-0.19	-0.21	1.96
	(0.47)	(0.82)	(0.33)	(0.18)	(0.41)	(0.70)	(0.27)	(0.57)	(0.31)	(0.12)	(0.59)	(2.06)	(0.58)	(0.23)	(0.53)	(0.16)	(0.09)	(0.33)	(0.16)	(1.62)
13. Eggs	-1.54	3.11	-0.11	-0.61	-0.23	1.14	-0.12	0.61	-0.79	-0.19	0.50	1.59	1.32	-0.29	-0.12	-0.66	-0.09	0.03	-1.19*	0.45
	(1.29)	(2.14)	(0.47)	(0.36)	(0.62)	(1.16)	(0.61)	(0.87)	(0.63)	(0.27)	(0.91)	(2.45)	(1.39)	(0.40)	(0.86)	(0.43)	(0.19)	(0.55)	(0.57)	(2.42)
14. Fish	-0.06	-0.08	-0.31	-0.18	-0.06	0.06	-0.27	-0.34	0.20	-0.08	-0.16	-0.03	-0.21	-0.99**	0.20	0.04	0.01	-0.00	-0.16	2.30**
	(0.35)	(0.51)	(0.17)	(0.10)	(0.19)	(0.30)	(0.17)	(0.27)	(0.17)	(0.10)	(0.34)	(0.70)	(0.27)	(0.19)	(0.26)	(0.12)	(0.06)	(0.24)	(0.10)	(0.49)
15. Dairy	-0.22	-0.19	-0.23	0.09	-0.31	0.30	-0.13	0.07	-0.13	0.05	0.27	0.19	-0.05	0.13	-0.63*	-0.15	-0.07	-0.11	0.04	0.90
	(0.25)	(0.37)	(0.16)	(0.08)	(0.23)	(0.34)	(0.16)	(0.21)	(0.14)	(0.07)	(0.28)	(0.61)	(0.23)	(0.10)	(0.31)	(0.10)	(0.06)	(0.16)	(0.10)	(0.61)
16. Oils	1.80*	-0.51	-0.25	-0.20*	-0.07	0.24	-0.15	-0.01	0.82**	-0.17*	0.11	-0.11	-0.35**	0.07	-0.26*	-0.44	-0.08	0.02	-0.59**	1.37**
	(0.83)	(0.79)	(0.16)	(0.10)	(0.09)	(0.17)	(0.15)	(0.09)	(0.21)	(0.08)	(0.16)	(0.20)	(0.12)	(0.06)	(0.11)	(0.37)	(0.05)	(0.12)	(0.15)	(0.22)
17. Coffee	-0.15	0.23	0.10	0.14	0.35*	-0.28	0.15	-0.21	0.04	0.19	-0.44*	-0.21	-0.10	0.01	-0.23	-0.19	-0.75**	1.00**	0.01	1.52**
	(0.29)	(0.42)	(0.10)	(0.08)	(0.16)	(0.18)	(0.13)	(0.16)	(0.11)	(0.10)	(0.22)	(0.33)	(0.15)	(0.08)	(0.16)	(0.11)	(0.09)	(0.20)	(0.09)	(0.31)
18. SoftDrink	-0.56	-0.42	-0.08	-0.14	-0.29	-0.40	-0.90	0.03	-0.49	0.45	-0.10	-0.53	0.02	-0.00	-0.28	-0.02	0.68*	-0.40	0.26	2.13*
	(0.60)	(0.85)	(0.26)	(0.18)	(0.33)	(0.45)	(0.48)	(0.39)	(0.31)	(0.24)	(0.62)	(0.96)	(0.37)	(0.24)	(0.37)	(0.22)	(0.30)	(0.63)	(0.22)	(0.88)
19. OtherFood	2.47**	-0.15	0.11	-0.06	-0.11	-0.15	0.21	-0.11	1.11**	-0.08	0.47**	-0.40	-0.69**	-0.09	0.05	-0.64**	0.01	0.24*	-1.01**	1.15**
	(0.59)	(0.35)	(0.10)	(0.06)	(0.10)	(0.15)	(0.13)	(0.11)	(0.21)	(0.06)	(0.17)	(0.21)	(0.16)	(0.06)	(0.13)	(0.17)	(0.04)	(0.12)	(0.12)	(0.22)

Note: This table shows the sample-wide median elasticity of food demand (quantity consumed) with respect to food prices (columns 1 thru 19) and total household expenditures (the last column). For a list of items in each food group, see Suppl. Table 47.

Suppl. Table 9: Food demand elasticities with respect to food prices and total household expenditures for Q1 consumers (with per capita expenditures less than $1.90 per day) in Uganda

	Food Group																			Exp.
	Rice	Maize	Wheat	Cassava	Roots	Sugar	Pulses	Nuts	Vegetables	Fruit	RedMeat	Poultry	Eggs	Fish	Dairy	Fats	Coffee	SoftDrink	OtherFood	
1. Rice	-2.18**	0.71**	-0.02	0.46*	0.46*	-0.10	0.47*	-0.42**	-0.88**	0.12	0.13	1.04**	-0.05	-0.27	0.41*	0.12	0.00	0.55	-0.04*	2.44**
	(0.34)	(0.25)	(0.20)	(0.23)	(0.22)	(0.12)	(0.19)	(0.11)	(0.18)	(0.13)	(0.36)	(0.34)	(0.16)	(0.17)	(0.18)	(0.07)	(0.02)	(0.30)	(0.02)	(0.50)
2. Maize	0.28**	-0.86**	-0.05	-0.20*	0.04	0.05	-0.17**	0.03	0.10*	0.07	0.03	-0.04	0.11*	0.11	-0.12	0.02	0.01*	-0.11	-0.01*	0.87**
	(0.07)	(0.11)	(0.07)	(0.09)	(0.08)	(0.03)	(0.06)	(0.03)	(0.05)	(0.05)	(0.10)	(0.10)	(0.05)	(0.07)	(0.06)	(0.02)	(0.01)	(0.08)	(0.00)	(0.14)
3. Wheat	0.01	-0.09	-0.87**	-0.09	-0.14	-0.13**	-0.16*	-0.03	0.10*	0.04	0.27*	-0.26	-0.07	-0.16*	0.02	-0.05*	-0.02**	-0.09	-0.00	1.10**
	(0.09)	(0.10)	(0.09)	(0.10)	(0.10)	(0.04)	(0.07)	(0.04)	(0.05)	(0.06)	(0.12)	(0.14)	(0.05)	(0.07)	(0.07)	(0.02)	(0.01)	(0.10)	(0.00)	(0.19)
4. Cassava	0.19**	-0.18*	-0.05	-0.50**	-0.08	0.04	0.05	-0.02	-0.11*	-0.05	-0.20*	-0.07	0.00	0.22**	-0.04	-0.03	0.01	0.01	-0.01**	0.78**
	(0.07)	(0.09)	(0.07)	(0.13)	(0.08)	(0.03)	(0.06)	(0.03)	(0.05)	(0.05)	(0.10)	(0.11)	(0.05)	(0.07)	(0.07)	(0.02)	(0.01)	(0.09)	(0.00)	(0.15)
5. Roots	0.18**	0.02	-0.07	-0.09	-1.04**	0.07*	-0.11*	-0.03	-0.05	-0.01	0.23*	0.17	-0.03	0.10	-0.03	-0.06**	-0.01	0.05	-0.00	1.03**
	(0.06)	(0.07)	(0.06)	(0.07)	(0.10)	(0.03)	(0.06)	(0.03)	(0.04)	(0.05)	(0.09)	(0.11)	(0.05)	(0.06)	(0.05)	(0.02)	(0.01)	(0.09)	(0.00)	(0.13)
6. Sugar	-0.07	0.11	-0.29**	0.07	0.16	0.11	0.27**	0.12*	-0.54**	0.00	-0.48	0.23	-0.00	-0.12	0.08	-0.11	-0.03	0.22	-0.04*	1.58**
	(0.12)	(0.11)	(0.09)	(0.11)	(0.11)	(0.19)	(0.10)	(0.06)	(0.14)	(0.08)	(0.25)	(0.16)	(0.09)	(0.08)	(0.08)	(0.07)	(0.03)	(0.16)	(0.01)	(0.24)
7. Pulses	0.24**	-0.19**	-0.12*	0.06	-0.13	0.14**	-1.14**	0.05	0.16**	0.01	-0.20	0.15	0.04	0.11	0.01	0.01	0.03**	-0.32**	-0.00	0.70**
	(0.07)	(0.07)	(0.06)	(0.07)	(0.07)	(0.04)	(0.08)	(0.03)	(0.05)	(0.04)	(0.11)	(0.08)	(0.04)	(0.06)	(0.06)	(0.02)	(0.01)	(0.10)	(0.00)	(0.11)
8. Nuts	-0.28**	0.06	-0.06	-0.09	-0.09	0.10*	0.08	-1.03**	0.01	-0.12**	0.12	0.22**	0.12*	0.04	0.06	0.06*	-0.02*	0.10	-0.01	1.15**
	(0.07)	(0.07)	(0.06)	(0.07)	(0.07)	(0.04)	(0.07)	(0.04)	(0.06)	(0.05)	(0.14)	(0.08)	(0.05)	(0.05)	(0.05)	(0.03)	(0.01)	(0.10)	(0.01)	(0.12)
9. Vegetables	-0.50**	0.21*	0.15*	-0.20*	-0.05	-0.31**	0.27**	0.03	-1.37**	-0.04	0.28	0.20	0.05	-0.26**	0.08	0.05	-0.03	0.41**	-0.04**	0.53**
	(0.12)	(0.10)	(0.07)	(0.09)	(0.09)	(0.09)	(0.10)	(0.05)	(0.17)	(0.06)	(0.19)	(0.13)	(0.06)	(0.08)	(0.08)	(0.05)	(0.02)	(0.15)	(0.01)	(0.19)
10. Fruit	0.12	0.13	0.06	-0.18	-0.07	-0.00	-0.03	-0.15**	-0.10	-0.96**	0.20	-0.27	0.08	0.22*	-0.24*	0.05	0.00	0.26	-0.00	1.69**
	(0.10)	(0.12)	(0.10)	(0.11)	(0.15)	(0.07)	(0.10)	(0.05)	(0.08)	(0.11)	(0.15)	(0.18)	(0.10)	(0.10)	(0.10)	(0.04)	(0.01)	(0.15)	(0.01)	(0.26)
11. RedMeat	0.07	-0.05	0.21	-0.39**	0.22	-0.24*	-0.34*	0.04	0.14	0.10	-2.08**	-0.69**	0.29**	0.21	-0.24*	0.02	-0.03	0.18	-0.04**	2.34**
	(0.14)	(0.14)	(0.11)	(0.13)	(0.14)	(0.11)	(0.13)	(0.07)	(0.12)	(0.08)	(0.32)	(0.22)	(0.11)	(0.11)	(0.12)	(0.06)	(0.02)	(0.21)	(0.01)	(0.39)
12. Poultry	0.44**	-0.25	-0.32*	-0.28	0.01	0.04	-0.06	0.04	-0.01	-0.18*	-0.72**	-4.22**	0.08	0.48*	-0.57**	0.13*	0.04*	-0.12	-0.01	4.03**
	(0.13)	(0.16)	(0.13)	(0.15)	(0.19)	(0.06)	(0.11)	(0.05)	(0.10)	(0.09)	(0.23)	(0.91)	(0.08)	(0.20)	(0.19)	(0.05)	(0.01)	(0.14)	(0.01)	(1.23)
13. Eggs	-0.12	0.67	-0.37	-0.07	-0.33	-0.03	0.14	0.33*	0.12	0.22	1.42*	0.42	-1.20**	0.17	-0.49	-0.03	0.04	0.84	-0.01	2.00**
	(0.34)	(0.35)	(0.26)	(0.33)	(0.40)	(0.20)	(0.25)	(0.15)	(0.23)	(0.26)	(0.58)	(0.44)	(0.29)	(0.25)	(0.26)	(0.13)	(0.04)	(0.49)	(0.02)	(0.60)
14. Fish	-0.14	0.16	-0.19*	0.37**	0.17	-0.07	0.11	0.02	-0.27**	0.16*	0.30	0.74*	0.05	-1.13**	0.21*	0.01	0.00	0.22	0.00	1.30**
	(0.10)	(0.13)	(0.10)	(0.13)	(0.13)	(0.05)	(0.10)	(0.04)	(0.08)	(0.07)	(0.17)	(0.32)	(0.07)	(0.15)	(0.11)	(0.03)	(0.01)	(0.13)	(0.01)	(0.37)
15. Dairy	0.31**	-0.32*	0.01	-0.17	-0.14	0.05	-0.06	0.04	0.04	-0.21*	-0.37	-0.89**	-0.15	0.25*	-1.06**	0.14**	0.01	0.08	-0.01	1.90**
	(0.12)	(0.14)	(0.11)	(0.14)	(0.13)	(0.05)	(0.11)	(0.04)	(0.09)	(0.08)	(0.20)	(0.30)	(0.08)	(0.12)	(0.15)	(0.04)	(0.01)	(0.15)	(0.01)	(0.45)
16. Fats	0.24	0.11	-0.16*	-0.14	-0.36**	-0.16	0.01	0.12	0.10	0.12	0.14	0.66**	-0.01	0.05	0.34**	-0.52**	-0.00	-0.12	-0.04*	1.02**
	(0.13)	(0.12)	(0.08)	(0.11)	(0.13)	(0.12)	(0.11)	(0.07)	(0.13)	(0.08)	(0.25)	(0.25)	(0.10)	(0.09)	(0.10)	(0.13)	(0.03)	(0.17)	(0.01)	(0.31)
17. Coffee	0.05	0.25	-0.27**	0.19	-0.17	-0.23	0.44**	-0.19*	-0.31	0.03	-0.37	0.74*	0.14	0.04	0.12	-0.02	-0.80**	0.56**	0.02	1.01**
	(0.15)	(0.15)	(0.10)	(0.14)	(0.15)	(0.22)	(0.14)	(0.09)	(0.25)	(0.10)	(0.35)	(0.29)	(0.14)	(0.11)	(0.10)	(0.13)	(0.10)	(0.21)	(0.04)	(0.38)
18. SoftDrink	0.80	-0.57	-0.33	-0.05	0.14	0.29	-1.33*	0.14	0.85	0.42	0.53	-0.39	0.54	0.49	0.13	-0.13	0.11*	-0.92	0.12**	2.73*
	(0.51)	(0.52)	(0.41)	(0.50)	(0.55)	(0.28)	(0.53)	(0.24)	(0.44)	(0.38)	(0.92)	(0.67)	(0.41)	(0.38)	(0.40)	(0.18)	(0.05)	(0.96)	(0.04)	(1.08)
19. OtherFood	-0.11	-0.08	-0.01	-0.12*	0.05	-0.12	0.04	-0.02	-0.27**	0.02	-0.29*	0.17*	0.00	0.05	-0.03	-0.09*	0.02	0.40**	-0.31**	0.24
	(0.06)	(0.06)	(0.04)	(0.06)	(0.05)	(0.06)	(0.05)	(0.03)	(0.09)	(0.04)	(0.12)	(0.07)	(0.04)	(0.04)	(0.04)	(0.03)	(0.02)	(0.09)	(0.09)	(0.13)

Note: This table shows the sample-wide median elasticity of food demand (quantity consumed) with respect to food prices (columns 1 thru 19) and total household expenditures (the last column). For a list of items in each food group, see Suppl. Table 48.

Suppl. Table 10: Food demand elasticities with respect to food prices and total household expenditures for Q2 consumers (with per capita expenditures between $1.90 and $3.20 per day) in Uganda

	Food Group																			Exp.
	Rice	Maize	Wheat	Cassava	Roots	Sugar	Pulses	Nuts	Vegetables	Fruit	RedMeat	Poultry	Eggs	Fish	Dairy	Fats	Coffee	SoftDrink	OtherFood	
1. Rice	-1.22**	0.26	-0.05	0.20	0.22	0.03	0.13	-0.27**	-0.46**	0.07	0.02	0.48**	-0.05	-0.19	0.15	0.06	0.02	0.07	-0.02	1.84**
	(0.22)	(0.16)	(0.13)	(0.17)	(0.16)	(0.09)	(0.12)	(0.07)	(0.12)	(0.09)	(0.26)	(0.18)	(0.12)	(0.12)	(0.12)	(0.05)	(0.01)	(0.19)	(0.01)	(0.28)
2. Maize	0.13*	-0.82**	-0.05	-0.15	0.16*	0.03	-0.08	0.01	0.10*	0.06	0.08	-0.03	0.15**	0.11	-0.10	0.04*	0.01	0.04	-0.00	0.73**
	(0.06)	(0.10)	(0.06)	(0.08)	(0.07)	(0.03)	(0.05)	(0.03)	(0.04)	(0.05)	(0.09)	(0.09)	(0.05)	(0.06)	(0.06)	(0.02)	(0.01)	(0.08)	(0.00)	(0.12)
3. Wheat	-0.02	-0.09	-0.83**	-0.05	-0.08	-0.08*	-0.06	0.01	0.04	0.05	0.04	-0.12	-0.05	-0.12	-0.01	-0.01	-0.01	-0.03	-0.00	1.08**
	(0.07)	(0.08)	(0.08)	(0.10)	(0.09)	(0.03)	(0.06)	(0.03)	(0.05)	(0.06)	(0.11)	(0.11)	(0.05)	(0.07)	(0.06)	(0.02)	(0.00)	(0.10)	(0.00)	(0.15)
4. Cassava	0.11	-0.14	-0.02	-0.42**	-0.05	0.02	0.02	-0.04	-0.07	0.03	-0.07	-0.03	-0.02	0.13*	-0.09	-0.03	0.00	0.03	-0.01**	0.69**
	(0.07)	(0.09)	(0.07)	(0.13)	(0.09)	(0.03)	(0.06)	(0.03)	(0.05)	(0.04)	(0.09)	(0.09)	(0.05)	(0.06)	(0.06)	(0.02)	(0.01)	(0.10)	(0.00)	(0.14)
5. Roots	0.11*	0.12	-0.03	-0.05	-0.92**	0.04	-0.01	-0.02	-0.06	0.00	0.04	0.04	-0.02	0.11*	-0.02	-0.03*	-0.00	0.01	-0.00	0.82**
	(0.05)	(0.06)	(0.05)	(0.06)	(0.08)	(0.03)	(0.04)	(0.02)	(0.03)	(0.04)	(0.07)	(0.09)	(0.05)	(0.04)	(0.04)	(0.02)	(0.00)	(0.07)	(0.00)	(0.10)
6. Sugar	0.05	0.03	-0.15*	0.02	0.10	-0.38**	0.05	0.07	-0.37**	-0.00	-0.41*	0.09	-0.04	-0.11*	-0.06	0.02	-0.05*	0.21	-0.03**	1.24**
	(0.09)	(0.08)	(0.06)	(0.08)	(0.09)	(0.11)	(0.07)	(0.04)	(0.10)	(0.06)	(0.17)	(0.09)	(0.07)	(0.06)	(0.06)	(0.05)	(0.02)	(0.11)	(0.01)	(0.15)
7. Pulses	0.11	-0.09	-0.04	0.03	0.02	0.05	-1.13**	0.00	0.06	0.08	0.02	0.10	0.10*	0.01	0.01	-0.05*	0.02**	-0.08	-0.00	0.55**
	(0.06)	(0.06)	(0.05)	(0.07)	(0.07)	(0.03)	(0.07)	(0.03)	(0.05)	(0.04)	(0.10)	(0.07)	(0.04)	(0.05)	(0.05)	(0.02)	(0.01)	(0.09)	(0.00)	(0.10)
8. Nuts	-0.21**	-0.00	0.02	-0.11*	-0.07	0.07	-0.02	-0.95**	0.07	-0.04	0.20*	0.09	0.10**	-0.01	0.10*	-0.01	-0.01*	0.06	-0.00	1.00**
	(0.06)	(0.06)	(0.04)	(0.05)	(0.06)	(0.03)	(0.05)	(0.04)	(0.05)	(0.04)	(0.10)	(0.06)	(0.04)	(0.04)	(0.04)	(0.02)	(0.01)	(0.08)	(0.00)	(0.09)
9. Vegetables	-0.40**	0.21*	0.07	-0.17	-0.17	-0.30**	0.08	0.08	-1.57**	-0.07	0.49*	0.17	0.12	-0.10	0.09	0.12*	-0.01	0.60**	-0.06**	0.84**
	(0.11)	(0.09)	(0.07)	(0.10)	(0.10)	(0.09)	(0.09)	(0.05)	(0.19)	(0.06)	(0.19)	(0.10)	(0.07)	(0.07)	(0.07)	(0.05)	(0.02)	(0.16)	(0.02)	(0.16)
10. Fruit	0.08	0.10	0.07	0.03	-0.02	-0.01	0.09	-0.05	-0.09	-0.92**	0.03	-0.14	0.05	0.13	-0.10	0.02	0.00	0.14	0.01	1.31**
	(0.08)	(0.11)	(0.08)	(0.09)	(0.13)	(0.05)	(0.08)	(0.04)	(0.06)	(0.10)	(0.12)	(0.13)	(0.08)	(0.07)	(0.08)	(0.03)	(0.01)	(0.12)	(0.01)	(0.17)
11. RedMeat	0.02	0.04	0.02	-0.13	-0.03	-0.19**	-0.04	0.09	0.22*	0.01	-1.65**	-0.28*	0.05	0.13	-0.13	-0.00	-0.01	-0.03	-0.00	1.62**
	(0.11)	(0.10)	(0.08)	(0.10)	(0.11)	(0.07)	(0.09)	(0.05)	(0.09)	(0.06)	(0.24)	(0.13)	(0.09)	(0.08)	(0.09)	(0.05)	(0.01)	(0.15)	(0.01)	(0.21)
12. Poultry	0.22**	-0.12	-0.12	-0.11	-0.10	0.01	-0.00	0.01	0.04	-0.10	-0.34*	-2.26**	0.03	0.25*	-0.27**	0.08*	0.02	-0.07	-0.00	2.47**
	(0.08)	(0.10)	(0.08)	(0.10)	(0.15)	(0.04)	(0.07)	(0.03)	(0.06)	(0.07)	(0.13)	(0.52)	(0.07)	(0.11)	(0.10)	(0.03)	(0.01)	(0.11)	(0.00)	(0.60)
13. Eggs	-0.13	0.80**	-0.20	-0.15	-0.19	-0.10	0.40	0.25*	0.28	0.13	0.25	0.14	-1.17**	0.14	-0.36	-0.02	0.02	0.04	0.04*	1.61**
	(0.29)	(0.31)	(0.22)	(0.31)	(0.36)	(0.16)	(0.21)	(0.11)	(0.19)	(0.22)	(0.49)	(0.38)	(0.27)	(0.20)	(0.21)	(0.10)	(0.03)	(0.42)	(0.02)	(0.44)
14. Fish	-0.12	0.15	-0.14	0.17	0.18	-0.07*	-0.03	-0.01	-0.09	0.10	0.19	0.39*	0.04	-1.05**	0.06	-0.01	-0.00	0.12	0.01	1.27**
	(0.07)	(0.09)	(0.07)	(0.10)	(0.10)	(0.04)	(0.07)	(0.03)	(0.05)	(0.05)	(0.11)	(0.18)	(0.05)	(0.10)	(0.07)	(0.02)	(0.01)	(0.09)	(0.00)	(0.23)
15. Dairy	0.13	-0.21*	-0.01	-0.20	-0.09	-0.05	-0.02	0.07*	0.06	-0.09	-0.21	-0.40*	-0.11	0.07	-0.81**	0.04	-0.00	-0.03	-0.00	1.32**
	(0.08)	(0.11)	(0.08)	(0.10)	(0.10)	(0.04)	(0.08)	(0.03)	(0.06)	(0.06)	(0.15)	(0.16)	(0.06)	(0.08)	(0.11)	(0.03)	(0.01)	(0.12)	(0.00)	(0.24)
16. Fats	0.15	0.15	-0.02	-0.16	-0.19	0.04	-0.19*	-0.02	0.23*	0.05	0.03	0.37*	-0.01	-0.00	0.11	-0.51**	0.03	0.08	-0.06**	0.96**
	(0.09)	(0.09)	(0.06)	(0.09)	(0.10)	(0.09)	(0.08)	(0.05)	(0.11)	(0.07)	(0.20)	(0.14)	(0.08)	(0.06)	(0.07)	(0.11)	(0.02)	(0.13)	(0.01)	(0.20)
17. Coffee	0.15	0.15	-0.12	0.04	-0.09	-0.39*	0.22*	-0.12*	-0.11	0.03	-0.23	0.35*	0.09	0.00	-0.03	0.13	-0.75**	0.32*	0.06	1.08**
	(0.12)	(0.11)	(0.07)	(0.10)	(0.13)	(0.17)	(0.10)	(0.06)	(0.21)	(0.08)	(0.26)	(0.17)	(0.11)	(0.07)	(0.08)	(0.11)	(0.09)	(0.16)	(0.03)	(0.25)
18. SoftDrink	0.10	0.09	-0.09	0.06	0.00	0.27	-0.27	0.07	0.90*	0.21	-0.13	-0.21	0.02	0.24	-0.08	0.05	0.05	-1.27	0.10**	1.69*
	(0.36)	(0.41)	(0.32)	(0.44)	(0.46)	(0.21)	(0.34)	(0.18)	(0.37)	(0.29)	(0.70)	(0.48)	(0.33)	(0.27)	(0.32)	(0.14)	(0.04)	(0.75)	(0.04)	(0.66)
19. OtherFood	-0.09	-0.01	-0.02	-0.18**	-0.00	-0.22**	-0.03	-0.00	-0.43**	0.10*	0.02	0.11	0.15**	0.08*	-0.02	-0.24**	0.06*	0.54**	-0.55**	0.54**
	(0.07)	(0.06)	(0.05)	(0.06)	(0.07)	(0.07)	(0.05)	(0.03)	(0.11)	(0.04)	(0.12)	(0.06)	(0.05)	(0.04)	(0.04)	(0.04)	(0.03)	(0.10)	(0.08)	(0.12)

Note: This table shows the sample-wide median elasticity of food demand (quantity consumed) with respect to food prices (columns 1 thru 19) and total household expenditures (the last column). For a list of items in each food group, see Suppl. Table 48.

Suppl. Table 11: Food demand elasticities with respect to food prices and total household expenditures for Q3 consumers (with per capita expenditures between $3.20 and $5.50 per day) in Uganda

	Food Group																			Exp.
	Rice	Maize	Wheat	Cassava	Roots	Sugar	Pulses	Nuts	Vegetables	Fruit	RedMeat	Poultry	Eggs	Fish	Dairy	Fats	Coffee	SoftDrink	OtherFood	
1. Rice	-0.71**	0.03	-0.07	0.06	0.12	0.11	-0.05	-0.18**	-0.24*	0.05	-0.02	0.20	-0.05	-0.15	0.02	0.04	0.02	-0.18	-0.01	1.34**
	(0.21)	(0.14)	(0.11)	(0.17)	(0.15)	(0.09)	(0.12)	(0.07)	(0.11)	(0.10)	(0.25)	(0.14)	(0.11)	(0.11)	(0.11)	(0.05)	(0.01)	(0.18)	(0.01)	(0.21)
2. Maize	0.03	-0.79**	-0.05	-0.11	0.26**	0.00	-0.02	-0.01	0.11*	0.05	0.12	-0.02	0.18**	0.11	-0.08	0.04*	0.01	0.16	0.01	0.67**
	(0.07)	(0.11)	(0.06)	(0.10)	(0.09)	(0.03)	(0.06)	(0.03)	(0.05)	(0.05)	(0.11)	(0.10)	(0.06)	(0.07)	(0.06)	(0.02)	(0.01)	(0.10)	(0.00)	(0.12)
3. Wheat	-0.05	-0.10	-0.80**	-0.01	-0.03	-0.05	0.02	0.04	-0.01	0.05	-0.15	0.00	-0.03	-0.10	-0.03	0.02	-0.00	0.02	-0.00	1.19**
	(0.08)	(0.09)	(0.10)	(0.12)	(0.09)	(0.04)	(0.07)	(0.03)	(0.05)	(0.06)	(0.12)	(0.11)	(0.06)	(0.08)	(0.07)	(0.02)	(0.01)	(0.10)	(0.00)	(0.14)
4. Cassava	0.05	-0.12	0.01	-0.34*	-0.03	-0.00	-0.01	-0.06	-0.03	0.09	0.04	-0.00	-0.04	0.05	-0.14	-0.04	-0.00	0.04	-0.01*	0.64**
	(0.09)	(0.11)	(0.09)	(0.17)	(0.11)	(0.04)	(0.07)	(0.03)	(0.06)	(0.06)	(0.12)	(0.11)	(0.07)	(0.08)	(0.07)	(0.02)	(0.01)	(0.12)	(0.00)	(0.16)
5. Roots	0.06	0.20**	-0.00	-0.03	-0.84**	0.03	0.07	-0.02	-0.09*	0.01	-0.10	-0.05	-0.01	0.12*	-0.02	-0.01	-0.00	-0.01	-0.00	0.75**
	(0.05)	(0.07)	(0.05)	(0.08)	(0.10)	(0.03)	(0.05)	(0.02)	(0.04)	(0.05)	(0.09)	(0.11)	(0.05)	(0.05)	(0.05)	(0.02)	(0.01)	(0.08)	(0.00)	(0.11)
6. Sugar	0.14	-0.01	-0.06	-0.02	0.08	-0.65**	-0.09	0.05	-0.27**	0.00	-0.37*	0.02	-0.06	-0.10	-0.16*	0.10	-0.06**	0.22	-0.04**	0.86**
	(0.10)	(0.08)	(0.06)	(0.09)	(0.09)	(0.10)	(0.07)	(0.05)	(0.10)	(0.06)	(0.16)	(0.08)	(0.07)	(0.06)	(0.06)	(0.05)	(0.02)	(0.11)	(0.01)	(0.13)
7. Pulses	-0.01	-0.02	0.04	-0.02	0.15	-0.04	-1.14**	-0.05	-0.04	0.14*	0.22	0.06	0.16**	-0.09	0.00	-0.11**	0.01	0.15	-0.01	0.53**
	(0.08)	(0.08)	(0.06)	(0.09)	(0.08)	(0.04)	(0.09)	(0.04)	(0.06)	(0.06)	(0.12)	(0.08)	(0.05)	(0.06)	(0.07)	(0.02)	(0.01)	(0.10)	(0.00)	(0.12)
8. Nuts	-0.17**	-0.04	0.07	-0.13*	-0.06	0.04	-0.09	-0.90**	0.13**	0.02	0.27**	0.00	0.09*	-0.04	0.12*	-0.06*	-0.01	0.03	0.00	0.89**
	(0.06)	(0.06)	(0.05)	(0.06)	(0.06)	(0.04)	(0.06)	(0.04)	(0.05)	(0.04)	(0.10)	(0.06)	(0.04)	(0.03)	(0.05)	(0.02)	(0.01)	(0.08)	(0.00)	(0.09)
9. Vegetables	-0.27*	0.23*	-0.01	-0.09	-0.26*	-0.26**	-0.09	0.14**	-1.71**	-0.09	0.66**	0.13	0.18*	0.08	0.10	0.18**	0.01	0.73**	-0.06**	0.87**
	(0.12)	(0.10)	(0.08)	(0.12)	(0.12)	(0.09)	(0.10)	(0.05)	(0.22)	(0.07)	(0.21)	(0.09)	(0.08)	(0.07)	(0.08)	(0.06)	(0.02)	(0.18)	(0.02)	(0.16)
10. Fruit	0.06	0.07	0.07	0.15	0.00	-0.01	0.16*	0.01	-0.09	-0.89**	-0.07	-0.07	0.04	0.07	-0.02	-0.00	0.00	0.07	0.02**	1.21**
	(0.09)	(0.11)	(0.09)	(0.10)	(0.14)	(0.05)	(0.08)	(0.04)	(0.07)	(0.11)	(0.13)	(0.13)	(0.08)	(0.08)	(0.09)	(0.03)	(0.01)	(0.12)	(0.01)	(0.15)
11. RedMeat	-0.01	0.09	-0.10	0.00	-0.19	-0.17*	0.12	0.12*	0.27**	-0.04	-1.42**	-0.06	-0.08	0.08	-0.08	-0.02	-0.01	-0.16	0.02*	1.38**
	(0.11)	(0.11)	(0.08)	(0.11)	(0.11)	(0.07)	(0.08)	(0.05)	(0.09)	(0.06)	(0.23)	(0.11)	(0.09)	(0.07)	(0.09)	(0.04)	(0.01)	(0.14)	(0.01)	(0.16)
12. Poultry	0.08	-0.11	-0.03	-0.06	-0.23	-0.03	-0.03	-0.04	0.02	-0.07	-0.12	-1.09**	-0.01	0.08	-0.11	0.02	0.00	-0.05	-0.00	2.47**
	(0.07)	(0.11)	(0.08)	(0.10)	(0.16)	(0.04)	(0.07)	(0.03)	(0.05)	(0.07)	(0.12)	(0.42)	(0.08)	(0.09)	(0.08)	(0.03)	(0.01)	(0.11)	(0.00)	(0.48)
13. Eggs	-0.14	0.87**	-0.11	-0.20	-0.10	-0.14	0.55*	0.21	0.37	0.08	-0.43	-0.02	-1.16**	0.13	-0.29	-0.02	0.01	-0.43	0.08**	1.31**
	(0.29)	(0.32)	(0.22)	(0.34)	(0.37)	(0.15)	(0.22)	(0.11)	(0.20)	(0.22)	(0.49)	(0.41)	(0.28)	(0.20)	(0.21)	(0.10)	(0.03)	(0.42)	(0.02)	(0.43)
14. Fish	-0.10	0.15	-0.09	0.05	0.20*	-0.07	-0.12	-0.03	0.05	0.06	0.13	0.17	0.04	-0.98**	-0.03	-0.02	-0.00	0.06	0.01	1.04**
	(0.08)	(0.10)	(0.08)	(0.11)	(0.10)	(0.04)	(0.06)	(0.03)	(0.05)	(0.06)	(0.11)	(0.13)	(0.06)	(0.11)	(0.07)	(0.02)	(0.01)	(0.09)	(0.00)	(0.19)
15. Dairy	0.02	-0.17	-0.03	-0.24*	-0.07	-0.12**	-0.02	0.09*	0.06	-0.02	-0.12	-0.12	-0.09	-0.04	-0.66**	-0.02	-0.01	-0.11	-0.00	1.24**
	(0.08)	(0.11)	(0.08)	(0.11)	(0.11)	(0.04)	(0.08)	(0.04)	(0.06)	(0.07)	(0.16)	(0.12)	(0.06)	(0.07)	(0.12)	(0.03)	(0.01)	(0.13)	(0.01)	(0.17)
16. Fats	0.10	0.20*	0.09	-0.17	-0.05	0.19	-0.34**	-0.11*	0.34**	0.02	-0.04	0.19	-0.01	-0.03	-0.04	-0.47**	0.06*	0.22	-0.08**	0.63**
	(0.10)	(0.09)	(0.07)	(0.09)	(0.10)	(0.10)	(0.09)	(0.05)	(0.12)	(0.08)	(0.21)	(0.12)	(0.08)	(0.07)	(0.08)	(0.11)	(0.03)	(0.14)	(0.01)	(0.18)
17. Coffee	0.23	0.09	0.00	-0.05	-0.00	-0.50**	0.08	-0.06	0.05	0.03	-0.13	0.09	0.06	-0.02	-0.14	0.25*	-0.71**	0.17	0.09*	0.77**
	(0.13)	(0.12)	(0.08)	(0.10)	(0.13)	(0.16)	(0.10)	(0.06)	(0.21)	(0.08)	(0.25)	(0.14)	(0.10)	(0.07)	(0.09)	(0.11)	(0.09)	(0.16)	(0.04)	(0.22)
18. SoftDrink	-0.28	0.44	0.03	0.10	-0.10	0.26	0.29	0.03	0.91*	0.09	-0.49	-0.12	-0.25	0.10	-0.20	0.14	0.02	-1.45*	0.09**	1.33*
	(0.36)	(0.43)	(0.31)	(0.46)	(0.47)	(0.21)	(0.32)	(0.17)	(0.37)	(0.28)	(0.65)	(0.47)	(0.33)	(0.27)	(0.33)	(0.13)	(0.04)	(0.70)	(0.03)	(0.59)
19. OtherFood	-0.06	0.11	-0.02	-0.20**	-0.04	-0.30**	-0.09	0.03	-0.52**	0.18**	0.38**	0.02	0.30**	0.10*	-0.00	-0.38**	0.09**	0.62**	-0.92**	0.78**
	(0.08)	(0.08)	(0.06)	(0.08)	(0.08)	(0.08)	(0.06)	(0.04)	(0.14)	(0.05)	(0.14)	(0.06)	(0.07)	(0.05)	(0.06)	(0.05)	(0.03)	(0.13)	(0.10)	(0.12)

Note: This table shows the sample-wide median elasticity of food demand (quantity consumed) with respect to food prices (columns 1 thru 19) and total household expenditures (the last column). For a list of items in each food group, see Suppl. Table 48.

Suppl. Table 12: Food demand elasticities with respect to food prices and total household expenditures for Q4 consumers (with per capita expenditures greater than $5.50 per day) in Uganda

	Food Group																			Exp.
	Rice	Maize	Wheat	Cassava	Roots	Sugar	Pulses	Nuts	Vegetables	Fruit	RedMeat	Poultry	Eggs	Fish	Dairy	Fats	Coffee	SoftDrink	OtherFood	
1. Rice	-0.09	-0.25	-0.09	-0.10	-0.01	0.20	-0.28	-0.09	-0.02	0.03	-0.07	-0.10	-0.05	-0.09	-0.12	0.01	0.03	-0.48*	0.00	0.80**
	(0.28)	(0.19)	(0.13)	(0.21)	(0.18)	(0.11)	(0.16)	(0.09)	(0.13)	(0.14)	(0.32)	(0.18)	(0.13)	(0.15)	(0.14)	(0.06)	(0.02)	(0.23)	(0.01)	(0.22)
2. Maize	-0.14	-0.74**	-0.06	-0.07	0.41**	-0.04	0.08	-0.05	0.11	0.03	0.19	-0.01	0.23**	0.11	-0.07	0.06*	0.00	0.35*	0.02**	0.65**
	(0.10)	(0.16)	(0.10)	(0.14)	(0.13)	(0.05)	(0.09)	(0.04)	(0.07)	(0.08)	(0.16)	(0.15)	(0.08)	(0.10)	(0.09)	(0.03)	(0.01)	(0.14)	(0.01)	(0.15)
3. Wheat	-0.09	-0.10	-0.76**	0.05	0.04	0.01	0.14	0.10*	-0.09	0.06	-0.44*	0.18	-0.00	-0.05	-0.07	0.07*	0.01	0.09	-0.00	1.29**
	(0.10)	(0.13)	(0.15)	(0.17)	(0.12)	(0.05)	(0.09)	(0.05)	(0.07)	(0.09)	(0.18)	(0.15)	(0.07)	(0.11)	(0.10)	(0.03)	(0.01)	(0.13)	(0.01)	(0.15)
4. Cassava	-0.06	-0.07	0.05	-0.20	0.01	-0.04	-0.07	-0.09	0.03	0.21*	0.22	0.05	-0.07	-0.10	-0.22	-0.05	-0.01	0.07	-0.01	0.60**
	(0.13)	(0.16)	(0.15)	(0.28)	(0.18)	(0.06)	(0.11)	(0.05)	(0.10)	(0.09)	(0.20)	(0.18)	(0.11)	(0.13)	(0.12)	(0.03)	(0.01)	(0.18)	(0.01)	(0.22)
5. Roots	0.00	0.34**	0.05	-0.01	-0.70**	0.01	0.20*	-0.02	-0.14*	0.03	-0.35*	-0.22	0.00	0.14	-0.01	0.02	0.00	-0.06	-0.00	0.66**
	(0.08)	(0.12)	(0.08)	(0.13)	(0.15)	(0.05)	(0.08)	(0.04)	(0.06)	(0.08)	(0.15)	(0.18)	(0.08)	(0.08)	(0.08)	(0.03)	(0.01)	(0.13)	(0.01)	(0.16)
6. Sugar	0.29	-0.09	0.05	-0.08	0.03	-1.02**	-0.32**	0.01	-0.16	0.00	-0.35	-0.08	-0.10	-0.10	-0.31**	0.23**	-0.09**	0.25	-0.04**	0.47*
	(0.15)	(0.12)	(0.09)	(0.13)	(0.13)	(0.16)	(0.12)	(0.08)	(0.13)	(0.10)	(0.23)	(0.12)	(0.09)	(0.09)	(0.10)	(0.07)	(0.03)	(0.17)	(0.01)	(0.18)
7. Pulses	-0.23	0.11	0.16	-0.09	0.36**	-0.20**	-1.16**	-0.15*	-0.22*	0.23*	0.56**	-0.04	0.25**	-0.27**	-0.01	-0.21**	-0.01	0.55**	-0.01	0.65**
	(0.13)	(0.13)	(0.10)	(0.14)	(0.13)	(0.07)	(0.14)	(0.06)	(0.10)	(0.09)	(0.19)	(0.14)	(0.08)	(0.09)	(0.11)	(0.04)	(0.01)	(0.15)	(0.01)	(0.15)
8. Nuts	-0.11	-0.11	0.15*	-0.17	-0.04	0.00	-0.20*	-0.81**	0.20**	0.11	0.39**	-0.13	0.08	-0.09	0.17*	-0.13**	0.00	-0.01	0.01	0.71**
	(0.10)	(0.08)	(0.07)	(0.09)	(0.09)	(0.06)	(0.08)	(0.06)	(0.06)	(0.07)	(0.14)	(0.08)	(0.06)	(0.05)	(0.07)	(0.04)	(0.01)	(0.11)	(0.01)	(0.11)
9. Vegetables	-0.03	0.25	-0.14	0.04	-0.40*	-0.18	-0.38*	0.23**	-1.92**	-0.12	0.93**	0.06	0.27*	0.37**	0.11	0.28**	0.03	0.93**	-0.07**	0.72**
	(0.16)	(0.15)	(0.11)	(0.18)	(0.17)	(0.12)	(0.15)	(0.08)	(0.29)	(0.11)	(0.30)	(0.13)	(0.11)	(0.13)	(0.12)	(0.08)	(0.03)	(0.26)	(0.02)	(0.20)
10. Fruit	0.03	0.03	0.08	0.31*	0.04	-0.01	0.27*	0.09	-0.10	-0.84**	-0.20	0.03	0.01	0.00	0.08	-0.03	0.00	-0.02	0.03**	1.06**
	(0.14)	(0.14)	(0.12)	(0.14)	(0.17)	(0.07)	(0.11)	(0.06)	(0.09)	(0.14)	(0.19)	(0.17)	(0.10)	(0.10)	(0.12)	(0.05)	(0.01)	(0.16)	(0.01)	(0.16)
11. RedMeat	-0.05	0.15	-0.27*	0.17	-0.42**	-0.15	0.34**	0.17**	0.35**	-0.10	-1.16**	0.24	-0.27*	0.02	-0.00	-0.04	-0.00	-0.34*	0.04**	1.10**
	(0.15)	(0.14)	(0.12)	(0.15)	(0.15)	(0.08)	(0.11)	(0.06)	(0.11)	(0.09)	(0.28)	(0.14)	(0.11)	(0.09)	(0.12)	(0.05)	(0.02)	(0.17)	(0.01)	(0.17)
12. Poultry	-0.11	-0.08	0.09	-0.00	-0.39*	-0.08	-0.09	-0.11**	-0.02	-0.02	0.15	0.41	-0.05	-0.13	0.10	-0.04	-0.02*	-0.02	-0.01*	2.62**
	(0.08)	(0.13)	(0.09)	(0.13)	(0.20)	(0.05)	(0.08)	(0.04)	(0.06)	(0.08)	(0.14)	(0.52)	(0.09)	(0.11)	(0.09)	(0.03)	(0.01)	(0.13)	(0.01)	(0.56)
13. Eggs	-0.14	0.95*	0.00	-0.28	0.00	-0.18	0.73*	0.16	0.49	0.03	-1.22*	-0.21	-1.13**	0.12	-0.19	-0.01	0.00	-1.00	0.11**	0.94*
	(0.34)	(0.40)	(0.26)	(0.43)	(0.45)	(0.18)	(0.29)	(0.14)	(0.25)	(0.25)	(0.62)	(0.53)	(0.32)	(0.24)	(0.26)	(0.11)	(0.03)	(0.51)	(0.03)	(0.46)
14. Fish	-0.08	0.15	-0.04	-0.14	0.24	-0.07	-0.26**	-0.06	0.23**	0.01	0.05	-0.14	0.04	-0.89**	-0.17	-0.03	-0.00	-0.03	0.01	0.75**
	(0.12)	(0.15)	(0.12)	(0.17)	(0.14)	(0.06)	(0.10)	(0.04)	(0.08)	(0.09)	(0.15)	(0.19)	(0.08)	(0.17)	(0.10)	(0.04)	(0.01)	(0.14)	(0.01)	(0.24)
15. Dairy	-0.11	-0.12	-0.07	-0.31*	-0.05	-0.21**	-0.03	0.11*	0.05	0.06	-0.01	0.23	-0.07	-0.18*	-0.44**	-0.10**	-0.03**	-0.20	0.00	1.19**
	(0.11)	(0.13)	(0.10)	(0.14)	(0.14)	(0.06)	(0.10)	(0.05)	(0.08)	(0.09)	(0.19)	(0.15)	(0.08)	(0.09)	(0.15)	(0.04)	(0.01)	(0.16)	(0.01)	(0.19)
16. Fats	0.03	0.26	0.25*	-0.20	0.13	0.43**	-0.60**	-0.27**	0.51**	-0.04	-0.15	-0.07	-0.01	-0.09	-0.27*	-0.41**	0.10**	0.46*	-0.12**	0.25
	(0.16)	(0.14)	(0.11)	(0.13)	(0.14)	(0.15)	(0.15)	(0.09)	(0.18)	(0.12)	(0.28)	(0.18)	(0.11)	(0.11)	(0.13)	(0.14)	(0.04)	(0.19)	(0.02)	(0.28)
17. Coffee	0.35	0.01	0.15	-0.19	0.11	-0.65**	-0.12	0.02	0.26	0.04	0.02	-0.28	0.02	-0.05	-0.28*	0.42**	-0.65**	-0.04	0.13**	0.39
	(0.21)	(0.17)	(0.13)	(0.15)	(0.17)	(0.21)	(0.14)	(0.09)	(0.26)	(0.13)	(0.33)	(0.20)	(0.13)	(0.12)	(0.14)	(0.14)	(0.11)	(0.23)	(0.05)	(0.30)
18. SoftDrink	-0.69	0.82	0.16	0.14	-0.20	0.25	0.90*	-0.02	0.90	-0.03	-0.88	-0.02	-0.57	-0.05	-0.33	0.25	-0.01	-1.67*	0.08	1.09*
	(0.47)	(0.55)	(0.36)	(0.54)	(0.53)	(0.25)	(0.44)	(0.20)	(0.47)	(0.31)	(0.72)	(0.57)	(0.40)	(0.33)	(0.41)	(0.16)	(0.04)	(0.77)	(0.04)	(0.55)
19. OtherFood	0.01	0.33**	-0.01	-0.19	-0.10	-0.41**	-0.15	0.09	-0.63**	0.32**	1.00**	-0.15	0.55**	0.14	0.03	-0.58**	0.15**	0.68**	-1.59**	0.89**
	(0.12)	(0.12)	(0.09)	(0.12)	(0.13)	(0.11)	(0.10)	(0.06)	(0.18)	(0.09)	(0.24)	(0.10)	(0.11)	(0.07)	(0.10)	(0.08)	(0.04)	(0.18)	(0.15)	(0.15)

Note: This table shows the sample-wide median elasticity of food demand (quantity consumed) with respect to food prices (columns 1 thru 19) and total household expenditures (the last column). For a list of items in each food group, see Suppl. Table 48.

Suppl. Table 13: Food demand elasticities with respect to food prices and total household expenditures for Q1 consumers (with per capita expenditures less than $1.90 per day) in Tanzania

	Food Group																			Exp.
	Rice	Maize	Wheat	Cassava	Roots	Sugar	Pulses	Nuts	Vegetables	Fruit	RedMeat	Poultry	Eggs	Fish	Dairy	Oils	Coffee	SoftDrink	OtherFood	
1. Rice	-1.58	0.01	-0.29	0.47	-0.55	-0.47	0.09	0.05	-0.35	0.09	0.55	0.38	-0.04	-0.54	-0.62	0.10	0.14	0.25	-0.08	2.21
	(0.87)	(0.58)	(0.35)	(0.56)	(0.43)	(0.29)	(0.31)	(0.42)	(0.33)	(0.25)	(0.60)	(0.68)	(0.18)	(0.35)	(0.54)	(0.10)	(0.09)	(0.20)	(0.07)	(1.55)
2. Maize	0.08	-0.40*	-0.00	-0.05	-0.09	0.02	-0.03	-0.18*	-0.05	0.13*	-0.09	-0.28*	-0.03	0.08	0.24*	-0.04*	0.00	-0.05	-0.01	0.75**
	(0.13)	(0.18)	(0.06)	(0.10)	(0.07)	(0.03)	(0.06)	(0.08)	(0.05)	(0.06)	(0.12)	(0.13)	(0.04)	(0.05)	(0.11)	(0.02)	(0.01)	(0.03)	(0.01)	(0.19)
3. Wheat	-0.34	-0.03	-1.66**	-0.07	0.49*	-0.05	0.21	0.33	-0.14	0.21	-0.57	0.14	0.08	0.35*	-0.50	0.17*	-0.02	-0.17	0.03	0.99*
	(0.35)	(0.23)	(0.32)	(0.24)	(0.20)	(0.14)	(0.23)	(0.20)	(0.13)	(0.20)	(0.32)	(0.27)	(0.09)	(0.16)	(0.26)	(0.07)	(0.05)	(0.12)	(0.04)	(0.47)
4. Cassava	0.44	-0.16	-0.04	-1.03**	-0.08	0.11	0.03	0.22	-0.12	-0.14	0.58	0.35	0.05	0.19	0.01	0.11*	0.02	-0.07	0.03	1.06
	(0.38)	(0.27)	(0.15)	(0.32)	(0.16)	(0.08)	(0.14)	(0.25)	(0.13)	(0.12)	(0.34)	(0.36)	(0.10)	(0.13)	(0.25)	(0.05)	(0.03)	(0.08)	(0.03)	(0.65)
5. Roots	-0.52	-0.52*	0.33*	-0.14	-1.14**	0.23*	-0.73**	0.06	-0.14	-0.06	0.15	-0.37	0.11	-0.14	0.23	0.07	0.01	0.15	-0.10**	1.97**
	(0.32)	(0.22)	(0.16)	(0.19)	(0.20)	(0.09)	(0.21)	(0.15)	(0.11)	(0.12)	(0.27)	(0.22)	(0.07)	(0.12)	(0.19)	(0.04)	(0.03)	(0.09)	(0.03)	(0.52)
6. Sugar	-1.03**	0.07	-0.10	0.30	0.60**	-0.14	1.16**	-0.02	0.30	-0.51**	-1.08**	-0.44	0.21*	0.10	-0.32	0.03	-0.25**	0.17	0.04	1.35*
	(0.38)	(0.22)	(0.25)	(0.21)	(0.20)	(0.31)	(0.33)	(0.17)	(0.18)	(0.18)	(0.36)	(0.24)	(0.08)	(0.18)	(0.18)	(0.11)	(0.09)	(0.11)	(0.05)	(0.53)
7. Pulses	0.14	-0.21	0.14	0.03	-0.69**	0.48**	-1.02**	-0.21	-0.27*	-0.02	0.04	-0.02	-0.01	-0.13	0.35*	-0.33**	0.06	0.00	0.14**	1.37**
	(0.25)	(0.18)	(0.18)	(0.17)	(0.18)	(0.15)	(0.25)	(0.14)	(0.13)	(0.13)	(0.24)	(0.17)	(0.05)	(0.10)	(0.17)	(0.10)	(0.05)	(0.07)	(0.04)	(0.31)
8. Nuts	0.10	-0.75	0.32	0.37	0.12	-0.00	-0.26	-0.77	-0.24	0.13	-0.21	0.13	-0.11	-0.05	-0.66	-0.12	0.04	0.16	0.08	0.77
	(0.49)	(0.48)	(0.23)	(0.45)	(0.21)	(0.10)	(0.22)	(0.40)	(0.20)	(0.16)	(0.46)	(0.58)	(0.17)	(0.16)	(0.41)	(0.08)	(0.04)	(0.13)	(0.04)	(0.96)
9. Vegetables	-0.12	-0.08	-0.06	-0.08	-0.02	0.10	-0.13	-0.13	-0.38*	-0.20**	0.13	-0.11	0.04	0.31**	0.09	-0.11**	0.04	0.03	-0.01	0.58**
	(0.13)	(0.11)	(0.07)	(0.10)	(0.07)	(0.06)	(0.09)	(0.07)	(0.16)	(0.07)	(0.14)	(0.10)	(0.03)	(0.10)	(0.08)	(0.04)	(0.02)	(0.04)	(0.01)	(0.20)
10. Fruit	0.12	0.28	0.22	-0.35	-0.13	-0.40**	-0.14	0.12	-0.70**	-1.05**	-0.21	0.41	0.08	-0.54**	0.04	-0.04	-0.06	-0.11	-0.03	2.86**
	(0.38)	(0.30)	(0.27)	(0.26)	(0.22)	(0.14)	(0.22)	(0.22)	(0.18)	(0.24)	(0.39)	(0.30)	(0.10)	(0.19)	(0.27)	(0.07)	(0.04)	(0.12)	(0.04)	(0.62)
11. RedMeat	0.52	-0.81	-0.51	0.58	0.11	-0.53*	-0.08	-0.22	-0.01	-0.15	-1.91**	0.44	-0.17	-0.04	0.27	-0.09	-0.01	-0.28	-0.02	3.38**
	(0.58)	(0.58)	(0.32)	(0.45)	(0.32)	(0.23)	(0.28)	(0.38)	(0.25)	(0.26)	(0.73)	(0.49)	(0.18)	(0.19)	(0.40)	(0.09)	(0.05)	(0.19)	(0.05)	(1.26)
12. Poultry	0.21	-1.80**	0.03	0.15	-0.49*	-0.24	-0.28	0.01	-0.62**	0.15	0.30	-1.74	0.09	-0.17	0.44	-0.18	-0.00	0.08	-0.06	6.11**
	(0.67)	(0.51)	(0.24)	(0.48)	(0.24)	(0.15)	(0.20)	(0.46)	(0.23)	(0.17)	(0.43)	(0.92)	(0.18)	(0.21)	(0.42)	(0.09)	(0.03)	(0.11)	(0.05)	(2.35)
13. Eggs	-0.14	-0.57	0.19	0.21	0.39	0.31*	-0.07	-0.35	0.13	0.16	-0.58	0.39	-1.20**	-0.03	-0.67	-0.03	0.06	0.12	-0.02	1.77
	(0.55)	(0.59)	(0.26)	(0.49)	(0.27)	(0.15)	(0.22)	(0.43)	(0.24)	(0.20)	(0.54)	(0.68)	(0.28)	(0.18)	(0.44)	(0.07)	(0.04)	(0.13)	(0.04)	(1.70)
14. Fish	-0.64*	0.32	0.36*	0.32	-0.14	0.06	-0.15	-0.05	0.59**	-0.38**	0.03	-0.05	-0.00	-1.06**	-0.19	0.07	0.04	0.04	-0.03	0.79*
	(0.29)	(0.19)	(0.16)	(0.18)	(0.15)	(0.10)	(0.13)	(0.15)	(0.15)	(0.15)	(0.22)	(0.22)	(0.06)	(0.17)	(0.15)	(0.05)	(0.04)	(0.08)	(0.03)	(0.37)
15. Dairy	-0.88	0.90	-0.63	-0.05	0.33	-0.23	0.49	-0.84	0.09	0.04	0.40	0.90	-0.28	-0.26	-1.05	0.20*	-0.01	-0.04	-0.09	2.19
	(0.76)	(0.54)	(0.40)	(0.55)	(0.35)	(0.16)	(0.35)	(0.49)	(0.24)	(0.27)	(0.60)	(0.79)	(0.22)	(0.23)	(0.84)	(0.10)	(0.06)	(0.16)	(0.06)	(1.30)
16. Fats	0.29	-0.40**	0.30*	0.32**	0.23*	0.04	-0.78**	-0.24*	-0.44**	-0.00	-0.10	-0.21	-0.01	0.12	0.35**	-0.62**	-0.00	0.07	0.09	1.19**
	(0.17)	(0.14)	(0.14)	(0.12)	(0.10)	(0.12)	(0.22)	(0.11)	(0.12)	(0.09)	(0.17)	(0.13)	(0.04)	(0.09)	(0.11)	(0.20)	(0.06)	(0.05)	(0.06)	(0.25)
17. Coffee	1.30*	-0.15	-0.13	0.15	0.05	-1.05**	0.58	0.29	0.42	-0.36	-0.11	0.04	0.16	0.28	-0.04	-0.04	-0.78**	0.21	0.04	2.18**
	(0.53)	(0.38)	(0.38)	(0.33)	(0.30)	(0.36)	(0.45)	(0.27)	(0.28)	(0.23)	(0.43)	(0.31)	(0.09)	(0.28)	(0.31)	(0.25)	(0.18)	(0.16)	(0.10)	(0.66)
18. SoftDrink	0.94	-0.78	-0.52	-0.37	0.62	0.29	-0.02	0.48	0.10	-0.27	-1.05	0.43	0.13	0.10	-0.10	0.10	0.08	-0.82**	-0.03	1.88
	(0.78)	(0.56)	(0.47)	(0.50)	(0.44)	(0.24)	(0.35)	(0.44)	(0.27)	(0.36)	(0.79)	(0.53)	(0.17)	(0.31)	(0.46)	(0.11)	(0.08)	(0.31)	(0.06)	(0.96)
19. OtherFood	-0.35	-0.09	0.15	0.29	-0.47**	0.13	0.90**	0.32*	0.00	-0.03	0.05	-0.05	-0.01	-0.10	-0.27	0.24	0.04	-0.02	-0.51**	0.00
	(0.26)	(0.17)	(0.16)	(0.18)	(0.18)	(0.12)	(0.30)	(0.14)	(0.11)	(0.12)	(0.21)	(0.16)	(0.05)	(0.13)	(0.16)	(0.15)	(0.05)	(0.06)	(0.15)	(0.40)

Note: This table shows the sample-wide median elasticity of food demand (quantity consumed) with respect to food prices (columns 1 thru 19) and total household expenditures (the last column). For a list of items in each food group, see Suppl. Table 49.

Suppl. Table 14: Food demand elasticities with respect to food prices and total household expenditures for Q2 consumers (with per capita expenditures between $1.90 and $3.20 per day) in Tanzania

	Food Group																			Exp.
	Rice	Maize	Wheat	Cassava	Roots	Sugar	Pulses	Nuts	Vegetables	Fruit	RedMeat	Poultry	Eggs	Fish	Dairy	Oils	Coffee	SoftDrink	OtherFood	
1. Rice	-0.88	0.05	-0.09	0.23	-0.11	-0.23	-0.06	0.02	-0.22	0.13	0.12	0.22	-0.06	-0.26	-0.47	-0.01	0.09	0.12	-0.02	1.20
	(0.62)	(0.43)	(0.23)	(0.38)	(0.25)	(0.18)	(0.21)	(0.33)	(0.24)	(0.18)	(0.47)	(0.54)	(0.18)	(0.22)	(0.44)	(0.06)	(0.07)	(0.14)	(0.04)	(0.87)
2. Maize	0.05	-0.38*	-0.01	-0.11	-0.10	-0.00	0.00	-0.10	-0.05	0.09	0.04	-0.25	-0.03	0.08	0.21*	-0.01	0.00	-0.01	-0.01	0.68**
	(0.14)	(0.19)	(0.05)	(0.09)	(0.06)	(0.03)	(0.04)	(0.08)	(0.05)	(0.05)	(0.12)	(0.14)	(0.04)	(0.05)	(0.10)	(0.01)	(0.01)	(0.03)	(0.01)	(0.20)
3. Wheat	-0.14	-0.13	-0.86**	-0.16	0.27	-0.16	0.18	0.18	-0.24*	0.10	-0.28	0.14	0.06	0.10	-0.26	0.12*	-0.01	-0.10	0.01	1.39**
	(0.27)	(0.18)	(0.25)	(0.18)	(0.15)	(0.10)	(0.19)	(0.15)	(0.11)	(0.15)	(0.26)	(0.22)	(0.08)	(0.12)	(0.20)	(0.06)	(0.04)	(0.08)	(0.03)	(0.32)
4. Cassava	0.28	-0.28	-0.11	-0.68*	-0.23	-0.01	0.06	0.08	-0.10	-0.04	0.52	0.12	0.07	0.08	0.19	0.04	-0.01	-0.05	0.01	0.87*
	(0.30)	(0.22)	(0.13)	(0.28)	(0.14)	(0.06)	(0.11)	(0.18)	(0.09)	(0.09)	(0.29)	(0.28)	(0.10)	(0.09)	(0.20)	(0.03)	(0.02)	(0.06)	(0.02)	(0.44)
5. Roots	-0.15	-0.38**	0.20	-0.24	-0.81**	0.13*	-0.48**	0.01	-0.10	-0.07	0.09	-0.21	0.11	-0.06	0.20	0.04	0.01	0.07	-0.04	1.53**
	(0.21)	(0.15)	(0.11)	(0.13)	(0.15)	(0.06)	(0.14)	(0.11)	(0.07)	(0.08)	(0.19)	(0.15)	(0.06)	(0.08)	(0.13)	(0.03)	(0.02)	(0.05)	(0.02)	(0.29)
6. Sugar	-0.53*	-0.09	-0.24	-0.04	0.30*	0.01	0.75**	-0.06	0.15	-0.03	-0.65**	-0.17	0.15*	0.00	-0.17	0.05	-0.19**	0.07	-0.02	1.17**
	(0.23)	(0.16)	(0.16)	(0.13)	(0.13)	(0.25)	(0.22)	(0.10)	(0.12)	(0.10)	(0.25)	(0.14)	(0.06)	(0.12)	(0.12)	(0.08)	(0.06)	(0.07)	(0.04)	(0.26)
7. Pulses	-0.07	-0.04	0.17	0.05	-0.53**	0.42**	-0.43	-0.07	-0.16	-0.07	-0.20	0.04	-0.02	-0.16	0.12	-0.22**	0.07	-0.01	0.14**	0.99**
	(0.20)	(0.12)	(0.15)	(0.13)	(0.14)	(0.12)	(0.23)	(0.10)	(0.10)	(0.10)	(0.19)	(0.13)	(0.05)	(0.09)	(0.13)	(0.08)	(0.04)	(0.05)	(0.04)	(0.22)
8. Nuts	0.03	-0.39	0.21	0.11	0.04	-0.04	-0.10	-0.78*	-0.19	0.05	-0.07	0.23	-0.13	-0.12	-0.33	-0.08	0.03	0.07	0.04	1.07
	(0.51)	(0.42)	(0.21)	(0.36)	(0.21)	(0.09)	(0.17)	(0.39)	(0.18)	(0.14)	(0.49)	(0.70)	(0.20)	(0.15)	(0.35)	(0.06)	(0.03)	(0.12)	(0.03)	(0.85)
9. Vegetables	-0.19	-0.12	-0.14	-0.10	-0.05	0.08	-0.11	-0.11	-0.45**	-0.17*	0.13	-0.10	0.04	0.24**	0.14	-0.12**	0.02	0.03	-0.01	0.71**
	(0.15)	(0.11)	(0.08)	(0.09)	(0.07)	(0.05)	(0.08)	(0.08)	(0.16)	(0.07)	(0.14)	(0.11)	(0.04)	(0.09)	(0.09)	(0.04)	(0.02)	(0.04)	(0.01)	(0.19)
10. Fruit	0.20	0.19	0.11	-0.09	-0.12	-0.03	-0.13	0.04	-0.32**	-0.81**	-0.36	0.22	0.04	-0.23*	-0.11	-0.01	-0.05	-0.02	0.00	1.53**
	(0.22)	(0.16)	(0.18)	(0.14)	(0.12)	(0.07)	(0.14)	(0.11)	(0.09)	(0.15)	(0.23)	(0.16)	(0.07)	(0.10)	(0.15)	(0.04)	(0.03)	(0.07)	(0.02)	(0.27)
11. RedMeat	0.09	-0.09	-0.22	0.42	0.07	-0.31*	-0.22	-0.06	0.05	-0.23	-1.37**	0.29	-0.16	-0.01	-0.14	0.01	-0.01	-0.22	0.00	1.97**
	(0.44)	(0.33)	(0.22)	(0.31)	(0.23)	(0.16)	(0.21)	(0.29)	(0.16)	(0.18)	(0.46)	(0.40)	(0.16)	(0.13)	(0.26)	(0.06)	(0.04)	(0.14)	(0.03)	(0.66)
12. Poultry	0.08	-1.00**	0.03	0.00	-0.29*	-0.14	-0.11	0.09	-0.29*	0.06	0.17	-1.25*	0.09	-0.10	0.26	-0.12*	-0.02	0.07	-0.03	3.85**
	(0.43)	(0.32)	(0.14)	(0.26)	(0.14)	(0.08)	(0.11)	(0.33)	(0.12)	(0.09)	(0.32)	(0.59)	(0.13)	(0.12)	(0.26)	(0.05)	(0.02)	(0.07)	(0.02)	(1.18)
13. Eggs	-0.27	-0.42	0.13	0.21	0.34	0.22	-0.13	-0.32	0.08	0.08	-0.66	0.38	-1.21**	-0.09	-0.60	-0.05	0.04	0.07	-0.02	2.13**
	(0.54)	(0.43)	(0.22)	(0.40)	(0.23)	(0.13)	(0.18)	(0.39)	(0.16)	(0.17)	(0.52)	(0.59)	(0.25)	(0.15)	(0.38)	(0.05)	(0.03)	(0.11)	(0.03)	(0.82)
14. Fish	-0.38	0.25	0.12	0.11	-0.05	0.01	-0.18	-0.10	0.35**	-0.19	0.02	-0.02	-0.02	-0.88**	-0.18	-0.03	0.02	0.05	-0.02	0.90**
	(0.20)	(0.15)	(0.12)	(0.12)	(0.11)	(0.08)	(0.11)	(0.11)	(0.11)	(0.10)	(0.17)	(0.17)	(0.06)	(0.14)	(0.12)	(0.04)	(0.03)	(0.06)	(0.03)	(0.24)
15. Dairy	-0.82	0.64	-0.32	0.26	0.30	-0.14	0.13	-0.36	0.16	-0.12	-0.21	0.58	-0.26	-0.22	-1.23*	0.11	0.03	-0.02	-0.04	1.70*
	(0.55)	(0.39)	(0.26)	(0.34)	(0.24)	(0.10)	(0.21)	(0.31)	(0.16)	(0.17)	(0.41)	(0.52)	(0.18)	(0.16)	(0.56)	(0.06)	(0.04)	(0.11)	(0.04)	(0.80)
16. Fats	-0.03	-0.12	0.25*	0.11	0.14*	0.06	-0.46**	-0.13*	-0.33**	0.01	0.09	-0.14	-0.02	-0.06	0.21**	-0.23	0.06	0.03	-0.00	0.95**
	(0.13)	(0.09)	(0.10)	(0.07)	(0.07)	(0.09)	(0.17)	(0.07)	(0.09)	(0.07)	(0.13)	(0.09)	(0.04)	(0.07)	(0.07)	(0.20)	(0.04)	(0.04)	(0.05)	(0.16)
17. Coffee	0.85*	-0.08	-0.04	-0.13	0.08	-0.80**	0.54	0.19	0.17	-0.25	-0.07	-0.06	0.10	0.12	0.16	0.17	-0.62**	0.15	-0.07	1.40**
	(0.34)	(0.22)	(0.25)	(0.20)	(0.17)	(0.23)	(0.31)	(0.15)	(0.18)	(0.14)	(0.28)	(0.20)	(0.07)	(0.18)	(0.20)	(0.16)	(0.12)	(0.10)	(0.06)	(0.36)
18. SoftDrink	0.52	-0.18	-0.32	-0.22	0.31	0.14	-0.05	0.20	0.12	-0.07	-0.96	0.38	0.09	0.13	-0.05	0.05	0.07	-0.75**	-0.01	1.34*
	(0.59)	(0.39)	(0.33)	(0.32)	(0.30)	(0.17)	(0.27)	(0.35)	(0.20)	(0.23)	(0.65)	(0.44)	(0.16)	(0.25)	(0.34)	(0.08)	(0.06)	(0.24)	(0.04)	(0.68)
19. OtherFood	-0.11	-0.17	0.11	0.08	-0.26	-0.07	1.05**	0.23	-0.02	0.06	0.12	-0.04	-0.03	-0.12	-0.16	0.00	-0.06	-0.01	-0.72**	0.22
	(0.27)	(0.18)	(0.20)	(0.17)	(0.16)	(0.15)	(0.38)	(0.14)	(0.13)	(0.14)	(0.28)	(0.19)	(0.06)	(0.16)	(0.18)	(0.16)	(0.07)	(0.07)	(0.14)	(0.38)

Note: This table shows the sample-wide median elasticity of food demand (quantity consumed) with respect to food prices (columns 1 thru 19) and total household expenditures (the last column). For a list of items in each food group, see Suppl. Table 49.

Suppl. Table 15: Food demand elasticities with respect to food prices and total household expenditures for Q3 consumers (with per capita expenditures between $3.20 and $5.50 per day) in Tanzania

	Food Group																			Exp.
	Rice	Maize	Wheat	Cassava	Roots	Sugar	Pulses	Nuts	Vegetables	Fruit	RedMeat	Poultry	Eggs	Fish	Dairy	Oils	Coffee	SoftDrink	OtherFood	
1. Rice	-0.47	-0.02	0.01	0.07	0.11	-0.10	-0.20	-0.01	-0.20	0.15	-0.12	0.14	-0.07	-0.11	-0.41	-0.09	0.06	0.04	0.01	1.15
	(0.80)	(0.43)	(0.22)	(0.38)	(0.26)	(0.14)	(0.23)	(0.38)	(0.25)	(0.20)	(0.55)	(0.70)	(0.21)	(0.18)	(0.47)	(0.09)	(0.06)	(0.13)	(0.04)	(0.74)
2. Maize	0.03	-0.32	-0.02	-0.20	-0.12	-0.04	0.05	-0.00	-0.05	0.04	0.21	-0.23	-0.02	0.09	0.18	0.02	-0.00	0.03	-0.01	0.51*
	(0.21)	(0.23)	(0.07)	(0.12)	(0.09)	(0.04)	(0.05)	(0.12)	(0.06)	(0.05)	(0.19)	(0.20)	(0.05)	(0.07)	(0.13)	(0.02)	(0.01)	(0.04)	(0.01)	(0.26)
3. Wheat	-0.00	-0.13	-0.28	-0.20	0.14	-0.22*	0.17	0.09	-0.27*	0.03	-0.09	0.14	0.05	-0.07	-0.10	0.10	0.00	-0.05	0.00	1.37**
	(0.24)	(0.17)	(0.28)	(0.16)	(0.14)	(0.09)	(0.17)	(0.14)	(0.12)	(0.13)	(0.24)	(0.22)	(0.07)	(0.11)	(0.18)	(0.06)	(0.03)	(0.07)	(0.03)	(0.27)
4. Cassava	0.13	-0.42	-0.17	-0.27	-0.38*	-0.15	0.09	-0.06	-0.08	0.06	0.49	-0.09	0.09	-0.02	0.42	-0.02	-0.05	-0.03	-0.02	0.58
	(0.38)	(0.26)	(0.15)	(0.37)	(0.19)	(0.08)	(0.12)	(0.22)	(0.10)	(0.10)	(0.33)	(0.35)	(0.13)	(0.09)	(0.28)	(0.03)	(0.03)	(0.07)	(0.02)	(0.50)
5. Roots	0.12	-0.29*	0.12	-0.33*	-0.54**	0.06	-0.31*	-0.02	-0.09	-0.08	0.07	-0.10	0.11	0.00	0.20	0.02	0.01	0.02	0.00	1.14**
	(0.21)	(0.14)	(0.10)	(0.14)	(0.18)	(0.05)	(0.12)	(0.12)	(0.07)	(0.07)	(0.19)	(0.17)	(0.06)	(0.08)	(0.13)	(0.03)	(0.02)	(0.05)	(0.02)	(0.25)
6. Sugar	-0.25	-0.21	-0.35*	-0.28*	0.14	0.16	0.57**	-0.09	0.06	0.29**	-0.43*	-0.03	0.12*	-0.06	-0.09	0.06	-0.17**	0.02	-0.07	1.04**
	(0.19)	(0.14)	(0.14)	(0.11)	(0.10)	(0.25)	(0.19)	(0.08)	(0.10)	(0.10)	(0.20)	(0.12)	(0.05)	(0.11)	(0.11)	(0.08)	(0.05)	(0.06)	(0.04)	(0.21)
7. Pulses	-0.28	0.09	0.21	0.08	-0.40**	0.38**	0.20	0.05	-0.07	-0.11	-0.44*	0.10	-0.04	-0.19	-0.09	-0.12	0.09	-0.02	0.14**	0.74**
	(0.21)	(0.11)	(0.17)	(0.12)	(0.14)	(0.12)	(0.30)	(0.10)	(0.10)	(0.10)	(0.21)	(0.15)	(0.05)	(0.10)	(0.14)	(0.08)	(0.04)	(0.06)	(0.04)	(0.23)
8. Nuts	-0.05	-0.08	0.10	-0.10	-0.05	-0.09	0.05	-0.79	-0.14	-0.03	0.04	0.30	-0.14	-0.18	-0.04	-0.05	0.02	-0.00	0.01	1.44
	(0.63)	(0.43)	(0.22)	(0.36)	(0.25)	(0.09)	(0.16)	(0.48)	(0.19)	(0.14)	(0.57)	(0.93)	(0.23)	(0.17)	(0.38)	(0.05)	(0.03)	(0.14)	(0.03)	(0.81)
9. Vegetables	-0.24	-0.12	-0.21	-0.09	-0.07	0.05	-0.06	-0.07	-0.54**	-0.10	0.12	-0.07	0.05	0.16	0.18	-0.12*	0.00	0.03	-0.00	0.60*
	(0.20)	(0.13)	(0.11)	(0.10)	(0.09)	(0.06)	(0.10)	(0.11)	(0.17)	(0.07)	(0.17)	(0.17)	(0.05)	(0.09)	(0.12)	(0.06)	(0.03)	(0.05)	(0.02)	(0.23)
10. Fruit	0.27	0.05	0.04	0.05	-0.12	0.20**	-0.14	-0.02	-0.15	-0.64**	-0.46*	0.11	0.03	-0.05	-0.23	0.01	-0.04	0.03	0.02	1.11**
	(0.21)	(0.14)	(0.15)	(0.12)	(0.11)	(0.07)	(0.12)	(0.10)	(0.08)	(0.15)	(0.20)	(0.13)	(0.06)	(0.09)	(0.13)	(0.04)	(0.02)	(0.05)	(0.02)	(0.22)
11. RedMeat	-0.16	0.20	-0.07	0.29	0.03	-0.20	-0.33	0.02	0.03	-0.27	-1.07**	0.20	-0.16	-0.01	-0.38	0.06	-0.01	-0.18	0.01	1.69**
	(0.45)	(0.29)	(0.19)	(0.26)	(0.20)	(0.12)	(0.22)	(0.28)	(0.14)	(0.17)	(0.40)	(0.45)	(0.16)	(0.12)	(0.28)	(0.06)	(0.03)	(0.13)	(0.03)	(0.52)
12. Poultry	0.04	-0.55	0.04	-0.12	-0.15	-0.05	-0.01	0.13	-0.15	0.02	0.12	-0.92	0.09	-0.04	0.16	-0.07	-0.02	0.06	-0.01	2.53**
	(0.51)	(0.29)	(0.14)	(0.24)	(0.15)	(0.06)	(0.10)	(0.38)	(0.12)	(0.07)	(0.37)	(0.66)	(0.14)	(0.11)	(0.26)	(0.04)	(0.02)	(0.07)	(0.02)	(0.82)
13. Eggs	-0.36	-0.29	0.08	0.21	0.31	0.15	-0.15	-0.29	0.06	0.02	-0.71	0.37	-1.22**	-0.13	-0.55	-0.06	0.02	0.04	-0.02	2.34**
	(0.59)	(0.40)	(0.21)	(0.37)	(0.22)	(0.11)	(0.17)	(0.39)	(0.15)	(0.15)	(0.53)	(0.61)	(0.24)	(0.14)	(0.38)	(0.05)	(0.03)	(0.10)	(0.03)	(0.71)
14. Fish	-0.19	0.21	-0.06	-0.04	0.01	-0.04	-0.21	-0.15	0.18*	-0.04	0.02	0.01	-0.04	-0.71**	-0.18	-0.11*	0.01	0.06	-0.02	0.96**
	(0.19)	(0.15)	(0.12)	(0.11)	(0.11)	(0.08)	(0.12)	(0.11)	(0.09)	(0.09)	(0.17)	(0.18)	(0.06)	(0.15)	(0.12)	(0.05)	(0.03)	(0.06)	(0.02)	(0.23)
15. Dairy	-0.78	0.37	-0.12	0.48	0.26	-0.08	-0.15	-0.05	0.17	-0.25	-0.68	0.34	-0.24	-0.21	-1.37**	0.04	0.05	-0.00	-0.00	1.76*
	(0.56)	(0.32)	(0.23)	(0.35)	(0.22)	(0.09)	(0.20)	(0.31)	(0.15)	(0.17)	(0.43)	(0.51)	(0.17)	(0.15)	(0.52)	(0.05)	(0.04)	(0.10)	(0.03)	(0.68)
16. Fats	-0.28*	0.08	0.23*	-0.06	0.08	0.09	-0.25	-0.06	-0.27**	0.02	0.25	-0.10	-0.02	-0.20**	0.12	0.12	0.11*	0.00	-0.08	0.80**
	(0.13)	(0.08)	(0.10)	(0.06)	(0.07)	(0.10)	(0.17)	(0.06)	(0.09)	(0.07)	(0.14)	(0.08)	(0.03)	(0.08)	(0.07)	(0.24)	(0.04)	(0.03)	(0.05)	(0.15)
17. Coffee	0.61*	-0.08	0.02	-0.32*	0.10	-0.66**	0.50	0.12	-0.00	-0.19	-0.05	-0.12	0.06	0.03	0.30	0.31*	-0.52**	0.13	-0.14*	1.05**
	(0.28)	(0.17)	(0.21)	(0.16)	(0.14)	(0.19)	(0.27)	(0.11)	(0.16)	(0.12)	(0.25)	(0.19)	(0.06)	(0.14)	(0.17)	(0.14)	(0.12)	(0.08)	(0.06)	(0.28)
18. SoftDrink	0.23	0.21	-0.19	-0.12	0.07	0.04	-0.09	-0.01	0.11	0.07	-0.95	0.36	0.06	0.16	-0.01	-0.00	0.07	-0.68**	0.00	1.23*
	(0.60)	(0.40)	(0.30)	(0.31)	(0.28)	(0.16)	(0.26)	(0.38)	(0.20)	(0.21)	(0.66)	(0.53)	(0.17)	(0.26)	(0.34)	(0.08)	(0.06)	(0.25)	(0.04)	(0.62)
19. OtherFood	0.21	-0.20	0.04	-0.18	0.06	-0.33	1.09*	0.09	-0.02	0.16	0.19	-0.02	-0.04	-0.14	0.02	-0.31	-0.18*	0.02	-1.02**	0.39
	(0.32)	(0.21)	(0.25)	(0.19)	(0.20)	(0.22)	(0.45)	(0.14)	(0.18)	(0.17)	(0.36)	(0.25)	(0.08)	(0.18)	(0.22)	(0.22)	(0.09)	(0.08)	(0.13)	(0.41)

Note: This table shows the sample-wide median elasticity of food demand (quantity consumed) with respect to food prices (columns 1 thru 19) and total household expenditures (the last column). For a list of items in each food group, see Suppl. Table 49.

Suppl. Table 16: Food demand elasticities with respect to food prices and total household expenditures for Q4 consumers (with per capita expenditures greater than $5.50 per day) in Tanzania

	Food Group																			Exp.
	Rice	Maize	Wheat	Cassava	Roots	Sugar	Pulses	Nuts	Vegetables	Fruit	RedMeat	Poultry	Eggs	Fish	Dairy	Oils	Coffee	SoftDrink	OtherFood	
1. Rice	-0.03	-0.14	0.09	-0.09	0.33	-0.01	-0.38	-0.05	-0.24	0.17	-0.42	0.06	-0.10	-0.01	-0.42	-0.20	0.04	-0.02	0.04	1.62
	(1.16)	(0.49)	(0.26)	(0.46)	(0.36)	(0.14)	(0.34)	(0.47)	(0.31)	(0.25)	(0.71)	(0.95)	(0.23)	(0.20)	(0.56)	(0.16)	(0.06)	(0.15)	(0.05)	(0.91)
2. Maize	-0.01	-0.21	-0.03	-0.36	-0.15	-0.10	0.13	0.19	-0.04	-0.04	0.56	-0.17	0.00	0.13	0.13	0.10	-0.01	0.11	-0.01	0.08
	(0.44)	(0.35)	(0.13)	(0.24)	(0.17)	(0.08)	(0.11)	(0.28)	(0.11)	(0.10)	(0.47)	(0.39)	(0.11)	(0.14)	(0.23)	(0.06)	(0.02)	(0.09)	(0.02)	(0.57)
3. Wheat	0.16	-0.13	0.34	-0.25	0.03	-0.28**	0.18	0.00	-0.29*	-0.04	0.12	0.15	0.03	-0.23	0.07	0.08	0.01	-0.01	-0.01	1.07**
	(0.26)	(0.18)	(0.38)	(0.16)	(0.15)	(0.11)	(0.18)	(0.15)	(0.13)	(0.13)	(0.26)	(0.26)	(0.07)	(0.13)	(0.20)	(0.06)	(0.04)	(0.07)	(0.03)	(0.25)
4. Cassava	-0.06	-0.64	-0.25	0.33	-0.60	-0.34*	0.13	-0.28	-0.04	0.22	0.43	-0.42	0.13	-0.17	0.76	-0.11	-0.10*	-0.00	-0.06	-0.07
	(0.73)	(0.41)	(0.23)	(0.68)	(0.34)	(0.14)	(0.18)	(0.40)	(0.16)	(0.18)	(0.54)	(0.63)	(0.20)	(0.18)	(0.55)	(0.06)	(0.04)	(0.12)	(0.04)	(0.82)
5. Roots	0.50	-0.25	0.06	-0.47	-0.17	-0.00	-0.15	-0.06	-0.08	-0.08	0.08	0.03	0.13	0.08	0.22	0.01	0.02	-0.04	0.05	0.49
	(0.42)	(0.23)	(0.16)	(0.26)	(0.33)	(0.07)	(0.16)	(0.22)	(0.11)	(0.11)	(0.31)	(0.31)	(0.11)	(0.13)	(0.23)	(0.04)	(0.03)	(0.08)	(0.03)	(0.41)
6. Sugar	0.03	-0.37*	-0.49**	-0.56**	-0.02	0.45	0.42	-0.14	-0.02	0.69**	-0.23	0.14	0.10	-0.13	-0.00	0.08	-0.17**	-0.03	-0.13*	0.81**
	(0.25)	(0.17)	(0.19)	(0.15)	(0.12)	(0.33)	(0.23)	(0.10)	(0.13)	(0.16)	(0.22)	(0.17)	(0.06)	(0.13)	(0.14)	(0.11)	(0.06)	(0.07)	(0.05)	(0.21)
7. Pulses	-0.62*	0.26	0.26	0.11	-0.22	0.32	1.16*	0.25	0.03	-0.18	-0.82*	0.20	-0.06	-0.24	-0.42	0.00	0.11	-0.04	0.14*	0.50
	(0.31)	(0.17)	(0.26)	(0.17)	(0.20)	(0.17)	(0.52)	(0.16)	(0.16)	(0.15)	(0.32)	(0.26)	(0.07)	(0.14)	(0.23)	(0.12)	(0.06)	(0.09)	(0.06)	(0.30)
8. Nuts	-0.18	0.31	-0.05	-0.38	-0.17	-0.15	0.23	-0.80	-0.08	-0.16	0.13	0.39	-0.16	-0.28	0.29	-0.01	0.01	-0.10	-0.03	2.27*
	(0.87)	(0.52)	(0.27)	(0.52)	(0.35)	(0.12)	(0.22)	(0.67)	(0.24)	(0.19)	(0.73)	(1.27)	(0.27)	(0.23)	(0.56)	(0.07)	(0.03)	(0.18)	(0.03)	(1.13)
9. Vegetables	-0.28	-0.09	-0.29	-0.07	-0.10	0.01	0.03	-0.00	-0.69**	0.01	0.12	-0.02	0.06	0.03	0.24	-0.10	-0.03	0.03	0.00	0.13
	(0.38)	(0.21)	(0.20)	(0.16)	(0.16)	(0.11)	(0.17)	(0.21)	(0.20)	(0.12)	(0.33)	(0.33)	(0.10)	(0.12)	(0.23)	(0.09)	(0.05)	(0.08)	(0.04)	(0.52)
10. Fruit	0.34	-0.15	-0.03	0.19	-0.13	0.47**	-0.19	-0.09	-0.02	-0.43*	-0.58*	0.01	0.01	0.13	-0.37*	0.02	-0.03	0.08	0.04	0.97**
	(0.28)	(0.16)	(0.17)	(0.15)	(0.14)	(0.10)	(0.14)	(0.13)	(0.10)	(0.19)	(0.24)	(0.18)	(0.06)	(0.11)	(0.17)	(0.05)	(0.03)	(0.07)	(0.03)	(0.21)
11. RedMeat	-0.43	0.44	0.04	0.18	-0.01	-0.12	-0.48	0.08	-0.01	-0.35	-0.84	0.13	-0.18	-0.03	-0.64	0.10	-0.01	-0.17	0.01	2.01**
	(0.52)	(0.31)	(0.19)	(0.24)	(0.19)	(0.10)	(0.27)	(0.30)	(0.14)	(0.20)	(0.45)	(0.52)	(0.15)	(0.13)	(0.37)	(0.07)	(0.03)	(0.13)	(0.03)	(0.64)
12. Poultry	0.06	-0.23	0.09	-0.25	0.00	0.04	0.08	0.18	-0.03	-0.01	0.14	-0.55	0.10	0.01	0.08	-0.02	-0.02	0.06	-0.00	1.21
	(0.85)	(0.41)	(0.23)	(0.37)	(0.24)	(0.08)	(0.16)	(0.60)	(0.18)	(0.12)	(0.60)	(1.10)	(0.21)	(0.17)	(0.42)	(0.05)	(0.03)	(0.12)	(0.03)	(0.95)
13. Eggs	-0.45	-0.15	0.02	0.22	0.29	0.10	-0.17	-0.27	0.04	-0.04	-0.78	0.35	-1.23**	-0.17	-0.52	-0.07	0.00	0.01	-0.02	2.58**
	(0.61)	(0.36)	(0.19)	(0.34)	(0.21)	(0.09)	(0.16)	(0.39)	(0.14)	(0.13)	(0.53)	(0.66)	(0.22)	(0.14)	(0.39)	(0.05)	(0.02)	(0.09)	(0.02)	(0.84)
14. Fish	0.02	0.18	-0.29	-0.22	0.09	-0.10	-0.25	-0.20	-0.00	0.12	0.01	0.04	-0.06	-0.50*	-0.18	-0.21**	-0.01	0.07	-0.02	0.98**
	(0.25)	(0.17)	(0.16)	(0.15)	(0.15)	(0.09)	(0.14)	(0.14)	(0.10)	(0.11)	(0.22)	(0.24)	(0.06)	(0.20)	(0.15)	(0.07)	(0.03)	(0.08)	(0.03)	(0.24)
15. Dairy	-0.83	0.10	0.03	0.72	0.22	-0.04	-0.47	0.25	0.16	-0.41	-1.22*	0.13	-0.25	-0.22	-1.54*	-0.03	0.08	0.01	0.03	2.33**
	(0.68)	(0.33)	(0.26)	(0.48)	(0.26)	(0.11)	(0.27)	(0.42)	(0.15)	(0.22)	(0.60)	(0.68)	(0.19)	(0.18)	(0.60)	(0.05)	(0.05)	(0.11)	(0.04)	(0.79)
16. Fats	-0.63**	0.33**	0.23	-0.28**	0.01	0.12	-0.00	0.03	-0.23	0.05	0.48**	-0.06	-0.03	-0.40**	0.02	0.68*	0.18**	-0.03	-0.19**	0.66**
	(0.20)	(0.12)	(0.15)	(0.09)	(0.09)	(0.15)	(0.22)	(0.09)	(0.14)	(0.09)	(0.18)	(0.10)	(0.04)	(0.11)	(0.09)	(0.35)	(0.06)	(0.05)	(0.07)	(0.17)
17. Coffee	0.45	-0.13	0.08	-0.55**	0.13	-0.61**	0.52	0.08	-0.18	-0.15	-0.02	-0.19	0.04	-0.06	0.45*	0.47**	-0.36*	0.12	-0.23**	0.82**
	(0.34)	(0.21)	(0.26)	(0.19)	(0.20)	(0.22)	(0.31)	(0.13)	(0.21)	(0.15)	(0.32)	(0.26)	(0.07)	(0.17)	(0.21)	(0.18)	(0.16)	(0.09)	(0.08)	(0.28)
18. SoftDrink	-0.14	0.68	-0.06	-0.03	-0.22	-0.09	-0.16	-0.27	0.08	0.25	-1.06	0.36	0.03	0.20	0.03	-0.07	0.07	-0.57	0.02	1.56*
	(0.78)	(0.54)	(0.36)	(0.43)	(0.38)	(0.19)	(0.32)	(0.51)	(0.28)	(0.29)	(0.80)	(0.77)	(0.20)	(0.33)	(0.44)	(0.10)	(0.07)	(0.34)	(0.06)	(0.74)
19. OtherFood	0.71	-0.15	-0.06	-0.55	0.57	-0.69*	1.06	-0.14	0.02	0.32	0.30	0.02	-0.05	-0.13	0.30	-0.76*	-0.35*	0.05	-1.49**	0.32
	(0.50)	(0.27)	(0.33)	(0.29)	(0.33)	(0.34)	(0.54)	(0.19)	(0.26)	(0.24)	(0.47)	(0.40)	(0.10)	(0.23)	(0.31)	(0.37)	(0.15)	(0.12)	(0.21)	(0.50)

Note: This table shows the sample-wide median elasticity of food demand (quantity consumed) with respect to food prices (columns 1 thru 19) and total household expenditures (the last column). For a list of items in each food group, see Suppl. Table 49.

Suppl. Table 17: Food demand elasticities with respect to food prices and total household expenditures for Q1 consumers (with per capita expenditures less than $1.90 per day) in Nigeria

	Food Group																			Exp.
	Rice	Maize	Wheat	Cassava	Roots	Sugar	Pulses	Nuts	Vegetables	Fruit	RedMeat	Poultry	Eggs	Fish	Dairy	Oils	Coffee	SoftDrink	OtherFood	
1. Rice	-0.58**	-0.03	-0.15	-0.16**	-0.01	-0.07*	-0.14**	0.18**	-0.14**	0.15**	0.30**	-0.05	0.08	0.07	-0.19**	-0.08**	0.10**	0.12**	-0.03	1.00**
	(0.10)	(0.05)	(0.09)	(0.04)	(0.08)	(0.03)	(0.04)	(0.04)	(0.05)	(0.06)	(0.10)	(0.07)	(0.05)	(0.06)	(0.06)	(0.03)	(0.03)	(0.04)	(0.02)	(0.11)
2. Maize	-0.13	-0.60**	0.11	-0.01	-0.10	-0.01	-0.13*	-0.01	-0.16**	0.01	-0.11	0.56**	0.00	-0.29**	-0.09	-0.02	0.07	-0.12*	0.02	2.01**
	(0.08)	(0.13)	(0.13)	(0.04)	(0.13)	(0.04)	(0.05)	(0.05)	(0.06)	(0.08)	(0.11)	(0.18)	(0.06)	(0.10)	(0.06)	(0.02)	(0.04)	(0.05)	(0.02)	(0.26)
3. Wheat	-0.12	0.12	-0.14	0.02	-0.10	-0.12*	-0.07	-0.09	-0.14*	-0.20*	-0.36*	-0.03	-0.09	-0.14	0.08	-0.03	0.05	-0.03	0.08*	1.12**
	(0.07)	(0.07)	(0.32)	(0.04)	(0.13)	(0.05)	(0.05)	(0.05)	(0.07)	(0.10)	(0.16)	(0.10)	(0.05)	(0.09)	(0.05)	(0.03)	(0.04)	(0.05)	(0.03)	(0.23)
4. Cassava	-0.22**	0.04	0.11	-0.52**	-0.74**	0.23**	-0.08	0.07	0.52**	0.04	0.13	-0.24**	-0.12**	-0.01	0.33**	-0.07**	0.20**	0.27**	-0.05*	0.49**
	(0.06)	(0.05)	(0.09)	(0.07)	(0.11)	(0.04)	(0.05)	(0.04)	(0.06)	(0.05)	(0.08)	(0.07)	(0.04)	(0.06)	(0.06)	(0.02)	(0.04)	(0.05)	(0.02)	(0.12)
5. Roots	0.02	-0.02	-0.10	-0.51**	-0.71**	0.05	0.03	0.06	-0.01	0.10	0.02	0.10	0.10	0.05	0.05	-0.06	-0.04	0.11	0.01	0.59
	(0.10)	(0.10)	(0.20)	(0.16)	(0.21)	(0.05)	(0.07)	(0.07)	(0.07)	(0.12)	(0.15)	(0.16)	(0.08)	(0.12)	(0.08)	(0.04)	(0.05)	(0.08)	(0.03)	(0.32)
6. Sugar	-0.26*	0.00	-0.62**	0.61**	0.17	-0.75**	-0.15	-0.21**	0.08	-0.15	0.09	0.02	-0.20	0.08	0.02	0.01	0.08	-0.64**	0.17**	0.76**
	(0.12)	(0.11)	(0.19)	(0.11)	(0.17)	(0.13)	(0.09)	(0.08)	(0.10)	(0.11)	(0.20)	(0.13)	(0.13)	(0.12)	(0.11)	(0.04)	(0.07)	(0.11)	(0.04)	(0.23)
7. Pulses	-0.19**	-0.09	-0.09	-0.09*	0.04	-0.06	-0.30**	-0.01	-0.10*	-0.06	-0.21**	-0.18*	-0.10*	0.15*	0.27**	-0.04	0.03	-0.08	0.03	0.55**
	(0.05)	(0.05)	(0.08)	(0.04)	(0.08)	(0.03)	(0.08)	(0.03)	(0.04)	(0.05)	(0.08)	(0.08)	(0.05)	(0.07)	(0.05)	(0.02)	(0.03)	(0.04)	(0.02)	(0.10)
8. Nuts	0.73**	0.03	-0.25	0.17	0.25	-0.15*	0.03	-0.71**	-0.06	0.28*	0.09	-0.28	0.06	0.18	-0.35**	0.15**	-0.07	0.27**	-0.14**	-0.16
	(0.17)	(0.12)	(0.21)	(0.09)	(0.18)	(0.07)	(0.09)	(0.11)	(0.09)	(0.13)	(0.18)	(0.27)	(0.11)	(0.12)	(0.11)	(0.05)	(0.06)	(0.09)	(0.05)	(0.42)
9. Vegetables	-0.14*	-0.06	-0.18*	0.42**	-0.02	0.03	-0.09*	-0.05	-0.99**	0.03	0.13	-0.15*	-0.04	0.07	0.16**	-0.03	0.02	0.10**	-0.05**	0.46**
	(0.06)	(0.04)	(0.08)	(0.07)	(0.07)	(0.03)	(0.04)	(0.03)	(0.07)	(0.05)	(0.09)	(0.07)	(0.06)	(0.05)	(0.05)	(0.02)	(0.03)	(0.04)	(0.02)	(0.11)
10. Fruit	0.88*	0.11	-0.88	0.16	0.54	-0.13	-0.07	0.37	0.25	-1.37**	1.12	-0.60	0.12	0.96*	-0.73*	0.23	-0.10	-0.14	0.05	-1.30
	(0.41)	(0.29)	(0.59)	(0.18)	(0.51)	(0.14)	(0.18)	(0.23)	(0.23)	(0.37)	(0.60)	(0.54)	(0.20)	(0.43)	(0.34)	(0.12)	(0.15)	(0.21)	(0.09)	(1.17)
11. RedMeat	0.36*	-0.09	-0.68*	0.07	-0.01	0.02	-0.21*	0.01	0.07	0.30*	-0.33	0.08	0.08	0.39**	-0.32**	0.04	-0.03	0.05	-0.04	1.45**
	(0.15)	(0.10)	(0.27)	(0.07)	(0.17)	(0.07)	(0.09)	(0.07)	(0.10)	(0.14)	(0.26)	(0.15)	(0.10)	(0.15)	(0.12)	(0.04)	(0.06)	(0.09)	(0.04)	(0.32)
12. Poultry	0.14	0.62**	0.17	-0.12*	0.25	0.02	-0.05	-0.10	0.02	-0.18	0.17	-2.42**	0.25**	0.11	-0.13	0.02	-0.24**	-0.02	-0.01	-1.48*
	(0.09)	(0.16)	(0.21)	(0.06)	(0.15)	(0.04)	(0.07)	(0.08)	(0.08)	(0.10)	(0.12)	(0.70)	(0.07)	(0.11)	(0.10)	(0.05)	(0.07)	(0.06)	(0.03)	(0.70)
13. Eggs	0.65	0.02	-0.95	-0.57**	0.72	-0.38	-0.51*	0.16	-0.23	0.22	0.49	1.50**	-0.59	1.22**	-0.21	0.25*	-0.63**	-0.31	-0.37**	1.16
	(0.40)	(0.35)	(0.50)	(0.21)	(0.45)	(0.24)	(0.26)	(0.23)	(0.37)	(0.30)	(0.59)	(0.51)	(0.51)	(0.40)	(0.28)	(0.12)	(0.20)	(0.24)	(0.13)	(0.84)
14. Fish	0.06	-0.23**	-0.27	-0.04	0.01	0.02	0.08	0.04	0.01	0.22*	0.38**	-0.00	0.19*	-0.76**	-0.11	-0.09**	-0.02	0.07	0.01	1.30**
	(0.08)	(0.09)	(0.16)	(0.05)	(0.13)	(0.04)	(0.07)	(0.04)	(0.05)	(0.09)	(0.14)	(0.13)	(0.08)	(0.11)	(0.07)	(0.03)	(0.04)	(0.06)	(0.03)	(0.21)
15. Dairy	-0.60**	-0.19	0.51*	0.74**	0.19	0.02	0.65**	-0.39**	0.52**	-0.62**	-0.87**	-0.40	-0.10	-0.26	-0.56**	0.28**	0.18*	-0.28**	0.10**	0.02
	(0.16)	(0.13)	(0.20)	(0.13)	(0.19)	(0.08)	(0.13)	(0.09)	(0.12)	(0.13)	(0.21)	(0.28)	(0.11)	(0.15)	(0.15)	(0.06)	(0.08)	(0.09)	(0.04)	(0.35)
16. Oils	-0.08*	0.07**	0.00	-0.07**	-0.09**	0.00	-0.04*	0.04**	-0.04	0.03	0.10**	-0.14**	0.05*	-0.06*	0.09**	-0.56**	0.02	-0.05*	-0.03**	0.52**
	(0.03)	(0.02)	(0.04)	(0.02)	(0.03)	(0.01)	(0.02)	(0.01)	(0.02)	(0.02)	(0.03)	(0.03)	(0.02)	(0.02)	(0.02)	(0.03)	(0.01)	(0.02)	(0.01)	(0.04)
17. Coffee	0.74**	0.36*	0.53	0.88**	-0.22	0.13	0.16	-0.14	0.16	-0.15	-0.14	-1.27**	-0.54**	-0.06	0.35*	0.14*	-0.81**	-0.09	0.16**	0.41
	(0.20)	(0.17)	(0.29)	(0.17)	(0.24)	(0.11)	(0.13)	(0.10)	(0.14)	(0.18)	(0.25)	(0.42)	(0.16)	(0.18)	(0.16)	(0.07)	(0.13)	(0.12)	(0.05)	(0.47)
18. SoftDrink	0.90**	-0.45	-0.10	1.08**	0.68	-0.94**	-0.23	0.49**	0.57**	-0.21	0.30	-0.11	-0.24	0.43	-0.48*	-0.13	-0.08	-1.20**	0.32**	-0.22
	(0.32)	(0.24)	(0.43)	(0.32)	(0.39)	(0.29)	(0.20)	(0.19)	(0.22)	(0.26)	(0.40)	(0.34)	(0.20)	(0.28)	(0.23)	(0.10)	(0.12)	(0.21)	(0.12)	(0.65)
19. OtherFood	-0.16	0.17	0.74**	-0.21*	0.04	0.27**	0.13	-0.30**	-0.29**	0.04	-0.15	-0.19	-0.31*	0.11	0.17*	-0.14**	0.15**	0.34**	-0.32	0.73**
	(0.10)	(0.09)	(0.23)	(0.09)	(0.14)	(0.09)	(0.09)	(0.10)	(0.11)	(0.09)	(0.16)	(0.14)	(0.12)	(0.11)	(0.08)	(0.05)	(0.06)	(0.11)	(0.17)	(0.23)

Note: This table shows the sample-wide median elasticity of food demand (quantity consumed) with respect to food prices (columns 1 thru 19) and total household expenditures (the last column). For a list of items in each food group, see Suppl. Table 50.

Suppl. Table 18: Food demand elasticities with respect to food prices and total household expenditures for Q2 consumers (with per capita expenditures between $1.90 and $3.20 per day) in Nigeria

	Food Group																			Exp.
	Rice	Maize	Wheat	Cassava	Roots	Sugar	Pulses	Nuts	Vegetables	Fruit	RedMeat	Poultry	Eggs	Fish	Dairy	Oils	Coffee	SoftDrink	OtherFood	
1. Rice	-0.57**	-0.04	0.01	-0.07*	-0.06	-0.03	-0.12**	0.11**	-0.08*	0.05	0.12	-0.07	0.05	-0.01	-0.11**	-0.02	0.05*	0.06*	0.00	0.86**
	(0.08)	(0.04)	(0.06)	(0.03)	(0.06)	(0.02)	(0.03)	(0.03)	(0.03)	(0.04)	(0.06)	(0.05)	(0.03)	(0.04)	(0.04)	(0.02)	(0.02)	(0.03)	(0.01)	(0.09)
2. Maize	-0.11*	-0.45**	-0.10	-0.04	0.03	-0.01	-0.09**	0.06	-0.07	0.10	0.05	0.44**	0.03	-0.14*	-0.08*	-0.00	0.05*	-0.02	-0.00	1.48**
	(0.06)	(0.11)	(0.10)	(0.03)	(0.10)	(0.02)	(0.03)	(0.04)	(0.04)	(0.06)	(0.09)	(0.12)	(0.04)	(0.06)	(0.04)	(0.01)	(0.02)	(0.04)	(0.02)	(0.20)
3. Wheat	-0.01	-0.03	-0.24	-0.04	0.10	-0.04	-0.09*	0.04	-0.05	0.02	-0.13	0.17	-0.03	0.04	0.03	-0.00	0.10*	0.02	0.04*	1.02**
	(0.06)	(0.07)	(0.26)	(0.04)	(0.13)	(0.03)	(0.04)	(0.04)	(0.05)	(0.08)	(0.11)	(0.10)	(0.04)	(0.08)	(0.04)	(0.02)	(0.04)	(0.05)	(0.02)	(0.25)
4. Cassava	-0.08*	-0.01	-0.03	-0.58**	-0.50**	0.10**	-0.16**	0.02	0.24**	0.03	0.11*	-0.14**	-0.06*	-0.00	0.22**	-0.04**	0.10**	0.12**	-0.03*	0.58**
	(0.04)	(0.03)	(0.06)	(0.05)	(0.08)	(0.02)	(0.03)	(0.02)	(0.03)	(0.04)	(0.05)	(0.04)	(0.02)	(0.04)	(0.04)	(0.01)	(0.02)	(0.03)	(0.01)	(0.09)
5. Roots	-0.06	0.04	0.12	-0.36**	-0.84**	0.02	0.01	-0.04	-0.07	-0.10	-0.12	-0.12	0.03	-0.05	0.04	-0.05	-0.07	-0.02	0.01	0.86**
	(0.08)	(0.08)	(0.16)	(0.12)	(0.17)	(0.04)	(0.04)	(0.05)	(0.06)	(0.11)	(0.13)	(0.11)	(0.04)	(0.08)	(0.06)	(0.03)	(0.04)	(0.06)	(0.02)	(0.28)
6. Sugar	-0.13	-0.03	-0.16	0.27**	0.08	-0.75**	-0.18**	-0.16**	0.07	-0.14	0.01	0.03	-0.10	0.11	0.03	0.02	0.02	-0.39**	0.09**	0.95**
	(0.09)	(0.07)	(0.12)	(0.07)	(0.13)	(0.09)	(0.06)	(0.05)	(0.06)	(0.08)	(0.14)	(0.09)	(0.09)	(0.09)	(0.07)	(0.03)	(0.05)	(0.07)	(0.03)	(0.18)
7. Pulses	-0.19**	-0.06*	-0.12*	-0.18**	0.02	-0.07**	-0.32**	-0.02	-0.05	0.01	-0.11	-0.10	-0.06	0.13**	0.14**	-0.03	0.07**	-0.00	0.02	0.67**
	(0.05)	(0.03)	(0.06)	(0.03)	(0.06)	(0.02)	(0.06)	(0.03)	(0.03)	(0.04)	(0.07)	(0.06)	(0.03)	(0.05)	(0.04)	(0.02)	(0.02)	(0.03)	(0.01)	(0.08)
8. Nuts	0.40**	0.14	0.17	0.04	-0.12	-0.12*	-0.02	-0.90**	-0.18*	-0.10	-0.22	-0.35	0.06	-0.07	-0.18*	0.09**	-0.13*	0.02	-0.04	0.75*
	(0.13)	(0.10)	(0.18)	(0.07)	(0.17)	(0.05)	(0.06)	(0.08)	(0.08)	(0.12)	(0.17)	(0.20)	(0.07)	(0.10)	(0.08)	(0.03)	(0.05)	(0.07)	(0.03)	(0.38)
9. Vegetables	-0.10	-0.03	-0.04	0.24**	-0.08	0.03	-0.05	-0.09**	-0.88**	-0.04	0.03	-0.16**	-0.02	-0.01	0.14**	-0.06**	-0.01	0.06*	-0.02	0.55**
	(0.05)	(0.04)	(0.08)	(0.05)	(0.07)	(0.02)	(0.03)	(0.03)	(0.05)	(0.05)	(0.07)	(0.06)	(0.05)	(0.04)	(0.04)	(0.02)	(0.02)	(0.03)	(0.01)	(0.12)
10. Fruit	0.22	0.28	0.12	0.09	-0.34	-0.12	0.04	-0.11	-0.08	-1.52**	0.00	-0.91	-0.08	0.04	-0.33	0.04	-0.14	-0.28	0.10	0.52
	(0.25)	(0.27)	(0.47)	(0.14)	(0.48)	(0.10)	(0.13)	(0.18)	(0.18)	(0.38)	(0.39)	(0.56)	(0.14)	(0.26)	(0.21)	(0.07)	(0.12)	(0.22)	(0.08)	(0.97)
11. RedMeat	0.08	0.04	-0.20	0.06	-0.16	-0.00	-0.10	-0.08	-0.02	-0.01	-0.56**	-0.14	0.05	0.04	-0.09	0.02	-0.05	-0.09	0.01	1.41**
	(0.08)	(0.07)	(0.15)	(0.05)	(0.13)	(0.04)	(0.05)	(0.05)	(0.06)	(0.09)	(0.17)	(0.09)	(0.06)	(0.08)	(0.06)	(0.02)	(0.04)	(0.06)	(0.02)	(0.26)
12. Poultry	-0.15*	0.33**	0.15	-0.13**	-0.18	0.00	-0.10*	-0.13*	-0.17**	-0.29**	-0.19*	-1.73**	0.09*	-0.13	-0.04	-0.11**	-0.17**	-0.12*	0.00	1.82**
	(0.07)	(0.10)	(0.13)	(0.04)	(0.11)	(0.02)	(0.04)	(0.06)	(0.05)	(0.09)	(0.10)	(0.43)	(0.04)	(0.08)	(0.06)	(0.03)	(0.05)	(0.05)	(0.02)	(0.45)
13. Eggs	0.27	0.15	-0.31	-0.33**	0.14	-0.18	-0.29*	0.12	-0.14	-0.16	0.28	0.52*	-0.65*	0.35	-0.16	0.15*	-0.41**	-0.27	-0.15*	1.66**
	(0.24)	(0.20)	(0.28)	(0.12)	(0.25)	(0.15)	(0.14)	(0.13)	(0.21)	(0.18)	(0.33)	(0.26)	(0.30)	(0.23)	(0.16)	(0.07)	(0.11)	(0.14)	(0.08)	(0.51)
14. Fish	-0.03	-0.08	0.06	-0.02	-0.07	0.03	0.07	-0.03	-0.03	0.00	0.07	-0.09	0.06	-0.86**	-0.04	-0.06**	-0.05*	-0.01	0.03	1.01**
	(0.04)	(0.05)	(0.09)	(0.03)	(0.08)	(0.02)	(0.04)	(0.03)	(0.03)	(0.05)	(0.07)	(0.07)	(0.04)	(0.06)	(0.04)	(0.02)	(0.03)	(0.04)	(0.02)	(0.14)
15. Dairy	-0.39**	-0.18	0.09	0.50**	0.13	0.02	0.27**	-0.19**	0.30**	-0.30**	-0.27	-0.10	-0.07	-0.13	-0.52**	0.06	0.16**	-0.19**	0.02	1.18**
	(0.12)	(0.09)	(0.15)	(0.09)	(0.15)	(0.05)	(0.08)	(0.06)	(0.09)	(0.10)	(0.15)	(0.17)	(0.07)	(0.10)	(0.12)	(0.03)	(0.05)	(0.07)	(0.03)	(0.25)
16. Oils	-0.01	0.04*	0.04	-0.05**	-0.06*	0.01	-0.03	0.04**	-0.06**	0.01	0.08**	-0.11**	0.05**	-0.06**	0.04*	-0.59**	0.02	-0.04**	-0.01	0.53**
	(0.03)	(0.02)	(0.03)	(0.01)	(0.03)	(0.01)	(0.02)	(0.01)	(0.02)	(0.02)	(0.03)	(0.02)	(0.02)	(0.02)	(0.02)	(0.03)	(0.01)	(0.02)	(0.01)	(0.04)
17. Coffee	0.25*	0.18	0.51**	0.36**	-0.41*	0.02	0.21**	-0.22**	-0.05	-0.20	-0.24	-0.74**	-0.31**	-0.28*	0.26**	0.03	-0.79**	-0.06	0.12**	1.32**
	(0.13)	(0.10)	(0.18)	(0.10)	(0.17)	(0.06)	(0.08)	(0.07)	(0.08)	(0.11)	(0.16)	(0.23)	(0.09)	(0.12)	(0.09)	(0.04)	(0.08)	(0.07)	(0.03)	(0.34)
18. SoftDrink	0.30	-0.05	0.11	0.42*	-0.10	-0.48**	-0.01	0.03	0.20	-0.38	-0.38	-0.51	-0.20	-0.05	-0.30	-0.13	-0.06	-1.05**	0.22**	0.91
	(0.20)	(0.16)	(0.32)	(0.17)	(0.31)	(0.17)	(0.12)	(0.11)	(0.14)	(0.23)	(0.31)	(0.29)	(0.13)	(0.19)	(0.15)	(0.08)	(0.08)	(0.14)	(0.08)	(0.61)
19. OtherFood	0.02	0.01	0.34*	-0.18*	0.06	0.16**	0.09	-0.09	-0.12	0.20	0.12	0.06	-0.14	0.19	0.05	-0.07	0.17**	0.33**	-0.39**	0.87**
	(0.08)	(0.08)	(0.15)	(0.08)	(0.14)	(0.06)	(0.07)	(0.06)	(0.08)	(0.10)	(0.13)	(0.10)	(0.08)	(0.10)	(0.06)	(0.04)	(0.06)	(0.10)	(0.15)	(0.24)

Note: This table shows the sample-wide median elasticity of food demand (quantity consumed) with respect to food prices (columns 1 thru 19) and total household expenditures (the last column). For a list of items in each food group, see Suppl. Table 50.

Suppl. Table 19: Food demand elasticities with respect to food prices and total household expenditures for Q3 consumers (with per capita expenditures between \$3.20 and \$5.50 per day) in Nigeria

	Food Group																			Exp.
	Rice	Maize	Wheat	Cassava	Roots	Sugar	Pulses	Nuts	Vegetables	Fruit	RedMeat	Poultry	Eggs	Fish	Dairy	Oils	Coffee	SoftDrink	OtherFood	
1. Rice	-0.49**	-0.06	0.15*	0.01	-0.10	0.00	-0.12**	0.06*	-0.04	-0.04	-0.03	-0.10	0.03	-0.08	-0.05	0.03	0.02	0.01	0.03**	0.55**
	(0.08)	(0.04)	(0.07)	(0.03)	(0.07)	(0.02)	(0.03)	(0.03)	(0.03)	(0.05)	(0.07)	(0.05)	(0.03)	(0.04)	(0.03)	(0.02)	(0.02)	(0.03)	(0.01)	(0.12)
2. Maize	-0.12	-0.23	-0.38**	-0.08*	0.15	-0.02	-0.05	0.13**	0.00	0.22*	0.22	0.36**	0.07	0.00	-0.10*	-0.01	0.04	0.08	-0.03	1.16**
	(0.07)	(0.14)	(0.13)	(0.04)	(0.13)	(0.03)	(0.03)	(0.05)	(0.04)	(0.09)	(0.12)	(0.12)	(0.04)	(0.07)	(0.04)	(0.02)	(0.03)	(0.05)	(0.02)	(0.27)
3. Wheat	0.09	-0.24*	-0.48	-0.13*	0.32	0.05	-0.11*	0.20*	0.04	0.28	0.12	0.40*	0.03	0.24*	-0.04	0.02	0.14*	0.07	-0.00	1.35**
	(0.08)	(0.12)	(0.26)	(0.06)	(0.20)	(0.03)	(0.05)	(0.09)	(0.07)	(0.15)	(0.15)	(0.18)	(0.04)	(0.12)	(0.05)	(0.02)	(0.06)	(0.07)	(0.02)	(0.37)
4. Cassava	0.02	-0.05	-0.13*	-0.57**	-0.41**	0.01	-0.22**	-0.02	0.06*	0.03	0.12*	-0.08*	-0.03	0.01	0.18**	-0.02	0.04	0.03	-0.02	0.63**
	(0.04)	(0.03)	(0.06)	(0.05)	(0.07)	(0.02)	(0.03)	(0.02)	(0.03)	(0.04)	(0.05)	(0.03)	(0.02)	(0.04)	(0.03)	(0.01)	(0.02)	(0.03)	(0.01)	(0.10)
5. Roots	-0.09	0.10	0.33	-0.28**	-0.89**	0.01	0.01	-0.12	-0.09	-0.25*	-0.22	-0.29*	-0.02	-0.10	0.05	-0.02	-0.09*	-0.11	0.01	0.67*
	(0.07)	(0.08)	(0.18)	(0.09)	(0.17)	(0.03)	(0.03)	(0.06)	(0.05)	(0.12)	(0.13)	(0.13)	(0.03)	(0.08)	(0.05)	(0.02)	(0.04)	(0.07)	(0.02)	(0.28)
6. Sugar	-0.04	-0.05	0.19	0.00	-0.00	-0.76**	-0.21**	-0.12*	0.05	-0.14	-0.07	0.04	-0.02	0.12	0.03	0.02	-0.03	-0.19**	0.03	1.38**
	(0.09)	(0.07)	(0.11)	(0.06)	(0.13)	(0.07)	(0.06)	(0.05)	(0.06)	(0.07)	(0.13)	(0.07)	(0.08)	(0.09)	(0.06)	(0.03)	(0.04)	(0.06)	(0.02)	(0.17)
7. Pulses	-0.21**	-0.05	-0.15**	-0.27**	0.00	-0.08**	-0.29**	-0.03	-0.01	0.08*	-0.03	-0.03	-0.03	0.11**	0.04	-0.02	0.11**	0.06*	0.01	0.83**
	(0.05)	(0.03)	(0.05)	(0.04)	(0.05)	(0.02)	(0.06)	(0.02)	(0.03)	(0.04)	(0.06)	(0.04)	(0.03)	(0.04)	(0.04)	(0.02)	(0.02)	(0.03)	(0.01)	(0.09)
8. Nuts	0.18	0.25*	0.61**	-0.04	-0.40	-0.10*	-0.05	-1.04**	-0.25**	-0.41**	-0.47*	-0.42*	0.06	-0.25*	-0.05	0.08**	-0.18**	-0.17*	0.04	0.94*
	(0.10)	(0.11)	(0.22)	(0.06)	(0.21)	(0.04)	(0.05)	(0.08)	(0.08)	(0.16)	(0.19)	(0.18)	(0.06)	(0.12)	(0.06)	(0.03)	(0.06)	(0.08)	(0.03)	(0.41)
9. Vegetables	-0.05	0.02	0.13	0.07*	-0.14	0.03	0.00	-0.12**	-0.76**	-0.11*	-0.06	-0.17**	-0.00	-0.07	0.13**	-0.07**	-0.04	0.02	0.01	0.48**
	(0.05)	(0.04)	(0.09)	(0.04)	(0.08)	(0.02)	(0.03)	(0.04)	(0.05)	(0.05)	(0.07)	(0.06)	(0.04)	(0.04)	(0.04)	(0.02)	(0.02)	(0.03)	(0.01)	(0.14)
10. Fruit	-0.13	0.40	0.85	0.06	-0.84	-0.10	0.13	-0.41	-0.22	-1.59**	-0.68	-1.06	-0.21	-0.48	-0.08	-0.02	-0.15	-0.36	0.14	0.94
	(0.19)	(0.29)	(0.57)	(0.11)	(0.56)	(0.08)	(0.12)	(0.24)	(0.17)	(0.39)	(0.46)	(0.60)	(0.13)	(0.29)	(0.11)	(0.05)	(0.11)	(0.23)	(0.09)	(0.82)
11. RedMeat	-0.06	0.14	0.12	0.06	-0.25*	-0.01	-0.03	-0.15**	-0.06	-0.21*	-0.66**	-0.28**	0.03	-0.16*	0.06	0.01	-0.06	-0.18**	0.05*	1.06**
	(0.07)	(0.07)	(0.13)	(0.04)	(0.13)	(0.03)	(0.04)	(0.06)	(0.05)	(0.10)	(0.14)	(0.10)	(0.03)	(0.07)	(0.04)	(0.02)	(0.03)	(0.06)	(0.02)	(0.24)
12. Poultry	-0.25**	0.18**	0.25	-0.15**	-0.45**	-0.01	-0.10**	-0.15**	-0.22**	-0.36**	-0.42**	-1.29**	-0.02	-0.29**	0.00	-0.12**	-0.12**	-0.20**	0.02	3.12**
	(0.06)	(0.07)	(0.14)	(0.03)	(0.13)	(0.02)	(0.03)	(0.05)	(0.05)	(0.10)	(0.11)	(0.19)	(0.02)	(0.07)	(0.04)	(0.02)	(0.03)	(0.05)	(0.02)	(0.52)
13. Eggs	0.04	0.22	0.11	-0.16	-0.22	-0.04	-0.13	0.09	-0.08	-0.38**	0.12	-0.09	-0.71**	-0.19	-0.12	0.08	-0.26**	-0.24**	-0.01	2.11**
	(0.15)	(0.13)	(0.20)	(0.09)	(0.18)	(0.10)	(0.09)	(0.09)	(0.13)	(0.14)	(0.20)	(0.13)	(0.17)	(0.13)	(0.10)	(0.05)	(0.07)	(0.09)	(0.05)	(0.39)
14. Fish	-0.08	0.01	0.30**	0.00	-0.11	0.04*	0.07**	-0.08*	-0.05	-0.16**	-0.15*	-0.15*	-0.02	-0.90**	0.02	-0.03*	-0.08**	-0.07*	0.04**	0.57**
	(0.04)	(0.04)	(0.10)	(0.03)	(0.08)	(0.02)	(0.03)	(0.03)	(0.03)	(0.06)	(0.07)	(0.06)	(0.03)	(0.05)	(0.03)	(0.01)	(0.02)	(0.04)	(0.01)	(0.15)
15. Dairy	-0.23*	-0.16*	-0.15	0.32**	0.06	0.02	0.02	-0.06	0.16*	-0.08	0.11	0.10	-0.05	-0.05	-0.52**	-0.07*	0.13**	-0.14*	-0.03	2.01**
	(0.10)	(0.08)	(0.14)	(0.07)	(0.14)	(0.04)	(0.06)	(0.05)	(0.07)	(0.08)	(0.12)	(0.10)	(0.05)	(0.07)	(0.10)	(0.03)	(0.04)	(0.06)	(0.02)	(0.25)
16. Oils	0.06	0.00	0.09*	-0.03	-0.03	0.02	-0.01	0.05**	-0.09**	-0.01	0.06	-0.06**	0.05**	-0.06**	-0.01	-0.64**	0.01	-0.04*	0.00	0.55**
	(0.03)	(0.02)	(0.04)	(0.02)	(0.03)	(0.01)	(0.02)	(0.01)	(0.02)	(0.02)	(0.03)	(0.02)	(0.01)	(0.02)	(0.02)	(0.03)	(0.01)	(0.02)	(0.01)	(0.05)
17. Coffee	0.02	0.10	0.50**	0.08	-0.47**	-0.03	0.24**	-0.25**	-0.14*	-0.21*	-0.27*	-0.44**	-0.18**	-0.37**	0.20**	-0.00	-0.80**	-0.05	0.10**	1.62**
	(0.09)	(0.07)	(0.14)	(0.07)	(0.15)	(0.04)	(0.06)	(0.07)	(0.06)	(0.09)	(0.13)	(0.13)	(0.06)	(0.10)	(0.07)	(0.03)	(0.06)	(0.06)	(0.03)	(0.31)
18. SoftDrink	0.00	0.19	0.28	0.06	-0.51	-0.20*	0.14	-0.22	0.02	-0.46*	-0.74*	-0.71*	-0.16	-0.30	-0.18	-0.10	-0.05	-0.96**	0.16*	1.13*
	(0.15)	(0.15)	(0.27)	(0.09)	(0.31)	(0.09)	(0.09)	(0.12)	(0.10)	(0.23)	(0.33)	(0.31)	(0.09)	(0.17)	(0.10)	(0.05)	(0.06)	(0.11)	(0.07)	(0.54)
19. OtherFood	0.20	-0.16	-0.00	-0.15	0.07	0.06	0.05	0.10	0.02	0.35*	0.39*	0.31*	0.00	0.25*	-0.07	-0.00	0.19**	0.32**	-0.46**	1.13**
	(0.10)	(0.10)	(0.14)	(0.08)	(0.17)	(0.05)	(0.07)	(0.07)	(0.08)	(0.14)	(0.17)	(0.14)	(0.07)	(0.12)	(0.07)	(0.03)	(0.07)	(0.10)	(0.13)	(0.29)

Note: This table shows the sample-wide median elasticity of food demand (quantity consumed) with respect to food prices (columns 1 thru 19) and total household expenditures (the last column). For a list of items in each food group, see Suppl. Table 50.

Suppl. Table 20: Food demand elasticities with respect to food prices and total household expenditures for Q4 consumers (with per capita expenditures greater than $5.50 per day) in Nigeria

	Food Group																			Exp.
	Rice	Maize	Wheat	Cassava	Roots	Sugar	Pulses	Nuts	Vegetables	Fruit	RedMeat	Poultry	Eggs	Fish	Dairy	Oils	Coffee	SoftDrink	OtherFood	
1. Rice	-0.37**	-0.10	0.37**	0.13**	-0.17	0.05	-0.13**	-0.01	0.02	-0.16*	-0.27*	-0.15	-0.00	-0.21**	0.04	0.11**	-0.02	-0.06	0.08**	0.16
	(0.12)	(0.07)	(0.14)	(0.05)	(0.12)	(0.04)	(0.05)	(0.04)	(0.05)	(0.08)	(0.12)	(0.10)	(0.03)	(0.08)	(0.05)	(0.03)	(0.03)	(0.06)	(0.02)	(0.22)
2. Maize	-0.19	-0.02	-0.89**	-0.16*	0.29	-0.04	-0.04	0.24**	0.05	0.37**	0.45*	0.18	0.12*	0.14	-0.11	-0.06*	0.02	0.21**	-0.09**	1.62**
	(0.11)	(0.22)	(0.21)	(0.06)	(0.19)	(0.05)	(0.05)	(0.08)	(0.07)	(0.14)	(0.18)	(0.17)	(0.06)	(0.11)	(0.07)	(0.03)	(0.05)	(0.08)	(0.03)	(0.35)
3. Wheat	0.23	-0.51*	-0.83**	-0.25*	0.58*	0.18**	-0.14*	0.40*	0.17	0.62*	0.45	0.66*	0.12	0.48*	-0.14	0.04	0.17*	0.13	-0.07	2.06**
	(0.13)	(0.21)	(0.29)	(0.11)	(0.28)	(0.07)	(0.07)	(0.16)	(0.10)	(0.24)	(0.24)	(0.29)	(0.07)	(0.19)	(0.08)	(0.03)	(0.07)	(0.09)	(0.04)	(0.56)
4. Cassava	0.15**	-0.10*	-0.25**	-0.52**	-0.32**	-0.10**	-0.30**	-0.06	-0.14**	0.04	0.14	-0.01	0.01	0.03	0.15**	0.02	-0.03	-0.09*	-0.01	0.38**
	(0.05)	(0.05)	(0.09)	(0.07)	(0.09)	(0.03)	(0.04)	(0.03)	(0.05)	(0.05)	(0.08)	(0.05)	(0.03)	(0.05)	(0.05)	(0.02)	(0.03)	(0.04)	(0.02)	(0.12)
5. Roots	-0.13	0.18	0.63*	-0.20*	-0.92**	-0.00	0.02	-0.22*	-0.11	-0.44*	-0.33	-0.49*	-0.08	-0.15	0.07	0.02	-0.12*	-0.22*	0.02	-0.01
	(0.10)	(0.12)	(0.31)	(0.08)	(0.22)	(0.04)	(0.05)	(0.10)	(0.07)	(0.20)	(0.19)	(0.22)	(0.05)	(0.12)	(0.07)	(0.02)	(0.06)	(0.11)	(0.03)	(0.44)
6. Sugar	0.10	-0.07	0.66**	-0.29**	-0.07	-0.77**	-0.23**	-0.08	0.06	-0.12	-0.14	0.05	0.07	0.17	0.05	0.04	-0.08	0.02	-0.03	1.39**
	(0.12)	(0.10)	(0.14)	(0.09)	(0.16)	(0.08)	(0.07)	(0.07)	(0.07)	(0.09)	(0.18)	(0.09)	(0.08)	(0.12)	(0.08)	(0.04)	(0.05)	(0.07)	(0.03)	(0.21)
7. Pulses	-0.21**	-0.02	-0.17*	-0.39**	-0.01	-0.10**	-0.23**	-0.04	0.06	0.17**	0.08	0.06	0.02	0.10	-0.09	0.00	0.17**	0.16**	0.01	0.77**
	(0.06)	(0.05)	(0.07)	(0.05)	(0.07)	(0.03)	(0.08)	(0.03)	(0.05)	(0.05)	(0.08)	(0.05)	(0.04)	(0.06)	(0.05)	(0.02)	(0.03)	(0.04)	(0.02)	(0.11)
8. Nuts	0.00	0.41*	1.21**	-0.11	-0.67*	-0.05	-0.04	-1.20**	-0.28*	-0.77**	-0.71*	-0.48	0.06	-0.41*	0.11	0.11**	-0.24**	-0.39**	0.15**	0.19
	(0.12)	(0.17)	(0.38)	(0.08)	(0.30)	(0.06)	(0.06)	(0.13)	(0.11)	(0.26)	(0.28)	(0.25)	(0.07)	(0.17)	(0.08)	(0.04)	(0.08)	(0.14)	(0.05)	(0.50)
9. Vegetables	0.03	0.09	0.41*	-0.17*	-0.22	0.04	0.08	-0.18**	-0.56**	-0.20*	-0.20	-0.18	0.02	-0.16*	0.11	-0.09**	-0.07	-0.04	0.06*	0.03
	(0.08)	(0.07)	(0.17)	(0.07)	(0.12)	(0.03)	(0.06)	(0.06)	(0.10)	(0.09)	(0.12)	(0.10)	(0.05)	(0.07)	(0.06)	(0.03)	(0.04)	(0.06)	(0.03)	(0.25)
10. Fruit	-0.34	0.56	1.67	0.09	-1.22	-0.07	0.28	-0.68	-0.28	-1.65**	-1.22	-1.20	-0.32	-0.86	0.17	-0.02	-0.15	-0.42	0.20	-0.29
	(0.27)	(0.39)	(1.06)	(0.13)	(0.81)	(0.09)	(0.18)	(0.40)	(0.22)	(0.49)	(0.78)	(0.74)	(0.19)	(0.51)	(0.16)	(0.05)	(0.13)	(0.30)	(0.13)	(1.09)
11. RedMeat	-0.22*	0.26*	0.49*	0.10	-0.35	-0.02	0.05	-0.23**	-0.11	-0.45**	-0.70**	-0.46**	0.03	-0.38**	0.24**	0.02	-0.07	-0.29**	0.10**	0.32
	(0.10)	(0.11)	(0.23)	(0.06)	(0.18)	(0.05)	(0.05)	(0.09)	(0.07)	(0.17)	(0.17)	(0.17)	(0.04)	(0.13)	(0.08)	(0.02)	(0.04)	(0.11)	(0.04)	(0.34)
12. Poultry	-0.18**	0.08	0.44**	-0.06	-0.54**	-0.00	-0.02	-0.14**	-0.15**	-0.39**	-0.50**	-0.90**	-0.11**	-0.28**	0.07	-0.04**	-0.07*	-0.25**	0.06**	2.51**
	(0.06)	(0.07)	(0.16)	(0.03)	(0.14)	(0.02)	(0.03)	(0.05)	(0.05)	(0.11)	(0.12)	(0.19)	(0.03)	(0.07)	(0.04)	(0.02)	(0.03)	(0.06)	(0.02)	(0.41)
13. Eggs	-0.07	0.29*	0.50*	0.00	-0.43*	0.07	0.02	0.08	0.02	-0.54**	0.06	-0.57**	-0.76**	-0.54**	-0.07	0.06	-0.12	-0.20*	0.11*	1.61**
	(0.12)	(0.13)	(0.23)	(0.10)	(0.21)	(0.09)	(0.10)	(0.09)	(0.13)	(0.15)	(0.20)	(0.14)	(0.15)	(0.14)	(0.10)	(0.04)	(0.07)	(0.08)	(0.05)	(0.34)
14. Fish	-0.19**	0.12	0.60**	0.02	-0.19	0.06	0.07	-0.16**	-0.10*	-0.36**	-0.43**	-0.23*	-0.12**	-0.95**	0.08*	-0.02	-0.11**	-0.14*	0.05*	0.21
	(0.07)	(0.07)	(0.18)	(0.04)	(0.13)	(0.04)	(0.04)	(0.05)	(0.04)	(0.10)	(0.13)	(0.10)	(0.04)	(0.08)	(0.04)	(0.02)	(0.04)	(0.06)	(0.02)	(0.23)
15. Dairy	-0.01	-0.13	-0.31*	0.19**	0.06	0.02	-0.16*	0.07	0.08	0.11	0.50**	0.27*	-0.04	0.09	-0.51**	-0.14**	0.12*	-0.08	-0.07**	1.95**
	(0.10)	(0.08)	(0.15)	(0.07)	(0.15)	(0.05)	(0.07)	(0.05)	(0.07)	(0.08)	(0.13)	(0.12)	(0.05)	(0.08)	(0.10)	(0.04)	(0.05)	(0.06)	(0.03)	(0.24)
16. Oils	0.17**	-0.05	0.19**	0.02	0.02	0.04	0.01	0.06**	-0.12**	-0.04	0.02	0.01	0.04*	-0.05	-0.09**	-0.73**	0.01	-0.03	0.03**	0.43**
	(0.04)	(0.03)	(0.05)	(0.03)	(0.05)	(0.02)	(0.02)	(0.02)	(0.03)	(0.03)	(0.05)	(0.03)	(0.02)	(0.03)	(0.03)	(0.04)	(0.02)	(0.03)	(0.01)	(0.07)
17. Coffee	-0.09	0.05	0.58**	-0.09	-0.49**	-0.07	0.31**	-0.28**	-0.17*	-0.22*	-0.26	-0.21	-0.09	-0.39**	0.19**	-0.00	-0.77**	-0.04	0.09**	1.03**
	(0.09)	(0.08)	(0.19)	(0.07)	(0.17)	(0.05)	(0.07)	(0.08)	(0.07)	(0.11)	(0.14)	(0.13)	(0.05)	(0.11)	(0.07)	(0.03)	(0.07)	(0.07)	(0.03)	(0.27)
18. SoftDrink	-0.17	0.41	0.50	-0.19	-0.78	0.03	0.30*	-0.43*	-0.07	-0.52	-1.00*	-0.88*	-0.14	-0.43	-0.08	-0.04	-0.03	-0.88**	0.13	0.28
	(0.17)	(0.22)	(0.36)	(0.11)	(0.41)	(0.07)	(0.13)	(0.20)	(0.11)	(0.29)	(0.46)	(0.41)	(0.09)	(0.23)	(0.09)	(0.05)	(0.08)	(0.12)	(0.07)	(0.61)
19. OtherFood	0.44**	-0.38*	-0.49*	-0.12	0.07	-0.06	0.01	0.37**	0.22	0.55**	0.74**	0.64**	0.21*	0.33*	-0.24*	0.09	0.21*	0.28*	-0.57**	1.59**
	(0.17)	(0.16)	(0.24)	(0.11)	(0.23)	(0.06)	(0.09)	(0.12)	(0.12)	(0.20)	(0.26)	(0.24)	(0.10)	(0.15)	(0.11)	(0.05)	(0.09)	(0.11)	(0.12)	(0.39)

Note: This table shows the sample-wide median elasticity of food demand (quantity consumed) with respect to food prices (columns 1 thru 19) and total household expenditures (the last column). For a list of items in each food group, see Suppl. Table 50.

Suppl. Table 21: Nutrient intake elasticities with respect to food prices and total household expenditures for Q1 consumers (with per capita expenditures less than $1.90 per day) in Malawi

	Food Group																		Exp.
	Rice	Maize	Wheat	Cassava	Roots	Sugar	Pulses	Nuts	Vegetables	Fruit	RedMeat	Poultry	Eggs	Dairy	Oils	Tea	SoftDrink	OtherFood	Exp.
Kcal	0.04	-0.38**	-0.02	-0.02	-0.09**	-0.06**	-0.08**	-0.02	-0.06	0.00	-0.02	-0.03	-0.02	-0.04	0.00	-0.00	0.00	-0.02**	0.86**
	(0.05)	(0.07)	(0.03)	(0.04)	(0.03)	(0.02)	(0.03)	(0.02)	(0.04)	(0.03)	(0.05)	(0.07)	(0.02)	(0.04)	(0.02)	(0.01)	(0.03)	(0.01)	(0.08)
Protein	0.04	-0.43**	-0.03	-0.01	-0.08**	-0.03	-0.12**	-0.01	-0.07	0.02	-0.03	-0.06	-0.02	-0.07	0.01	0.00	-0.01	-0.02**	0.97**
	(0.04)	(0.07)	(0.02)	(0.04)	(0.03)	(0.02)	(0.04)	(0.02)	(0.04)	(0.03)	(0.05)	(0.08)	(0.03)	(0.05)	(0.02)	(0.01)	(0.03)	(0.01)	(0.08)
Fat	0.15*	-0.39**	-0.13**	-0.00	-0.06	0.00	-0.03	-0.09**	-0.00	-0.02	-0.02	-0.12	-0.02	-0.15**	-0.15**	-0.01	-0.02	-0.04**	1.32**
	(0.07)	(0.08)	(0.05)	(0.05)	(0.04)	(0.04)	(0.05)	(0.03)	(0.06)	(0.03)	(0.09)	(0.10)	(0.04)	(0.06)	(0.04)	(0.01)	(0.04)	(0.01)	(0.11)
Carbohydrates	0.02	-0.37**	-0.01	-0.02	-0.09**	-0.08**	-0.09**	-0.01	-0.06	-0.00	-0.02	-0.01	-0.02	-0.02	0.01	-0.00	0.01	-0.01	0.79**
	(0.05)	(0.07)	(0.03)	(0.04)	(0.03)	(0.02)	(0.03)	(0.02)	(0.04)	(0.03)	(0.05)	(0.07)	(0.03)	(0.05)	(0.02)	(0.01)	(0.03)	(0.01)	(0.07)
Iron	0.05	-0.36**	-0.05	-0.06	-0.14**	-0.03	-0.12**	-0.02	-0.11*	0.02	0.01	-0.03	-0.02	-0.00	0.01	0.01	0.01	-0.02**	0.87**
	(0.05)	(0.07)	(0.03)	(0.04)	(0.03)	(0.02)	(0.04)	(0.02)	(0.05)	(0.03)	(0.05)	(0.08)	(0.03)	(0.05)	(0.02)	(0.01)	(0.03)	(0.01)	(0.09)
Zinc	0.04	-0.42**	0.00	0.00	-0.07*	-0.04*	-0.10**	-0.01	-0.07	0.01	-0.03	-0.04	-0.02	-0.09*	0.01	0.00	-0.01	-0.02**	0.88**
	(0.04)	(0.07)	(0.02)	(0.04)	(0.03)	(0.02)	(0.03)	(0.02)	(0.04)	(0.03)	(0.05)	(0.07)	(0.02)	(0.04)	(0.02)	(0.01)	(0.03)	(0.01)	(0.08)
Vitamin A	0.14*	-0.32**	-0.12**	-0.04	-0.23**	0.04	-0.00	-0.06*	-0.12	0.02	-0.00	-0.04	-0.00	-0.03	-0.08*	-0.01	0.03	-0.02*	0.97**
	(0.07)	(0.07)	(0.05)	(0.05)	(0.05)	(0.04)	(0.04)	(0.03)	(0.07)	(0.04)	(0.08)	(0.09)	(0.04)	(0.06)	(0.03)	(0.01)	(0.04)	(0.01)	(0.12)
Total Folate	0.02	-0.36**	-0.04	-0.08	-0.13**	-0.03	-0.16**	-0.02	-0.12*	0.01	0.01	-0.04	0.01	-0.02	0.02	-0.00	0.00	-0.02*	0.87**
	(0.05)	(0.07)	(0.03)	(0.05)	(0.04)	(0.03)	(0.05)	(0.03)	(0.05)	(0.04)	(0.06)	(0.10)	(0.03)	(0.05)	(0.02)	(0.01)	(0.04)	(0.01)	(0.11)

Note: This table shows the sample-wide median elasticity of macro- and micro-nutrient intake (quantity demanded) with respect to food prices (columns 1 thru 18) and total household expenditures (the last column). For a list of items in each food group, see Suppl. Table 46.

Suppl. Table 22: Nutrient intake elasticities with respect to food prices and total household expenditures for Q2 consumers (with per capita expenditures between $1.90 and $3.20 per day) in Malawi

	Food Group																		Exp.
	Rice	Maize	Wheat	Cassava	Roots	Sugar	Pulses	Nuts	Vegetables	Fruit	RedMeat	Poultry	Eggs	Dairy	Oils	Tea	SoftDrink	OtherFood	Exp.
Kcal	-0.04	-0.24**	-0.02	-0.05	-0.07**	-0.08**	-0.06*	0.00	-0.06	-0.01	0.05	0.03	-0.01	-0.04	-0.00	-0.00	0.02	-0.01**	0.83**
	(0.04)	(0.05)	(0.02)	(0.03)	(0.02)	(0.01)	(0.03)	(0.01)	(0.03)	(0.02)	(0.04)	(0.07)	(0.02)	(0.03)	(0.01)	(0.00)	(0.02)	(0.00)	(0.07)
Protein	-0.02	-0.28**	-0.03	-0.05	-0.06*	-0.03*	-0.08*	0.01	-0.05	0.01	0.03	0.02	-0.02	-0.06	0.02	0.01	0.00	-0.01**	0.96**
	(0.04)	(0.06)	(0.02)	(0.03)	(0.03)	(0.02)	(0.04)	(0.02)	(0.03)	(0.02)	(0.05)	(0.09)	(0.02)	(0.03)	(0.01)	(0.01)	(0.03)	(0.00)	(0.08)
Fat	0.05	-0.23**	-0.08*	-0.03	-0.05	0.02	0.00	-0.06*	0.03	-0.03	0.09	-0.05	-0.03	-0.15**	-0.18**	0.01	0.03	-0.04**	1.35**
	(0.05)	(0.07)	(0.04)	(0.04)	(0.04)	(0.03)	(0.05)	(0.03)	(0.05)	(0.03)	(0.09)	(0.12)	(0.04)	(0.05)	(0.04)	(0.01)	(0.04)	(0.01)	(0.12)
Carbohydrates	-0.06	-0.24**	-0.00	-0.05	-0.07**	-0.10**	-0.07**	0.01	-0.07*	-0.01	0.04	0.04	-0.01	-0.02	0.02	-0.00	0.02	-0.01	0.74**
	(0.04)	(0.06)	(0.02)	(0.03)	(0.02)	(0.02)	(0.03)	(0.01)	(0.03)	(0.02)	(0.04)	(0.06)	(0.02)	(0.03)	(0.01)	(0.01)	(0.02)	(0.00)	(0.07)
Iron	-0.02	-0.24**	-0.03	-0.10**	-0.12**	-0.04*	-0.09*	-0.00	-0.08*	0.01	0.08	0.04	-0.02	-0.01	0.02	0.00	0.03	-0.02**	0.89**
	(0.04)	(0.05)	(0.02)	(0.03)	(0.03)	(0.02)	(0.04)	(0.02)	(0.04)	(0.02)	(0.05)	(0.08)	(0.02)	(0.04)	(0.01)	(0.01)	(0.03)	(0.01)	(0.08)
Zinc	-0.03	-0.27**	0.01	-0.03	-0.06*	-0.04**	-0.06*	0.01	-0.06	0.01	0.02	0.04	-0.03	-0.08*	0.02	0.01	0.01	-0.01**	0.82**
	(0.03)	(0.06)	(0.02)	(0.03)	(0.02)	(0.01)	(0.03)	(0.02)	(0.03)	(0.02)	(0.04)	(0.07)	(0.02)	(0.03)	(0.01)	(0.01)	(0.02)	(0.00)	(0.07)
Vitamin A	0.06	-0.22**	-0.10*	-0.06	-0.18**	0.02	0.02	-0.04	-0.06	0.01	0.09	-0.00	0.00	-0.06	-0.14**	-0.01	0.05	-0.03**	1.01**
	(0.05)	(0.05)	(0.04)	(0.04)	(0.04)	(0.03)	(0.04)	(0.02)	(0.06)	(0.03)	(0.07)	(0.09)	(0.04)	(0.05)	(0.03)	(0.01)	(0.03)	(0.01)	(0.10)
Total Folate	-0.03	-0.24**	-0.05	-0.10**	-0.10**	-0.04*	-0.12*	0.00	-0.10*	-0.01	0.09	0.05	0.01	-0.02	0.01	0.00	0.02	-0.01*	0.90**
	(0.04)	(0.05)	(0.03)	(0.04)	(0.03)	(0.02)	(0.05)	(0.02)	(0.04)	(0.02)	(0.06)	(0.09)	(0.03)	(0.04)	(0.02)	(0.01)	(0.03)	(0.01)	(0.09)

Note: This table shows the sample-wide median elasticity of macro- and micro-nutrient intake (quantity demanded) with respect to food prices (columns 1 thru 18) and total household expenditures (the last column). For a list of items in each food group, see Suppl. Table 46.

Suppl. Table 23: Nutrient intake elasticities with respect to food prices and total household expenditures for Q3 consumers (with per capita expenditures between $3.20 and $5.50 per day) in Malawi

	Food Group																		Exp.
	Rice	Maize	Wheat	Cassava	Roots	Sugar	Pulses	Nuts	Vegetables	Fruit	RedMeat	Poultry	Eggs	Dairy	Oils	Tea	SoftDrink	OtherFood	Exp.
Kcal	-0.12**	-0.11	-0.01	-0.07*	-0.05	-0.08**	-0.03	0.02	-0.05	-0.02	0.12*	0.10	-0.01	-0.05	-0.01	0.00	0.04	-0.01*	0.81**
	(0.04)	(0.06)	(0.02)	(0.03)	(0.03)	(0.01)	(0.03)	(0.02)	(0.03)	(0.02)	(0.05)	(0.09)	(0.02)	(0.03)	(0.02)	(0.01)	(0.02)	(0.00)	(0.08)
Protein	-0.08*	-0.13	-0.03	-0.08*	-0.04	-0.03*	-0.03	0.03	-0.04	0.01	0.09	0.12	-0.03	-0.04	0.02	0.01*	0.02	-0.01	0.97**
	(0.04)	(0.07)	(0.02)	(0.03)	(0.04)	(0.02)	(0.05)	(0.02)	(0.03)	(0.02)	(0.07)	(0.11)	(0.02)	(0.03)	(0.02)	(0.01)	(0.03)	(0.00)	(0.11)
Fat	-0.04	-0.10	-0.05	-0.05	-0.04	0.03	0.04	-0.03	0.04	-0.05	0.17	0.05	-0.03	-0.14**	-0.18**	0.02	0.07	-0.04**	1.34**
	(0.05)	(0.07)	(0.04)	(0.04)	(0.05)	(0.03)	(0.06)	(0.03)	(0.05)	(0.03)	(0.10)	(0.15)	(0.04)	(0.04)	(0.04)	(0.01)	(0.04)	(0.01)	(0.14)
Carbohydrates	-0.14**	-0.11	0.00	-0.07	-0.06	-0.11**	-0.04	0.03*	-0.07*	-0.02	0.12*	0.10	-0.01	-0.03	0.02	0.00	0.04	-0.00	0.68**
	(0.04)	(0.07)	(0.02)	(0.04)	(0.03)	(0.02)	(0.03)	(0.02)	(0.03)	(0.02)	(0.05)	(0.08)	(0.02)	(0.03)	(0.02)	(0.01)	(0.03)	(0.00)	(0.08)
Iron	-0.08	-0.13*	-0.02	-0.12**	-0.08*	-0.05**	-0.04	0.03	-0.06	0.01	0.15*	0.12	-0.02	-0.02	0.03	0.00	0.04	-0.01	0.92**
	(0.04)	(0.06)	(0.03)	(0.04)	(0.04)	(0.02)	(0.05)	(0.02)	(0.05)	(0.02)	(0.07)	(0.11)	(0.03)	(0.03)	(0.02)	(0.01)	(0.03)	(0.01)	(0.10)
Zinc	-0.09*	-0.11	0.02	-0.07	-0.04	-0.04**	-0.02	0.04*	-0.04	0.01	0.09	0.14	-0.03	-0.06*	0.03	0.01**	0.02	-0.00	0.79**
	(0.04)	(0.07)	(0.02)	(0.04)	(0.03)	(0.01)	(0.04)	(0.02)	(0.03)	(0.02)	(0.06)	(0.10)	(0.02)	(0.03)	(0.02)	(0.01)	(0.03)	(0.00)	(0.10)
Vitamin A	-0.02	-0.13*	-0.08*	-0.08	-0.13**	0.01	0.04	-0.02	-0.02	-0.00	0.16	0.03	0.00	-0.09*	-0.17**	-0.00	0.07*	-0.04**	1.05**
	(0.05)	(0.06)	(0.04)	(0.05)	(0.05)	(0.03)	(0.05)	(0.02)	(0.07)	(0.03)	(0.08)	(0.11)	(0.04)	(0.04)	(0.04)	(0.01)	(0.04)	(0.01)	(0.11)
Total Folate	-0.09	-0.13	-0.05	-0.12**	-0.06	-0.05**	-0.07	0.02	-0.08	-0.02	0.18*	0.14	0.00	-0.01	0.01	0.00	0.04	-0.01	0.93**
	(0.04)	(0.06)	(0.03)	(0.04)	(0.04)	(0.02)	(0.06)	(0.02)	(0.05)	(0.02)	(0.07)	(0.11)	(0.03)	(0.04)	(0.02)	(0.01)	(0.03)	(0.01)	(0.11)

Note: This table shows the sample-wide median elasticity of macro- and micro-nutrient intake (quantity demanded) with respect to food prices (columns 1 thru 18) and total household expenditures (the last column). For a list of items in each food group, see Suppl. Table 46.

Suppl. Table 24: Nutrient intake elasticities with respect to food prices and total household expenditures for Q4 consumers (with per capita expenditures greater than $5.50 per day) in Malawi

	Food Group																		Exp.
	Rice	Maize	Wheat	Cassava	Roots	Sugar	Pulses	Nuts	Vegetables	Fruit	RedMeat	Poultry	Eggs	Dairy	Oils	Tea	SoftDrink	OtherFood	Exp.
Kcal	-0.28**	0.12	0.01	-0.12*	-0.03	-0.09**	0.03	0.06*	-0.06	-0.04	0.29**	0.23	-0.01	-0.03	-0.01	0.02	0.09*	-0.01	0.84**
	(0.08)	(0.11)	(0.05)	(0.06)	(0.06)	(0.03)	(0.06)	(0.03)	(0.05)	(0.03)	(0.09)	(0.14)	(0.03)	(0.05)	(0.03)	(0.01)	(0.05)	(0.01)	(0.12)
Protein	-0.21**	0.13	-0.01	-0.16**	-0.01	-0.04	0.06	0.07*	-0.01	-0.01	0.24*	0.31	-0.04	-0.01	0.04	0.02*	0.07	0.01	1.03**
	(0.06)	(0.11)	(0.04)	(0.05)	(0.06)	(0.02)	(0.07)	(0.03)	(0.05)	(0.03)	(0.11)	(0.17)	(0.03)	(0.04)	(0.03)	(0.01)	(0.04)	(0.01)	(0.14)
Fat	-0.18*	0.09	-0.00	-0.11	-0.03	0.04	0.10	0.00	0.04	-0.07*	0.34*	0.22	-0.02	-0.11	-0.16**	0.04**	0.15*	-0.04**	1.30**
	(0.08)	(0.08)	(0.05)	(0.06)	(0.07)	(0.04)	(0.09)	(0.04)	(0.07)	(0.04)	(0.13)	(0.19)	(0.04)	(0.06)	(0.06)	(0.01)	(0.06)	(0.01)	(0.17)
Carbohydrates	-0.31**	0.12	0.01	-0.12	-0.03	-0.13**	0.00	0.07*	-0.08	-0.04	0.30**	0.22	-0.00	-0.02	0.02	0.01	0.09	-0.00	0.69**
	(0.09)	(0.12)	(0.05)	(0.07)	(0.06)	(0.03)	(0.06)	(0.03)	(0.06)	(0.04)	(0.10)	(0.13)	(0.04)	(0.05)	(0.03)	(0.01)	(0.05)	(0.01)	(0.12)
Iron	-0.19**	0.10	-0.01	-0.19**	-0.03	-0.08**	0.05	0.08*	-0.01	-0.00	0.29*	0.29	-0.03	-0.02	0.03	0.00	0.07	0.00	1.03**
	(0.07)	(0.10)	(0.05)	(0.06)	(0.07)	(0.03)	(0.07)	(0.04)	(0.07)	(0.04)	(0.12)	(0.17)	(0.03)	(0.05)	(0.03)	(0.01)	(0.05)	(0.01)	(0.14)
Zinc	-0.24**	0.18	0.03	-0.15*	-0.01	-0.05*	0.07	0.08*	-0.02	0.00	0.23*	0.34*	-0.04	-0.00	0.04	0.03**	0.06	0.01	0.82**
	(0.07)	(0.12)	(0.05)	(0.06)	(0.06)	(0.02)	(0.07)	(0.03)	(0.06)	(0.04)	(0.11)	(0.16)	(0.03)	(0.05)	(0.03)	(0.01)	(0.05)	(0.01)	(0.14)
Vitamin A	-0.16*	0.03	-0.05	-0.13*	-0.08	-0.01	0.08	0.00	0.02	-0.03	0.33**	0.11	0.01	-0.11	-0.19**	0.02	0.14*	-0.05**	1.08**
	(0.08)	(0.08)	(0.06)	(0.06)	(0.08)	(0.04)	(0.07)	(0.04)	(0.09)	(0.04)	(0.11)	(0.16)	(0.05)	(0.06)	(0.06)	(0.02)	(0.06)	(0.02)	(0.15)
Total Folate	-0.20**	0.11	-0.04	-0.17*	-0.00	-0.08**	0.01	0.06	-0.02	-0.05	0.36**	0.30	-0.01	0.00	0.02	0.00	0.06	0.00	1.07**
	(0.07)	(0.10)	(0.05)	(0.07)	(0.06)	(0.03)	(0.09)	(0.04)	(0.08)	(0.04)	(0.12)	(0.16)	(0.04)	(0.05)	(0.03)	(0.01)	(0.05)	(0.01)	(0.14)

Note: This table shows the sample-wide median elasticity of macro- and micro-nutrient intake (quantity demanded) with respect to food prices (columns 1 thru 18) and total household expenditures (the last column). For a list of items in each food group, see Suppl. Table 46.

Suppl. Table 25: Nutrient intake elasticities with respect to food prices and total household expenditures for Q1 consumers (with per capita expenditures less than \$1.90 per day) in Niger

	Food Group																			Exp.
	Rice	Millet	Wheat	Cassava	Roots	Sugar	Pulses	Nuts	Vegetables	Fruit	RedMeat	Poultry	Eggs	Fish	Dairy	Oils	Coffee	SoftDrink	OtherFood	
Kcal	-0.00	-0.86**	-0.21**	0.04	0.08	-0.06	-0.10	-0.03	0.00	-0.00	-0.04	0.06	-0.03	0.03	-0.09	-0.02	-0.01	0.01	-0.14**	1.16**
	(0.14)	(0.20)	(0.05)	(0.03)	(0.05)	(0.07)	(0.08)	(0.05)	(0.05)	(0.03)	(0.05)	(0.13)	(0.06)	(0.03)	(0.07)	(0.05)	(0.03)	(0.05)	(0.05)	(0.25)
Protein	0.03	-0.84**	-0.22**	0.02	0.05	-0.03	-0.23*	-0.03	0.06	0.01	-0.04	0.05	-0.12	0.06	-0.09	0.05	-0.02	-0.02	-0.23**	1.23**
	(0.17)	(0.23)	(0.05)	(0.03)	(0.05)	(0.07)	(0.11)	(0.05)	(0.05)	(0.03)	(0.05)	(0.14)	(0.07)	(0.03)	(0.06)	(0.07)	(0.03)	(0.05)	(0.06)	(0.24)
Fat	0.28	-0.64**	-0.16**	0.05	0.01	-0.05	-0.08	-0.07	-0.07	-0.07*	-0.03	0.10	-0.09	-0.01	-0.06	-0.26**	0.01	0.02	-0.01	1.21**
	(0.16)	(0.19)	(0.04)	(0.03)	(0.04)	(0.05)	(0.09)	(0.04)	(0.05)	(0.03)	(0.04)	(0.09)	(0.05)	(0.02)	(0.06)	(0.06)	(0.03)	(0.04)	(0.06)	(0.21)
Carbohydrates	-0.08	-0.92**	-0.21**	0.04	0.10	-0.06	-0.07	-0.02	0.01	0.01	-0.04	0.05	-0.00	0.04	-0.09	0.02	-0.01	0.01	-0.16**	1.12**
	(0.16)	(0.23)	(0.06)	(0.03)	(0.06)	(0.07)	(0.09)	(0.06)	(0.06)	(0.03)	(0.06)	(0.14)	(0.07)	(0.03)	(0.08)	(0.06)	(0.03)	(0.05)	(0.06)	(0.29)
Iron	0.04	-0.98**	-0.14**	0.03	0.06	-0.04	-0.07	-0.01	0.10	0.01	-0.05	0.07	-0.00	0.03	-0.09	0.12*	-0.04	-0.02	-0.42**	0.86**
	(0.17)	(0.21)	(0.04)	(0.03)	(0.05)	(0.06)	(0.07)	(0.04)	(0.05)	(0.03)	(0.05)	(0.14)	(0.05)	(0.02)	(0.06)	(0.06)	(0.02)	(0.05)	(0.05)	(0.23)
Zinc	-0.05	-0.93**	-0.17**	0.03	0.10	-0.04	-0.10	-0.02	0.02	0.00	-0.06	0.06	-0.05	0.03	-0.12	0.03	-0.03	0.00	-0.19**	1.04**
	(0.18)	(0.24)	(0.05)	(0.03)	(0.05)	(0.07)	(0.10)	(0.05)	(0.05)	(0.03)	(0.06)	(0.16)	(0.06)	(0.03)	(0.07)	(0.07)	(0.03)	(0.05)	(0.06)	(0.27)
Vitamin A	0.67**	-0.49	-0.05	0.09*	-0.10	-0.08	0.04	-0.02	-0.10	-0.06	0.05	-0.02	-0.08	-0.03	0.08	-0.24*	0.03	-0.01	-0.11	1.13**
	(0.24)	(0.30)	(0.05)	(0.04)	(0.05)	(0.08)	(0.11)	(0.06)	(0.09)	(0.04)	(0.05)	(0.12)	(0.06)	(0.04)	(0.07)	(0.10)	(0.04)	(0.06)	(0.07)	(0.26)
Total Folate	0.04	-0.82**	-0.16**	0.00	0.08	-0.03	-0.24	-0.04	-0.00	-0.00	-0.05	0.09	-0.16	0.04	-0.11	0.01	-0.04	-0.03	-0.16*	1.06**
	(0.21)	(0.29)	(0.06)	(0.04)	(0.06)	(0.08)	(0.14)	(0.06)	(0.06)	(0.04)	(0.06)	(0.17)	(0.08)	(0.04)	(0.07)	(0.08)	(0.03)	(0.06)	(0.07)	(0.28)

Note: This table shows the sample-wide median elasticity of macro- and micro-nutrient intake (quantity demanded) with respect to food prices (columns 1 thru 19) and total household expenditures (the last column). For a list of items in each food group, see Suppl. Table 47.

Suppl. Table 26: Nutrient intake elasticities with respect to food prices and total household expenditures for Q2 consumers (with per capita expenditures between $1.90 and $3.20 per day) in Niger

	Food Group																			Exp.
	Rice	Millet	Wheat	Cassava	Roots	Sugar	Pulses	Nuts	Vegetables	Fruit	RedMeat	Poultry	Eggs	Fish	Dairy	Oils	Coffee	SoftDrink	OtherFood	
Kcal	-0.18	-0.84**	-0.25**	0.04	0.02	-0.00	-0.11*	-0.01	-0.08	0.04	-0.00	0.06	-0.00	0.02	-0.08	-0.03	0.00	-0.01	-0.07	0.99**
	(0.15)	(0.17)	(0.03)	(0.02)	(0.03)	(0.05)	(0.05)	(0.04)	(0.05)	(0.02)	(0.04)	(0.11)	(0.04)	(0.02)	(0.05)	(0.05)	(0.02)	(0.03)	(0.04)	(0.20)
Protein	-0.16	-0.80**	-0.26**	0.04	-0.02	0.00	-0.22**	-0.03	-0.04	0.05*	-0.00	0.06	-0.05	0.02	-0.09	0.01	-0.00	-0.04	-0.13**	1.09**
	(0.14)	(0.18)	(0.04)	(0.03)	(0.04)	(0.06)	(0.06)	(0.04)	(0.05)	(0.02)	(0.04)	(0.12)	(0.05)	(0.02)	(0.05)	(0.06)	(0.02)	(0.04)	(0.03)	(0.19)
Fat	0.32	-0.65**	-0.20**	0.02	-0.04	0.03	-0.10	-0.06	0.00	-0.04	0.01	0.09	-0.09*	-0.00	-0.09*	-0.26**	-0.01	0.00	-0.10**	1.15**
	(0.18)	(0.19)	(0.03)	(0.02)	(0.03)	(0.04)	(0.05)	(0.03)	(0.04)	(0.03)	(0.04)	(0.08)	(0.04)	(0.02)	(0.04)	(0.05)	(0.02)	(0.03)	(0.03)	(0.18)
Carbohydrates	-0.32	-0.90**	-0.26**	0.04	0.05	-0.01	-0.09	0.00	-0.11*	0.05*	-0.00	0.05	0.03	0.02	-0.08	0.02	0.00	-0.01	-0.06	0.92**
	(0.18)	(0.20)	(0.04)	(0.03)	(0.04)	(0.06)	(0.06)	(0.04)	(0.05)	(0.02)	(0.05)	(0.12)	(0.05)	(0.02)	(0.06)	(0.07)	(0.02)	(0.04)	(0.04)	(0.22)
Iron	0.05	-0.95**	-0.14**	0.03	0.00	-0.01	-0.05	-0.01	0.07	0.05*	0.01	0.06	-0.01	0.01	-0.06	0.03	-0.02	-0.01	-0.39**	0.80**
	(0.19)	(0.20)	(0.04)	(0.02)	(0.04)	(0.06)	(0.05)	(0.04)	(0.06)	(0.02)	(0.05)	(0.13)	(0.05)	(0.02)	(0.05)	(0.07)	(0.02)	(0.04)	(0.04)	(0.21)
Zinc	-0.29	-0.94**	-0.21**	0.04	0.03	0.01	-0.12	-0.02	-0.12*	0.05*	-0.04	0.09	0.00	0.01	-0.10	0.01	-0.01	-0.02	-0.08	0.91**
	(0.19)	(0.22)	(0.04)	(0.03)	(0.04)	(0.07)	(0.07)	(0.05)	(0.06)	(0.03)	(0.05)	(0.15)	(0.05)	(0.02)	(0.06)	(0.07)	(0.02)	(0.04)	(0.04)	(0.24)
Vitamin A	0.84**	-0.78*	-0.09	0.06	-0.08	-0.04	-0.01	-0.05	0.10	-0.08**	0.07	-0.10	-0.21**	-0.00	-0.01	-0.22*	0.01	-0.02	-0.13	1.40**
	(0.26)	(0.30)	(0.05)	(0.04)	(0.05)	(0.07)	(0.07)	(0.05)	(0.09)	(0.03)	(0.05)	(0.12)	(0.06)	(0.03)	(0.06)	(0.09)	(0.03)	(0.05)	(0.07)	(0.20)
Total Folate	-0.21	-0.87**	-0.14**	0.03	0.01	-0.01	-0.26**	-0.04	-0.15**	0.05	-0.02	0.09	-0.09	0.02	-0.10	0.00	-0.01	-0.07	-0.02	0.85**
	(0.18)	(0.25)	(0.05)	(0.04)	(0.05)	(0.07)	(0.09)	(0.06)	(0.06)	(0.03)	(0.06)	(0.18)	(0.06)	(0.03)	(0.06)	(0.07)	(0.02)	(0.05)	(0.05)	(0.25)

Note: This table shows the sample-wide median elasticity of macro- and micro-nutrient intake (quantity demanded) with respect to food prices (columns 1 thru 19) and total household expenditures (the last column). For a list of items in each food group, see Suppl. Table 47.

Suppl. Table 27: Nutrient intake elasticities with respect to food prices and total household expenditures for Q3 consumers (with per capita expenditures between $3.20 and $5.50 per day) in Niger

	Food Group																			Exp.
	Rice	Millet	Wheat	Cassava	Roots	Sugar	Pulses	Nuts	Vegetables	Fruit	RedMeat	Poultry	Eggs	Fish	Dairy	Oils	Coffee	SoftDrink	OtherFood	
Kcal	-0.37	-0.85**	-0.27**	0.04	-0.02	0.04	-0.13**	-0.04	-0.17*	0.07*	0.02	0.08	0.03	-0.00	-0.09	-0.01	0.00	-0.04	0.01	0.87**
	(0.22)	(0.22)	(0.05)	(0.03)	(0.04)	(0.09)	(0.05)	(0.06)	(0.08)	(0.03)	(0.07)	(0.19)	(0.07)	(0.03)	(0.07)	(0.07)	(0.02)	(0.05)	(0.04)	(0.27)
Protein	-0.28	-0.67**	-0.19**	0.04	-0.07	0.05	-0.20**	-0.14	-0.15	0.05	-0.00	0.11	-0.00	-0.03	-0.10	-0.01	-0.02	-0.05	-0.02	0.88*
	(0.18)	(0.22)	(0.07)	(0.04)	(0.07)	(0.13)	(0.06)	(0.12)	(0.08)	(0.03)	(0.12)	(0.31)	(0.10)	(0.05)	(0.10)	(0.07)	(0.03)	(0.08)	(0.04)	(0.39)
Fat	0.18	-0.61**	-0.18**	0.00	-0.08	0.10	-0.12**	-0.09	0.00	-0.01	-0.04	0.13	-0.04	-0.00	-0.13*	-0.19**	-0.03	-0.01	-0.12**	1.06**
	(0.19)	(0.18)	(0.05)	(0.03)	(0.04)	(0.08)	(0.05)	(0.07)	(0.08)	(0.03)	(0.08)	(0.17)	(0.07)	(0.03)	(0.06)	(0.05)	(0.02)	(0.05)	(0.03)	(0.26)
Carbohydrates	-0.52*	-0.95**	-0.31**	0.05	0.00	0.03	-0.11*	0.00	-0.23**	0.09*	0.03	0.06	0.06	0.00	-0.08	0.03	0.02	-0.05	0.04	0.81**
	(0.26)	(0.26)	(0.05)	(0.04)	(0.04)	(0.09)	(0.05)	(0.05)	(0.08)	(0.04)	(0.07)	(0.17)	(0.07)	(0.03)	(0.07)	(0.09)	(0.02)	(0.05)	(0.05)	(0.26)
Iron	-0.10	-0.94**	-0.16**	0.04	-0.03	0.03	-0.06	-0.03	-0.05	0.08*	0.05	0.09	-0.01	-0.01	-0.07	-0.06	0.00	-0.01	-0.27**	0.71*
	(0.28)	(0.26)	(0.05)	(0.03)	(0.05)	(0.10)	(0.06)	(0.06)	(0.11)	(0.04)	(0.07)	(0.20)	(0.08)	(0.03)	(0.07)	(0.10)	(0.02)	(0.05)	(0.04)	(0.29)
Zinc	-0.46	-0.91**	-0.19**	0.06	-0.02	0.07	-0.13*	-0.10	-0.24*	0.07	-0.04	0.14	0.05	-0.01	-0.10	0.01	-0.01	-0.04	0.02	0.76
	(0.24)	(0.27)	(0.07)	(0.04)	(0.07)	(0.13)	(0.06)	(0.11)	(0.10)	(0.04)	(0.11)	(0.31)	(0.10)	(0.05)	(0.10)	(0.09)	(0.03)	(0.07)	(0.05)	(0.40)
Vitamin A	0.64**	-1.13**	-0.07	0.08	-0.06	-0.01	-0.07	-0.08	0.11	-0.07**	0.05	-0.16	-0.30**	0.04	-0.15	-0.05	-0.00	-0.05	-0.01	1.42**
	(0.23)	(0.34)	(0.05)	(0.05)	(0.05)	(0.09)	(0.06)	(0.06)	(0.11)	(0.03)	(0.07)	(0.15)	(0.08)	(0.03)	(0.08)	(0.09)	(0.02)	(0.05)	(0.11)	(0.20)
Total Folate	-0.41	-0.93**	-0.11	0.04	-0.04	0.03	-0.27**	-0.13	-0.26**	0.06	-0.00	0.13	-0.03	-0.01	-0.11	0.03	-0.01	-0.10	0.09	0.69
	(0.22)	(0.31)	(0.07)	(0.05)	(0.08)	(0.15)	(0.07)	(0.12)	(0.09)	(0.04)	(0.13)	(0.34)	(0.11)	(0.05)	(0.10)	(0.09)	(0.03)	(0.08)	(0.06)	(0.42)

Note: This table shows the sample-wide median elasticity of macro- and micro-nutrient intake (quantity demanded) with respect to food prices (columns 1 thru 19) and total household expenditures (the last column). For a list of items in each food group, see Suppl. Table 47.

Suppl. Table 28: Nutrient intake elasticities with respect to food prices and total household expenditures for Q4 consumers (with per capita expenditures greater than $5.50 per day) in Niger

	Food Group																			Exp.
	Rice	Millet	Wheat	Cassava	Roots	Sugar	Pulses	Nuts	Vegetables	Fruit	RedMeat	Poultry	Eggs	Fish	Dairy	Oils	Coffee	SoftDrink	OtherFood	
Kcal	-0.52	-0.73	-0.29	0.04	-0.02	0.02	-0.23	0.07	-0.26	0.05	0.09	0.17	0.16	-0.01	-0.02	0.05	-0.01	-0.14	0.08	0.80
	(0.60)	(1.10)	(0.33)	(0.15)	(0.45)	(0.67)	(0.33)	(0.51)	(0.31)	(0.26)	(0.55)	(1.38)	(0.64)	(0.19)	(0.65)	(0.31)	(0.14)	(0.35)	(0.19)	(1.56)
Protein	-0.63*	-0.79*	-0.29**	0.06	-0.19*	0.10	-0.32**	-0.05	-0.31**	0.12*	-0.05	0.16	0.06	-0.07	-0.11	-0.01	0.01	-0.14	0.14*	0.94**
	(0.32)	(0.31)	(0.08)	(0.05)	(0.08)	(0.15)	(0.09)	(0.11)	(0.12)	(0.05)	(0.15)	(0.30)	(0.13)	(0.06)	(0.11)	(0.11)	(0.03)	(0.09)	(0.07)	(0.32)
Fat	0.48	-0.57	-0.22**	-0.06	-0.15**	0.19	-0.22**	-0.02	0.17	-0.02	-0.09	0.10	-0.09	0.00	-0.17*	-0.25	-0.04	-0.07	-0.23**	1.13**
	(0.38)	(0.33)	(0.07)	(0.05)	(0.05)	(0.10)	(0.08)	(0.06)	(0.13)	(0.05)	(0.11)	(0.15)	(0.10)	(0.04)	(0.07)	(0.13)	(0.03)	(0.07)	(0.07)	(0.21)
Carbohydrates	-0.80	-0.75	-0.31	0.07	0.06	-0.05	-0.21	0.13	-0.39	0.05	0.17	0.21	0.25	0.00	0.05	0.16	-0.01	-0.17	0.16	0.66
	(0.77)	(1.42)	(0.42)	(0.20)	(0.59)	(0.87)	(0.42)	(0.66)	(0.40)	(0.34)	(0.70)	(1.78)	(0.83)	(0.24)	(0.84)	(0.41)	(0.18)	(0.45)	(0.25)	(2.01)
Iron	-0.65	-1.08*	-0.19	0.07	-0.11	0.11	-0.18	-0.01	-0.38	0.20*	0.11	0.24	0.11	-0.04	-0.08	-0.14	0.04	-0.08	-0.07	0.53
	(0.51)	(0.47)	(0.11)	(0.07)	(0.09)	(0.19)	(0.11)	(0.11)	(0.22)	(0.09)	(0.16)	(0.35)	(0.18)	(0.07)	(0.13)	(0.19)	(0.04)	(0.11)	(0.08)	(0.42)
Zinc	-0.82*	-1.08**	-0.27**	0.08	-0.13	0.13	-0.23*	-0.04	-0.45**	0.15*	-0.08	0.21	0.13	-0.05	-0.09	0.02	0.01	-0.12	0.16	0.80*
	(0.42)	(0.40)	(0.10)	(0.06)	(0.08)	(0.17)	(0.10)	(0.11)	(0.16)	(0.07)	(0.16)	(0.35)	(0.16)	(0.06)	(0.12)	(0.15)	(0.03)	(0.10)	(0.08)	(0.38)
Vitamin A	1.11*	-0.94*	-0.14	-0.04	-0.07	0.11	-0.11	-0.06	0.47**	-0.11*	0.08	-0.17	-0.38**	0.06	-0.23**	-0.19	-0.04	-0.02	-0.27*	1.35**
	(0.44)	(0.46)	(0.09)	(0.06)	(0.06)	(0.12)	(0.09)	(0.07)	(0.14)	(0.05)	(0.10)	(0.16)	(0.10)	(0.04)	(0.08)	(0.19)	(0.03)	(0.07)	(0.12)	(0.16)
Total Folate	-0.85*	-0.92*	-0.14	0.02	-0.25	0.06	-0.62**	-0.08	-0.45*	0.21*	0.09	0.31	0.08	-0.07	-0.13	-0.02	0.05	-0.27	0.17	0.55
	(0.41)	(0.46)	(0.12)	(0.08)	(0.13)	(0.23)	(0.16)	(0.15)	(0.18)	(0.09)	(0.22)	(0.42)	(0.20)	(0.09)	(0.17)	(0.16)	(0.05)	(0.15)	(0.10)	(0.45)

Note: This table shows the sample-wide median elasticity of macro- and micro-nutrient intake (quantity demanded) with respect to food prices (columns 1 thru 19) and total household expenditures (the last column). For a list of items in each food group, see Suppl. Table 47.

Suppl. Table 29: Nutrient intake elasticities with respect to food prices and total household expenditures for Q1 consumers (with per capita expenditures less than $1.90 per day) in Uganda

	Food Group																			Exp.
	Rice	Maize	Wheat	Cassava	Roots	Sugar	Pulses	Nuts	Vegetables	Fruit	RedMeat	Poultry	Eggs	Fish	Dairy	Fats	Coffee	SoftDrink	OtherFood	
Kcal	0.12**	-0.12**	-0.16**	-0.15**	-0.22**	-0.00	-0.15**	-0.07**	0.00	0.01	0.02	0.17*	0.02	0.04	0.05	-0.11**	-0.00	-0.04	-0.01**	1.03**
	(0.04)	(0.04)	(0.03)	(0.04)	(0.04)	(0.03)	(0.03)	(0.02)	(0.03)	(0.02)	(0.06)	(0.07)	(0.03)	(0.03)	(0.03)	(0.03)	(0.01)	(0.05)	(0.00)	(0.08)
Protein	0.01	-0.09**	-0.16**	0.00	-0.07*	0.01	-0.28**	-0.12**	-0.06**	0.03	-0.01	0.16*	0.05*	-0.21**	0.01	0.01	0.00	0.01	-0.01**	1.11**
	(0.03)	(0.03)	(0.02)	(0.04)	(0.03)	(0.01)	(0.02)	(0.01)	(0.02)	(0.02)	(0.04)	(0.07)	(0.02)	(0.03)	(0.03)	(0.01)	(0.00)	(0.04)	(0.00)	(0.07)
Fat	0.11	0.05	-0.14**	-0.13	-0.26**	-0.09	0.00	-0.14**	0.06	0.05	0.09	0.44**	0.02	0.04	0.21**	-0.32**	-0.01	-0.04	-0.03**	1.08**
	(0.09)	(0.08)	(0.05)	(0.07)	(0.09)	(0.08)	(0.07)	(0.05)	(0.09)	(0.05)	(0.17)	(0.17)	(0.07)	(0.06)	(0.07)	(0.08)	(0.02)	(0.12)	(0.01)	(0.20)
Carbohydrates	0.12**	-0.22**	-0.16**	-0.19**	-0.21**	0.03*	-0.20**	-0.03**	-0.06**	-0.02	0.00	0.01	0.02	0.08**	-0.03	-0.01	0.00	-0.03	-0.01**	0.95**
	(0.03)	(0.03)	(0.03)	(0.04)	(0.03)	(0.01)	(0.02)	(0.01)	(0.02)	(0.02)	(0.04)	(0.05)	(0.02)	(0.03)	(0.02)	(0.01)	(0.00)	(0.04)	(0.00)	(0.06)
Iron	-0.06	-0.07*	-0.09**	-0.13**	-0.13**	-0.05*	-0.21**	-0.08**	-0.35**	-0.02	0.06	0.10*	0.04*	-0.05	0.01	0.01	-0.00	0.06	-0.02**	0.85**
	(0.04)	(0.03)	(0.02)	(0.03)	(0.03)	(0.03)	(0.03)	(0.01)	(0.05)	(0.02)	(0.05)	(0.04)	(0.02)	(0.03)	(0.02)	(0.01)	(0.01)	(0.05)	(0.00)	(0.06)
Zinc	0.05	-0.15**	-0.13**	-0.10**	-0.11**	0.01	-0.29**	-0.15**	-0.03	-0.03*	-0.10**	0.02	0.06**	-0.00	-0.04*	0.01	0.00	-0.01	-0.01**	1.09**
	(0.02)	(0.02)	(0.02)	(0.03)	(0.02)	(0.01)	(0.02)	(0.01)	(0.02)	(0.01)	(0.04)	(0.03)	(0.02)	(0.02)	(0.02)	(0.01)	(0.00)	(0.03)	(0.00)	(0.05)
Vitamin A	0.18	0.10	-0.13*	-0.15	-0.38**	-0.14	0.01	0.09	-0.02	0.08	0.16	0.54**	-0.02	0.04	0.25**	-0.41**	-0.01	-0.04	-0.03**	1.02**
	(0.10)	(0.09)	(0.06)	(0.09)	(0.11)	(0.09)	(0.09)	(0.05)	(0.10)	(0.06)	(0.20)	(0.20)	(0.08)	(0.07)	(0.08)	(0.10)	(0.02)	(0.14)	(0.01)	(0.24)
Total Folate	0.01	-0.08**	-0.11**	-0.07*	-0.16**	0.04**	-0.40**	-0.21**	-0.09**	-0.04**	0.01	0.13**	0.05**	0.02	0.01	0.02	0.00	-0.04	-0.01**	0.90**
	(0.03)	(0.03)	(0.02)	(0.03)	(0.02)	(0.02)	(0.02)	(0.01)	(0.03)	(0.02)	(0.04)	(0.04)	(0.02)	(0.02)	(0.02)	(0.01)	(0.00)	(0.03)	(0.00)	(0.05)

Note: This table shows the sample-wide median elasticity of macro- and micro-nutrient intake (quantity demanded) with respect to food prices (columns 1 thru 19) and total household expenditures (the last column). For a list of items in each food group, see Suppl. Table 48.

Suppl. Table 30: Nutrient intake elasticities with respect to food prices and total household expenditures for Q2 consumers (with per capita expenditures between $1.90 and $3.20 per day) in Uganda

	Food Group																			Exp.
	Rice	Maize	Wheat	Cassava	Roots	Sugar	Pulses	Nuts	Vegetables	Fruit	RedMeat	Poultry	Eggs	Fish	Dairy	Fats	Coffee	SoftDrink	OtherFood	
Kcal	0.04	-0.07*	-0.08**	-0.12**	-0.13**	0.01	-0.17**	-0.12**	0.04	0.02	0.02	0.11*	0.03	-0.00	-0.00	-0.13**	0.01	0.04	-0.02**	0.92**
	(0.03)	(0.03)	(0.02)	(0.04)	(0.04)	(0.02)	(0.02)	(0.01)	(0.03)	(0.02)	(0.05)	(0.05)	(0.02)	(0.02)	(0.02)	(0.03)	(0.01)	(0.05)	(0.00)	(0.07)
Protein	-0.04	-0.05	-0.10**	-0.02	-0.02	-0.01	-0.26**	-0.17**	-0.02	0.03*	0.05	0.09*	0.05**	-0.22**	-0.01	-0.01	0.00	0.04	-0.00	0.98**
	(0.02)	(0.03)	(0.02)	(0.03)	(0.03)	(0.01)	(0.02)	(0.01)	(0.02)	(0.01)	(0.03)	(0.04)	(0.02)	(0.03)	(0.02)	(0.01)	(0.00)	(0.03)	(0.00)	(0.05)
Fat	0.05	0.08	-0.03	-0.14*	-0.14*	0.03	-0.13*	-0.23**	0.16*	0.03	0.04	0.23*	0.01	-0.01	0.07	-0.32**	0.02	0.07	-0.04**	0.99**
	(0.06)	(0.06)	(0.04)	(0.06)	(0.07)	(0.06)	(0.05)	(0.03)	(0.07)	(0.04)	(0.13)	(0.10)	(0.05)	(0.04)	(0.05)	(0.07)	(0.02)	(0.09)	(0.01)	(0.13)
Carbohydrates	0.04*	-0.17**	-0.11**	-0.12**	-0.16**	-0.02	-0.16**	-0.04**	-0.07**	0.01	0.01	0.02	0.03	0.05*	-0.05*	-0.01	-0.00	0.04	-0.01**	0.85**
	(0.02)	(0.03)	(0.02)	(0.04)	(0.03)	(0.01)	(0.02)	(0.01)	(0.02)	(0.02)	(0.04)	(0.04)	(0.02)	(0.02)	(0.02)	(0.01)	(0.00)	(0.03)	(0.00)	(0.05)
Iron	-0.05*	-0.06**	-0.09**	-0.09**	-0.10**	-0.04*	-0.28**	-0.12**	-0.25**	0.01	0.12**	0.07*	0.06**	-0.03*	0.01	0.01	0.00	0.11**	-0.01**	0.83**
	(0.02)	(0.02)	(0.01)	(0.02)	(0.02)	(0.02)	(0.02)	(0.01)	(0.04)	(0.01)	(0.04)	(0.03)	(0.01)	(0.02)	(0.02)	(0.01)	(0.00)	(0.03)	(0.00)	(0.04)
Zinc	-0.03	-0.10**	-0.08**	-0.07**	-0.06*	-0.00	-0.25**	-0.19**	-0.03	-0.00	0.00	0.03	0.05**	-0.04**	-0.03	-0.01	0.00	0.04	-0.00**	0.93**
	(0.02)	(0.02)	(0.02)	(0.02)	(0.02)	(0.01)	(0.01)	(0.01)	(0.02)	(0.01)	(0.03)	(0.03)	(0.01)	(0.02)	(0.01)	(0.01)	(0.00)	(0.03)	(0.00)	(0.04)
Vitamin A	0.11	0.15*	-0.02	-0.15*	-0.24**	0.02	-0.15*	-0.01	0.11	0.03	0.05	0.30*	-0.02	0.01	0.07	-0.41**	0.02	0.10	-0.05**	0.96**
	(0.07)	(0.07)	(0.05)	(0.07)	(0.08)	(0.08)	(0.06)	(0.04)	(0.09)	(0.05)	(0.16)	(0.12)	(0.07)	(0.05)	(0.06)	(0.09)	(0.02)	(0.11)	(0.01)	(0.17)
Total Folate	-0.04	-0.04	-0.06**	-0.07**	-0.10**	0.01	-0.37**	-0.27**	-0.08**	-0.00	0.11**	0.07*	0.07**	-0.02	0.02	-0.01	0.00	0.05	-0.01**	0.82**
	(0.02)	(0.02)	(0.02)	(0.03)	(0.02)	(0.01)	(0.02)	(0.01)	(0.02)	(0.01)	(0.04)	(0.03)	(0.02)	(0.02)	(0.02)	(0.01)	(0.00)	(0.03)	(0.00)	(0.04)

Note: This table shows the sample-wide median elasticity of macro- and micro-nutrient intake (quantity demanded) with respect to food prices (columns 1 thru 19) and total household expenditures (the last column). For a list of items in each food group, see Suppl. Table 48.

Suppl. Table 31: Nutrient intake elasticities with respect to food prices and total household expenditures for Q3 consumers (with per capita expenditures between $3.20 and $5.50 per day) in Uganda

	Food Group																			Exp.
	Rice	Maize	Wheat	Cassava	Roots	Sugar	Pulses	Nuts	Vegetables	Fruit	RedMeat	Poultry	Eggs	Fish	Dairy	Fats	Coffee	SoftDrink	OtherFood	
Kcal	-0.01	-0.02	-0.02	-0.11**	-0.08	0.02	-0.19**	-0.17**	0.08*	0.02	0.01	0.05	0.02	-0.03	-0.04	-0.13**	0.01	0.09	-0.02**	0.79**
	(0.03)	(0.03)	(0.03)	(0.04)	(0.04)	(0.03)	(0.03)	(0.02)	(0.03)	(0.02)	(0.06)	(0.06)	(0.03)	(0.03)	(0.03)	(0.03)	(0.01)	(0.05)	(0.00)	(0.07)
Protein	-0.08**	-0.01	-0.05*	-0.06	0.01	-0.04*	-0.23**	-0.21**	0.01	0.04*	0.06	0.03	0.04*	-0.22**	-0.02	-0.03**	-0.00	0.05	0.00	0.91**
	(0.03)	(0.03)	(0.03)	(0.04)	(0.03)	(0.02)	(0.02)	(0.01)	(0.02)	(0.02)	(0.04)	(0.04)	(0.02)	(0.03)	(0.02)	(0.01)	(0.00)	(0.03)	(0.00)	(0.05)
Fat	0.01	0.10	0.06	-0.15*	-0.05	0.12	-0.23**	-0.30**	0.25**	0.02	0.01	0.11	0.01	-0.03	-0.01	-0.30**	0.03*	0.14	-0.05**	0.76**
	(0.06)	(0.06)	(0.04)	(0.06)	(0.07)	(0.06)	(0.06)	(0.03)	(0.08)	(0.05)	(0.13)	(0.08)	(0.05)	(0.05)	(0.05)	(0.07)	(0.02)	(0.09)	(0.01)	(0.12)
Carbohydrates	-0.01	-0.13**	-0.07**	-0.09*	-0.13**	-0.05**	-0.13**	-0.05**	-0.08**	0.02	0.01	-0.00	0.04	0.03	-0.06**	-0.00	-0.00	0.07	-0.01**	0.79**
	(0.02)	(0.03)	(0.02)	(0.04)	(0.04)	(0.01)	(0.02)	(0.01)	(0.02)	(0.02)	(0.04)	(0.05)	(0.02)	(0.02)	(0.02)	(0.01)	(0.00)	(0.04)	(0.00)	(0.06)
Iron	-0.08**	-0.03	-0.06**	-0.08**	-0.07**	-0.05**	-0.27**	-0.17**	-0.20**	0.03	0.15**	0.03	0.07**	-0.04*	0.01	-0.01	0.00	0.15**	-0.01**	0.81**
	(0.02)	(0.02)	(0.02)	(0.02)	(0.03)	(0.01)	(0.02)	(0.01)	(0.03)	(0.01)	(0.04)	(0.03)	(0.02)	(0.02)	(0.02)	(0.01)	(0.00)	(0.03)	(0.00)	(0.04)
Zinc	-0.07**	-0.06**	-0.04*	-0.07*	-0.04	-0.03*	-0.20**	-0.22**	-0.01	0.01	0.01	0.01	0.05*	-0.06**	-0.02	-0.03**	-0.00	0.06	-0.00	0.90**
	(0.02)	(0.02)	(0.02)	(0.03)	(0.03)	(0.01)	(0.02)	(0.01)	(0.02)	(0.02)	(0.04)	(0.03)	(0.02)	(0.02)	(0.02)	(0.01)	(0.00)	(0.03)	(0.00)	(0.04)
Vitamin A	0.07	0.19**	0.07	-0.15*	-0.12	0.14	-0.26**	-0.08	0.20*	0.00	-0.02	0.15	-0.02	-0.01	-0.05	-0.38**	0.05*	0.20	-0.07**	0.69**
	(0.08)	(0.07)	(0.05)	(0.07)	(0.08)	(0.08)	(0.07)	(0.04)	(0.10)	(0.06)	(0.17)	(0.11)	(0.07)	(0.06)	(0.06)	(0.09)	(0.02)	(0.11)	(0.01)	(0.15)
Total Folate	-0.09**	-0.01	-0.02	-0.08**	-0.06*	-0.02	-0.32**	-0.34**	-0.08**	0.03	0.18**	0.02	0.08**	-0.05*	0.03	-0.04**	-0.00	0.10**	-0.00	0.80**
	(0.03)	(0.02)	(0.02)	(0.03)	(0.03)	(0.02)	(0.02)	(0.01)	(0.03)	(0.02)	(0.04)	(0.03)	(0.02)	(0.02)	(0.02)	(0.01)	(0.00)	(0.04)	(0.00)	(0.05)

Note: This table shows the sample-wide median elasticity of macro- and micro-nutrient intake (quantity demanded) with respect to food prices (columns 1 thru 19) and total household expenditures (the last column). For a list of items in each food group, see Suppl. Table 48.

Suppl. Table 32: Nutrient intake elasticities with respect to food prices and total household expenditures for Q4 consumers (with per capita expenditures greater than $5.50 per day) in Uganda

	Food Group																			Exp.
	Rice	Maize	Wheat	Cassava	Roots	Sugar	Pulses	Nuts	Vegetables	Fruit	RedMeat	Poultry	Eggs	Fish	Dairy	Fats	Coffee	SoftDrink	OtherFood	
Kcal	-0.05	0.03	0.06	-0.11	-0.02	0.03	-0.23**	-0.22**	0.13**	0.02	-0.03	-0.06	0.02	-0.07	-0.09*	-0.12**	0.02*	0.13	-0.03**	0.64**
	(0.05)	(0.05)	(0.04)	(0.06)	(0.06)	(0.04)	(0.05)	(0.03)	(0.05)	(0.04)	(0.08)	(0.08)	(0.04)	(0.04)	(0.04)	(0.04)	(0.01)	(0.07)	(0.01)	(0.10)
Protein	-0.11**	0.03	0.00	-0.10	0.03	-0.07**	-0.22**	-0.22**	0.07*	0.06	0.06	-0.05	0.02	-0.25**	-0.03	-0.07**	-0.00	0.03	0.01**	0.83**
	(0.04)	(0.04)	(0.04)	(0.06)	(0.05)	(0.03)	(0.03)	(0.02)	(0.03)	(0.03)	(0.06)	(0.06)	(0.03)	(0.05)	(0.03)	(0.01)	(0.00)	(0.05)	(0.00)	(0.07)
Fat	-0.03	0.12	0.17*	-0.17	0.04	0.22**	-0.37**	-0.39**	0.36**	0.01	-0.03	-0.06	-0.01	-0.09	-0.12	-0.27**	0.06**	0.22	-0.06**	0.52**
	(0.10)	(0.08)	(0.07)	(0.09)	(0.09)	(0.08)	(0.10)	(0.06)	(0.10)	(0.07)	(0.16)	(0.11)	(0.07)	(0.07)	(0.08)	(0.08)	(0.02)	(0.12)	(0.01)	(0.17)
Carbohydrates	-0.05	-0.04	-0.01	-0.05	-0.11	-0.12**	-0.10**	-0.06**	-0.10**	0.03	-0.04	-0.07	0.05	0.00	-0.07*	0.02	-0.01	0.09	-0.00	0.71**
	(0.04)	(0.05)	(0.03)	(0.06)	(0.06)	(0.02)	(0.03)	(0.02)	(0.03)	(0.03)	(0.07)	(0.08)	(0.04)	(0.04)	(0.04)	(0.01)	(0.00)	(0.06)	(0.00)	(0.08)
Iron	-0.11**	0.02	-0.00	-0.07	-0.03	-0.08**	-0.26**	-0.20**	-0.20**	0.05*	0.19**	-0.05	0.08**	-0.05	0.02	-0.03*	0.00	0.20**	-0.01	0.76**
	(0.03)	(0.03)	(0.03)	(0.04)	(0.04)	(0.02)	(0.03)	(0.02)	(0.04)	(0.02)	(0.06)	(0.05)	(0.02)	(0.03)	(0.03)	(0.01)	(0.00)	(0.05)	(0.00)	(0.06)
Zinc	-0.11**	-0.02	-0.00	-0.08	-0.02	-0.07**	-0.16**	-0.23**	0.03	0.03	0.03	-0.02	0.02	-0.10**	-0.01	-0.06**	-0.00	0.03	0.01**	0.83**
	(0.03)	(0.04)	(0.03)	(0.05)	(0.05)	(0.02)	(0.03)	(0.02)	(0.03)	(0.03)	(0.06)	(0.05)	(0.03)	(0.03)	(0.03)	(0.01)	(0.00)	(0.06)	(0.00)	(0.06)
Vitamin A	0.01	0.27*	0.19*	-0.17	0.02	0.32**	-0.44**	-0.19**	0.33*	-0.04	-0.15	-0.07	-0.03	-0.05	-0.23*	-0.31**	0.08**	0.35*	-0.09**	0.38
	(0.12)	(0.11)	(0.08)	(0.11)	(0.11)	(0.11)	(0.12)	(0.07)	(0.14)	(0.09)	(0.22)	(0.15)	(0.09)	(0.08)	(0.10)	(0.11)	(0.03)	(0.16)	(0.02)	(0.23)
Total Folate	-0.12**	0.02	0.06	-0.10*	-0.02	-0.06*	-0.31**	-0.37**	-0.07*	0.07*	0.26**	-0.07	0.09**	-0.08**	0.05	-0.08**	0.00	0.14*	0.00	0.74**
	(0.04)	(0.04)	(0.04)	(0.05)	(0.05)	(0.03)	(0.03)	(0.02)	(0.04)	(0.03)	(0.07)	(0.05)	(0.03)	(0.03)	(0.04)	(0.02)	(0.01)	(0.06)	(0.00)	(0.07)

Note: This table shows the sample-wide median elasticity of macro- and micro-nutrient intake (quantity demanded) with respect to food prices (columns 1 thru 19) and total household expenditures (the last column). For a list of items in each food group, see Suppl. Table 48.

Suppl. Table 33: Nutrient intake elasticities with respect to food prices and total household expenditures for Q1 consumers (with per capita expenditures less than $1.90 per day) in Tanzania

	Food Group																			Exp.
	Rice	Maize	Wheat	Cassava	Roots	Sugar	Pulses	Nuts	Vegetables	Fruit	RedMeat	Poultry	Eggs	Fish	Dairy	Fats	Coffee	SoftDrink	OtherFood	
Kcal	-0.01	-0.30**	-0.11*	-0.15*	-0.12*	0.04	-0.10*	-0.09	-0.12**	0.04	-0.00	-0.08	-0.01	0.05	0.07	-0.02	0.01	-0.02	0.01	1.08**
	(0.10)	(0.12)	(0.05)	(0.07)	(0.05)	(0.03)	(0.05)	(0.06)	(0.04)	(0.04)	(0.09)	(0.09)	(0.03)	(0.04)	(0.08)	(0.02)	(0.01)	(0.02)	(0.01)	(0.14)
Protein	-0.06	-0.25*	-0.10*	-0.01	-0.16**	0.07*	-0.20**	-0.16*	-0.09*	0.03	-0.06	-0.07	-0.03	-0.06	0.05	-0.05*	0.02*	-0.02	0.01	1.13**
	(0.09)	(0.11)	(0.05)	(0.07)	(0.05)	(0.04)	(0.05)	(0.07)	(0.04)	(0.04)	(0.08)	(0.09)	(0.02)	(0.04)	(0.09)	(0.02)	(0.01)	(0.02)	(0.01)	(0.14)
Fat	0.06	-0.34*	0.00	0.11	0.05	-0.02	-0.24**	-0.29**	-0.20**	0.03	-0.15	-0.04	-0.05	-0.00	-0.01	-0.16**	0.01	0.01	0.02	1.23**
	(0.12)	(0.16)	(0.06)	(0.11)	(0.06)	(0.04)	(0.07)	(0.10)	(0.06)	(0.05)	(0.11)	(0.14)	(0.04)	(0.05)	(0.10)	(0.05)	(0.02)	(0.03)	(0.02)	(0.23)
Carbohydrates	-0.00	-0.30*	-0.13*	-0.21*	-0.13*	0.05	-0.06	-0.04	-0.11*	0.04	0.02	-0.09	0.01	0.08	0.09	0.00	0.01	-0.03	0.00	1.03**
	(0.12)	(0.12)	(0.05)	(0.09)	(0.06)	(0.04)	(0.05)	(0.07)	(0.04)	(0.05)	(0.11)	(0.10)	(0.03)	(0.05)	(0.08)	(0.02)	(0.01)	(0.03)	(0.01)	(0.17)
Iron	0.01	-0.32**	-0.11*	-0.12	-0.16**	0.08*	-0.15**	-0.10	-0.13**	0.05	-0.02	-0.13	-0.01	0.06	0.12	-0.04*	0.02	-0.03	0.01	1.00**
	(0.10)	(0.12)	(0.05)	(0.07)	(0.06)	(0.03)	(0.05)	(0.06)	(0.04)	(0.04)	(0.09)	(0.09)	(0.03)	(0.04)	(0.08)	(0.02)	(0.01)	(0.02)	(0.01)	(0.13)
Zinc	-0.03	-0.30*	-0.10*	-0.06	-0.15**	0.05	-0.14**	-0.14*	-0.11**	0.05	-0.04	-0.10	-0.02	0.02	0.08	-0.04*	0.02	-0.02	0.00	1.08**
	(0.09)	(0.12)	(0.04)	(0.07)	(0.05)	(0.03)	(0.05)	(0.06)	(0.04)	(0.04)	(0.08)	(0.09)	(0.02)	(0.04)	(0.08)	(0.02)	(0.01)	(0.02)	(0.01)	(0.14)
Vitamin A	-0.07	-0.25**	0.11	0.05	-0.10	0.09	-0.47**	-0.17**	-0.32**	-0.08	0.05	-0.14	0.01	0.10	0.15*	-0.25**	0.01	0.06*	0.01	1.23**
	(0.11)	(0.09)	(0.07)	(0.07)	(0.06)	(0.06)	(0.10)	(0.06)	(0.09)	(0.05)	(0.10)	(0.08)	(0.02)	(0.05)	(0.07)	(0.09)	(0.03)	(0.03)	(0.03)	(0.16)
Total Folate	0.03	-0.27**	0.03	-0.13	-0.33**	0.19**	-0.40**	-0.13	-0.20**	-0.03	0.06	-0.05	0.00	0.01	0.13	-0.10**	0.03*	0.00	0.04**	1.15**
	(0.12)	(0.10)	(0.07)	(0.09)	(0.08)	(0.05)	(0.08)	(0.07)	(0.06)	(0.06)	(0.11)	(0.09)	(0.03)	(0.05)	(0.09)	(0.03)	(0.02)	(0.03)	(0.01)	(0.16)

Note: This table shows the sample-wide median elasticity of macro- and micro-nutrient intake (quantity demanded) with respect to food prices (columns 1 thru 19) and total household expenditures (the last column). For a list of items in each food group, see Suppl. Table 49.

Suppl. Table 34: Nutrient intake elasticities with respect to food prices and total household expenditures for Q2 consumers (with per capita expenditures between $1.90 and $3.20 per day) in Tanzania

	Food Group																			Exp.
	Rice	Maize	Wheat	Cassava	Roots	Sugar	Pulses	Nuts	Vegetables	Fruit	RedMeat	Poultry	Eggs	Fish	Dairy	Fats	Coffee	SoftDrink	OtherFood	
Kcal	-0.07	-0.24*	-0.06	-0.09	-0.11*	-0.01	-0.04	-0.06	-0.10**	0.02	0.00	-0.07	-0.01	-0.01	0.04	-0.02	0.01	-0.00	0.00	0.97**
	(0.09)	(0.10)	(0.04)	(0.06)	(0.05)	(0.03)	(0.04)	(0.05)	(0.03)	(0.03)	(0.08)	(0.09)	(0.03)	(0.04)	(0.09)	(0.01)	(0.01)	(0.02)	(0.01)	(0.14)
Protein	-0.12	-0.17	-0.04	-0.01	-0.13**	0.02	-0.11*	-0.10	-0.06	-0.01	-0.09	-0.04	-0.05	-0.10**	-0.02	-0.04*	0.02*	-0.00	0.01	1.06**
	(0.09)	(0.10)	(0.04)	(0.07)	(0.05)	(0.03)	(0.05)	(0.06)	(0.03)	(0.03)	(0.08)	(0.10)	(0.03)	(0.04)	(0.09)	(0.02)	(0.01)	(0.02)	(0.01)	(0.14)
Fat	-0.05	-0.15	0.02	0.05	0.02	-0.03	-0.16**	-0.19*	-0.15**	-0.04	-0.14	-0.00	-0.07	-0.06	-0.06	-0.07	0.02	-0.01	0.00	1.17**
	(0.11)	(0.12)	(0.05)	(0.09)	(0.06)	(0.03)	(0.06)	(0.08)	(0.05)	(0.04)	(0.11)	(0.14)	(0.05)	(0.04)	(0.08)	(0.05)	(0.01)	(0.03)	(0.01)	(0.19)
Carbohydrates	-0.06	-0.27**	-0.08	-0.14*	-0.14**	-0.00	-0.00	-0.03	-0.10**	0.04	0.04	-0.09	0.01	0.02	0.08	-0.00	0.00	-0.00	-0.00	0.91**
	(0.10)	(0.10)	(0.05)	(0.07)	(0.05)	(0.03)	(0.04)	(0.06)	(0.03)	(0.03)	(0.09)	(0.09)	(0.03)	(0.05)	(0.09)	(0.01)	(0.01)	(0.02)	(0.01)	(0.15)
Iron	-0.03	-0.27**	-0.04	-0.10	-0.17**	0.03	-0.07	-0.07	-0.11**	0.02	0.01	-0.11	-0.01	0.02	0.10	-0.03*	0.01	-0.00	0.00	0.91**
	(0.08)	(0.10)	(0.04)	(0.06)	(0.05)	(0.03)	(0.04)	(0.05)	(0.03)	(0.03)	(0.08)	(0.09)	(0.02)	(0.04)	(0.08)	(0.01)	(0.01)	(0.02)	(0.01)	(0.13)
Zinc	-0.09	-0.22	-0.05	-0.04	-0.13*	-0.00	-0.07	-0.09	-0.10**	0.02	-0.05	-0.06	-0.03	-0.02	0.02	-0.03*	0.02	-0.00	0.01	0.99**
	(0.09)	(0.11)	(0.04)	(0.07)	(0.05)	(0.03)	(0.04)	(0.06)	(0.03)	(0.03)	(0.09)	(0.11)	(0.03)	(0.04)	(0.10)	(0.01)	(0.01)	(0.02)	(0.01)	(0.16)
Vitamin A	-0.14	-0.12	0.08	0.00	-0.03	0.07	-0.31**	-0.12**	-0.28**	-0.06	0.06	-0.08	-0.02	0.01	0.07	-0.12	0.04	0.03	-0.01	1.07**
	(0.08)	(0.06)	(0.05)	(0.05)	(0.05)	(0.05)	(0.08)	(0.04)	(0.07)	(0.04)	(0.08)	(0.06)	(0.02)	(0.05)	(0.05)	(0.10)	(0.02)	(0.02)	(0.02)	(0.11)
Total Folate	-0.06	-0.18**	0.02	-0.08	-0.27**	0.14**	-0.19*	-0.09	-0.16**	-0.05	-0.02	-0.03	-0.01	-0.03	0.07	-0.08**	0.03*	0.00	0.04**	1.00**
	(0.09)	(0.07)	(0.05)	(0.07)	(0.06)	(0.04)	(0.07)	(0.05)	(0.04)	(0.04)	(0.08)	(0.07)	(0.03)	(0.04)	(0.07)	(0.03)	(0.01)	(0.02)	(0.01)	(0.12)

Note: This table shows the sample-wide median elasticity of macro- and micro-nutrient intake (quantity demanded) with respect to food prices (columns 1 thru 19) and total household expenditures (the last column). For a list of items in each food group, see Suppl. Table 49.

Suppl. Table 35: Nutrient intake elasticities with respect to food prices and total household expenditures for Q3 consumers (with per capita expenditures between $3.20 and $5.50 per day) in Tanzania

	Food Group																			Exp.
	Rice	Maize	Wheat	Cassava	Roots	Sugar	Pulses	Nuts	Vegetables	Fruit	RedMeat	Poultry	Eggs	Fish	Dairy	Fats	Coffee	SoftDrink	OtherFood	
Kcal	-0.11	-0.16	-0.02	-0.10	-0.09	-0.02	-0.01	-0.03	-0.09*	0.01	-0.01	-0.05	-0.02	-0.03	-0.02	0.00	0.01	0.01	-0.00	0.88**
	(0.14)	(0.12)	(0.05)	(0.08)	(0.07)	(0.03)	(0.05)	(0.08)	(0.04)	(0.04)	(0.14)	(0.14)	(0.04)	(0.05)	(0.12)	(0.02)	(0.01)	(0.03)	(0.01)	(0.19)
Protein	-0.16	-0.07	-0.01	-0.02	-0.09	-0.00	-0.03	-0.04	-0.06	-0.04	-0.15	-0.01	-0.07	-0.10*	-0.12	-0.03	0.02	0.00	0.02	1.00**
	(0.14)	(0.11)	(0.05)	(0.09)	(0.07)	(0.04)	(0.07)	(0.09)	(0.04)	(0.04)	(0.13)	(0.15)	(0.05)	(0.04)	(0.12)	(0.02)	(0.01)	(0.03)	(0.01)	(0.19)
Fat	-0.17	-0.00	0.05	0.00	0.01	-0.01	-0.12	-0.09	-0.12*	-0.08*	-0.13	0.01	-0.08	-0.10*	-0.13	0.04	0.03*	-0.01	-0.02	1.13**
	(0.15)	(0.10)	(0.05)	(0.09)	(0.06)	(0.04)	(0.07)	(0.09)	(0.05)	(0.04)	(0.13)	(0.17)	(0.05)	(0.04)	(0.09)	(0.07)	(0.01)	(0.03)	(0.01)	(0.18)
Carbohydrates	-0.07	-0.22	-0.04	-0.15	-0.11	-0.02	0.03	-0.01	-0.10*	0.04	0.05	-0.08	0.00	0.00	0.04	-0.00	0.00	0.02	-0.00	0.78**
	(0.16)	(0.13)	(0.06)	(0.09)	(0.07)	(0.03)	(0.05)	(0.09)	(0.05)	(0.04)	(0.15)	(0.15)	(0.04)	(0.06)	(0.13)	(0.02)	(0.01)	(0.03)	(0.01)	(0.20)
Iron	-0.06	-0.19	-0.00	-0.12	-0.15*	0.01	0.02	-0.02	-0.10**	-0.01	0.03	-0.09	-0.02	0.00	0.06	-0.02	0.01	0.01	0.00	0.76**
	(0.12)	(0.12)	(0.04)	(0.08)	(0.06)	(0.03)	(0.05)	(0.07)	(0.04)	(0.03)	(0.12)	(0.13)	(0.04)	(0.05)	(0.10)	(0.02)	(0.01)	(0.03)	(0.01)	(0.18)
Zinc	-0.13	-0.12	-0.01	-0.05	-0.09	-0.02	-0.03	-0.03	-0.09	-0.02	-0.09	-0.03	-0.05	-0.03	-0.07	-0.02	0.02	0.00	0.01	0.91**
	(0.16)	(0.13)	(0.05)	(0.10)	(0.07)	(0.03)	(0.07)	(0.09)	(0.05)	(0.04)	(0.15)	(0.16)	(0.05)	(0.05)	(0.14)	(0.02)	(0.01)	(0.03)	(0.01)	(0.22)
Vitamin A	-0.24*	-0.02	0.07	-0.06	-0.02	0.06	-0.20*	-0.06	-0.26**	-0.04	0.09	-0.05	-0.04	-0.09	0.02	0.04	0.06*	0.01	-0.04	0.93**
	(0.10)	(0.07)	(0.06)	(0.06)	(0.05)	(0.05)	(0.09)	(0.04)	(0.07)	(0.04)	(0.09)	(0.06)	(0.03)	(0.05)	(0.06)	(0.13)	(0.02)	(0.02)	(0.02)	(0.12)
Total Folate	-0.12	-0.09	0.04	-0.07	-0.23**	0.11*	0.03	-0.04	-0.13**	-0.07	-0.10	0.00	-0.02	-0.06	0.01	-0.05	0.03*	0.00	0.04**	0.84**
	(0.11)	(0.07)	(0.06)	(0.07)	(0.06)	(0.04)	(0.09)	(0.06)	(0.04)	(0.04)	(0.10)	(0.10)	(0.03)	(0.04)	(0.08)	(0.03)	(0.01)	(0.02)	(0.01)	(0.15)

Note: This table shows the sample-wide median elasticity of macro- and micro-nutrient intake (quantity demanded) with respect to food prices (columns 1 thru 19) and total household expenditures (the last column). For a list of items in each food group, see Suppl. Table 49.

Suppl. Table 36: Nutrient intake elasticities with respect to food prices and total household expenditures for Q4 consumers (with per capita expenditures greater than $5.50 per day) in Tanzania

	Food Group																			Exp.
	Rice	Maize	Wheat	Cassava	Roots	Sugar	Pulses	Nuts	Vegetables	Fruit	RedMeat	Poultry	Eggs	Fish	Dairy	Fats	Coffee	SoftDrink	OtherFood	
Kcal	-0.09	-0.09	0.03	-0.17	-0.02	-0.01	0.04	0.03	-0.11	0.01	-0.02	-0.00	-0.04	-0.06	-0.11	0.04	0.01	0.01	-0.01	0.85*
	(0.28)	(0.15)	(0.08)	(0.13)	(0.10)	(0.04)	(0.10)	(0.16)	(0.07)	(0.06)	(0.27)	(0.23)	(0.08)	(0.07)	(0.18)	(0.04)	(0.02)	(0.05)	(0.01)	(0.35)
Protein	-0.19	0.03	0.03	-0.07	-0.01	-0.02	0.06	0.04	-0.07	-0.08	-0.23	0.03	-0.10	-0.10	-0.25	-0.02	0.02	0.00	0.02	1.06**
	(0.26)	(0.13)	(0.07)	(0.13)	(0.10)	(0.04)	(0.14)	(0.16)	(0.06)	(0.05)	(0.23)	(0.24)	(0.08)	(0.06)	(0.18)	(0.03)	(0.02)	(0.05)	(0.01)	(0.35)
Fat	-0.31	0.14	0.09	-0.08	-0.01	0.02	-0.07	-0.00	-0.10	-0.12*	-0.08	0.03	-0.10	-0.17**	-0.22	0.22*	0.06**	-0.02	-0.06*	1.19**
	(0.21)	(0.10)	(0.07)	(0.11)	(0.07)	(0.06)	(0.10)	(0.13)	(0.07)	(0.05)	(0.17)	(0.20)	(0.07)	(0.06)	(0.13)	(0.10)	(0.02)	(0.04)	(0.02)	(0.27)
Carbohydrates	0.00	-0.19	0.00	-0.22	-0.03	-0.02	0.08	0.04	-0.11	0.06	0.05	-0.02	0.00	-0.01	-0.03	-0.00	-0.00	0.03	0.00	0.67
	(0.32)	(0.18)	(0.10)	(0.14)	(0.12)	(0.06)	(0.11)	(0.18)	(0.08)	(0.08)	(0.32)	(0.25)	(0.08)	(0.08)	(0.21)	(0.05)	(0.02)	(0.05)	(0.02)	(0.39)
Iron	-0.06	-0.09	0.02	-0.20	-0.07	-0.03	0.17	0.06	-0.10	-0.04	0.04	-0.02	-0.03	-0.01	-0.03	-0.00	0.00	0.03	-0.01	0.58
	(0.26)	(0.16)	(0.08)	(0.13)	(0.10)	(0.04)	(0.11)	(0.16)	(0.06)	(0.06)	(0.26)	(0.22)	(0.07)	(0.07)	(0.17)	(0.04)	(0.02)	(0.05)	(0.01)	(0.36)
Zinc	-0.15	-0.02	0.03	-0.10	-0.01	-0.03	0.01	0.06	-0.10	-0.06	-0.15	0.01	-0.07	-0.03	-0.21	-0.01	0.01	0.01	0.01	0.98*
	(0.31)	(0.17)	(0.08)	(0.15)	(0.12)	(0.04)	(0.14)	(0.18)	(0.08)	(0.06)	(0.29)	(0.28)	(0.09)	(0.07)	(0.21)	(0.04)	(0.02)	(0.06)	(0.01)	(0.42)
Vitamin A	-0.43*	0.13	0.07	-0.16	-0.00	0.07	-0.05	0.01	-0.28**	-0.01	0.18	-0.01	-0.07	-0.23**	-0.04	0.33	0.10**	-0.01	-0.10**	0.72**
	(0.18)	(0.10)	(0.10)	(0.08)	(0.07)	(0.08)	(0.13)	(0.08)	(0.09)	(0.06)	(0.16)	(0.10)	(0.05)	(0.08)	(0.10)	(0.19)	(0.03)	(0.03)	(0.04)	(0.21)
Total Folate	-0.19	-0.01	0.05	-0.08	-0.11	0.06	0.36*	0.04	-0.13*	-0.09	-0.22	0.06	-0.04	-0.09	-0.10	-0.02	0.02	0.00	0.02	0.63*
	(0.19)	(0.10)	(0.09)	(0.10)	(0.09)	(0.06)	(0.16)	(0.12)	(0.06)	(0.06)	(0.19)	(0.17)	(0.06)	(0.06)	(0.13)	(0.04)	(0.02)	(0.04)	(0.02)	(0.28)

Note: This table shows the sample-wide median elasticity of macro- and micro-nutrient intake (quantity demanded) with respect to food prices (columns 1 thru 19) and total household expenditures (the last column). For a list of items in each food group, see Suppl. Table 49.

Suppl. Table 37: Nutrient intake elasticities with respect to food prices and total household expenditures for Q1 consumers (with per capita expenditures less than $1.90 per day) in Nigeria

	Food Group																			Exp.
	Rice	Maize	Wheat	Cassava	Roots	Sugar	Pulses	Nuts	Vegetables	Fruit	RedMeat	Poultry	Eggs	Fish	Dairy	Oils	Coffee	SoftDrink	OtherFood	
Kcal	-0.17**	-0.03	-0.08	-0.08**	-0.14**	-0.05*	-0.09**	-0.00	-0.07*	-0.04	-0.09	0.00	-0.03	-0.06	0.04	-0.11**	0.06**	0.00	0.02	0.98**
	(0.04)	(0.03)	(0.13)	(0.02)	(0.05)	(0.02)	(0.02)	(0.03)	(0.03)	(0.04)	(0.07)	(0.04)	(0.03)	(0.04)	(0.02)	(0.01)	(0.02)	(0.03)	(0.01)	(0.10)
Protein	-0.14**	-0.06	-0.11	-0.04	-0.06	-0.06**	-0.11**	-0.02	-0.12**	-0.04	-0.14	0.00	-0.03	-0.07	0.03	-0.04**	0.05**	-0.00	0.03*	1.05**
	(0.04)	(0.04)	(0.15)	(0.02)	(0.05)	(0.02)	(0.02)	(0.03)	(0.03)	(0.05)	(0.08)	(0.04)	(0.03)	(0.05)	(0.03)	(0.01)	(0.02)	(0.03)	(0.02)	(0.10)
Fat	-0.06*	0.02	-0.04	-0.04**	-0.07*	-0.02	-0.06**	-0.01	-0.06**	0.00	-0.01	-0.08*	0.02	-0.06*	0.04*	-0.35**	0.03*	-0.03	-0.01	0.75**
	(0.03)	(0.02)	(0.06)	(0.01)	(0.03)	(0.01)	(0.02)	(0.01)	(0.02)	(0.02)	(0.04)	(0.03)	(0.02)	(0.02)	(0.02)	(0.02)	(0.01)	(0.02)	(0.01)	(0.06)
Carbohydrates	-0.21**	-0.04	-0.08	-0.10**	-0.17**	-0.05*	-0.10**	0.00	-0.07*	-0.05	-0.10	0.02	-0.04	-0.06	0.04	-0.05**	0.07**	0.01	0.03	1.03**
	(0.05)	(0.04)	(0.14)	(0.03)	(0.06)	(0.02)	(0.02)	(0.03)	(0.03)	(0.05)	(0.08)	(0.05)	(0.03)	(0.05)	(0.03)	(0.01)	(0.02)	(0.03)	(0.02)	(0.11)
Iron	-0.14**	-0.02	-0.06	-0.05	-0.12	-0.05	-0.09**	-0.04	-0.14**	-0.08	-0.19*	0.01	-0.06	-0.08	0.07*	-0.04**	0.06**	0.01	0.03	1.05**
	(0.04)	(0.05)	(0.18)	(0.02)	(0.07)	(0.03)	(0.03)	(0.03)	(0.04)	(0.05)	(0.09)	(0.06)	(0.04)	(0.05)	(0.03)	(0.01)	(0.02)	(0.03)	(0.02)	(0.13)
Zinc	-0.16**	-0.11**	-0.08	-0.08**	-0.13**	-0.03	-0.13**	-0.01	-0.08**	-0.02	-0.11	0.03	-0.03	-0.05	0.04	-0.04**	0.06**	0.01	0.02	1.06**
	(0.04)	(0.04)	(0.11)	(0.02)	(0.05)	(0.02)	(0.02)	(0.02)	(0.03)	(0.04)	(0.07)	(0.04)	(0.03)	(0.04)	(0.02)	(0.01)	(0.01)	(0.02)	(0.01)	(0.10)
Vitamin A	-0.08**	0.04*	0.00	-0.06**	-0.09**	0.00	-0.04*	0.03**	-0.06**	0.02	0.09**	-0.11**	0.04*	-0.06**	0.07**	-0.51**	0.02*	-0.05**	-0.03**	0.58**
	(0.03)	(0.02)	(0.03)	(0.02)	(0.03)	(0.01)	(0.02)	(0.01)	(0.02)	(0.02)	(0.03)	(0.03)	(0.02)	(0.02)	(0.02)	(0.03)	(0.01)	(0.02)	(0.01)	(0.04)
Total Folate	-0.14**	-0.10**	-0.08	-0.06**	-0.11*	-0.04*	-0.15**	-0.02	-0.13**	-0.06	-0.14*	0.01	-0.05	-0.03	0.09**	-0.03**	0.05**	-0.01	0.03*	0.94**
	(0.03)	(0.04)	(0.11)	(0.02)	(0.05)	(0.02)	(0.03)	(0.02)	(0.03)	(0.04)	(0.06)	(0.05)	(0.03)	(0.04)	(0.02)	(0.01)	(0.02)	(0.02)	(0.01)	(0.10)

Note: This table shows the sample-wide median elasticity of macro- and micro-nutrient intake (quantity demanded) with respect to food prices (columns 1 thru 19) and total household expenditures (the last column). For a list of items in each food group, see Suppl. Table 50.

Suppl. Table 38: Nutrient intake elasticities with respect to food prices and total household expenditures for Q2 consumers (with per capita expenditures between $1.90 and $3.20 per day) in Nigeria

	Food Group																			Exp.
	Rice	Maize	Wheat	Cassava	Roots	Sugar	Pulses	Nuts	Vegetables	Fruit	RedMeat	Poultry	Eggs	Fish	Dairy	Oils	Coffee	SoftDrink	OtherFood	
Kcal	-0.13**	-0.07**	-0.08	-0.10**	-0.10*	-0.02	-0.10**	0.02	-0.04**	0.01	-0.02	0.04	-0.00	-0.02	0.01	-0.09**	0.05**	0.01	0.01	0.91**
	(0.03)	(0.02)	(0.07)	(0.02)	(0.04)	(0.01)	(0.01)	(0.02)	(0.02)	(0.03)	(0.04)	(0.04)	(0.02)	(0.03)	(0.01)	(0.01)	(0.01)	(0.02)	(0.01)	(0.08)
Protein	-0.13**	-0.08**	-0.10	-0.08**	-0.03	-0.03*	-0.12**	0.00	-0.07**	0.01	-0.07	0.03	-0.00	-0.05	0.00	-0.02*	0.05**	0.00	0.02*	0.98**
	(0.02)	(0.02)	(0.08)	(0.01)	(0.04)	(0.01)	(0.02)	(0.02)	(0.02)	(0.03)	(0.04)	(0.04)	(0.02)	(0.03)	(0.02)	(0.01)	(0.02)	(0.02)	(0.01)	(0.09)
Fat	-0.03	-0.00	-0.01	-0.04**	-0.05	-0.00	-0.05**	0.00	-0.06**	0.01	-0.00	-0.06	0.03*	-0.05**	0.01	-0.37**	0.02*	-0.03*	-0.00	0.75**
	(0.02)	(0.02)	(0.03)	(0.01)	(0.03)	(0.01)	(0.01)	(0.01)	(0.01)	(0.02)	(0.03)	(0.03)	(0.01)	(0.02)	(0.01)	(0.02)	(0.01)	(0.01)	(0.01)	(0.05)
Carbohydrates	-0.17**	-0.08**	-0.09	-0.13**	-0.12**	-0.03*	-0.11**	0.03	-0.03	0.01	-0.02	0.07	-0.01	-0.01	0.01	-0.02*	0.06**	0.02	0.01	0.95**
	(0.03)	(0.03)	(0.08)	(0.02)	(0.05)	(0.01)	(0.02)	(0.02)	(0.02)	(0.03)	(0.04)	(0.05)	(0.02)	(0.03)	(0.02)	(0.01)	(0.02)	(0.02)	(0.01)	(0.09)
Iron	-0.09**	-0.08**	-0.11	-0.09**	-0.05	-0.01	-0.10**	0.01	-0.08**	0.02	-0.05	0.08	-0.02	-0.00	0.03	-0.02*	0.07**	0.03	0.00	0.96**
	(0.03)	(0.03)	(0.11)	(0.02)	(0.06)	(0.01)	(0.02)	(0.02)	(0.02)	(0.04)	(0.05)	(0.05)	(0.02)	(0.04)	(0.02)	(0.01)	(0.02)	(0.02)	(0.01)	(0.12)
Zinc	-0.14**	-0.10**	-0.09	-0.11**	-0.09*	-0.02	-0.13**	0.00	-0.05**	0.02	-0.05	0.05	-0.01	-0.02	0.02	-0.02**	0.05**	0.01	0.01	0.98**
	(0.02)	(0.02)	(0.06)	(0.01)	(0.04)	(0.01)	(0.02)	(0.01)	(0.01)	(0.03)	(0.04)	(0.04)	(0.02)	(0.02)	(0.01)	(0.01)	(0.01)	(0.01)	(0.01)	(0.08)
Vitamin A	-0.02	0.02	0.03	-0.04**	-0.07*	0.01	-0.03	0.04**	-0.08**	0.00	0.08**	-0.09**	0.04**	-0.06**	0.03*	-0.54**	0.02	-0.04**	-0.01	0.59**
	(0.03)	(0.01)	(0.03)	(0.01)	(0.03)	(0.01)	(0.02)	(0.01)	(0.02)	(0.02)	(0.03)	(0.02)	(0.01)	(0.02)	(0.01)	(0.02)	(0.01)	(0.02)	(0.01)	(0.04)
Total Folate	-0.11**	-0.09**	-0.10	-0.12**	-0.08	-0.03*	-0.15**	-0.01	-0.09**	-0.02	-0.06	0.02	-0.02	0.01	0.05**	-0.02*	0.05**	0.01	0.01	0.89**
	(0.02)	(0.03)	(0.06)	(0.02)	(0.04)	(0.01)	(0.02)	(0.02)	(0.02)	(0.03)	(0.04)	(0.05)	(0.02)	(0.03)	(0.02)	(0.01)	(0.02)	(0.02)	(0.01)	(0.09)

Note: This table shows the sample-wide median elasticity of macro- and micro-nutrient intake (quantity demanded) with respect to food prices (columns 1 thru 19) and total household expenditures (the last column). For a list of items in each food group, see Suppl. Table 50.

Suppl. Table 39: Nutrient intake elasticities with respect to food prices and total household expenditures for Q3 consumers (with per capita expenditures between \$3.20 and \$5.50 per day) in Nigeria

	Food Group																			Exp.
	Rice	Maize	Wheat	Cassava	Roots	Sugar	Pulses	Nuts	Vegetables	Fruit	RedMeat	Poultry	Eggs	Fish	Dairy	Oils	Coffee	SoftDrink	OtherFood	
Kcal	-0.10**	-0.08*	-0.06	-0.13**	-0.08	-0.01	-0.09**	0.04	-0.03	0.03	0.00	0.03	0.02	0.00	-0.01	-0.09**	0.04*	-0.00	0.00	0.89**
	(0.03)	(0.03)	(0.06)	(0.02)	(0.06)	(0.01)	(0.01)	(0.03)	(0.02)	(0.05)	(0.05)	(0.06)	(0.01)	(0.04)	(0.02)	(0.01)	(0.02)	(0.02)	(0.01)	(0.11)
Protein	-0.12**	-0.07*	-0.08	-0.11**	-0.03	-0.00	-0.10**	0.01	-0.04	0.02	-0.06	0.02	0.01	-0.06	-0.01	-0.00	0.04*	0.00	0.01	0.98**
	(0.02)	(0.03)	(0.06)	(0.02)	(0.06)	(0.01)	(0.02)	(0.03)	(0.02)	(0.05)	(0.05)	(0.07)	(0.01)	(0.04)	(0.02)	(0.01)	(0.02)	(0.02)	(0.01)	(0.11)
Fat	0.01	-0.01	0.05	-0.04**	-0.05	0.01	-0.03**	0.00	-0.08**	-0.02	-0.02	-0.06	0.03**	-0.06*	-0.02	-0.40**	0.01	-0.04*	0.01	0.78**
	(0.02)	(0.02)	(0.03)	(0.01)	(0.04)	(0.01)	(0.01)	(0.02)	(0.02)	(0.03)	(0.03)	(0.04)	(0.01)	(0.02)	(0.01)	(0.02)	(0.01)	(0.02)	(0.01)	(0.06)
Carbohydrates	-0.13**	-0.10**	-0.10	-0.16**	-0.10	-0.01	-0.11**	0.05	-0.02	0.04	0.02	0.07	0.01	0.03	-0.00	0.00	0.05*	0.01	0.00	0.91**
	(0.03)	(0.04)	(0.07)	(0.02)	(0.07)	(0.01)	(0.02)	(0.03)	(0.02)	(0.06)	(0.06)	(0.07)	(0.02)	(0.04)	(0.02)	(0.01)	(0.02)	(0.03)	(0.01)	(0.13)
Iron	-0.06	-0.11**	-0.17*	-0.15**	-0.01	0.01	-0.11**	0.05	-0.05*	0.09	0.03	0.12	0.01	0.05	0.00	-0.00	0.06*	0.04	-0.02	0.99**
	(0.03)	(0.04)	(0.08)	(0.02)	(0.07)	(0.01)	(0.02)	(0.04)	(0.02)	(0.06)	(0.06)	(0.08)	(0.02)	(0.05)	(0.02)	(0.01)	(0.02)	(0.03)	(0.01)	(0.14)
Zinc	-0.13**	-0.07*	-0.09	-0.14**	-0.08	-0.01	-0.12**	0.01	-0.04*	0.03	-0.04	0.02	0.01	-0.02	-0.00	-0.00	0.04*	0.00	0.01	0.94**
	(0.02)	(0.03)	(0.05)	(0.01)	(0.05)	(0.01)	(0.02)	(0.03)	(0.02)	(0.05)	(0.04)	(0.06)	(0.01)	(0.03)	(0.02)	(0.01)	(0.02)	(0.02)	(0.01)	(0.10)
Vitamin A	0.04	0.00	0.09*	-0.03	-0.05	0.02	-0.01	0.04**	-0.11**	-0.03	0.05	-0.07**	0.04**	-0.07**	-0.01	-0.58**	0.01	-0.04*	0.00	0.60**
	(0.03)	(0.02)	(0.04)	(0.02)	(0.03)	(0.01)	(0.02)	(0.01)	(0.02)	(0.02)	(0.03)	(0.02)	(0.01)	(0.02)	(0.02)	(0.03)	(0.01)	(0.02)	(0.01)	(0.05)
Total Folate	-0.11**	-0.07	-0.09	-0.17**	-0.08	-0.02	-0.14**	-0.00	-0.07**	0.01	-0.02	0.02	-0.00	0.03	0.01	-0.01	0.05*	0.02	0.01	0.92**
	(0.03)	(0.04)	(0.06)	(0.02)	(0.07)	(0.01)	(0.02)	(0.03)	(0.02)	(0.06)	(0.06)	(0.07)	(0.02)	(0.04)	(0.02)	(0.01)	(0.02)	(0.03)	(0.01)	(0.11)

Note: This table shows the sample-wide median elasticity of macro- and micro-nutrient intake (quantity demanded) with respect to food prices (columns 1 thru 19) and total household expenditures (the last column). For a list of items in each food group, see Suppl. Table 50.

Suppl. Table 40: Nutrient intake elasticities with respect to food prices and total household expenditures for Q4 consumers (with per capita expenditures greater than $5.50 per day) in Nigeria

	Food Group																			Exp.
	Rice	Maize	Wheat	Cassava	Roots	Sugar	Pulses	Nuts	Vegetables	Fruit	RedMeat	Poultry	Eggs	Fish	Dairy	Oils	Coffee	SoftDrink	OtherFood	
Kcal	-0.04	-0.07	0.03	-0.13**	-0.10	0.01	-0.08**	0.02	-0.02	-0.01	-0.02	-0.01	0.02	-0.01	-0.02	-0.10**	0.01	-0.03	0.01	0.75**
	(0.04)	(0.05)	(0.10)	(0.02)	(0.09)	(0.02)	(0.02)	(0.05)	(0.03)	(0.08)	(0.09)	(0.09)	(0.02)	(0.07)	(0.03)	(0.01)	(0.02)	(0.04)	(0.01)	(0.19)
Protein	-0.13**	-0.04	0.04	-0.13**	-0.08	0.02	-0.08**	-0.02	-0.01	-0.03	-0.09	-0.05	0.00	-0.12	-0.01	0.01	0.03	-0.03	0.02	0.85**
	(0.04)	(0.06)	(0.10)	(0.02)	(0.09)	(0.01)	(0.02)	(0.05)	(0.03)	(0.09)	(0.08)	(0.09)	(0.02)	(0.06)	(0.03)	(0.01)	(0.02)	(0.04)	(0.02)	(0.18)
Fat	0.07*	-0.01	0.18**	-0.01	-0.05	0.03	-0.00	-0.01	-0.09**	-0.08	-0.06	-0.05	0.03	-0.09*	-0.05*	-0.47**	-0.00	-0.06*	0.03**	0.61**
	(0.03)	(0.03)	(0.06)	(0.02)	(0.06)	(0.01)	(0.02)	(0.03)	(0.02)	(0.05)	(0.05)	(0.05)	(0.02)	(0.04)	(0.02)	(0.03)	(0.02)	(0.03)	(0.01)	(0.09)
Carbohydrates	-0.08	-0.10	-0.04	-0.18**	-0.13	0.00	-0.11**	0.03	0.00	0.03	0.00	0.02	0.02	0.04	-0.01	0.03**	0.02	-0.02	0.00	0.77**
	(0.05)	(0.06)	(0.12)	(0.03)	(0.10)	(0.02)	(0.02)	(0.06)	(0.04)	(0.10)	(0.11)	(0.11)	(0.03)	(0.08)	(0.03)	(0.01)	(0.03)	(0.04)	(0.02)	(0.23)
Iron	-0.02	-0.11	-0.14	-0.20**	-0.02	0.02	-0.09**	0.05	-0.02	0.11	0.07	0.09	0.04	0.05	-0.03	0.01	0.05	0.03	-0.03	0.94**
	(0.05)	(0.07)	(0.12)	(0.03)	(0.10)	(0.02)	(0.03)	(0.06)	(0.03)	(0.10)	(0.10)	(0.11)	(0.03)	(0.08)	(0.04)	(0.01)	(0.03)	(0.04)	(0.01)	(0.22)
Zinc	-0.12**	-0.03	-0.01	-0.16**	-0.11	0.00	-0.09**	-0.02	-0.02	-0.01	-0.06	-0.04	0.02	-0.05	-0.00	0.02	0.03	-0.02	0.01	0.80**
	(0.03)	(0.05)	(0.10)	(0.02)	(0.08)	(0.01)	(0.02)	(0.05)	(0.03)	(0.08)	(0.07)	(0.09)	(0.02)	(0.06)	(0.03)	(0.01)	(0.02)	(0.04)	(0.01)	(0.17)
Vitamin A	0.15**	-0.03	0.19**	0.01	-0.02	0.04	0.02	0.04*	-0.13**	-0.06	0.00	-0.02	0.03	-0.07*	-0.08**	-0.65**	0.00	-0.03	0.03**	0.46**
	(0.04)	(0.03)	(0.06)	(0.02)	(0.05)	(0.02)	(0.02)	(0.02)	(0.02)	(0.04)	(0.05)	(0.04)	(0.02)	(0.03)	(0.03)	(0.03)	(0.02)	(0.03)	(0.01)	(0.07)
Total Folate	-0.10*	-0.03	-0.02	-0.24**	-0.12	-0.01	-0.10**	-0.03	-0.03	0.00	-0.01	-0.02	0.01	0.01	-0.03	0.01	0.05	0.02	0.01	0.79**
	(0.04)	(0.06)	(0.12)	(0.03)	(0.11)	(0.02)	(0.04)	(0.06)	(0.04)	(0.09)	(0.11)	(0.11)	(0.03)	(0.08)	(0.03)	(0.01)	(0.03)	(0.04)	(0.02)	(0.21)

Note: This table shows the sample-wide median elasticity of macro- and micro-nutrient intake (quantity demanded) with respect to food prices (columns 1 thru 19) and total household expenditures (the last column). For a list of items in each food group, see Suppl. Table 50.

Suppl. Table 41: Average share in total expenditures and unit value, by food group and consumer total expenditure quartile (Malawi)

	Average Share in Total Expenditures				Average Unit Value (MWK/kg)			
	Q1	Q2	Q3	Q4	Q1	Q2	Q3	Q4
Rice	0.011	0.021	0.026	0.022	306.507	339.088	372.693	419.358
Maize	0.338	0.256	0.184	0.088	107.728	124.724	139.369	156.795
Wheat	0.018	0.024	0.032	0.033	408.212	444.919	481.809	534.398
Cassava	0.019	0.017	0.011	0.004	79.052	84.189	93.323	100.539
Roots	0.032	0.031	0.026	0.019	76.244	85.688	93.489	107.856
Sugar	0.031	0.042	0.039	0.023	232.329	261.079	283.394	312.479
Pulses	0.063	0.059	0.046	0.025	323.756	355.109	398.133	429.051
Nuts	0.009	0.008	0.007	0.004	852.901	931.380	973.583	1,053.580
Vegetables	0.089	0.068	0.055	0.034	150.961	173.234	195.940	229.132
Fruit	0.021	0.018	0.017	0.012	93.277	102.701	114.427	131.339
RedMeat	0.017	0.037	0.049	0.044	867.089	965.400	1,039.554	1,164.562
Poultry	0.011	0.029	0.043	0.036	887.616	962.782	1,088.685	1,241.915
Eggs	0.010	0.015	0.020	0.016	498.913	554.001	586.303	645.483
Dairy	0.001	0.005	0.013	0.018	887.178	964.654	1,009.831	1,166.918
Oils	0.026	0.032	0.031	0.023	696.881	768.567	831.379	961.203
Tea	0.002	0.004	0.005	0.002	1,830.433	1,959.544	2,016.131	2,218.155
SoftDrink	0.002	0.004	0.010	0.019	416.621	448.536	473.794	507.545
OtherFood	0.022	0.013	0.009	0.006	388.983	438.085	476.786	516.283
Food (total)	0.722	0.682	0.621	0.430	166.363	226.191	278.132	248.676
Non-food	0.278	0.318	0.379	0.570				
N (total)	2763	2290	1736	1300				
N (rural)	2563	1870	1125	434				
N (urban)	200	420	611	866				

Notes: This table shows the mean value of each food group's share in total expenditures (and the non-food numéraire good's share) in columns 1-4 and each food group's mean unit value (and the non-food numéraire good's median unit value) in columns 5-8. Both budget shares and unit values are summarized by per-capita total expenditures quartile. Mean budget shares and unit values are weighted using survey weights. The unit value for total food is also weighted by budget share. Food groups whose names have been shortened are marked with ∓; The food items contained in each food group are listed in Suppl. Table 46.

Suppl. Table 42: Average share in total expenditures and unit value, by food group and consumer total expenditure quartile (Niger)

	Average Share in Total Expenditures				Average Unit Value (FCFA/kg)			
	Q1	Q2	Q3	Q4	Q1	Q2	Q3	Q4
Rice	0.044	0.065	0.086	0.084	619.943	567.671	693.850	595.668
Millet	0.306	0.255	0.169	0.101	199.748	253.184	220.344	228.564
Wheat	0.102	0.114	0.115	0.096	502.132	650.978	668.956	774.936
Cassava	0.007	0.008	0.009	0.010	347.700	190.830	220.763	143.940
Roots	0.002	0.004	0.008	0.011	394.866	385.131	400.714	416.912
Sugar	0.012	0.013	0.014	0.015	705.521	528.160	577.127	637.779
Pulses	0.022	0.024	0.025	0.025	487.537	384.868	388.725	394.579
Nuts	0.005	0.005	0.005	0.011	547.933	514.073	589.454	449.734
Vegetables	0.029	0.030	0.032	0.036	285.186	318.864	367.737	370.978
Fruit	0.004	0.005	0.007	0.013	545.007	608.086	603.263	673.877
RedMeat	0.009	0.018	0.029	0.062	4,605.060	5,814.219	7,066.457	6,641.323
Poultry	0.005	0.011	0.019	0.022	1,565.126	1,463.999	2,323.772	1,657.256
Eggs	0.001	0.001	0.002	0.005	3,049.641	2,941.884	3,072.327	3,488.583
Fish	0.001	0.002	0.003	0.006	766.375	884.441	1,086.544	1,495.065
Dairy	0.018	0.023	0.031	0.035	1,241.928	1,319.857	2,060.866	1,512.296
Oils	0.028	0.030	0.032	0.035	983.426	1,034.690	1,023.291	1,012.482
Coffee	0.004	0.004	0.004	0.005	1,498.529	1,823.261	1,806.977	1,690.258
SoftDrink	0.001	0.001	0.002	0.007	967.218	1,101.485	1,135.306	1,158.958
OtherFood	0.049	0.035	0.031	0.035	260.541	329.089	355.092	327.054
Food (total)	0.646	0.648	0.626	0.614	287.342	392.486	620.845	1,076.875
Non-food	0.354	0.352	0.374	0.386				
N (total)	2747	4023	3949	2367				
N (rural)	2177	2700	2140	896				
N (urban)	570	1323	1809	1471				

Notes: This table shows the mean value of each food group's share in total expenditures (and the non-food numéraire good's share) in columns 1-4 and each food group's mean unit value (and the non-food numéraire good's median unit value) in columns 5-8. Both budget shares and unit values are summarized by per-capita total expenditures quartile. Mean budget shares and unit values are weighted using survey weights. The unit value for total food is also weighted by budget share. Food groups whose names have been shortened are marked with $^{\mp}$; The food items contained in each food group are listed in Suppl. Table 47.

Suppl. Table 43: Average share in total expenditures and unit value, by food group and consumer total expenditure quartile (Uganda)

	Average Share in Total Expenditures				Average Unit Value (Ush/kg)			
	Q1	Q2	Q3	Q4	Q1	Q2	Q3	Q4
Rice	0.008	0.014	0.015	0.014	2,161.790	2,322.322	2,470.625	2,601.512
Maize	0.076	0.058	0.039	0.019	1,318.455	1,400.177	1,514.690	1,721.361
Wheat	0.040	0.023	0.016	0.009	1,577.497	1,709.052	1,861.234	2,235.266
Cassava	0.082	0.051	0.033	0.011	1,043.369	1,154.466	1,310.100	1,428.040
Roots	0.118	0.116	0.100	0.062	1,182.521	1,345.990	1,506.402	1,716.287
Sugar	0.024	0.026	0.023	0.016	2,725.869	2,819.504	2,891.002	2,973.619
Pulses	0.086	0.065	0.043	0.020	1,879.733	1,887.826	1,916.392	2,190.330
Nuts	0.030	0.028	0.024	0.016	2,284.041	2,457.112	2,634.997	2,948.494
Vegetables	0.056	0.036	0.028	0.017	1,085.187	1,220.480	1,308.991	1,677.607
Fruit	0.016	0.019	0.019	0.015	1,354.964	1,512.094	1,653.149	1,917.954
RedMeat	0.029	0.044	0.050	0.035	5,256.431	5,919.944	6,365.319	6,843.545
Poultry	0.008	0.011	0.012	0.012	5,287.055	6,493.422	7,451.735	8,205.345
Eggs	0.002	0.003	0.003	0.003	5,205.824	5,418.223	5,328.306	5,932.724
Fish	0.024	0.025	0.023	0.016	2,066.671	2,301.457	2,507.731	3,004.500
Dairy	0.016	0.019	0.020	0.017	875.645	942.555	1,003.263	1,165.514
Fats	0.015	0.014	0.012	0.009	3,934.033	4,785.747	5,447.792	7,336.529
Coffee	0.003	0.002	0.002	0.001	2,193.004	2,598.187	2,825.309	3,334.685
SoftDrink	0.001	0.003	0.004	0.006	1,995.697	2,082.537	2,098.779	2,216.920
OtherFood	0.009	0.005	0.003	0.001	747.881	784.755	797.189	841.352
Food (total)	0.643	0.562	0.468	0.300	1,097.135	1,198.445	1,170.235	919.933
Non-food	0.357	0.438	0.532	0.700				
N (total)	5325	3933	3089	2073				
N (rural)	4751	3175	2238	926				
N (urban)	574	758	851	1147				

Notes: This table shows the mean value of each food group's share in total expenditures (and the non-food numéraire good's share) in columns 1-4 and each food group's mean unit value (and the non-food numéraire good's median unit value) in columns 5-8. Both budget shares and unit values are summarized by per-capita total expenditures quartile. Mean budget shares and unit values are weighted using survey weights. The unit value for total food is also weighted by budget share. Food groups whose names have been shortened are marked with ∓; The food items contained in each food group are listed in Suppl. Table 48.

Suppl. Table 44: Average share in total expenditures and unit value, by food group and consumer total expenditure quartile (Tanzania)

	Average Share in Total Expenditures				Average Unit Value (TSH/kg)			
	Q1	Q2	Q3	Q4	Q1	Q2	Q3	Q4
Rice	0.031	0.055	0.069	0.064	1,228.467	1,274.172	1,323.853	1,371.846
Maize	0.208	0.171	0.123	0.064	595.312	702.856	773.873	866.564
Wheat	0.033	0.031	0.030	0.035	1,489.333	1,563.167	1,604.134	1,634.997
Cassava	0.066	0.040	0.022	0.009	398.402	467.742	521.574	606.245
Roots	0.047	0.048	0.049	0.039	551.436	605.850	666.766	766.607
Sugar	0.020	0.029	0.031	0.026	1,703.119	1,804.091	1,818.215	1,793.095
Pulses	0.066	0.052	0.040	0.025	1,244.628	1,298.853	1,348.238	1,456.047
Nuts	0.021	0.017	0.011	0.008	1,557.738	1,728.052	1,872.843	2,027.661
Vegetables	0.101	0.072	0.058	0.045	933.512	957.894	1,020.581	1,081.338
Fruit	0.019	0.027	0.030	0.031	740.767	774.080	833.018	938.750
RedMeat	0.017	0.038	0.055	0.063	3,679.092	3,925.640	4,203.616	4,627.812
Poultry	0.008	0.018	0.023	0.027	3,831.064	4,315.279	4,605.063	5,054.357
Eggs	0.002	0.003	0.004	0.007	2,579.020	2,768.887	2,699.234	2,676.343
Fish	0.032	0.035	0.033	0.030	2,703.553	2,840.958	3,062.765	3,470.994
Dairy	0.015	0.018	0.022	0.023	847.562	997.622	1,170.759	1,466.053
Fats	0.023	0.025	0.025	0.021	2,549.917	2,765.289	2,840.955	2,900.636
Coffee	0.003	0.004	0.005	0.005	8,918.182	9,480.105	9,581.581	9,812.320
SoftDrink	0.002	0.003	0.003	0.002	437.146	495.446	546.281	617.810
OtherFood	0.011	0.007	0.005	0.004	885.539	858.090	852.038	893.737
Food (total)	0.726	0.692	0.638	0.527	807.798	1,004.543	1,127.184	1,163.662
Non-food	0.274	0.308	0.362	0.473				
N (total)	1508	2668	2872	2148				
N (rural)	1357	2106	1841	831				
N (urban)	151	562	1031	1317				

Notes: This table shows the mean value of each food group's share in total expenditures (and the non-food numéraire good's share) in columns 1-4 and each food group's mean unit value (and the non-food numéraire good's median unit value) in columns 5-8. Both budget shares and unit values are summarized by per-capita total expenditures quartile. Mean budget shares and unit values are weighted using survey weights. The unit value for total food is also weighted by budget share. Food groups whose names have been shortened are marked with $^{\mp}$; The food items contained in each food group are listed in Suppl. Table 49.

Suppl. Table 45: Average share in total expenditures and unit value, by food group and consumer total expenditure quartile (Nigeria)

	Average Share in Total Expenditures				Average Unit Value (NGN/kg)			
	Q1	Q2	Q3	Q4	Q1	Q2	Q3	Q4
Rice	0.094	0.088	0.067	0.042	210.598	212.907	214.709	212.501
Maize	0.039	0.028	0.017	0.007	103.846	104.615	105.026	103.606
Wheat	0.122	0.082	0.057	0.036	180.092	186.772	192.078	196.121
Cassava	0.048	0.043	0.034	0.022	114.608	115.193	116.868	117.363
Roots	0.059	0.067	0.064	0.051	104.139	106.523	109.195	115.988
Sugar	0.013	0.011	0.008	0.007	314.046	341.394	359.314	371.265
Pulses	0.049	0.044	0.034	0.023	219.619	227.799	233.515	240.490
Nuts	0.007	0.007	0.006	0.004	441.020	439.037	444.962	445.710
Vegetables	0.072	0.062	0.049	0.037	171.646	172.398	178.463	189.735
Fruit	0.007	0.009	0.012	0.013	113.931	111.529	110.931	112.420
RedMeat	0.041	0.057	0.062	0.049	757.448	769.787	782.080	787.084
Poultry	0.004	0.006	0.012	0.016	793.046	793.691	785.326	782.514
Eggs	0.001	0.002	0.004	0.005	452.005	457.629	464.624	479.729
Fish	0.064	0.066	0.061	0.053	596.452	640.633	674.682	702.611
Dairy	0.009	0.010	0.012	0.016	1,032.546	1,034.263	1,026.712	1,028.114
Oils	0.070	0.053	0.038	0.026	279.591	282.327	284.206	279.287
Coffee	0.002	0.005	0.008	0.010	1,791.513	1,752.238	1,726.166	1,739.829
SoftDrink	0.004	0.007	0.011	0.014	93.587	92.915	92.773	97.915
OtherFood	0.011	0.008	0.005	0.003	278.228	290.249	291.843	284.736
Food (total)	0.716	0.656	0.562	0.435	206.033	218.772	213.702	193.448
Non-food	0.284	0.344	0.438	0.565				
N (total)	6680	8005	7275	4017				
N (rural)	5810	6037	4392	1795				
N (urban)	870	1968	2883	2222				

Notes: This table shows the mean value of each food group's share in total expenditures (and the non-food numéraire good's share) in columns 1-4 and each food group's mean unit value (and the non-food numéraire good's median unit value) in columns 5-8. Both budget shares and unit values are summarized by per-capita total expenditures quartile. Mean budget shares and unit values are weighted using survey weights. The unit value for total food is also weighted by budget share. Food groups whose names have been shortened are marked with $^{\mp}$; The food items contained in each food group are listed in Suppl. Table 50.

Suppl. Table 46: Malawi: Food items contained in each food group

**Food Group Name**	**Food Items Included**
Rice	Rice
Maize	Maize ufa mgaiwa (normal flour); maize ufa refined (fine flour); maize ufa madeya (bran flour); maize grain (not as ufa); green maize
Wheat	Sorghum (mapira); bread; buns and scones; biscuits; spaghetti, macaroni, and pasta
Cassava	Cassava tubers; cassava flour
Roots	White sweet potato; orange sweet potato; Irish potato; plantain and cooking banana
Sugar	Sugar; sugar cane
Pulses	White bean; brown bean; pigeon pea (nandolo); soybean flour; ground bean (nzama); cowpea (khobwe)
Nuts	Groundnut flour
Vegetables	Onion; cabbage; nkhwani; Chinese cabbage; gathered wild green leaves; tomato; cucumber; pumpkin; okra/therere
Fruit	Mango; banana; papaya; guava; avocado; wild fruit (masau, malambe, etc.)
Red Meat	Beef; goat; pork
Poultry	Chicken
Eggs	Eggs
Dairy	Fresh milk; powdered milk; margarine (Blue Band)
Fats	Cooking oil
Tea	Tea
Soft Drink	Squash (sobo drink concentrate); fruit juice; freezes (flavored ice); soft drinks (coca-cola, Fanta, Sprite, etc.)
Other Food	Salt; spices; yeast, baking powder and bicarbonate of soda

Note: This table shows how individual food items listed in the Malawi survey are mapped onto food groups. Food items are separated by semicolons.

Suppl. Table 47: Niger: Food items contained in each food group

Food Group Name	Food Items Included
Rice	Rice
Millet	Millet
Wheat	Wheat flour; sorghum; fonio; other grains; cornstarch; pasta; bread; biscuit; corn fritters; cakes; other pastries (cakes, pastries)
Cassava	Cassava flour (attiéké, gari, tapioca, etc.); cassava tuber
Roots	Yam tuber; potato; taro and cocoyam; sweet potato; other tubers
Sugar	Sugar; honey; confectionery
Pulses	Bean fritters; beans; dry pea; other pulses; peanut butter
Nuts	Bambara groundnut; peanuts in shell; shelled peanuts; groundnut cake; cola nut
Vegetables	Salad (lettuce); fresh onion; fresh okra; fresh tomato; fresh pepper; eggplant; carrot; green bean; cucumber; pea; squash, zucchini; other vegetable; dried tomato; dried okra; tomato paste; baobab leaves; other leafy vegetables
Fruit	Mango; pineapple; orange; other citrus (mandarin, lemon, grapefruit); sweet banana; watermelon; dates; sugar cane; melon; palmyra/doumier (African fan palm fruit); other fruits
Red Meat	Beef; camel meat; mutton; goat meat; game; other meats
Poultry	Poultry; giblets
Eggs	Eggs
Fish	Fresh fish; smoked fish; stockfish; canned fish; other canned fish products
Dairy	Fresh milk; curd; powdered milk; cheese; yogurt; other dairy products
Oils	Palm oil; peanut oil; cottonseed oil; corn oil; other oils (soy, sheabutter); butter
Coffee	Cocoa/chocolate; coffee in cans; tea bag; other teas
Soft Drink	Fruit juice; juice powder; soft drinks
Other Food	Maggi cube; soumbala (of sorrel); yodo; malahya; salt; pimento; other spices (ginger, garlic, etc.)

Note: This table shows how individual food items listed in the Niger survey are mapped onto food groups. Food items are separated by semicolons.

Suppl. Table 48: Uganda: Food items contained in each food group

**Food Group Name**	**Food Items Included**
Rice	Rice
Maize	Maize (grains); maize (cobs); maize (flour)
Wheat	Bread; millet (flour); sorghum (flour); wheat (flour); chapati; loaf; bun
Cassava	Cassava (dry); cassava (flour)
Roots	Plantains (matooke), bunch; plantains (matooke), cluster; plantains (matooke), heap; plantains (matooke), other units; sweet potatoes (fresh); sweet potatoes (dry); cassava (fresh); cassava (dry/flour); Irish potatoes; sweet potato flour
Sugar	Sugar
Pulses	Beans (fresh); beans (dry)
Nuts	Ground nuts (in shell); ground nut seeds (unshelled); ground nuts (pounded); peas (fresh); simsim; peas (dry); ground nut paste
Vegetables	Onions; tomatoes; cabbages; dodo; other vegetables; green pepper; pumpkins; carrots; eggplant
Fruit	Passion fruits; sweet bananas; mangoes; oranges; other fruits; avocado; pineapple; pawpaw; apples; watermelon
Red Meat	Beef; pork; goat meat; other meat
Poultry	Chicken (local)
Eggs	Eggs
Fish	Fresh fish; dry/smoked fish
Dairy	Fresh milk; infant formula food
Fats	Cooking oil; ghee margarine, butter, etc
Coffee	Coffee; tea
Soft Drink	Soda; other drinks
Other Food	Salt

Note: This table shows how individual food items listed in the Uganda survey are mapped onto food groups. Food items are separated by semicolons.

Suppl. Table 49: Tanzania: Food items contained in each food group

Food Group Name	Food Items Included
Rice	Rice (paddy); rice (husked)
Maize	Maize (grain); maize (flour)
Wheat	Millet and sorghum (grain); millet and sorghum (flour); wheat, barley grain and other cereals; bread; buns, cakes and biscuits; macaroni and spaghetti; other cereal products
Cassava	Cassava fresh; cassava dry / flour
Roots	Sweet potatoes; yams / cocoyams; Irish potatoes; cooking bananas, plantains; other starches
Sugar	Sugar; sweets; honey, syrups, jams, marmalade, jellies
Pulses	Peas, beans, lentils and other pulses
Nuts	Ground-nuts in shell / shelled; Cashew, almond and other nuts
Vegetables	Onions, tomatoes, carrots and green pepper; spinach, cabbage and other green vegetable; canned, dried and wild vegetables
Fruit	Coconuts (mature / immature); ripe bananas; citrus fruits; mangoes, avocados and other fruits
Red Meat	Goat meat; beef including minced sausage; pork including sausages and bacon
Poultry	Chicken and other poultry
Eggs	Eggs
Fish	Fresh fish and seafood (including dagaa); Dried / salted / canned fish and seafood (incl. dagaa)
Dairy	Fresh milk; Milk products (like cream, cheese, yoghurt, etc); Canned milk / milk powder
Oils	Cooking oil; Butter, margarine, ghee and other fat products
Coffee	Tea dry; Coffee and cocoa
Soft Drink	Sugar-cane; Other raw materials for drinks
Other Food	Salt; other spices

Note: This table shows how individual food items listed in the Tanzania survey are mapped onto food groups. Food items are separated by semicolons.

Suppl. Table 50: Nigeria: Food items contained in each food group

Food Group Name	Food Items Included
Rice	Rice, local; rice, imported
Maize	Maize; maize flour
Wheat	Guinea corn/sorghum; millet; bread; yam flour; wheat flour; other grains and flour
Cassava	Cassava flour; cassava (roots); gari (white); gari (yellow)
Roots	Yam (roots); cocoyam; plantains; sweet potatoes; potatoes
Sugar	Sugar; honey
Pulses	Soya beans; brown beans; white beans
Nuts	Groundnuts; other nuts/seeds/pulses
Vegetables	Tomatoes; tomato puree (canned); onions; garden eggplant; okra (fresh); okra (dried); pepper; leaves (cocoyam, spinach, etc.); other vegetables
Fruit	Bananas; orange/tangerine; mangoes; avocado; pear; pineapple; other fruits
Red Meat	Beef; mutton; pork; goat; wild game meat; other meat (excl. poultry)
Poultry	Chicken
Eggs	Agricultural eggs; local eggs
Fish	Fish (fresh); fish (frozen); fish (smoked); fish (dried); snails; seafood (lobster, crab, prawns)
Dairy	Fresh milk; milk powder; baby milk powder; tinned milk (unsweetened); other milk products
Oils	Palm oil; butter/margarine; groundnuts oil; other oil and fat
Coffee	Coffee; chocolate drinks (including Milo); tea
Soft Drink	Malt drinks; soft drinks (coca cola, spirit, etc); fruit juice canned/pack
Other Food	Condiments (salt, spices, pepper)

Note: This table shows how individual food items listed in the Nigeria survey are mapped onto food groups. Food items are separated by semicolons.

Suppl. Table 51: Summary statistics of baseline per capita kcal daily intake for Malawi households

	Obs.	Mean	Median	Std. dev.	Min	Max
Rice	7922	119.2274	0.0000	197.3364	0	1023
Maize	7922	2129.9597	1988.2274	1145.6306	0	8620
Wheat	7922	113.8472	0.0000	188.1407	0	2242
Cassava	7922	99.3202	0.0000	287.3284	0	4357
Roots	7922	129.7197	86.9400	153.5088	0	1361
Sugar	7922	146.2463	143.9032	120.6446	0	622
Pulses	7922	182.9135	129.3436	198.5998	0	1559
Nuts	7922	18.0835	0.0000	30.3504	0	190
Vegetables	7922	37.1558	22.3493	45.5764	0	386
Fruit	7922	49.5606	19.0341	106.3784	0	1208
Red meat	7922	45.8366	0.0000	81.6096	0	899
Poultry	7922	25.0889	0.0000	48.4482	0	240
Eggs	7922	59.0461	0.0000	139.0362	0	821
Dairy	7922	19.6915	0.0000	52.4833	0	531
Oils	7922	125.4994	96.7551	132.7077	0	657
Tea	7922	0.0000	0.0000	0.0000	0	0
Soft drink	7922	16.6784	0.0000	48.5118	0	557
Other food	7922	0.7978	0.0000	2.9870	0	37
Observations	7922					

Suppl. Table 52: Summary statistics of baseline per capita kcal daily intake for Niger households

	Obs.	Mean	Median	Std. dev.	Min	Max
Rice	13026	1278.5298	409.5238	29091.6724	0	2064000
Millet	13026	2796.7293	1577.5862	30366.5488	0	3267857
Wheat	13026	1656.3955	704.0816	12085.6825	0	639286
Cassava	13026	186.7228	0.0000	4618.3873	0	496503
Roots	13026	45.5800	0.0000	235.1935	0	11211
Sugar	13026	822.1403	50.7947	70805.5195	0	8067227
Pulses	13026	424.0799	0.0000	7478.2148	0	376979
Nuts	13026	127.7862	0.0000	4690.8573	0	488699
Vegetables	13026	146.8196	28.3956	2358.5554	0	193954
Fruit	13026	61.7323	0.0000	2129.8883	0	187435
Red meat	13026	285.5265	0.0000	7939.9702	0	543182
Poultry	13026	22.9634	0.0000	118.4931	0	7741
Eggs	13026	2.5453	0.0000	15.5948	0	618
Fish	13026	17.7168	0.0000	513.9434	0	49500
Dairy	13026	242.4877	0.0000	6162.6152	0	586905
Oils	13026	812.1010	235.9664	13239.2085	0	786555
Coffee	13026	3.9377	0.0000	78.2401	0	8132
Soft drink	13026	16.9258	0.0000	1209.7699	0	135714
Other food	13026	213.0883	109.4282	1651.0915	0	109615
Observations	13026					

Suppl. Table 53: Summary statistics of baseline per capita kcal daily intake for Uganda households

	Obs.	Mean	Median	Std. dev.	Min	Max
Rice	14420	76.0405	0.0000	194.5289	0	8571
Maize	14420	450.6351	173.9286	862.9230	0	29615
Wheat	14420	260.3953	0.0000	648.1499	0	10378
Cassava	14420	355.9780	67.6571	1644.9322	0	128821
Roots	14420	362.4900	133.4775	770.6267	0	45768
Sugar	14420	124.6477	79.1414	277.8456	0	20226
Pulses	14420	345.4692	193.2775	479.9681	0	8952
Nuts	14420	415.6451	108.3133	930.9314	0	29160
Vegetables	14420	37.6286	14.8294	59.3462	0	1154
Fruit	14420	57.7958	0.0000	207.1454	0	9806
Red meat	14420	57.5228	0.0000	230.9994	0	25233
Poultry	14420	15.1816	0.0000	60.7547	0	1807
Eggs	14420	28.5533	0.0000	142.1939	0	5091
Fish	14420	127.3542	0.0000	307.1592	0	6584
Dairy	14420	63.3725	0.0000	148.1272	0	3120
Fats	14420	987.9540	273.6190	1952.5758	0	52535
Coffee	14420	0.0806	0.0000	2.2560	0	260
Soft drink	14420	6.2807	0.0000	34.9848	0	1410
Other food	14420	0.0000	0.0000	0.0000	0	0
Observations	14420					

Suppl. Table 54: Summary statistics of baseline per capita kcal daily intake for Tanzania households

	Obs.	Mean	Median	Std. dev.	Min	Max
Rice	9014	503.0078	340.9524	593.0574	0	7755
Maize	9014	1152.9552	898.7918	1105.0960	0	9309
Wheat	9014	257.7145	82.9838	449.6229	0	7109
Cassava	9014	264.2915	0.0000	623.6213	0	5566
Roots	9014	137.9607	45.7433	242.7711	0	2914
Sugar	9014	152.9688	125.6494	164.9425	0	3046
Pulses	9014	208.1630	136.5714	243.3896	0	1711
Nuts	9014	70.2944	0.0000	190.0052	0	2526
Vegetables	9014	40.9177	33.5641	31.2824	0	234
Fruit	9014	124.6927	38.8094	183.6224	0	1714
Red meat	9014	92.3150	0.0000	170.8937	0	2210
Poultry	9014	24.4277	0.0000	67.4324	0	728
Eggs	9014	27.1022	0.0000	131.5184	0	2904
Fish	9014	61.7844	33.0833	84.8937	0	806
Dairy	9014	57.8227	0.0000	177.9941	0	4239
Fats	9014	171.6015	135.4571	170.4976	0	1506
Coffee	9014	0.3334	0.0093	3.8445	0	192
Soft drink	9014	3.2273	0.0000	14.0783	0	268
Other food	9014	5.5737	0.0000	23.6332	0	430
Observations	9014					

Suppl. Table 55: Summary statistics of baseline per capita kcal daily intake for Nigeria households

	Obs.	Mean	Median	Std. dev.	Min	Max
Rice	25455	409.1372	344.9495	348.1067	0	3794
Maize	25455	271.2280	0.0000	523.3536	0	4712
Wheat	25455	683.8091	196.2561	1005.9331	0	8079
Cassava	25455	210.9082	95.1146	388.5984	0	4943
Roots	25455	244.6058	148.3044	301.6948	0	2129
Sugar	25455	39.5120	2.8155	67.4650	0	953
Pulses	25455	199.2072	159.5168	209.8822	0	2524
Nuts	25455	28.5793	0.0000	89.4264	0	1604
Vegetables	25455	35.8859	29.1850	30.3195	0	334
Fruit	25455	20.2215	0.0000	44.7210	0	615
Red meat	25455	54.0248	34.1429	67.3298	0	669
Poultry	25455	9.2419	0.0000	35.2929	0	327
Eggs	25455	3.0692	0.0000	9.5261	0	137
Fish	25455	37.6061	23.1460	50.1577	0	570
Dairy	25455	17.1094	0.0000	41.4201	0	632
Oils	25455	379.5125	329.8528	266.4261	0	2124
Coffee	25455	0.7936	0.0000	2.5317	0	37
Soft drink	25455	9.7629	0.0000	29.1550	0	523
Other food	25455	19.0285	12.1992	24.4302	0	225
Observations	25455					

Suppl. Table 56: Malawi: Results from first stage instrumental variable regressions

	p1	p2	p3	p4	p5	p6	p7	p8	p9	p10	p11	p12	p13	p14	p15	p16	p17	p18	p19
IV_p1	0.96**	0.03	0.02	0.00	0.02	-0.00	-0.02	-0.03	0.05*	0.02	-0.00	0.00	0.12**	-0.01	0.01	-0.03	0.00	0.00	-0.00
	(0.01)	(0.02)	(0.02)	(0.02)	(0.02)	(0.01)	(0.02)	(0.02)	(0.02)	(0.03)	(0.01)	(0.01)	(0.04)	(0.02)	(0.02)	(0.03)	(0.02)	(0.05)	(0.00)
IV_p2	0.00	0.99**	0.00	0.01	0.01	-0.00	0.01	-0.00	0.01	0.01	0.00	-0.01	0.08**	0.00	0.00	0.01	0.02	-0.00	-0.00
	(0.01)	(0.01)	(0.01)	(0.01)	(0.01)	(0.01)	(0.01)	(0.01)	(0.01)	(0.02)	(0.00)	(0.01)	(0.02)	(0.01)	(0.01)	(0.02)	(0.01)	(0.03)	(0.00)
IV_p3	0.00	-0.01	0.89**	0.00	0.00	0.01	-0.02	0.01	0.01	-0.01	0.00	-0.00	-0.01	-0.00	-0.00	0.01	0.03	-0.01	0.01**
	(0.01)	(0.02)	(0.02)	(0.02)	(0.02)	(0.01)	(0.02)	(0.02)	(0.02)	(0.03)	(0.01)	(0.01)	(0.04)	(0.02)	(0.02)	(0.02)	(0.02)	(0.04)	(0.00)
IV_p4	0.00	-0.00	0.00	0.97**	0.02	0.02*	-0.01	0.03	0.00	0.01	-0.00	-0.00	0.04	0.00	-0.02	0.01	0.01	-0.03	0.01**
	(0.01)	(0.02)	(0.02)	(0.01)	(0.01)	(0.01)	(0.02)	(0.02)	(0.02)	(0.02)	(0.01)	(0.01)	(0.03)	(0.01)	(0.02)	(0.02)	(0.02)	(0.03)	(0.00)
IV_p5	0.01	0.01	-0.00	0.00	0.95**	0.00	0.01	0.01	0.02	0.01	0.01	0.00	-0.06	0.01	-0.02	0.00	-0.04	0.06	-0.00*
	(0.01)	(0.02)	(0.02)	(0.02)	(0.02)	(0.01)	(0.02)	(0.02)	(0.02)	(0.03)	(0.01)	(0.01)	(0.03)	(0.02)	(0.02)	(0.02)	(0.02)	(0.04)	(0.00)
IV_p6	-0.02	-0.07	0.00	0.02	-0.00	0.85**	-0.01	0.00	-0.04	-0.05	0.02	-0.01	-0.07	-0.00	0.14**	-0.07	-0.09	0.12	0.06**
	(0.03)	(0.04)	(0.04)	(0.04)	(0.04)	(0.02)	(0.05)	(0.04)	(0.05)	(0.06)	(0.02)	(0.03)	(0.08)	(0.04)	(0.05)	(0.06)	(0.05)	(0.10)	(0.00)
IV_p7	-0.01	0.00	-0.00	-0.01	-0.01	-0.00	0.92**	-0.01	0.03	0.03	-0.00	-0.00	-0.01	-0.01	-0.00	-0.02	-0.02	-0.01	0.00*
	(0.01)	(0.02)	(0.02)	(0.01)	(0.02)	(0.01)	(0.02)	(0.02)	(0.02)	(0.03)	(0.01)	(0.01)	(0.03)	(0.02)	(0.02)	(0.02)	(0.02)	(0.04)	(0.00)
IV_p8	0.00	0.00	-0.00	0.00	0.00	-0.00	0.00	1.00**	0.01	0.01	0.00	-0.00	0.03	-0.00	-0.01	0.01	-0.00	0.06**	-0.00
	(0.00)	(0.01)	(0.01)	(0.01)	(0.01)	(0.00)	(0.01)	(0.01)	(0.01)	(0.01)	(0.00)	(0.00)	(0.01)	(0.01)	(0.01)	(0.01)	(0.01)	(0.02)	(0.00)
IV_p9	0.02	-0.00	0.03	0.01	0.02	0.01	0.04	0.00	0.87**	-0.05	-0.01	0.01	-0.09	0.01	0.02	-0.04	0.09**	0.03	-0.01**
	(0.02)	(0.02)	(0.02)	(0.02)	(0.02)	(0.01)	(0.03)	(0.02)	(0.03)	(0.03)	(0.01)	(0.02)	(0.04)	(0.02)	(0.03)	(0.03)	(0.03)	(0.05)	(0.00)
IV_p10	-0.00	-0.01	0.00	0.01	0.01	-0.00	0.01	-0.01	-0.01	0.95**	0.00	-0.01	0.06*	0.01	-0.00	0.00	0.01	0.03	-0.00
	(0.01)	(0.01)	(0.01)	(0.01)	(0.01)	(0.01)	(0.01)	(0.01)	(0.02)	(0.02)	(0.00)	(0.01)	(0.02)	(0.01)	(0.01)	(0.02)	(0.01)	(0.03)	(0.00)
IV_p11	0.01	0.03	-0.01	-0.01	0.02	0.04*	0.01	0.01	-0.03	0.05	0.97**	0.00	0.13*	0.02	-0.04	0.05	-0.03	-0.00	0.02**
	(0.02)	(0.03)	(0.03)	(0.03)	(0.03)	(0.02)	(0.03)	(0.03)	(0.04)	(0.04)	(0.01)	(0.02)	(0.06)	(0.03)	(0.03)	(0.04)	(0.03)	(0.07)	(0.00)
IV_p12	0.00	0.00	0.01	-0.01	0.00	0.01	0.01	-0.01	0.01	-0.01	0.00	1.00**	0.11**	-0.01	-0.01	-0.01	0.00	0.03	-0.00**
	(0.01)	(0.01)	(0.01)	(0.01)	(0.01)	(0.01)	(0.01)	(0.01)	(0.01)	(0.02)	(0.00)	(0.01)	(0.02)	(0.01)	(0.01)	(0.01)	(0.01)	(0.03)	(0.00)
IV_p13	0.01	-0.00	0.01	-0.01	-0.01	-0.00	-0.01	0.00	-0.01	0.00	0.00	-0.00	0.42**	-0.01	0.01	0.03*	0.00	0.01	0.00**
	(0.01)	(0.01)	(0.01)	(0.01)	(0.01)	(0.00)	(0.01)	(0.01)	(0.01)	(0.01)	(0.00)	(0.01)	(0.02)	(0.01)	(0.01)	(0.01)	(0.01)	(0.02)	(0.00)
IV_p14	-0.00	0.01	0.01	0.00	-0.00	-0.00	-0.00	-0.01	-0.00	0.00	0.00	-0.00	0.01	0.99**	0.02	-0.00	0.02	0.00	0.00*
	(0.01)	(0.01)	(0.01)	(0.01)	(0.01)	(0.01)	(0.01)	(0.01)	(0.01)	(0.02)	(0.00)	(0.01)	(0.02)	(0.01)	(0.01)	(0.02)	(0.01)	(0.03)	(0.00)
IV_p15	0.00	0.01	-0.02	-0.02	-0.02	0.03**	-0.00	-0.02	-0.01	0.02	-0.00	-0.00	0.11**	0.00	0.94**	0.00	0.00	0.04	0.00**
	(0.01)	(0.02)	(0.02)	(0.01)	(0.02)	(0.01)	(0.02)	(0.02)	(0.02)	(0.02)	(0.01)	(0.01)	(0.03)	(0.01)	(0.02)	(0.02)	(0.02)	(0.04)	(0.00)
IV_p16	-0.00	0.00	-0.00	0.01	0.00	-0.00	-0.01	0.00	0.00	0.00	0.00	0.00	0.00	-0.01	-0.01	1.00**	0.00	-0.02	-0.00
	(0.01)	(0.01)	(0.01)	(0.01)	(0.01)	(0.00)	(0.01)	(0.01)	(0.01)	(0.01)	(0.00)	(0.01)	(0.02)	(0.01)	(0.01)	(0.01)	(0.01)	(0.02)	(0.00)
IV_p17	0.00	0.01	-0.01	0.01	-0.01	0.00	-0.01	0.00	-0.01	0.03	0.00	0.01	-0.01	-0.00	0.01	0.00	1.01**	0.01	0.00**
	(0.01)	(0.01)	(0.01)	(0.01)	(0.01)	(0.01)	(0.01)	(0.01)	(0.01)	(0.02)	(0.00)	(0.01)	(0.02)	(0.01)	(0.01)	(0.01)	(0.01)	(0.03)	(0.00)
IV_p18	-0.01	-0.00	0.00	0.01	0.01	0.00	-0.01	0.02	-0.00	0.01	-0.00	-0.01	0.07	0.01	0.01	-0.01	0.01	0.58**	0.00
	(0.01)	(0.02)	(0.02)	(0.02)	(0.02)	(0.01)	(0.02)	(0.02)	(0.03)	(0.03)	(0.01)	(0.01)	(0.04)	(0.02)	(0.02)	(0.03)	(0.02)	(0.05)	(0.00)
IV_p19	0.05	0.01	0.05	0.01	-0.02	0.09**	0.09	0.00	0.08	-0.03	0.00	0.01	0.08	-0.01	-0.08	0.09	-0.01	-0.04	0.89**
	(0.04)	(0.06)	(0.06)	(0.05)	(0.05)	(0.03)	(0.06)	(0.06)	(0.07)	(0.08)	(0.02)	(0.04)	(0.11)	(0.05)	(0.06)	(0.07)	(0.06)	(0.12)	(0.00)
F-stats	2,576.11	1,283.81	663.67	974.48	1,244.36	3,844.02	890.78	839.58	669.79	229.16	4,695.65	2,467.59	1,337.40	643.07	487.79	370.76	667.17	107.32	180,809.00
p-value	0.00	0.00	0.00	0.00	0.00	0.00	0.00	0.00	0.00	0.00	0.00	0.00	0.00	0.00	0.00	0.00	0.00	0.00	0.00
Obs.	8,089	8,089	8,089	8,089	8,089	8,089	8,089	8,089	8,089	8,089	8,089	8,089	8,089	8,089	8,089	8,089	8,089	8,089	8,089

Note: p_j denotes the price for food group j, and IV_p_j denotes the instrument for p_j, which is based on the average price index of neighboring households. Due to space limits, we report the coefficients on price instruments only. The regressions also include the vector of demand shifters. The F-stat checks for joint significance of all price instruments in a two-sided test. We report standard errors in parentheses.

Suppl. Table 57: Niger: Results from first stage instrumental variable regressions

	p1	p2	p3	p4	p5	p6	p7	p8	p9	p10	p11	p12	p13	p14	p15	p16	p17	p18	p19	p20
IV_p1	0.25**	0.07	0.02	-0.02	0.01	0.17	-0.03	0.03	0.01	0.10	-0.42**	0.02	0.06*	-0.01	0.61**	0.13	0.08	-0.02	0.13	-0.00**
	(0.05)	(0.06)	(0.11)	(0.06)	(0.04)	(0.10)	(0.07)	(0.04)	(0.07)	(0.06)	(0.06)	(0.06)	(0.02)	(0.04)	(0.07)	(0.08)	(0.07)	(0.02)	(0.09)	(0.00)
IV_p2	0.47*	1.08**	-0.91	0.35	0.12	-0.17	0.77*	0.29	0.04	-0.11	0.15	-0.71**	-0.10	0.04	-0.19	-0.15	-0.12	0.04	0.02	0.00**
	(0.22)	(0.26)	(0.47)	(0.24)	(0.16)	(0.41)	(0.30)	(0.19)	(0.32)	(0.24)	(0.24)	(0.24)	(0.10)	(0.16)	(0.32)	(0.34)	(0.29)	(0.10)	(0.37)	(0.00)
IV_p3	0.01*	-0.02**	0.62**	-0.00	-0.01**	0.03**	-0.01*	0.01	0.03**	-0.01*	0.02**	0.10**	-0.01**	-0.01	-0.06**	-0.02*	0.00	-0.00	0.01	0.00**
	(0.01)	(0.01)	(0.01)	(0.01)	(0.00)	(0.01)	(0.01)	(0.00)	(0.01)	(0.01)	(0.01)	(0.01)	(0.00)	(0.00)	(0.01)	(0.01)	(0.01)	(0.00)	(0.01)	(0.00)
IV_p4	0.07**	0.08**	0.08**	1.06**	-0.00	0.01	-0.13**	-0.00	0.03**	-0.02*	0.02*	-0.06**	0.01*	0.00	-0.05**	0.01	-0.04**	0.00	-0.00	-0.00**
	(0.01)	(0.01)	(0.01)	(0.01)	(0.00)	(0.01)	(0.01)	(0.01)	(0.01)	(0.01)	(0.01)	(0.01)	(0.00)	(0.00)	(0.01)	(0.01)	(0.01)	(0.00)	(0.01)	(0.00)
IV_p5	0.01	0.01	0.03	0.00	0.98**	-0.01	-0.04**	-0.02*	0.02	-0.02*	-0.03**	-0.01	0.01*	-0.01*	0.04**	-0.04**	-0.01	-0.00	-0.02	-0.00**
	(0.01)	(0.01)	(0.02)	(0.01)	(0.01)	(0.02)	(0.01)	(0.01)	(0.01)	(0.01)	(0.01)	(0.01)	(0.00)	(0.01)	(0.01)	(0.01)	(0.01)	(0.00)	(0.01)	(0.00)
IV_p6	-0.05**	0.01	-0.11**	-0.04**	-0.01	0.84**	0.11**	0.01	-0.01	0.01	0.03**	-0.02**	-0.00	-0.00	0.07**	0.06**	-0.02*	-0.00	0.04**	-0.00
	(0.01)	(0.01)	(0.01)	(0.01)	(0.00)	(0.01)	(0.01)	(0.01)	(0.01)	(0.01)	(0.01)	(0.01)	(0.00)	(0.00)	(0.01)	(0.01)	(0.01)	(0.00)	(0.01)	(0.00)
IV_p7	-0.03	-0.10**	-0.02	0.01	-0.01	0.07	1.46**	0.03	0.03	-0.07*	-0.07*	0.39**	-0.02	-0.01	-0.05	0.04	-0.02	0.02	-0.08	-0.00
	(0.03)	(0.03)	(0.05)	(0.03)	(0.02)	(0.05)	(0.04)	(0.02)	(0.04)	(0.03)	(0.03)	(0.03)	(0.01)	(0.02)	(0.04)	(0.04)	(0.03)	(0.01)	(0.04)	(0.00)
IV_p8	-0.02**	0.03**	0.01	0.01	-0.01	0.00	-0.00	0.98**	0.02**	0.01	0.04**	0.05**	-0.00	-0.00	-0.06**	0.02**	0.02**	-0.00	-0.01	0.00**
	(0.01)	(0.01)	(0.01)	(0.01)	(0.00)	(0.01)	(0.01)	(0.00)	(0.01)	(0.01)	(0.01)	(0.01)	(0.00)	(0.00)	(0.01)	(0.01)	(0.01)	(0.00)	(0.01)	(0.00)
IV_p9	-0.07**	-0.01	0.04	0.04**	0.00	0.01	0.12**	-0.01	0.87**	0.02*	-0.06**	-0.10**	-0.00	-0.00	0.13**	-0.01	0.03*	-0.00	0.12**	-0.00**
	(0.01)	(0.01)	(0.02)	(0.01)	(0.01)	(0.02)	(0.02)	(0.01)	(0.02)	(0.01)	(0.01)	(0.01)	(0.01)	(0.01)	(0.02)	(0.02)	(0.01)	(0.01)	(0.02)	(0.00)
IV_p10	0.01*	0.03**	0.08**	0.01	-0.00	-0.00	-0.03**	0.01	0.00	0.93**	-0.03**	0.04**	-0.00	-0.02**	-0.03**	0.00	0.00	-0.00	0.03*	-0.00*
	(0.01)	(0.01)	(0.01)	(0.01)	(0.00)	(0.01)	(0.01)	(0.01)	(0.01)	(0.01)	(0.01)	(0.01)	(0.00)	(0.00)	(0.01)	(0.01)	(0.01)	(0.00)	(0.01)	(0.00)
IV_p11	-0.01	-0.01	0.08**	-0.01	-0.00	0.00	0.01	-0.01	-0.02**	0.01	0.80**	-0.04**	0.01**	-0.00	0.03**	0.03**	0.01	-0.00	-0.00	-0.00*
	(0.01)	(0.01)	(0.01)	(0.01)	(0.00)	(0.01)	(0.01)	(0.01)	(0.01)	(0.01)	(0.01)	(0.01)	(0.00)	(0.00)	(0.01)	(0.01)	(0.01)	(0.00)	(0.01)	(0.00)
IV_p12	0.01	0.00	0.06**	0.01	-0.01	-0.02	-0.01	0.01	-0.01	-0.00	0.01	0.77**	-0.02**	0.00	0.05**	-0.03	0.00	-0.00	0.04*	0.00**
	(0.01)	(0.01)	(0.02)	(0.01)	(0.01)	(0.02)	(0.02)	(0.01)	(0.02)	(0.01)	(0.01)	(0.01)	(0.01)	(0.01)	(0.02)	(0.02)	(0.01)	(0.01)	(0.02)	(0.00)
IV_p13	0.01	0.01	-0.11**	0.01	0.00	0.03*	0.09**	-0.00	0.02	-0.00	0.04**	0.08**	0.94**	-0.01*	0.02	0.01	-0.00	0.00	0.06**	0.00**
	(0.01)	(0.01)	(0.02)	(0.01)	(0.01)	(0.02)	(0.01)	(0.01)	(0.01)	(0.01)	(0.01)	(0.01)	(0.00)	(0.01)	(0.01)	(0.01)	(0.01)	(0.00)	(0.01)	(0.00)
IV_p14	0.02**	0.04**	-0.05**	0.00	0.01*	0.02	0.05**	-0.00	0.02**	0.00	0.00	0.01**	-0.01**	1.02**	0.03**	0.01	0.03**	0.00	0.02**	-0.00
	(0.00)	(0.01)	(0.01)	(0.00)	(0.00)	(0.01)	(0.01)	(0.00)	(0.01)	(0.01)	(0.01)	(0.01)	(0.00)	(0.00)	(0.01)	(0.01)	(0.01)	(0.00)	(0.01)	(0.00)
IV_p15	-0.04**	0.00	0.00	-0.01	-0.00	-0.00	-0.02**	-0.01	0.02	0.02**	0.02**	0.01*	0.00	0.01*	0.73**	0.08**	0.01	-0.00	-0.02*	0.00**
	(0.01)	(0.01)	(0.01)	(0.01)	(0.00)	(0.01)	(0.01)	(0.01)	(0.01)	(0.01)	(0.01)	(0.01)	(0.00)	(0.00)	(0.01)	(0.01)	(0.01)	(0.00)	(0.01)	(0.00)
IV_p16	-0.03	0.11**	0.17**	-0.01	0.00	0.09**	0.02	0.01	-0.02	0.05**	0.03	0.03	0.04**	-0.01	0.00	0.64**	-0.01	0.00	0.07*	0.00
	(0.02)	(0.02)	(0.03)	(0.02)	(0.01)	(0.03)	(0.02)	(0.01)	(0.02)	(0.02)	(0.02)	(0.02)	(0.01)	(0.01)	(0.02)	(0.02)	(0.02)	(0.01)	(0.03)	(0.00)
IV_p17	0.02**	0.02**	-0.02*	0.01**	-0.00	0.02**	0.07**	-0.00	0.01	0.00	-0.01	-0.05**	-0.00	-0.00	0.07**	0.03**	0.95**	-0.00	0.00	0.00
	(0.00)	(0.01)	(0.01)	(0.00)	(0.00)	(0.01)	(0.01)	(0.00)	(0.01)	(0.00)	(0.00)	(0.00)	(0.00)	(0.00)	(0.01)	(0.01)	(0.01)	(0.00)	(0.01)	(0.00)
IV_p18	0.00	-0.05**	0.18**	0.01	0.02**	0.02	0.01	0.02**	0.01	0.03**	-0.00	0.01	0.04**	-0.01	-0.03*	0.04**	-0.07**	0.99**	0.12**	-0.00**
	(0.01)	(0.01)	(0.02)	(0.01)	(0.01)	(0.02)	(0.01)	(0.01)	(0.01)	(0.01)	(0.01)	(0.01)	(0.00)	(0.01)	(0.01)	(0.01)	(0.01)	(0.00)	(0.02)	(0.00)
IV_p19	0.08**	0.08**	-0.03	-0.01	0.00	0.00	0.07**	-0.00	0.02	-0.04**	0.01	0.07**	-0.01*	0.00	-0.04**	0.08**	-0.04**	-0.00	0.83**	0.00
	(0.01)	(0.01)	(0.02)	(0.01)	(0.01)	(0.02)	(0.01)	(0.01)	(0.01)	(0.01)	(0.01)	(0.01)	(0.00)	(0.01)	(0.01)	(0.01)	(0.01)	(0.00)	(0.01)	(0.00)
IV_p20	0.25	2.77	-10.68*	0.31	1.47	-0.90	6.84*	3.64	6.21	1.21	2.48	-3.77	-0.57	0.88	-19.40**	3.81	5.65	-0.91	3.17	-0.33**
	(2.37)	(2.77)	(4.98)	(2.49)	(1.64)	(4.36)	(3.21)	(2.02)	(3.33)	(2.58)	(2.55)	(2.54)	(1.11)	(1.67)	(3.38)	(3.63)	(3.09)	(1.07)	(3.95)	(0.00)
F-stats	34.22	59.31	823.06	1,887.53	546.53	328.71	123.68	2,591.69	188.43	416.02	1,756.54	324.20	1,346.15	3,850.48	195.23	72.18	2,678.51	2,145.08	272.67	22,361.30
p-value	0.00	0.00	0.00	0.00	0.00	0.00	0.00	0.00	0.00	0.00	0.00	0.00	0.00	0.00	0.00	0.00	0.00	0.00	0.00	0.00
Obs.	13,086	13,086	13,086	13,086	13,086	13,086	13,086	13,086	13,086	13,086	13,086	13,086	13,086	13,086	13,086	13,086	13,086	13,086	13,086	13,086

Note: p_j denotes the price for food group j, and IV_p_j denotes the instrument for p_j, which is based on the average price index of neighboring households. Due to space limits, we report the coefficients on price instruments only. TThe regressions also include the vector of demand shifters. The F-stat checks for joint significance of all price instruments in a two-sided test. We report standard errors in parentheses.

Suppl. Table 58: Uganda: Results from first stage instrumental variable regressions

	p1	p2	p3	p4	p5	p6	p7	p8	p9	p10	p11	p12	p13	p14	p15	p16	p17	p18	p19	p20
IV_p1	0.98**	0.03	0.03	-0.05	0.05*	-0.00	0.03	0.02	0.04	0.01	0.00	0.00	-0.00	-0.01	0.01	0.09*	0.02	0.00	0.01	0.00**
	(0.01)	(0.02)	(0.02)	(0.02)	(0.02)	(0.01)	(0.02)	(0.02)	(0.02)	(0.02)	(0.00)	(0.01)	(0.00)	(0.03)	(0.02)	(0.04)	(0.03)	(0.00)	(0.02)	(0.00)
IV_p2	-0.00	0.87**	0.01	0.01	0.04*	-0.00	0.05**	0.00	0.03*	-0.01	-0.01	0.00	-0.00	-0.03	0.01	-0.01	0.01	-0.00	0.02	-0.00
	(0.00)	(0.01)	(0.01)	(0.02)	(0.02)	(0.01)	(0.01)	(0.02)	(0.02)	(0.02)	(0.00)	(0.01)	(0.00)	(0.02)	(0.01)	(0.03)	(0.02)	(0.00)	(0.01)	(0.00)
IV_p3	0.00	0.05**	0.96**	-0.01	0.00	0.01*	0.02	-0.01	-0.00	-0.00	-0.00	-0.00	0.00	-0.00	0.00	-0.00	0.04**	0.00	-0.01	0.00
	(0.00)	(0.01)	(0.01)	(0.01)	(0.01)	(0.00)	(0.01)	(0.01)	(0.01)	(0.01)	(0.00)	(0.00)	(0.00)	(0.01)	(0.01)	(0.02)	(0.01)	(0.00)	(0.01)	(0.00)
IV_p4	0.00	-0.00	-0.01	0.81**	0.03*	0.01*	-0.01	0.01	0.02	-0.01	0.00	0.00	0.00	-0.01	0.01	0.02	0.02	-0.00	-0.01	0.00
	(0.00)	(0.01)	(0.01)	(0.01)	(0.01)	(0.01)	(0.01)	(0.01)	(0.01)	(0.01)	(0.00)	(0.00)	(0.00)	(0.01)	(0.01)	(0.02)	(0.01)	(0.00)	(0.01)	(0.00)
IV_p5	0.00	0.03*	0.01	0.04*	0.81**	-0.01	-0.01	-0.02	-0.03	-0.04*	0.00	0.00	-0.00	0.01	0.02	-0.01	0.02	-0.00	0.01	-0.00*
	(0.01)	(0.02)	(0.02)	(0.02)	(0.02)	(0.01)	(0.02)	(0.02)	(0.02)	(0.02)	(0.00)	(0.01)	(0.00)	(0.02)	(0.01)	(0.03)	(0.02)	(0.00)	(0.01)	(0.00)
IV_p6	0.01	-0.07*	-0.02	0.03	-0.03	1.00**	-0.12**	-0.06	-0.04	0.02	0.02**	0.01	0.00	-0.01	-0.04	-0.15*	-0.04	-0.00	-0.02	0.02**
	(0.01)	(0.03)	(0.03)	(0.04)	(0.04)	(0.02)	(0.03)	(0.04)	(0.04)	(0.04)	(0.01)	(0.01)	(0.01)	(0.04)	(0.02)	(0.07)	(0.04)	(0.00)	(0.03)	(0.00)
IV_p7	0.00	0.01	0.01	0.03	-0.01	0.01*	0.89**	0.00	-0.00	-0.02	-0.00	-0.00	0.00	0.02	-0.05**	0.01	-0.06**	-0.00	0.01	-0.00**
	(0.01)	(0.02)	(0.02)	(0.02)	(0.02)	(0.01)	(0.02)	(0.02)	(0.02)	(0.02)	(0.00)	(0.01)	(0.00)	(0.02)	(0.01)	(0.03)	(0.02)	(0.00)	(0.01)	(0.00)
IV_p8	0.00	0.01	0.00	0.03*	0.02	-0.01*	0.04**	0.97**	-0.01	-0.00	0.00	-0.01	-0.00	0.02	0.00	-0.02	0.00	0.00	0.01	-0.00**
	(0.00)	(0.01)	(0.01)	(0.01)	(0.01)	(0.01)	(0.01)	(0.01)	(0.01)	(0.01)	(0.00)	(0.00)	(0.00)	(0.01)	(0.01)	(0.02)	(0.01)	(0.00)	(0.01)	(0.00)
IV_p9	0.00	-0.02	-0.05	-0.08*	-0.07*	-0.00	-0.00	-0.00	0.50**	-0.02	-0.00	0.00	-0.01	-0.02	-0.02	-0.10	-0.03	0.00	-0.00	0.00
	(0.01)	(0.03)	(0.03)	(0.03)	(0.03)	(0.01)	(0.03)	(0.03)	(0.03)	(0.03)	(0.01)	(0.01)	(0.01)	(0.03)	(0.02)	(0.06)	(0.03)	(0.00)	(0.02)	(0.00)
IV_p10	0.00	-0.02*	-0.01	0.00	-0.03*	0.01	-0.01	-0.00	0.02	0.91**	0.00	-0.00	0.00	0.00	0.01	0.06**	0.01	-0.00	0.02*	0.00
	(0.00)	(0.01)	(0.01)	(0.01)	(0.01)	(0.00)	(0.01)	(0.01)	(0.01)	(0.01)	(0.00)	(0.00)	(0.00)	(0.01)	(0.01)	(0.02)	(0.01)	(0.00)	(0.01)	(0.00)
IV_p11	-0.00	-0.09	0.04	0.12*	0.05	0.00	0.03	-0.04	-0.09	0.11*	0.95**	0.00	-0.01	0.10	0.02	0.12	-0.03	0.00	0.06	0.01**
	(0.02)	(0.05)	(0.05)	(0.06)	(0.05)	(0.02)	(0.05)	(0.05)	(0.05)	(0.05)	(0.01)	(0.02)	(0.01)	(0.06)	(0.04)	(0.10)	(0.06)	(0.01)	(0.04)	(0.00)
IV_p12	0.00	0.01	0.00	-0.01	0.01	-0.01	0.02*	-0.01	0.00	-0.01	0.00	1.03**	-0.00	0.00	-0.01	-0.06*	0.01	0.00	0.01	0.00
	(0.00)	(0.01)	(0.01)	(0.01)	(0.01)	(0.00)	(0.01)	(0.01)	(0.01)	(0.01)	(0.00)	(0.00)	(0.00)	(0.01)	(0.01)	(0.02)	(0.01)	(0.00)	(0.01)	(0.00)
IV_p13	-0.00	-0.00	0.02	0.01	-0.04	0.01	0.02	0.04	0.08**	0.00	0.00	-0.01	1.02**	-0.01	0.00	0.10*	0.02	0.00	0.06**	0.00**
	(0.01)	(0.02)	(0.02)	(0.02)	(0.02)	(0.01)	(0.02)	(0.02)	(0.02)	(0.02)	(0.00)	(0.01)	(0.00)	(0.02)	(0.01)	(0.04)	(0.02)	(0.00)	(0.02)	(0.00)
IV_p14	-0.00	-0.03**	-0.00	-0.03*	0.01	-0.01	-0.00	0.01	0.01	-0.01	0.00	-0.00	-0.00	0.96**	-0.01	-0.01	0.02	-0.00	0.02	0.00
	(0.00)	(0.01)	(0.01)	(0.01)	(0.01)	(0.00)	(0.01)	(0.01)	(0.01)	(0.01)	(0.00)	(0.00)	(0.00)	(0.01)	(0.01)	(0.02)	(0.01)	(0.00)	(0.01)	(0.00)
IV_p15	-0.00	0.02	0.01	-0.00	0.02	0.00	0.05**	-0.00	0.02	0.02	0.00	-0.00	0.00	-0.01	1.03**	-0.02	-0.00	0.00	0.00	0.00
	(0.00)	(0.01)	(0.01)	(0.02)	(0.02)	(0.01)	(0.01)	(0.02)	(0.02)	(0.02)	(0.00)	(0.01)	(0.00)	(0.02)	(0.01)	(0.03)	(0.02)	(0.00)	(0.01)	(0.00)
IV_p16	-0.00	0.01	0.00	0.02	-0.01	-0.01	-0.01	-0.00	-0.04**	-0.00	-0.00	-0.01	-0.00	-0.01	-0.01	0.52**	0.00	0.00	-0.00	0.00
	(0.00)	(0.01)	(0.01)	(0.01)	(0.01)	(0.01)	(0.01)	(0.01)	(0.01)	(0.01)	(0.00)	(0.00)	(0.00)	(0.02)	(0.01)	(0.03)	(0.02)	(0.00)	(0.01)	(0.00)
IV_p17	-0.00	-0.00	0.00	-0.01	0.05*	0.01	0.00	-0.00	0.01	-0.02	-0.00	-0.00	0.00	-0.04	0.00	0.04	0.91**	-0.00	-0.00	0.00*
	(0.01)	(0.02)	(0.02)	(0.02)	(0.02)	(0.01)	(0.02)	(0.02)	(0.02)	(0.02)	(0.00)	(0.01)	(0.00)	(0.02)	(0.01)	(0.04)	(0.02)	(0.00)	(0.02)	(0.00)
IV_p18	0.01	0.04	0.04	0.00	-0.02	0.00	0.01	-0.01	0.14**	0.03	0.01*	0.01	0.01	-0.01	0.02	0.08	-0.03	0.99**	-0.06*	0.00
	(0.01)	(0.03)	(0.03)	(0.04)	(0.03)	(0.01)	(0.03)	(0.03)	(0.04)	(0.04)	(0.01)	(0.01)	(0.01)	(0.04)	(0.02)	(0.07)	(0.04)	(0.00)	(0.03)	(0.00)
IV_p19	0.01	0.05	-0.00	0.03	0.01	-0.02	0.02	0.04	-0.04	0.02	0.00	0.01	0.00	0.05	0.02	0.04	-0.07	0.00	0.84**	0.00
	(0.01)	(0.04)	(0.04)	(0.05)	(0.05)	(0.02)	(0.04)	(0.05)	(0.05)	(0.05)	(0.01)	(0.02)	(0.01)	(0.05)	(0.03)	(0.09)	(0.05)	(0.00)	(0.04)	(0.00)
IV_p20	0.24**	0.92**	0.36**	0.72**	0.47**	0.17**	0.77**	0.22	0.48**	0.36**	0.23**	0.04	0.04	0.04	0.17*	0.35	0.45**	0.01	0.18	1.03**
	(0.04)	(0.11)	(0.11)	(0.13)	(0.12)	(0.05)	(0.11)	(0.12)	(0.12)	(0.12)	(0.02)	(0.04)	(0.02)	(0.13)	(0.08)	(0.22)	(0.13)	(0.01)	(0.10)	(0.00)
F-stats	3,142.98	1,005.37	1,219.42	212.81	1,619.53	833.37	1,037.25	764.92	2,578.30	806.49	9,980.38	3,640.61	149,716.00	555.92	1,344.94	572.66	5,495.14	97,160.10	1,110.90	193,435.00
p-value	0.00	0.00	0.00	0.00	0.00	0.00	0.00	0.00	0.00	0.00	0.00	0.00	0.00	0.00	0.00	0.00	0.00	0.00	0.00	0.00
Obs.	14,420	14,420	14,420	14,420	14,420	14,420	14,420	14,420	14,420	14,420	14,420	14,420	14,420	14,420	14,420	14,420	14,420	14,420	14,420	14,420

Note: p_j denotes the price for food group j, and IV_p_j denotes the instrument for p_j, which is based on the average price index of neighboring households. Due to space limits, we report the coefficients on price instruments only. The regressions also include the vector of demand shifters. The F-stat checks for joint significance of all price instruments in a two-sided test. We report standard errors in parentheses.

Suppl. Table 59: Tanzania: Results from first stage instrumental variable regressions

	p1	p2	p3	p4	p5	p6	p7	p8	p9	p10	p11	p12	p13	p14	p15	p16	p17	p18	p19	p20
IV_p1	0.88**	0.03	-0.03	0.00	0.00	0.08**	0.05*	0.01	0.01	-0.01	0.01	0.01	-0.01	0.00	-0.04	-0.05	-0.05	-0.03	0.01	0.01**
	(0.02)	(0.03)	(0.03)	(0.02)	(0.02)	(0.02)	(0.02)	(0.02)	(0.04)	(0.03)	(0.01)	(0.01)	(0.01)	(0.04)	(0.03)	(0.03)	(0.05)	(0.03)	(0.05)	(0.00)
IV_p2	0.02	1.00**	-0.01	0.00	0.02	0.00	0.01	-0.01	0.04	-0.02	-0.02*	-0.03**	-0.00	0.05	0.02	-0.00	0.00	0.07**	-0.01	-0.00*
	(0.01)	(0.02)	(0.02)	(0.01)	(0.02)	(0.01)	(0.01)	(0.01)	(0.02)	(0.02)	(0.01)	(0.01)	(0.01)	(0.03)	(0.02)	(0.02)	(0.03)	(0.02)	(0.03)	(0.00)
IV_p3	-0.04*	-0.03	0.84**	-0.04*	-0.06**	-0.01	0.03	-0.03	-0.05	-0.05*	-0.01	0.01	-0.01	0.03	-0.06*	-0.06	-0.03	-0.02	-0.07	-0.00*
	(0.02)	(0.02)	(0.02)	(0.02)	(0.02)	(0.02)	(0.02)	(0.02)	(0.03)	(0.02)	(0.01)	(0.01)	(0.01)	(0.03)	(0.02)	(0.03)	(0.05)	(0.03)	(0.04)	(0.00)
IV_p4	0.00	0.01	0.00	0.98**	0.01	0.01	-0.02	0.00	0.02	0.01	0.00	-0.01	0.00	-0.03	0.00	0.01	-0.03	0.03*	-0.04	-0.00**
	(0.01)	(0.01)	(0.01)	(0.01)	(0.01)	(0.01)	(0.01)	(0.01)	(0.02)	(0.01)	(0.01)	(0.01)	(0.00)	(0.02)	(0.01)	(0.02)	(0.02)	(0.01)	(0.02)	(0.00)
IV_p5	-0.00	0.02	-0.04*	-0.00	0.92**	0.02	0.03*	0.01	-0.03	-0.02	-0.01	-0.02*	-0.01*	-0.02	-0.04*	-0.03	0.06	-0.01	0.03	0.00**
	(0.01)	(0.02)	(0.02)	(0.01)	(0.02)	(0.01)	(0.01)	(0.01)	(0.02)	(0.02)	(0.01)	(0.01)	(0.01)	(0.03)	(0.02)	(0.02)	(0.03)	(0.02)	(0.03)	(0.00)
IV_p6	0.07**	0.02	0.02	0.01	0.03	0.79**	0.00	0.04*	-0.08*	0.05	0.02	0.00	0.00	0.09*	0.13**	0.08*	0.04	0.03	0.14**	0.01**
	(0.02)	(0.03)	(0.03)	(0.02)	(0.02)	(0.02)	(0.02)	(0.02)	(0.04)	(0.03)	(0.01)	(0.02)	(0.01)	(0.04)	(0.03)	(0.03)	(0.05)	(0.03)	(0.05)	(0.00)
IV_p7	0.04	0.00	0.04	-0.02	0.05	0.01	0.69**	-0.00	0.05	0.05	0.02	0.01	0.01	-0.08	0.04	-0.04	-0.07	0.10*	-0.09	0.01**
	(0.03)	(0.03)	(0.03)	(0.03)	(0.03)	(0.03)	(0.03)	(0.03)	(0.05)	(0.04)	(0.02)	(0.02)	(0.01)	(0.05)	(0.04)	(0.04)	(0.07)	(0.04)	(0.06)	(0.00)
IV_p8	0.00	0.01	-0.03*	0.02	-0.00	0.03**	-0.00	1.00**	-0.00	0.01	0.01	0.00	0.00	0.03	0.02	0.01	-0.02	-0.00	0.00	-0.00
	(0.01)	(0.01)	(0.01)	(0.01)	(0.01)	(0.01)	(0.01)	(0.01)	(0.01)	(0.01)	(0.01)	(0.01)	(0.00)	(0.02)	(0.01)	(0.01)	(0.02)	(0.01)	(0.02)	(0.00)
IV_p9	0.01	-0.00	-0.03	-0.02	-0.00	-0.05*	0.00	-0.03	0.70**	-0.01	0.01	0.00	0.00	0.03	0.01	0.02	-0.01	-0.04	0.03	-0.00
	(0.02)	(0.02)	(0.02)	(0.02)	(0.02)	(0.02)	(0.02)	(0.02)	(0.03)	(0.02)	(0.01)	(0.01)	(0.01)	(0.03)	(0.02)	(0.03)	(0.04)	(0.03)	(0.04)	(0.00)
IV_p10	0.00	-0.03*	0.01	0.00	-0.00	0.01	0.01	0.01	0.06**	0.90**	-0.02*	-0.01	-0.01	-0.01	-0.02	0.03	-0.00	0.01	0.01	0.00
	(0.01)	(0.01)	(0.01)	(0.01)	(0.01)	(0.01)	(0.01)	(0.01)	(0.02)	(0.01)	(0.01)	(0.01)	(0.00)	(0.02)	(0.01)	(0.02)	(0.03)	(0.02)	(0.02)	(0.00)
IV_p11	0.02	-0.06	0.02	-0.01	-0.03	0.01	0.03	0.04	0.09*	-0.05	0.88**	-0.04*	-0.03**	0.00	-0.01	0.07	0.08	0.15**	0.06	-0.01**
	(0.02)	(0.03)	(0.03)	(0.03)	(0.03)	(0.03)	(0.02)	(0.03)	(0.04)	(0.03)	(0.02)	(0.02)	(0.01)	(0.05)	(0.03)	(0.04)	(0.06)	(0.04)	(0.06)	(0.00)
IV_p12	-0.00	-0.01	-0.00	0.00	0.00	-0.00	-0.01	-0.01	-0.00	0.00	0.00	1.00**	0.00	0.03	-0.01	0.01	-0.02	-0.04**	0.04	-0.00
	(0.01)	(0.01)	(0.01)	(0.01)	(0.01)	(0.01)	(0.01)	(0.01)	(0.01)	(0.01)	(0.01)	(0.01)	(0.00)	(0.02)	(0.01)	(0.01)	(0.02)	(0.01)	(0.02)	(0.00)
IV_p13	-0.00	0.02**	-0.00	-0.00	-0.00	-0.01*	0.01**	0.01*	-0.01	-0.01	-0.00	-0.00	1.00**	0.00	0.00	0.02**	0.01	0.02*	0.02	-0.00**
	(0.00)	(0.01)	(0.01)	(0.00)	(0.01)	(0.00)	(0.00)	(0.00)	(0.01)	(0.01)	(0.00)	(0.00)	(0.00)	(0.01)	(0.01)	(0.01)	(0.01)	(0.01)	(0.01)	(0.00)
IV_p14	0.00	0.03*	0.02	-0.01	-0.01	0.02	-0.02*	-0.01	0.01	-0.01	-0.00	0.01	0.01	0.78**	-0.01	0.05**	0.02	-0.02	-0.05*	-0.00**
	(0.01)	(0.01)	(0.01)	(0.01)	(0.01)	(0.01)	(0.01)	(0.01)	(0.02)	(0.02)	(0.01)	(0.01)	(0.00)	(0.02)	(0.02)	(0.02)	(0.03)	(0.02)	(0.03)	(0.00)
IV_p15	-0.02*	-0.01	-0.00	-0.00	-0.00	0.03**	-0.00	-0.00	0.02	0.02	-0.01	-0.00	0.00	-0.02	0.89**	0.02	0.03	0.01	0.01	-0.00
	(0.01)	(0.01)	(0.01)	(0.01)	(0.01)	(0.01)	(0.01)	(0.01)	(0.02)	(0.01)	(0.01)	(0.01)	(0.00)	(0.02)	(0.01)	(0.01)	(0.02)	(0.01)	(0.02)	(0.00)
IV_p16	-0.04	0.06*	-0.01	-0.00	-0.01	0.01	-0.01	0.01	0.01	0.01	0.02	-0.03	0.00	0.08	-0.01	0.51**	-0.24**	0.00	-0.09	0.01**
	(0.02)	(0.03)	(0.03)	(0.02)	(0.03)	(0.03)	(0.02)	(0.02)	(0.04)	(0.03)	(0.02)	(0.02)	(0.01)	(0.04)	(0.03)	(0.04)	(0.06)	(0.04)	(0.05)	(0.00)
IV_p17	-0.01	0.01	0.00	0.02	0.02	0.01	0.00	0.00	0.01	0.05**	0.02*	0.02*	0.00	0.03	0.02	-0.04**	0.84**	-0.01	0.00	0.00*
	(0.01)	(0.01)	(0.01)	(0.01)	(0.01)	(0.01)	(0.01)	(0.01)	(0.02)	(0.01)	(0.01)	(0.01)	(0.00)	(0.02)	(0.01)	(0.02)	(0.02)	(0.02)	(0.02)	(0.00)
IV_p18	-0.00	0.01	-0.01	-0.00	0.01	-0.00	0.01	-0.00	-0.02	0.01	0.01**	0.00	0.00	0.01	0.02*	-0.01	-0.03*	0.92**	0.00	-0.00
	(0.01)	(0.01)	(0.01)	(0.01)	(0.01)	(0.01)	(0.01)	(0.01)	(0.01)	(0.01)	(0.00)	(0.00)	(0.00)	(0.01)	(0.01)	(0.01)	(0.02)	(0.01)	(0.01)	(0.00)
IV_p19	-0.02	-0.02	-0.02	-0.02	-0.01	0.04*	-0.03*	-0.02	0.02	-0.04*	0.01	-0.01	-0.00	-0.06*	0.01	-0.02	0.04	0.06*	0.70**	-0.00**
	(0.01)	(0.02)	(0.02)	(0.02)	(0.02)	(0.02)	(0.01)	(0.01)	(0.02)	(0.02)	(0.01)	(0.01)	(0.01)	(0.03)	(0.02)	(0.02)	(0.04)	(0.02)	(0.03)	(0.00)
IV_p20	0.08	0.05	0.22	0.14	0.03	0.22*	0.17*	-0.03	0.06	0.04	0.09	0.24**	0.07	0.40*	0.06	0.72**	0.24	-0.20	0.25	1.01**
	(0.09)	(0.11)	(0.11)	(0.10)	(0.11)	(0.10)	(0.09)	(0.09)	(0.16)	(0.12)	(0.06)	(0.07)	(0.04)	(0.17)	(0.12)	(0.15)	(0.23)	(0.14)	(0.20)	(0.00)
F-stats	145.18	324.58	84.36	513.09	243.26	159.25	125.59	576.31	59.31	212.25	486.46	865.07	34,746.50	131.98	531.11	53.86	86.97	513.38	50.32	24,830.50
p-value	0.00	0.00	0.00	0.00	0.00	0.00	0.00	0.00	0.00	0.00	0.00	0.00	0.00	0.00	0.00	0.00	0.00	0.00	0.00	0.00
Obs.	9,196	9,196	9,196	9,196	9,196	9,196	9,196	9,196	9,196	9,196	9,196	9,196	9,196	9,196	9,196	9,196	9,196	9,196	9,196	9,196

Note: p_j denotes the price for food group j, and IV_p_j denotes the instrument for p_j, which is based on the average price index of neighboring households. Due to space limits, we report the coefficients on price instruments only. The regressions also include the vector of demand shifters. The F-stat checks for joint significance of all price instruments in a two-sided test. We report standard errors in parentheses.

Suppl. Table 60: Nigeria: Results from first stage instrumental variable regressions

	p1	p2	p3	p4	p5	p6	p7	p8	p9	p10	p11	p12	p13	p14	p15	p16	p17	p18	p19	p20
IV_p1	0.95**	-0.00	0.02	0.00	0.00	-0.01	0.06**	-0.01	0.01	-0.00	0.00	-0.00	0.00	0.00	-0.01	-0.01	-0.03*	-0.00	0.04	0.00**
	(0.01)	(0.01)	(0.01)	(0.01)	(0.01)	(0.02)	(0.01)	(0.01)	(0.02)	(0.01)	(0.01)	(0.00)	(0.00)	(0.02)	(0.01)	(0.01)	(0.01)	(0.01)	(0.03)	(0.00)
IV_p2	0.00	0.99**	0.00	0.01	0.01	0.01*	0.01**	0.00	0.01*	0.00	-0.01*	-0.00	0.00	0.01*	0.00	0.00	0.02**	0.01**	0.00	-0.00
	(0.00)	(0.00)	(0.00)	(0.00)	(0.00)	(0.01)	(0.00)	(0.00)	(0.01)	(0.00)	(0.00)	(0.00)	(0.00)	(0.01)	(0.00)	(0.00)	(0.01)	(0.00)	(0.01)	(0.00)
IV_p3	0.02*	0.00	0.97**	0.02	-0.01	-0.01	-0.01	0.00	0.00	-0.01	0.02*	0.00	0.00	-0.02	-0.00	0.00	-0.00	0.00	-0.04	-0.00**
	(0.01)	(0.01)	(0.01)	(0.01)	(0.01)	(0.02)	(0.01)	(0.01)	(0.01)	(0.01)	(0.01)	(0.00)	(0.00)	(0.01)	(0.01)	(0.01)	(0.01)	(0.01)	(0.03)	(0.00)
IV_p4	0.01	0.01	0.01	0.98**	0.00	-0.00	0.01	-0.00	0.00	0.01	0.00	0.00	0.00	-0.00	-0.01	-0.00	0.01	0.01	-0.01	-0.00*
	(0.01)	(0.01)	(0.01)	(0.01)	(0.01)	(0.01)	(0.01)	(0.01)	(0.01)	(0.01)	(0.01)	(0.00)	(0.00)	(0.01)	(0.01)	(0.01)	(0.01)	(0.01)	(0.02)	(0.00)
IV_p5	0.00	0.01*	0.01	0.01	0.99**	0.02	0.02**	0.01	0.01	0.00	0.00	-0.00	0.00	0.03**	-0.02*	0.00	-0.02*	-0.00	-0.02	-0.00**
	(0.01)	(0.00)	(0.01)	(0.01)	(0.01)	(0.01)	(0.01)	(0.01)	(0.01)	(0.01)	(0.01)	(0.00)	(0.00)	(0.01)	(0.01)	(0.01)	(0.01)	(0.01)	(0.02)	(0.00)
IV_p6	0.00	0.00	0.00	-0.00	0.01	1.00**	0.03**	0.00	0.02*	0.01*	0.00	0.00	0.00	0.01	0.00	0.00	0.01	0.03**	0.03*	-0.00
	(0.00)	(0.00)	(0.00)	(0.01)	(0.01)	(0.01)	(0.01)	(0.01)	(0.01)	(0.00)	(0.00)	(0.00)	(0.00)	(0.01)	(0.01)	(0.00)	(0.01)	(0.01)	(0.01)	(0.00)
IV_p7	0.05**	0.01	-0.00	0.01	0.03*	0.05**	0.99**	0.00	-0.00	-0.00	0.01	0.00	-0.00	0.03*	-0.01	0.02*	-0.03*	-0.02	-0.01	0.00
	(0.01)	(0.01)	(0.01)	(0.01)	(0.01)	(0.01)	(0.01)	(0.01)	(0.01)	(0.01)	(0.01)	(0.00)	(0.00)	(0.01)	(0.01)	(0.01)	(0.01)	(0.01)	(0.02)	(0.00)
IV_p8	0.00	0.00*	0.01*	-0.00	0.00	0.01*	0.00	1.02**	0.00	-0.00	0.01**	0.00	-0.00	0.00	0.00	0.00	-0.01**	0.00	0.00	-0.00*
	(0.00)	(0.00)	(0.00)	(0.00)	(0.00)	(0.00)	(0.00)	(0.00)	(0.00)	(0.00)	(0.00)	(0.00)	(0.00)	(0.00)	(0.00)	(0.00)	(0.00)	(0.00)	(0.01)	(0.00)
IV_p9	0.00	0.00	0.01	-0.01	0.01	0.02*	0.00	-0.00	0.97**	0.01*	0.01	0.00	0.00	0.04**	0.03**	0.01	0.04**	0.02**	0.04*	-0.00*
	(0.01)	(0.01)	(0.01)	(0.01)	(0.01)	(0.01)	(0.01)	(0.01)	(0.01)	(0.01)	(0.01)	(0.00)	(0.00)	(0.01)	(0.01)	(0.01)	(0.01)	(0.01)	(0.02)	(0.00)
IV_p10	0.01*	0.01*	0.01*	0.02**	0.01	0.01	0.02**	0.01	0.04**	0.97**	0.00	0.00	-0.00	0.02**	0.02**	0.01	0.03**	-0.01	0.02	0.00*
	(0.01)	(0.00)	(0.00)	(0.01)	(0.01)	(0.01)	(0.01)	(0.01)	(0.01)	(0.01)	(0.01)	(0.00)	(0.00)	(0.01)	(0.01)	(0.00)	(0.01)	(0.01)	(0.02)	(0.00)
IV_p11	0.02*	0.00	0.01	0.01	0.01	-0.00	0.02	0.03*	-0.00	0.00	0.99**	0.00	0.00	0.07**	0.02*	-0.00	0.07**	0.04**	-0.01	-0.00
	(0.01)	(0.01)	(0.01)	(0.01)	(0.01)	(0.02)	(0.01)	(0.01)	(0.02)	(0.01)	(0.01)	(0.00)	(0.00)	(0.02)	(0.01)	(0.01)	(0.01)	(0.01)	(0.03)	(0.00)
IV_p12	-0.00	0.00	0.00	0.00	-0.01	0.01**	0.01*	0.00	0.01	-0.00	0.01	1.00**	0.00	0.01**	-0.01	-0.00	-0.00	-0.01**	0.00	0.00**
	(0.00)	(0.00)	(0.00)	(0.00)	(0.00)	(0.01)	(0.00)	(0.00)	(0.00)	(0.00)	(0.00)	(0.00)	(0.00)	(0.00)	(0.00)	(0.00)	(0.00)	(0.00)	(0.01)	(0.00)
IV_p13	0.00	0.00	-0.00	-0.01	0.01	0.02*	-0.00	-0.01*	0.01	-0.01	0.00	0.00	1.00**	-0.02	0.01	0.00	0.01	-0.01*	0.03	-0.00**
	(0.01)	(0.00)	(0.01)	(0.01)	(0.01)	(0.01)	(0.01)	(0.01)	(0.01)	(0.01)	(0.01)	(0.00)	(0.00)	(0.01)	(0.01)	(0.00)	(0.01)	(0.01)	(0.02)	(0.00)
IV_p14	-0.01	-0.00	-0.00	0.00	0.01	0.00	0.00	-0.00	0.03**	0.00	0.01	-0.00	-0.00	0.87**	-0.01	0.00	-0.01	-0.00	-0.00	0.00**
	(0.00)	(0.00)	(0.00)	(0.01)	(0.01)	(0.01)	(0.01)	(0.01)	(0.01)	(0.01)	(0.01)	(0.00)	(0.00)	(0.01)	(0.01)	(0.00)	(0.01)	(0.01)	(0.01)	(0.00)
IV_p15	0.01	0.00	0.00	0.00	0.01	0.00	0.02*	0.01	0.01	0.00	-0.01	-0.00	0.00	0.01	1.00**	-0.00	0.01	0.01	-0.03	-0.00*
	(0.01)	(0.01)	(0.01)	(0.01)	(0.01)	(0.01)	(0.01)	(0.01)	(0.01)	(0.01)	(0.01)	(0.00)	(0.00)	(0.01)	(0.01)	(0.01)	(0.01)	(0.01)	(0.02)	(0.00)
IV_p16	-0.01	0.00	0.01	-0.01	0.02	0.03	0.04**	-0.01	0.02	0.02	0.00	-0.00	0.00	0.05**	0.02	0.97**	0.05**	0.04*	-0.03	0.00**
	(0.01)	(0.01)	(0.01)	(0.01)	(0.02)	(0.02)	(0.01)	(0.01)	(0.02)	(0.01)	(0.01)	(0.00)	(0.01)	(0.02)	(0.01)	(0.01)	(0.02)	(0.01)	(0.04)	(0.00)
IV_p17	-0.01	0.00	0.00	-0.01	-0.00	-0.02**	-0.01	-0.01**	-0.00	0.00	-0.00	-0.00	-0.00	-0.00	-0.00	-0.00	1.01**	0.00	-0.01	0.00
	(0.00)	(0.00)	(0.00)	(0.00)	(0.00)	(0.01)	(0.00)	(0.00)	(0.01)	(0.00)	(0.00)	(0.00)	(0.00)	(0.01)	(0.00)	(0.00)	(0.00)	(0.00)	(0.01)	(0.00)
IV_p18	-0.02*	-0.00	0.01*	0.01	0.01	0.04**	0.00	0.01	0.01	0.01	0.01	-0.00	-0.01*	0.01	0.01	-0.00	0.01	0.97**	0.01	0.00
	(0.01)	(0.00)	(0.01)	(0.01)	(0.01)	(0.01)	(0.01)	(0.01)	(0.01)	(0.01)	(0.01)	(0.00)	(0.00)	(0.01)	(0.01)	(0.01)	(0.01)	(0.01)	(0.02)	(0.00)
IV_p19	-0.00	0.00	-0.00	-0.00	0.00	-0.00	0.00	-0.00	0.00	0.00	-0.00	-0.00	-0.00	-0.00	0.00	0.00	0.01	0.02**	0.96**	-0.00
	(0.00)	(0.00)	(0.00)	(0.00)	(0.00)	(0.01)	(0.00)	(0.00)	(0.01)	(0.00)	(0.00)	(0.00)	(0.00)	(0.01)	(0.00)	(0.00)	(0.00)	(0.00)	(0.01)	(0.00)
IV_p20	0.27	-0.02	0.35	0.56*	-0.41	0.44	-0.23	0.21	0.13	-0.26	0.28	0.06	0.13	0.61	-0.09	-0.14	-0.04	0.66*	1.01	0.99**
	(0.22)	(0.18)	(0.21)	(0.25)	(0.28)	(0.36)	(0.25)	(0.24)	(0.35)	(0.23)	(0.22)	(0.08)	(0.09)	(0.34)	(0.27)	(0.19)	(0.31)	(0.26)	(0.64)	(0.00)
F-stats	695.98	3,000.62	729.21	518.18	551.60	594.10	1,407.05	2,702.38	392.03	1,158.49	424.30	17,902.30	3,310.81	749.14	890.75	477.47	2,410.24	832.69	507.28	2,411,237.00
p-value	0.00	0.00	0.00	0.00	0.00	0.00	0.00	0.00	0.00	0.00	0.00	0.00	0.00	0.00	0.00	0.00	0.00	0.00	0.00	0.00
Obs.	25,977	25,977	25,977	25,977	25,977	25,977	25,977	25,977	25,977	25,977	25,977	25,977	25,977	25,977	25,977	25,977	25,977	25,977	25,977	25,977

Note: p_j denotes the price for food group j, and IV_p_j denotes the instrument for p_j, which is based on the average price index of neighboring households. Due to space limits, we report the coefficients on price instruments only. The regressions also include the vector of demand shifters. The F-stat checks for joint significance of all price instruments in a two-sided test. We report standard errors in parentheses.

Suppl. Table 61: Association between residualized household preference variables and price instruments (Nigeria)

	Less preferred foods (1)	Cooking asset (2)	Information access (3)	FAFH Full meal (4)	FAFH Dish (5)	FAFH Snack (6)	FAFH Dairy (7)	FAFH Veg (8)	Mean (SD) of price IVs (9)
Constant	0.0153	-0.0007	0.0053	0.0047	0.0000	-0.0005	0.0094	0.0055	
	(1.9220)	(-0.0973)	(0.9752)	(0.6539)	(0.0087)	(-0.0660)	(1.7176)	(0.8878)	
ivp1	-0.0095	0.0142	0.0168	0.0411	0.0177	0.0456	-0.0479**	-0.0153	-0.0169
	(-0.2943)	(0.5704)	(0.7106)	(1.3328)	(0.9095)	(1.5308)	(-2.0304)	(-0.5806)	(0.2744)
ivp2	0.0508**	0.0108	-0.0128	0.0358**	0.0315**	-0.0082	-0.0040	-0.0142	-0.0664
	(3.1734)	(1.1502)	(-1.4140)	(3.0465)	(3.2717)	(-0.6517)	(-0.4018)	(-1.2922)	(0.4206)
ivp3	-0.0527	0.0081	0.0116	-0.0439	-0.0262	0.0260	-0.0289	-0.0162	-0.0184
	(-1.4831)	(0.2725)	(0.4197)	(-1.2819)	(-1.0313)	(0.7317)	(-1.0428)	(-0.5912)	(0.2521)
ivp4	-0.0317	0.0034	0.0100	-0.0040	-0.0025	0.0274	0.0398**	0.0097	-0.0363
	(-1.0696)	(0.1752)	(0.5996)	(-0.1740)	(-0.1354)	(1.0854)	(2.1230)	(0.4802)	(0.2639)
ivp5	-0.0303	0.0061	0.0152	-0.0014	0.0190	-0.0052	0.0115	0.0046	-0.0585
	(-1.3717)	(0.3684)	(0.9768)	(-0.0679)	(1.3799)	(-0.2460)	(0.7590)	(0.2514)	(0.2860)
ivp6	0.0007	0.0045	-0.0166	-0.0118	0.0037	-0.0245	-0.0132	-0.0240	-0.0421
	(0.0359)	(0.3612)	(-1.2723)	(-0.7595)	(0.2989)	(-1.3159)	(-1.0197)	(-1.6715)	(0.3656)
ivp7	-0.0023	-0.0246	-0.0061	-0.0099	-0.0226	-0.0282	-0.0040	0.0117	-0.0413
	(-0.0925)	(-1.5658)	(-0.4082)	(-0.4985)	(-1.4522)	(-1.2579)	(-0.2322)	(0.6088)	(0.4170)
ivp8	0.0224	-0.0026	0.0107	0.0114	-0.0020	0.0034	0.0250**	0.0051	-0.0912
	(1.6308)	(-0.2840)	(1.3683)	(0.9648)	(-0.2315)	(0.2896)	(2.6569)	(0.5535)	(0.5385)
ivp9	0.1700**	0.0024	0.0260	0.0456	0.0047	0.0091	0.0619**	0.0132	-0.0983
	(6.6436)	(0.1142)	(1.5032)	(1.7822)	(0.2784)	(0.3819)	(3.1941)	(0.6273)	(0.2635)
ivp10	-0.0034	0.0047	0.0141	0.0114	0.0071	-0.0044	-0.0193	0.0101	-0.0968
	(-0.1731)	(0.3503)	(1.3031)	(0.7037)	(0.6285)	(-0.2848)	(-1.6090)	(0.6853)	(0.3333)
ivp11	-0.0273	0.0491	-0.0207	-0.0026	-0.0006	0.0028	-0.0366	-0.0460	0.0008
	(-0.6332)	(1.6907)	(-0.8340)	(-0.0760)	(-0.0183)	(0.0763)	(-1.2371)	(-1.4641)	(0.1925)
ivp12	-0.0104	0.0010	0.0022	-0.0135	-0.0183	-0.0107	-0.0168	-0.0152	-0.0429
	(-0.7437)	(0.0957)	(0.2494)	(-1.1333)	(-1.9326)	(-0.8573)	(-1.9212)	(-1.3143)	(0.4360)
ivp13	-0.0253	-0.0139	0.0049	-0.0203	-0.0017	0.0311	0.0351**	0.0176	-0.0292
	(-0.9597)	(-0.8186)	(0.2753)	(-0.8844)	(-0.1098)	(1.4300)	(2.1347)	(0.9258)	(0.2230)
ivp14	-0.0358**	-0.0014	0.0182	-0.0056	0.0041	0.0076	0.0046	0.0205	-0.1038
	(-2.0115)	(-0.0949)	(1.6625)	(-0.3587)	(0.3046)	(0.5045)	(0.3304)	(1.5288)	(0.3966)
ivp15	-0.0442	-0.0116	-0.0030	-0.0452	0.0433**	0.0332	0.0080	0.0318	-0.0214
	(-1.4869)	(-0.4970)	(-0.1447)	(-1.5917)	(2.2275)	(1.1018)	(0.3309)	(1.3144)	(0.3457)
ivp16	0.1005**	0.0057	0.0325	0.0055	0.0022	-0.0783**	-0.0380	0.0558	-0.0119
	(2.2306)	(0.1837)	(1.1103)	(0.1505)	(0.0899)	(-2.2186)	(-1.2892)	(1.7281)	(0.1843)
ivp17	0.0508**	-0.0168	0.0192	0.0040	-0.0149	-0.0075	0.0301**	0.0149	-0.1254
	(3.3463)	(-1.3283)	(1.7268)	(0.2847)	(-1.4293)	(-0.5047)	(2.7583)	(1.2433)	(0.6260)
ivp18	-0.0117	0.0139	-0.0437**	-0.0320	-0.0132	-0.0172	-0.0030	0.0131	-0.0438
	(-0.5126)	(0.9835)	(-3.4641)	(-1.6714)	(-0.8691)	(-0.8343)	(-0.1492)	(0.6963)	(0.3220)
ivp19	-0.0090	0.0013	-0.0086	-0.0045	-0.0086	0.0001	-0.0026	0.0013	-0.1659
	(-0.8961)	(0.1865)	(-1.4486)	(-0.5027)	(-1.5527)	(0.0170)	(-0.3723)	(0.1916)	(0.6572)
ivp20	-0.1257	0.0053	-0.0662	0.0757	0.0152	0.0145	-0.0398	-0.1149	-0.0158
	(-1.6726)	(0.0880)	(-1.3223)	(1.1091)	(0.2877)	(0.2245)	(-0.7258)	(-1.8958)	(0.1774)
Mean of dep var (res.)	0.0023	-0.0100	-0.0083	-0.0045	-0.0030	-0.0042	-0.0063	-0.0091	
SD of dep var (res.)	0.4589	0.3668	0.3948	0.4595	0.3661	0.4497	0.3574	0.3839	
R-squared	0.010	0.001	0.003	0.002	0.002	0.002	0.005	0.002	
F-statistic	4.75	0.83	1.73	1.42	1.41	0.94	2.91	1.24	
Observations	23171	23277	23277	23303	23303	23303	23303	23303	

Note: This table shows the results of eight separate regressions that provide suggestive evidence in support of the price IV exclusion restrictions. Columns 1 through 3 depict the regression of food preference variables (residualized using the demand system expenditure variables and demand shifters as described in the text) on the vector of log price instruments. Columns 4 through 8 provide the same for food away from home consumption variables. The 9th column depicts the mean and standard deviation of each log price residual.

Suppl. Table 62: Mapping of food groups into Price discount (PD) categories within each country

	Staple Grains	Starchy Staples	Pulses & Nuts	Fruits & Vegetables	Animal-Source Foods
**Malawi**	Rice; Maize; Wheat & other cereals	Cassava; Roots, tubers & other starches	Pulses; Nuts & seeds	Fruits; Vegetables	Red meat; Poultry; Eggs; Dairy
**Niger**	Rice; Millet; Wheat & other cereals	Cassava; Roots, tubers & other starches	Pulses; Nuts & seeds	Fruits; Vegetables	Red meat; Poultry; Eggs; Fish & seafood
**Uganda**	Rice; Maize; Wheat & other cereals	Cassava; Roots, tubers & other starches	Pulses; Nuts & seeds	Fruits; Vegetables	Red meat; Poultry; Eggs; Fish & seafood
**Tanzania**	Rice; Maize; Wheat & other cereals	Cassava; Roots, tubers & other starches	Pulses; Nuts & seeds	Fruits; Vegetables	Red meat; Poultry; Eggs; Fish & seafood; Dairy
**Nigeria**	Rice; Maize; Wheat & other cereals	Cassava; Roots, tubers & other starches	Pulses; Nuts & seeds	Fruits; Vegetables	Red meat; Poultry; Eggs; Fish & seafood; Dairy

Note: This table shows the food groups included in each PD simulation.
