# Supplementary material for: Nutrient adequacy for poor households in Africa would improve with higher income but not necessarily with lower food prices
Source: Nat Food. 2024 Feb 21;5(2):171–81. doi: 10.1038/s43016-024-00927-w (PMC10896716; doi:10.1038/s43016-024-00927-w)
Supplement: Supplementary file 2 — Reporting Summary [file 43016_2024_927_MOESM2_ESM.pdf]

# nature portfolio

Corresponding author(s): Ellen McCullough

Last updated by author(s): Jan 10, 2024

# Reporting Summary

Nature Portfolio wishes to improve the reproducibility of the work that we publish. This form provides structure for consistency and transparency in reporting. For further information on Nature Portfolio policies, see our Editorial Policies and the Editorial Policy Checklist.

## Statistics

For all statistical analyses, confirm that the following items are present in the figure legend, table legend, main text, or Methods section.

| n/a | Confirmed | |
|---|---|---|
| ☐ | ☒ | The exact sample size (*n*) for each experimental group/condition, given as a discrete number and unit of measurement |
| ☒ | ☐ | A statement on whether measurements were taken from distinct samples or whether the same sample was measured repeatedly |
| ☐ | ☒ | The statistical test(s) used AND whether they are one- or two-sided *Only common tests should be described solely by name; describe more complex techniques in the Methods section.* |
| ☐ | ☒ | A description of all covariates tested |
| ☐ | ☒ | A description of any assumptions or corrections, such as tests of normality and adjustment for multiple comparisons |
| ☐ | ☒ | A full description of the statistical parameters including central tendency (e.g. means) or other basic estimates (e.g. regression coefficient) AND variation (e.g. standard deviation) or associated estimates of uncertainty (e.g. confidence intervals) |
| ☐ | ☒ | For null hypothesis testing, the test statistic (e.g. *F*, *t*, *r*) with confidence intervals, effect sizes, degrees of freedom and *P* value noted *Give P values as exact values whenever suitable.* |
| ☒ | ☐ | For Bayesian analysis, information on the choice of priors and Markov chain Monte Carlo settings |
| ☒ | ☐ | For hierarchical and complex designs, identification of the appropriate level for tests and full reporting of outcomes |
| ☒ | ☐ | Estimates of effect sizes (e.g. Cohen's *d*, Pearson's *r*), indicating how they were calculated |

*Our web collection on statistics for biologists contains articles on many of the points above.*

## Software and code

Policy information about availability of computer code

| | |
|---|---|
| Data collection | N/A (this study did not collect data) |
| Data analysis | We used Stata 16 to clean and prepare survey data, run policy simulations, and create visuals. We used SAS 9.4 to estimate the demand system. We used R Studio version 2022.12.0 to create visuals. Code to replicate all of the analysis reported in this manuscript are available through the Harvard Dataverse. |

For manuscripts utilizing custom algorithms or software that are central to the research but not yet described in published literature, software must be made available to editors and reviewers. We strongly encourage code deposition in a community repository (e.g. GitHub). See the Nature Portfolio guidelines for submitting code & software for further information.

## Data

Policy information about availability of data

All manuscripts must include a data availability statement. This statement should provide the following information, where applicable:

- Accession codes, unique identifiers, or web links for publicly available datasets
- A description of any restrictions on data availability
- For clinical datasets or third party data, please ensure that the statement adheres to our policy

The household data we use to model demand systems are available from the World Bank's Living Standards and Measurement Studies - Integrated Surveys in Agriculture website (https://www.worldbank.org/en/programs/lsms/initiatives/lsms-ISA). This study did not generate additional data.

## Research involving human participants, their data, or biological material

Policy information about studies with human participants or human data. See also policy information about sex, gender (identity/presentation), and sexual orientation and race, ethnicity and racism.

| | |
|---|---|
| Reporting on sex and gender | We use careful language around sex and gender. |
| Reporting on race, ethnicity, or other socially relevant groupings | See below. |
| Population characteristics | See below. |
| Recruitment | See below. |
| Ethics oversight | We were not required to file our study with IRB. We use publicly available observational data collected by others, and we are unable to identify households or individuals in our survey. |

Note that full information on the approval of the study protocol must also be provided in the manuscript.

# Field-specific reporting

Please select the one below that is the best fit for your research. If you are not sure, read the appropriate sections before making your selection.

☐ Life sciences ☒ Behavioural & social sciences ☐ Ecological, evolutionary & environmental sciences

For a reference copy of the document with all sections, see nature.com/documents/nr-reporting-summary-flat.pdf

# Behavioural & social sciences study design

All studies must disclose on these points even when the disclosure is negative.

| | |
|---|---|
| Study description | We use quantitative observational panel data to estimate the parameters of a consumer demand system for multiple goods. We implement the same methodology in five African countries. We use the demand system parameters to analyze the relationship between incomes, prices, and nutrient adequacy. |
| Research sample | We use data collected by national statistics offices. All datasets contain a nationally representative sample of urban and rural households for the full population. We used these data because they are the only set of internationally standardized household level panel surveys in Sub-Saharan Africa. More information is available at at https://www.worldbank.org/en/programs/lsms/initiatives/lsms-ISA. |
| Sampling strategy | The data use a stratified random sampling strategy, first selecting primary sampling units within major zone strata using the national population frame, The second stage involves creating a census of households within the primary sampling unit and randomly selecting households to include in the study. Sampling was conducted by each country's national statistics office in partnership with the World Bank. This study was not involved in sampling households. More information is available at at https://www.worldbank.org/en/programs/lsms/initiatives/lsms-ISA. |
| Data collection | We use secondary data collected by national statistics offices and made publicly available. The survey instruments are comprehensive as it is a multi-purpose, multi-topic survey. The questionnaires can be found at https://www.worldbank.org/en/programs/lsms/initiatives/lsms-ISA. There was no experimental condition. Data collectors and respondents were not aware of any hypotheses associated with this this study. |
| Timing | We use secondary data collected over multiple uninterrupted waves between 2008 and 2017. For Malawi the waves took place in 2010–11, 2012–13, and 2016–17. For Niger, data collection occurred durign 2011 and 2014. For Uganda, data collection occurred in 2005-06, 2009-10, 2011-12, 2013-14, 2015-16, 2018-19. For Tanzania, data collection occurred durign 2008-09, 2010-11, and 2012-13. For Nigeria data collection occurred in 2010-11, 2012-13, and 2015-16. |
| Data exclusions | We do not exclude observations from our analysis. |
| Non-participation | We do not have access to this information from the National Statistics Offices. |
| Randomization | It is not cost effective to randomly alter expenditure and food prices given the number of goods in our demand system (18018 per country). In order to estimate cross-price elasticities of demand it would require too many treatments. |

# Reporting for specific materials, systems and methods

We require information from authors about some types of materials, experimental systems and methods used in many studies. Here, indicate whether each material, system or method listed is relevant to your study. If you are not sure if a list item applies to your research, read the appropriate section before selecting a response.

## Materials & experimental systems

| n/a | Involved in the study |
|---|---|
| ☒ | ☐ Antibodies |
| ☒ | ☐ Eukaryotic cell lines |
| ☒ | ☐ Palaeontology and archaeology |
| ☒ | ☐ Animals and other organisms |
| ☒ | ☐ Clinical data |
| ☒ | ☐ Dual use research of concern |
| ☒ | ☐ Plants |

## Methods

| n/a | Involved in the study |
|---|---|
| ☒ | ☐ ChIP-seq |
| ☒ | ☐ Flow cytometry |
| ☒ | ☐ MRI-based neuroimaging |
